# A novel concept of photosynthetic soft membranes: a numerical study

**DOI:** 10.1186/s11671-023-03772-1

**Published:** 2023-02-09

**Authors:** Gabriele Falciani, Luca Bergamasco, Shannon A. Bonke, Indraneel Sen, Eliodoro Chiavazzo

**Affiliations:** 1 Department of Energy, Politecnico di Torino, Turin, Italy; 2 Yusuf Hamied Department of Chemistry, University of Cambridge, Cambridge, UK; 3 Department of Chemistry, Uppsala University, Uppsala, Sweden

**Keywords:** Solar fuels, Photosynthetic membranes, Self-assembly, Soap films, Surface science, Mass transfer

## Abstract

We focus on a novel concept of photosynthetic soft membranes, possibly able to allow the conversion of solar energy and carbon dioxide (CO$_2$) into green fuels. The considered membranes rely on self-assembled functional molecules in the form of soap films. We elaborate a multi-scale and multi-physics model to describe the relevant phenomena, investigating the expected performance of a single soft photosynthetic membrane. First, we present a macroscale continuum model, which accounts for the transport of gaseous and ionic species within the soap film, the chemical equilibria and the two involved photocatalytic half reactions of the CO$_2$ reduction and water oxidation at the two gas–surfactant–water interfaces of the soap film. Second, we introduce a mesoscale discrete Monte Carlo model, to deepen the investigation of the structure of the functional monolayers. Finally, the morphological information obtained at the mesoscale is integrated into the continuum model in a multi-scale framework. The developed tools are then used to perform sensitivity studies in a wide range of possible experimental conditions, to provide scenarios on fuel production by such a novel approach.

**Supplementary Information:**

The online version contains supplementary material available at 10.1186/s11671-023-03772-1.

## Background

The global population growth and the increasing energy demand pose serious challenges for a sustainable future. Utilization of fossil fuel resources and the emission in the atmosphere of greenhouse gases, carbon dioxide (CO$_2$) in particular, represent a major problem for climate change [1]. The Paris agreement, signed in 2016 by more than 170 nations, set ambitious targets for a reduction of CO$_2$ emissions, to the benefit of climate change mitigation [2]. It becomes then urgent to consider CO$_2$ as a potential resource instead of a mere polluting agent to eliminate, and to develop new technologies to convert it into valuable products [3]. In this view, here we focus on a recently suggested new approach for sustainable fuel production through photocatalytic conversion of the CO$_2$ into CO by exploiting solar radiation.

Current laboratory demonstrators of photocatalytic fuel production based on nanostructured solid-state materials have reached high solar-to-fuel energy conversion efficiency [4–6]. However, some critical bottlenecks still prevent proper development of commercially available technologies, such as the high cost of the materials, membrane aging, fuel–oxygen separation and proper use of the photoactive components [7, 8]. In aqueous phase devices, the fuel and the oxygen (O$_2$) microbubbles, that form and remain on the catalytic surfaces, reduce the photocatalytic activity [9]. In recent years, the use of photosynthetic assemblies, such as micelles [10, 11] and liposomes [12], to confine the reaction space has attracted increasing interest. Compartmentalization of molecular catalysts and photosensitizers improves indeed the electron transfer rates, by keeping both types of molecules at an interface and close enough to each other to allow their functional interaction [13].

A radically new concept of photosynthetic membranes has been recently proposed in the context of the European research project Sofia [14–16]. Here, the aim is to employ the photocatalytic properties of engineered surfactants in soft (soap film based) compartments for oxygen–fuel (i.e., carbon monoxide, CO) generation and separation. Soap films are composed of a water core stabilized by the presence of surfactant molecules that self-assemble at the gas–liquid interfaces [17, 18]. Ideally, a soap film membrane where molecular catalysts are self-assembled on water may avoid problems of photocatalytic-surface deactivation due to microbubble formation, keeping the produced oxygen and the fuel separated for a sufficiently long time. In the envisioned configuration, the soap film membrane separates two compartments filled with CO$_2$. The two half reactions of water oxidation and carbon dioxide reduction take place on the two opposite surfactant monolayers. The latter two half reactions are part of the desired overall photochemical reaction that will ultimately convert carbon dioxide into useful fuel using solar energy.

To our knowledge, the above new technology is still to be fully experimentally implemented in soap films. Nonetheless, half reactions have been observed in a number of self-assembled structures [10–12].

In this work, in an attempt to prepare a theoretical ground for the above new technology, we focus on the development of numerical models capable to incorporate the several physical and chemical phenomena occurring at multiple time and space scales that are expected to play an important role in soap film-based fuel generation. To this end, we propose a multi-scale and multi-physics framework to allow a better understanding of those processes. First, we introduce a continuum model, which accounts for the transport of gases through the soap film, the transport of charged species and the chemical equilibria in the water core (CO$_2$ dissociation and buffer), the adsorption and desorption of the gaseous species and chemical reactions in the two surfactant monolayers. Second, to investigate the main morphological features of the self-assembled photoactive molecules at the gas–liquid interfaces, we develop a discrete (coarse-grained) model based on a Metropolis Monte Carlo algorithm. The information on the structure of the monolayer at the mesoscale is then incorporated in the continuum model through an estimate of the resulting reaction constant.

We observe that the optimal conditions for fuel production depend on the interplay between the reactant species and the composition of the photoactive mixtures at the gas–surfactant–water interface, with a key role played by the electrostatic forces.

**Fig. 1:**
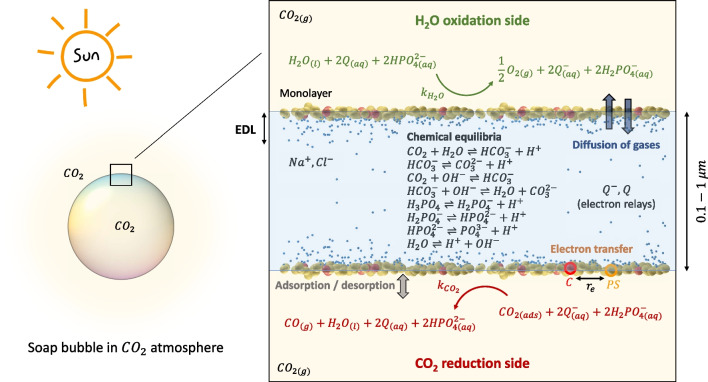
Physicochemical model of a (photo)reactive soap film. Soap bubbles (and soap films) are soft liquid membranes which are considered here for temporary gas compartmentalization. Soap films consist of a thin water core (typically ranging from few dozens of nanometers up to a few microns), decorated at the two interfaces by functional surfactant molecules that can, in principle, perform CO$_2$ reduction and H$_2$O oxidation when exposed to sunlight. These latter two half reactions are triggered by the interaction between photosensitizers (PS) and catalysts (C) and coupled by the diffusion of the electron relays (Q and Q$^-$) and of the phosphate buffer (which acts as a proton acceptor and donor) in the bulk. The gas solubilization, with the consequent CO$_2$ dissociation, occurs in the bulk where an equilibrium is established among water, gas and phosphate buffer. Electrostatic interactions are responsible for the development of an electric double layer (EDL) at the liquid side of the two surfactant monolayers, whereas Na$^+$ and Cl$^-$ guarantee the electroneutrality of the system. The produced gases tend to diffuse from one side to the other of the soap film membrane, driven by a concentration gradient resulting from the reactions

## Presentation of the hypothesis

The physicochemical processes occurring in the envisioned photocatalytic membranes are schematically reported in Fig. 1. The target overall photocatalytic conversion is that of CO$_2$ into CO. We assume the two surfactant monolayers of the soap film to be composed of photosensitizers (PS) and catalysts (C), which drive the two half reactions of water oxidation and carbon dioxide reduction. It is worth to remark that, in general, soap films are stabilized using base surfactants; however, in this case the PS (see the chemical structure of the considered ruthenium-based PS in Supplementary file 1: Fig. S1) is assumed to also play the role of a base surfactant [18]. In the photochemical reaction, the photosensitizer is responsible for light harvesting and electron transfer from or to the neighboring catalysts, which drives one of the two half reactions. We assume a Langmuir-type adsorption in the monolayer of the gaseous reactants. The two half reactions are coupled by the diffusion of the electron relays (Q and its reduced form Q$^-$) in the water core of the soap film, which allow electrons to transfer from one side to the other of the membrane. A concentration gradient, which drives the redox shuttles in the water core, is established due to the continuous consumption of Q at the water oxidation side and Q$^-$ at the CO$_2$ reduction side. Different types of electron relays could be used, both neutral and charged [19–22]. In this work, we assume a neutral electron relay that is reduced and becomes negatively charged when carrying an electron from the water oxidation side to the CO$_2$ reduction side. Protons are maintained in equilibrium owing to the presence of the phosphate buffer, which acts as a proton donor and acceptor. Electrostatic interactions between the charged surfactant monolayers and the ions in the bulk liquid are responsible for the development of an electric double layer (EDL); the Na$^+$ and Cl$^-$ contribute to the electroneutrality of the system.

## Testing the hypothesis

### Macroscale model of a photosynthetic soap film membrane

A continuum model representation is adopted for the transport of species across the soap film, as well as for the chemical equilibria and reactions. We adopt Fick’s diffusion to describe the transport of gases across the soap film, as proposed by Princen and coworkers [23–25]. A linear proportionality between the molar flux of the permeating gaseous species and the corresponding concentration difference across the soap film is defined as

1 $$\begin{aligned} \frac{\mathrm{d} n}{\mathrm{d} t} = k A_\mathrm{ML} (c_1-c_2), \end{aligned}$$

with *n* being the number of moles, *k* the soap film permeability, $A_\mathrm{ML}$ the surface area of the monolayer and $(c_1-c_2)$ the concentration difference across the film. In this model, the transport of gases is regulated by three mass transport resistances: those associated with the two surfactant monolayers, and that due to the thickness of the bulk liquid in the soap film. Thus, the soap film permeability can be defined as

2 $$\begin{aligned} k=\frac{H}{\frac{1}{k_\mathrm{ML1}} + \frac{1}{k_\mathrm{ML2}}+\frac{h}{D_w}}, \end{aligned}$$

with $k_\mathrm{ML1}$ and $k_\mathrm{ML2}$ being the monolayer permeability of the two surfactant monolayers forming the soap film, $D_w$ the diffusion coefficient of the gas in bulk water, *H* the Henry’s law constant and *h* the thickness of the water core. The adopted values for the diffusion coefficients and the Henry’s law constants are reported in Supplementary file 1: Tables S1–S3. These refer to the diffusion and the solubility of gases in pure water, which can be assumed for low bulk surfactant concentration [26]. As far as the gas permeation through the membrane is concerned, if the two surfactant monolayers present a different chemical structure, this is reflected in Eq. 2 as a disparity in the monolayer permeability values. The monolayer permeability $k_\mathrm{ML}$ is a parameter which encompasses all the microscopic interactions among the surfactant molecules at the gas–water interface and the permeating gases [25]. At the gas–surfactant–water interface, the molar rate of a given gas diffusing through a single surfactant monolayer is

3 $$\begin{aligned} \frac{\mathrm{d} n}{\mathrm{d} t} = k_\mathrm{ML} A_\mathrm{ML} ({\overline{c}}-c'), \end{aligned}$$

where $c'$ and ${\overline{c}}$ are the actual concentration and the equilibrium concentration of the considered gas in the liquid core of the film, respectively [24]. Equation 3 predicts a net flux, up to when equilibrium between the gas in solution and that in the gas phase is reached by Henry’s law as

4 $$\begin{aligned} {\overline{c}} = H c, \end{aligned}$$

with *c* denoting the actual concentration in the gas phase. Due to the lack of data on the gas diffusion through soap films made of photocatalytic surfactants, we assume the values for the monolayer permeability in [23], reported in Supplementary file 1: Table S4. However, it is important to note that, for sufficiently thick soap films like those analyzed in this work (i.e., > 200 nm), the monolayer permeability, $k_\mathrm{ML}$, has a negligible influence on the soap film permeability *k* [15]. Thick soap films are indeed required to keep the two produced gases (CO and O$_2$) separated for a sufficiently long time. This is typically in the order of dozens of seconds to dozens of minutes depending on the soap film thickness, ranging from hundreds of nanometers to microns [15].

We now introduce and discuss our representation for the photochemical phenomena. To the best of our knowledge, a kinetic model for photochemical reactions occurring on the two sides of a soap film membrane, i.e., in the two surfactant monolayers, has never been presented before. We therefore took inspiration by a similar effort by Bjelajac and coworkers [27], although the latter study has been recently developed for a different photocatalytic system.

The adsorption and desorption of the gaseous species to and from the gas–surfactant–water interface is described according to the Langmuir adsorption isotherm, and the photochemical half reactions are included as a single-step surface reaction. Atomistic simulations [15, 28–30] and experimental measurements [31–33] show that gas molecules tend to be trapped at the gas–water and gas–surfactant–water interfaces. A measure of the surface concentration can be obtained experimentally from the variation of the surface tension with the external pressure of the gas [34, 35]; however, experimental data for gas adsorption at the gas–surfactant–water interface are scarce. Hence, we resort to the available data for gas adsorption at the gas–water interface. Donaldson and coworkers [36] proposed a Langmuir-type adsorption isotherm for ammonia on a water surface, fitting their experimental data with the following Langmuir equation

5 $$\begin{aligned} \Theta = \frac{c_s}{\Gamma _s} = \frac{K_\mathrm{eq}c}{1+K_\mathrm{eq}c}, \end{aligned}$$

where $c_s$ is the surface concentration, $\mathrm {\Gamma }_s$ is the maximum surface concentration, $K_\mathrm{eq}$ is the equilibrium constant, and *c* is the concentration in the bulk. The fitting procedure provides $\mathrm {\Gamma }_s$ and $K_\mathrm{eq}$. Following a similar approach, we best fit the gas adsorption isotherms measured by Massoudi and coworkers [37] for the gases of our interest to extract the equilibrium parameters (see Supplementary file 1: Fig. S2, and the related fitting parameters reported in Supplementary file 1: Table S5). The obtained values for the maximum surface concentration $\mathrm {\Gamma }_s$ and equilibrium constant $K_\mathrm{eq}$ are two inputs for the continuum model. Particularly, we refer to a reaction rate (*r*) defined as follows [38]

6 $$\begin{aligned} r = v_\mathrm{ads} - v_\mathrm{des} = k_\mathrm{ads} c (\Gamma _s - c_s) - k_\mathrm{des} c_s, \end{aligned}$$

with $v_{ads}$ being the adsorption rate, $v_\mathrm{des}$ the desorption rate, $k_\mathrm{ads}$ the adsorption constant and $k_\mathrm{des}$ the desorption constant. In Eq. 6, the first term on the right-hand side accounts for the adsorption reaction, while the second term for the desorption reaction. At the equilibrium, the reaction rate is zero and, following from Eq. 6, the equilibrium constant reads as

7 $$\begin{aligned} K_\mathrm{eq} = \frac{k_\mathrm{ads}}{k_\mathrm{des}}. \end{aligned}$$

We assume that the surface species are always in equilibrium with their corresponding bulk phases, meaning that the adsorption and desorption reactions are much faster than the reduction of carbon dioxide or the oxidation of water.

In the water core, the transport of charged species is modeled using the Nernst–Planck equation as:

8 $$\begin{aligned}&\frac{\partial c_i}{\partial t} + \nabla \cdot {\mathbf {J}}_i = R_i; \end{aligned}$$

9 $$\begin{aligned}&{\mathbf {J}}_i = - D_i \nabla c_i - \frac{D_i z_i F c_i}{R T} \nabla V. \end{aligned}$$

The first term on the right-hand side of Eq. 9 represents the Fickian diffusion contribution, the second term is responsible for transport due to a spatially dependent electric potential *V*, $c_i$ is the bulk concentration of the *i*-th species, ${\mathbf {J}}_{\mathbf {i}}$ the molar flux, $R_i$ a source term related to chemical reactions, $D_i$ the diffusion coefficient in water, $z_i$ the valence of the ionic species, *T* the absolute temperature, *F* the Faraday constant and *R* the universal gas constant. For the sake of simplicity, for each species here we assume that the diffusion coefficient does not show spatial dependency. However, under strong nanoconfinement conditions (e.g., for sufficiently thin soap film membranes) viscosity, mass and heat transport coefficients might differ from bulk values and exhibit spatial dependency especially in a neighborhood of the boundary [39, 40]. Upon characterization (e.g., by molecular dynamics simulations), inclusion of those effects into the current model is straightforward. The considered system is electroneutral, and the charge is conserved; therefore, according to the Poisson equation, the following conditions hold

10 $$\begin{aligned}&{\mathbf {E}} = - \nabla V, \end{aligned}$$

11 $$\begin{aligned}&\nabla \cdot \left( \varepsilon _0 \varepsilon _r {\mathbf {E}} \right) = \rho , \end{aligned}$$

with $\rho$ being the charge density and $\varepsilon _r$ the dielectric constant of the medium. In the case of a soap film, the dielectric constant is that of water, which is assumed equal to 78.5 [41]. Similarly to transport coefficients, also the dielectric constant might exhibit spatial dependency in the close proximity of the membrane boundary [16] and it might be included in the model if needed. The kinetic model implemented is reported in Table 1. We did not consider the hydrogen reduction reaction since for similar systems the selectivity of the system to CO is about 80% [42].

**Table 1:** Kinetic model for the two half reactions in the two surfactant monolayers

*Carbon dioxide reduction*	
$$\mathrm {CO}_{2(g)} \rightleftharpoons \mathrm {CO}_{2\mathrm{(a d s)}}$$	
$$\mathrm {CO}_{2\mathrm{(a d s)}}+2 Q_{\mathrm{(a q)}}^{-}+2 \mathrm {H}_{2} \mathrm {PO}_{4\mathrm{(a q)}}^{-} {\mathop {\longrightarrow }\limits ^{k_{\mathrm{r e d}}}} \mathrm {CO}_{(g)}+\mathrm {H}_{2} \mathrm {O}_{(l)}+2 Q_{\mathrm{(a q)}}+2 \mathrm {HPO}_{4\mathrm{(a q)}}^{2-}$$	
*Water oxidation*	
$$\mathrm {H}_{2} \mathrm {O}_{(l)}+2 Q_{\mathrm{(a q)}}+2 \mathrm {HPO}_{4\mathrm{(a q)}}^{2-} {\mathop {\rightarrow }\limits ^{k_{\mathrm{o x}}}} \frac{1}{2} \mathrm {O}_{2(g)}+2 Q_{\mathrm{(a q)}}^{-}+2 \mathrm {H}_{2} \mathrm {PO}_{4\mathrm{(a q)}}^{-}$$	

Depending on the different types of adopted photosensitizers and catalysts, the reaction pathway(s) change significantly [43, 44]. Here, we introduce an effective reaction constant ${k_i}=k_\mathrm{red}$ for the CO$_2$ reduction and ${k_i}=k_\mathrm{ox}$ for the water oxidation. We assume first-order reactions for all the reactants except for water, which is treated as a solvent. Clearly, the above hypothesis shall be confirmed or further refined once experimental data become available. Thus, in this work, we define the reaction rates as follows:

12 $$\begin{aligned} r_\mathrm{red} & = k_\mathrm{red} [\mathrm{CO}_{2(\mathrm{ads})}][\mathrm{Q}^-][\mathrm{H}_2 \mathrm{P O}_4^-] \end{aligned}$$

13 $$\begin{aligned} r_\mathrm{ox} & = k_\mathrm{ox} [\mathrm{Q}] [\mathrm{H P O}_4^{2-}] \end{aligned}$$

We consider the dissolution of CO$_2$ [45] in carbonate and bicarbonate in water, with the consequent reduction of the pH in the water core of the soap film. The buffering role of the phosphate was included [46]. This is crucial, since the buffer regulates the pH and acts as the proton donor and acceptor for the reactions reported in Table 1. The equilibrium reactions considered in the water core along with their equilibrium and forward reaction constants are reported in Table 2. Note that the phosphate buffer and water are assumed to be always at equilibrium. The rate of consumption of the reactants and the generation of the products, both for the equilibrium reactions in the water core and the reactions in the surfactant monolayers, is coupled with the transport equations by using the source term $R_i$ in Eq. 8. Supplementary file 1: Figures S3, S4 summarize the equations and the domains where they are solved, along with their boundary conditions.

It is worth to remark that the two reaction equations in Table 1 can be split into a more detailed kinetic description, e.g., by including reaction pathways for the carbon dioxide reduction to carbon monoxide [44]. Nonetheless, this requires that the reactions constants become available upon dedicated experimental studies. In perspective, a more complex representation including the decomposition of the photosensitizers and catalysts could be also included with detailed mechanisms being possibly simplified and/or analyzed by adequate algorithms [47, 48].

### Mesoscale model of a surfactant monolayer

A mesoscale model was developed for understanding the self-assembling process of the photosensitizer and catalyst molecules in a single monolayer, and how this can possibly influence the expected performance of a soap film membrane. To this end, a tailored coarse-grained Metropolis Monte Carlo based algorithm has been developed. The model takes as an input the molecular characteristics of the coarse-grained amphiphilic molecules (photosensitizers and catalysts), namely their size, charge and dipole, and generates a series of consistent microstates of the self-assembled surfactant monolayer in the NVT ensemble, under fixed areal concentrations and temperature. Details are reported in the dedicated section (see “Methods” section). For the sake of validation only, the surface pressure predicted by the above mesoscopic model was compared against available experimental data for the popular sodium dodecyl sulfate (SDS) surfactant [49]. The results are shown in Supplementary file 1: Fig. S5. Subsequently, the mesoscopic model was used to investigate the behavior of different mixtures of catalysts and photosensitizers. A +2 charged photosensitizer [18, 21] and a neutral catalyst both for the CO$_2$ reduction side [22] and for the water oxidation side [21] were adopted. However, it is worth to note that other catalysts and photosensitizer may also be employed for other reactions of interest [42, 50]. The input data for the considered photoactive surfactants are reported in Supplementary file 1: Tables S6 and S7.

**Table 2:** Reaction equations, equilibrium constants and forward reactions constants adopted in the water core of the soap film

Reaction equations	Equilibrium constant	Forward reaction constant	References
$\mathrm {H}_{3} \mathrm {PO}_{4} \rightleftharpoons \mathrm {H}_{2} \mathrm {PO}_{4}^{-}+H^{+}$	$K_{1}=10^{-2.14}$ $\mathrm {M}$	$k_{1}=10^{5} \mathrm {~s}^{-1}$	[46]
$\mathrm {H}_{2} \mathrm {PO}_{4}^{-} \rightleftharpoons \mathrm {HPO}_{4}^{2-}+H^{+}$	$K_{2}=10^{-7.2}$ $\mathrm {M}$	$k_{2}=10^{5} \mathrm {~s}^{-1}$	[46]
$\mathrm {HPO}_{4}^{2-} \rightleftharpoons \mathrm {PO}_{4}^{3-}+H^{+}$	$K_{3}=10^{-12.37}$ $\mathrm {M}$	$k_{3}=10^{5} \mathrm {~s}^{-1}$	[46]
$\mathrm {CO}_{2}+\mathrm {H}_{2} \mathrm {O} \rightleftharpoons \mathrm {HCO}_{3}^{-}+H^{+}$	$K_{4}=10^{-6.3}$	$k_{4}=3.71 \times 10^{-2} \mathrm {~s}^{-1}$	[45]
$\mathrm {HCO}_{3}^{-} \rightleftharpoons \mathrm {CO}_{3}^{2-}+H^{+}$	$K_{5}=10^{-10.3}$ $\mathrm {M}$	$k_{5}=59.44 \mathrm {~s}^{-1}$	[45]
$\mathrm {CO}_{2}+\mathrm {OH}^{-} \rightleftharpoons \mathrm {HCO}_{3}^{-}$	$K_{4}/K_{\mathrm {H}_{2} \mathrm {O}}$	$k_{6}=2.23 \times 10^{3}$ $\mathrm {M}^{-1} \mathrm {~s}^{-1}$	[51]
$\mathrm {HCO}_{3}^{-}+\mathrm {OH}^{-} \rightleftharpoons \mathrm {H}_{2} \mathrm {O}+\mathrm {CO}_{3}^{2-}$	$K_{5}/K_{\mathrm {H}_{2} \mathrm {O}}$	$k_{7}=6 \times 10^{9}$ $\mathrm {M}^{-1} \mathrm {~s}^{-1}$	[51]
$\mathrm {H}_{2} \mathrm {O} \rightleftharpoons \mathrm {OH}^{-}+H^{+}$	$K_{\mathrm {H}_{2} \mathrm {O}}=10^{-14}$ $\mathrm {M}^{2}$	$\mathrm {-}$	[52]

### Incorporation of the mesoscale details in the macroscale model

In this section, we propose an approximate method to link the effective reaction constant, measured experimentally, to the microscopic structure of the reactive surfactant monolayers. For molecular catalysis in confined environments, the concentration of the catalyst has been reported to have a direct dependency on the products of the reaction [22]. Particularly, authors report a saturation after a critical catalyst concentration. In our case, a linear dependency of the reaction constant $k_i$ on the surface concentration of catalysts $\mathrm {\Gamma }_C$ is assumed, as experimental data on reactive soap films are not available yet. A similar approach was used to define the effect of the photon flux on the reaction kinetics. In support to that, authors in [21, 53] report a linear dependence of the reaction products, i.e., oxygen or carbon monoxide, respectively, with the incoming light irradiation. Moreover, Limburg and coworkers [21] report that, after a certain threshold, a further increase in the photon flux does not influence the oxygen production. Thus, we can assume the reaction constant to be proportional to a generic monotonic function of the photon flux $f(\phi )$. In this work, we restrict the analysis to constant irradiance, even though this analysis can be extended to other irradiance conditions. In order to incorporate the topology of the monolayer in the reaction constant, we consider the average number of photosensitizers at a certain distance from a catalyst as a main parameter ($r_e$ in Fig. 1). Indeed, electrons have to tunnel from the photosensitizer to the catalyst or vice versa (depending on the reaction, CO$_2$ reduction or water oxidation) in order for the reaction to occur. The probability of tunneling depends on many factors, such as the medium, the involved molecules and the distance between the two molecules [54, 55]. Here, we analyze only the effect of the latter term for different mixtures, since the subphase and the type of electron relays, photosensitizers and catalysts are kept the same. Thus, we model the dependency of the reaction constant on the number of photosensitizers which surround a catalyst in a radius $r_e$ denoted as $\left( \#PS\ \mathrm{close}\ \mathrm{to}\ 1\ C\right) _{r\le r_e}$. This distance is the result of the electronic interactions between the molecules and the characteristics of the medium. We assume $r_e$ = 1 nm [55], as a representative distance for an outer sphere electron transfer between the considered photosensitizer and the catalyst. It is worth to remark that more detailed information on this parameter could be obtained using spectroscopy methods [56, 57]. Finally, we define the effective reaction constant $k_i$ as follows

14 $$\begin{aligned} k_i=m_i \cdot \Gamma _C \cdot \left( \#PS\ \mathrm{close}\ \mathrm{to}\ 1\ C\right) _{r\le r_e} \cdot f(\phi ), \end{aligned}$$

where $m_i$ is a constant (model parameter) that depends on the chemical nature of the catalyst and of the photosensitizer. We assume the forward reaction constants in a 10:1 mixture (with initial concentrations of the buffer $\mathrm{c}_{0 \mathrm{H}_2 \mathrm{PO}_4^- }=\mathrm{c}_{0 \mathrm{HPO}_4^{2-} }=10$ mM, and of the electron relays $c_{0 Q}=c_{0 Q^- }=2.5$ mM). This corresponds to a photocatalytic turnover frequency (PTOF$_{\mathrm{H}_2\mathrm{O}}$), defined as the moles of oxygen over the moles of PS in time on the water oxidation side, equal to 0.2 s$^{-1}$. This value is coherent with the PTOF for water oxidation performed in a similar environment [21]. However, it is worth to remark that the CO$_2$ reduction reaction might be slower than the water oxidation reaction [22, 53].

Thick soap films are desirable in order to keep the produced CO and the O$_2$ separated for a sufficiently long time, as previously reported [15]. Therefore, we analyze the dependence of the CO production on the thickness of the soap film. We varied the thickness from 100 to 1000 nm, according to the representative average values for soap films [17, 57]. The CO produced for thinner films was slightly smaller than for films thicker than 400 nm, as shown in Supplementary file 1: Fig. S6. This is caused by the low amount of species in the bulk; that is, the amount of reactants in thinner films is not sufficient to develop the full electric double layer (EDL), and this implies a reduced local concentration of reactants close to the monolayers. Thus, in the following we assume an average film thickness equal to 400 nm.

### Scenario 1: dependence of the solar fuel production on the surface concentration of the photoactive molecules

**Fig. 2:**
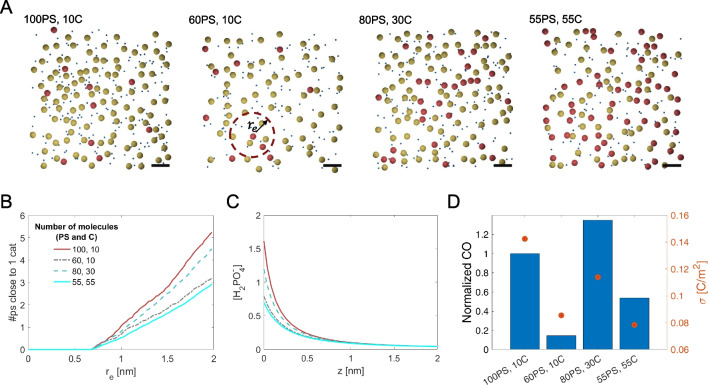
Surface concentration of the photoactive molecules and its influence on the CO production. **A** One of the resulting microstates from the Metropolis Monte Carlo simulations for each of the mixtures analyzed; the PS, their counterions and the C are shown as yellow, blue and red spheres, respectively. The scale (black) bar is 2 nm, and $r_e$ is the radial distance from a generic catalyst C. **B** Dependence of the average number of photosensitizers close to a single catalyst for the different mixtures reported in Table 3. The distance is calculated between the two geometric centers of a spherical representation for the photosensitizer and the catalyst. **C** Concentration profile (Gouy–Chapman diffuse layer) for the H$_2$PO$^-_4$ at the CO$_2$ reduction side. **D** CO production, normalized to the 10:1 case ($\mathrm{d}n/\mathrm{d}t=3.599\times10^{-7}$ mol m$^{-2}$ s$^{-1}$ with initial concentration of the phosphate buffer $\mathrm{c}_{0 \mathrm{H}_2 \mathrm{PO}_4^- }=\mathrm{c}_{0 \mathrm{HPO}_4^{2-} }=10$ mM, of the electron relays $\mathrm{c}_{0 Q}=\mathrm{c}_{0 Q^-}=2.5$ mM and external CO$_2$ pressure $p_{\mathrm{CO}_2}=1$ atm). The orange circles correspond to the surface charge density of the monolayer

In this section, we investigate how four different mixtures of photosensitizers and catalysts self-assemble, and how this can possibly impact on the reaction kinetics. We assume the same concentration and the same photosensitizer/catalyst ratio both for the surfactant monolayer where water oxidation occurs and for the monolayer where CO$_2$ is reduced. Figure 2A shows one of the microstates, resulting from the Monte Carlo Metropolis simulations, for each of the different mixtures (see Table 3). The estimated (average) cumulative number of photosensitizers close to a single catalyst, as function of the distance from the catalyst, is reported in Fig. 2B. The CO produced results from multiple factors, namely: the composition and topology of the monolayer, the surface charge of the monolayer and the charge of the reactants. As an example, even though the 8:3 mixture presents thrice the number of catalysts with respect to the 10:1 mixture, and thus, a thrice CO production may be expected, the CO production yields only in a 20% increase. In these two cases indeed, the $\left( \#PS\ \mathrm{close}\ \mathrm{to}\ 1\ C\right) _{r\le r_e}$ value is similar (Fig. 2B); however, the surface charge of the monolayer for the 8:3 mixture is significantly lower. This is due to the presence of a reduced number of charged surfactant molecules (i.e., the PS in this study) at the surface, which implies a lowering of the charged reactants close to the surface of the monolayer (Fig. 2C). This finally results in a lower CO production than possibly expected.

**Table 3:** Summary of the four analyzed mixtures: ratio between photosensitizer (PS) and catalyst (*C*), surface concentration of the photosensitizers $\left( \Gamma _{\mathrm{P S}}\right)$ and of the catalysts $\left( \Gamma _{C}\right)$, number of molecules of photosensitizers $\left( N_\mathrm{P S}\right)$ and of the catalysts $\left( N_{C}\right)$ in the simulation box $(15 \times 15 \mathrm {~nm})$ of the Metropolis Monte Carlo model

Ratio	$\Gamma _\mathrm{PS}\left( {\mathrm{mol m}}^{-2}\right)$	$\Gamma _{C}\left( {\mathrm{mol m}}^{-2}\right)$	${{N}}_\mathrm{P S}$	${{N}}_{C}$
10:1	$7.3803 \times 10^{-7}$	$7.3803 \times 10^{-8}$	100	10
6:1	$4.4282 \times 10^{-7}$	$7.3803 \times 10^{-8}$	60	10
8:3	$5.9043 \times 10^{-7}$	$2.2141 \times 10^{-7}$	80	30
1:1	$4.0592 \times 10^{-7}$	$4.0592 \times 10^{-7}$	55	55

### Scenario 2: dependence of the solar fuel production on the concentration of the reactants

An extensive sensitivity analysis was performed by varying the initial concentration of the reactants, namely: the concentration of the electron relays ($\mathrm{c}_Q$, $\mathrm{c}_{Q^-}$), of the phosphate buffer ($c_{\mathrm{H}_2 \mathrm{PO}_4^-}$, $\mathrm{c}_{\mathrm{HPO}_4^{2-}}$) and of the CO$_2$ pressure ($p_{\mathrm{CO}_2}$). A 10:1 mixture composition for both the surfactant monolayers is analyzed. We discuss in particular those cases of interest to elucidate on the competition between the reactants and their influence on the produced fuel.

The environment in the membrane is neutral to acidic, depending on the concentration of the phosphate buffer and on the external CO$_2$ pressure. The phosphate anions are attracted by the positively charged monolayer, buffering the zone where the reactions occur. If the external CO$_2$ pressure is high, or in the case of low buffer concentrations, the pH drops close to the surfactant monolayers as well as, more drastically in the water core (see Supplementary file 1: Fig. S7). This acidification of the soap film directly influences its stability and thickness [58, 59]. It is important to note that for very thin aqueous membranes, such as a soap film, the buffering capacity is strongly reduced as compared to a bulk solution. A manifestation of that phenomenon can be noticed in the CO yield variation as a function of the film thickness reported in Supplementary file 1: Fig. S6. Indeed, the overall moles of the buffer, as well as those of the other compounds, are lower than a generic bulk solution contained in a mm size vial since the membrane has a finite small volume.

**Fig. 3:**
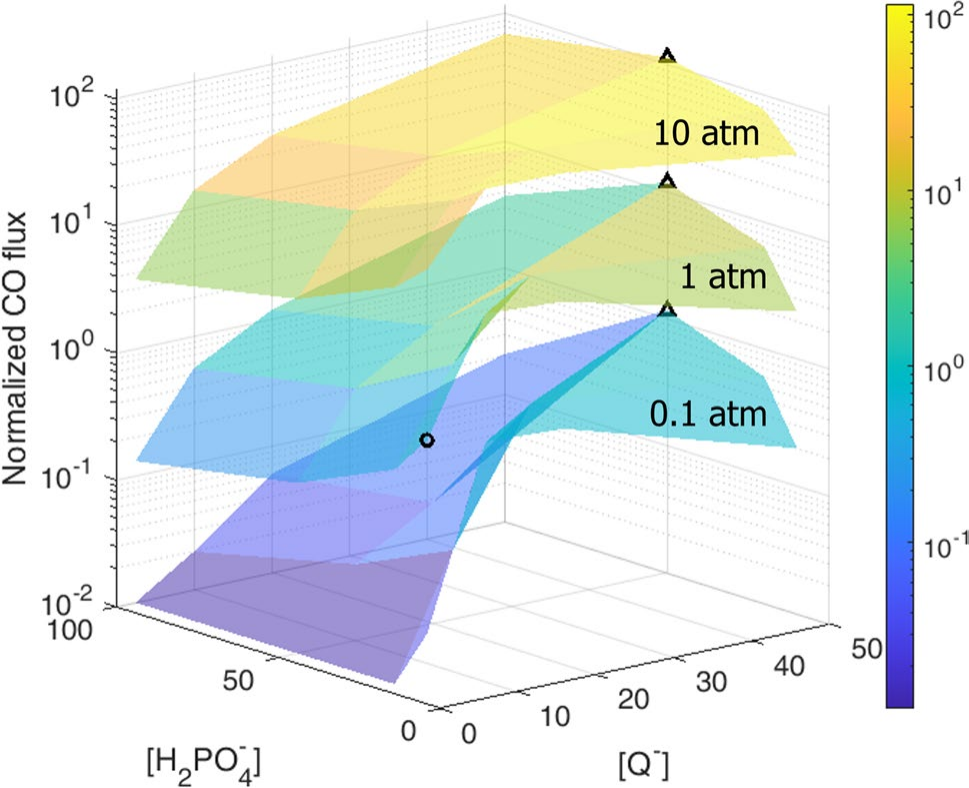
Dependence of the CO production on the initial concentration of the reactants. Normalized CO production depending on the initial molar concentration of the buffer ($\mathrm{c}_{0 \mathrm{H}_2 \mathrm{PO}_4^- }=\mathrm{c}_{0 \mathrm{HPO}_4^{2-} }=10,20,50,100$ mM), the electron relays ($c_{0 Q}=\mathrm{c}_{0 Q^-}=2.5,10,20,50$ mM) and the external CO$_2$ pressure ($p_{\mathrm{CO}_2}=0.1,1,10$ atm). The flux is normalized to the 10:1 case for $\mathrm{c}_{0 Q}=\mathrm{c}_{0 Q^- }=2.5$ mM, $\mathrm{c}_{0 \mathrm{H}_2 \mathrm{PO}_4^- }=\mathrm{c}_{0 \mathrm{HPO}_4^{2-} }=10$ mM and $\mathrm{p}_{\mathrm{CO}_2 }=1$ atm ($\mathrm{d}n/\mathrm{d}t=3.599\times10^{-7}$ mol m$^{-2}$ s$^{-1}$), black circle in the picture. The maximum CO produced at each pressure level is indicated with a triangle. The corresponding numerical values are reported in Supplementary file 1: Fig. S14

The non-monotonic behavior of the normalized CO flux with respect to the concentrations of the electron donor and the buffer, shown in Fig. 3, can be related to the electrostatic interactions and how the different local concentrations of the reactants equilibrate [60]. Particularly, a competition arises in the vicinity of the surfactant monolayers between the different ionic species dissolved in the water core that interact with each other. For instance, the variation of the phosphate concentration from 10 to 100 mM at constant concentration of electron relays results in a reduction of the local concentration of electron relays at the interface by a factor larger than 10 (see Supplementary file 1: Figs. S8–S11). The reason being that the attraction on the $HPO_4^{2-}$ by the positively charged monolayer is stronger than that of the single valence ions [41]. As an example, considering the case of external CO$_2$ pressure equal to 1 atm, and the initial concentration of the phosphate buffer equal to 20 mM, an increase in equal amount of electron relays Q and Q$^-$ does not correspond to a similar increase in the local concentrations of Q and Q$^-$ at the interfaces. The local concentration of the Q$^-$ close to the surfactant monolayers is much higher than the Q, due to the electrostatic attraction from the positively charged monolayers. This leads to a faster consumption of H$_2$PO$^-_4$ at the CO$_2$ reduction side, which shifts the local equilibria at the interface as well as the global equilibrium in the soap film. The latter phenomenon is reflected into a lowering of the pH with the addition of the electron relay, as shown in Supplementary file 1: Figs. S12, S13. A shift in the equilibrium leads to an increase of the H$_3$PO$_4$, which in our current model does not react as proton acceptor or donor. Considering this additional reaction path might result in an increase in the produced CO. However, this contribution can be assumed to be negligible, as H$_3$PO$_4$ is neutral and it is thus not attracted by the electrostatic interactions from the monolayer (thus leading to a relatively low local concentration). It has been previously reported that changes in the pH value have an effect on the photocatalytic production of fuels, as it may change the selectivity of the catalyst [61]. It can also directly influence the stability of the photoactive molecules, e.g., due to protonation–deprotonation of the species [62].

## Implications of the hypothesis

A radically new concept of soft and self-assembled soap film membranes for photocatalytic conversion of the CO$_2$ into solar fuel has been recently proposed [14]. With the aim of setting the scene for this new technology in the near future, we have elaborated a multi-scale and multi-physics modeling framework which, to our knowledge, enable for the first time to theoretically analyze this new concept. Particularly, the proposed model allows to investigate the role played by the several possible operating conditions and design factors on the photochemical conversion of the CO$_2$ into gaseous fuel (CO) within the envisioned membranes. The model includes both a continuum (i.e., macroscopic) and a discrete (i.e., mesoscopic) module. The macroscale module describes the transport of gases and ionic species in the soap film, the chemical equilibria in the water core, the adsorption/desorption of gases and the two chemical half reactions occurring at the surfactant monolayers. The mesoscale module predicts how photosensitizers and catalysts self-assemble at the gas–water interface. The effect of the molecular characteristics of the photosensitizers and of the catalysts, as well as their surface concentration, has been then incorporated in the macroscale model through the forward reaction constant.

The results obtained have allowed to quantify the critical importance of the electron relays, buffer concentration and external pressure of the carbon dioxide on the CO and O$_2$ production. The competition between the charged species at the interface was highlighted. It was found that an increase in the concentration of the phosphate buffer for a constant concentration of the electron relays results in a reduction of the local concentration of the electron relays at the interface. For a 10:1 photosensitizer–catalyst ratio, the parametric analysis has allowed to identify the optimal conditions for the production of CO. Particularly, these are: initial concentrations of the phosphate buffer and of the electron relays at 50 mM for all the tested range of the CO$_2$ pressure. The effect of the surface concentrations of the photosensitizer and catalyst has been also characterized. The optimal production of CO was obtained for a 8:3 photosensitizer–catalyst ratio.

We believe that the described model offers the remarkable possibility of disentangling the several different processes taking place in the soap film during the CO$_2$ conversion, and this is critical for optimal design of future soft photosynthetic membranes. However, we would like to remark that, due to the current lack of both fundamental and experimental knowledge on the considered systems, a number of simplifying assumptions were necessary throughout this preliminary study. In particular, the adopted version of the kinetic model is only an effective one and only partially includes the important properties of the self-assembled functional molecules. As such, far from being conclusive, this study rather aims at representing: (i) a starting point for future studies toward soft self-assembled membranes for photocatalytic purposes; (ii) a motivation to improve the current understanding of the morphological features of the self-assembled monolayers. In this respect, we believe that neutron scattering [63], sum frequency generation (SFG) spectroscopy [16] and statistical reconstruction of complex geometries [64] may help in shedding further light on such systems. Moreover, we believe that time-resolved spectroscopy investigating electron transfer [56, 57] as well as dedicated experiments to evaluate the fuel and/or oxygen production (even in model experimental setup such as those based on Langmuir troughs, micellar systems or other supramolecular self-assembled systems) can be used to probe the evolution of the two half reactions. Finally, we envision further applications of this model to describe different photochemical reactions inside a soap film and on its surfaces.

## Methods

### Macroscale model

A one-dimensional (1D) model is developed to analyze the performance of the photocatalytic soap film membrane. The model is implemented and solved via finite element method (FEM) in COMSOL Multiphysics 6.0. A symmetric mesh for the gas phase and the water core is adopted (see Supplementary file 1: Fig. S15). In particular, a coarser mesh is adopted to solve the diffusion of gases in the gas phase. A refined mesh, instead, is used to accurately solve the electric double layer (EDL) in the liquid. The number of discrete elements and the element ratio (grow rate) chosen are reported in Supplementary file 1: Table S8. Three studies were performed to calculate the production of CO and O$_2$ at steady-state conditions. First, the species are let equilibrate with the CO$_2$ dissolving in the water core and reacting with the other already present species (such as the phosphate buffer) for 10 s. Second, the electrostatic interactions are considered, where the positively and negatively charged ions are, respectively, repelled and attracted by the positively charged surfaces of the monolayers for 1.5 ms. In this step, the typical exponential decay of the Gouy–Chapman diffuse layer appears [37, 65]. The final step with the two half reactions takes around 10 s for the system to reach equilibrium conditions. The corresponding concentration of reactants after each of the previous studies is reported in Supplementary file 1: Figs. S16, S17. The input parameters of the model are summarized in Supplementary file 1: Tables S1–S5. A detailed and exhaustive explanation of the COMSOL model is reported in Supplementary file 2: Data S1.

### Mesoscale model

According to a multiple expansion truncated at the first order, the single surfactant molecule is approximated with a point charge and its dipole, as shown in Supplementary file 1: Fig. S18. In particular, for a surfactant with partial charges of its atoms $q_i$ with Cartesian coordinates $\mathbf {r_i}$, the total dipole is defined as $\varvec{\mu }=\sum q_i \mathbf {r_i}$. If the total charge of the system is zero, the system is neutral (as for nonionic surfactants) and the dipole moment does not depend on the reference system. However, this is not valid for those systems whose charge is overall positive (or negative); this is the case of ionic surfactants. In this case, the reference point chosen for the dipole representation is usually the center of mass (${\mathbf {R}}$) of the system, which yields: $\varvec{\mu }=\sum q_i (\mathbf {r_i-R}$), where ${\mathbf {R}}=\frac{1}{M}\sum m_i \mathbf {r_i}$ and $M=\sum m_i$ with $m_i$ being the atomic mass of the atom *i* in the molecule, and *M* the total mass of the molecule [66]. The head group of the surfactant is modeled as a hard sphere (bead) with diameter $d_\mathrm{surf}$. The volume occupied by the tail is considered by excluding all the configurations where two particles do not satisfy the condition $\arccos (r/(\mathrm{d}_{\mathrm{surf},i}+\mathrm{d}_{\mathrm{surf},j}))> | \alpha |$, with $\alpha = \arccos (\mathrm{d}_{\mathrm{surf},i}/(\mathrm{d}_{\mathrm{surf},i}+\mathrm{d}_{\mathrm{surf},j}))$ and *r* the distance between the two molecules (see Supplementary file 1: Fig. S18). Thus, an exclusion zone above each surfactant, which depends on their dimensions, is considered to avoid nonphysical overlapping along the z direction. Finally, the counterion is approximated as a point charge and a bead with diameter $\mathrm{d}_\mathrm{ion}$.

The Monte Carlo algorithm proceeds by inserting the coarse-grained surfactants molecules in a monolayer and, if present, their counterions in the bulk according to the following procedure (see Supplementary file 1: Fig. S19):

1. Randomly insert one surfactant (with its counterions if the surfactant is ionic);
2. Compute the energy variation of the system due to the particle insertion;
3. Compute the probability of retaining the inserted particles, namely: Probability $=\exp \left( -\Delta U / k_{B} T\right)$, where $\Delta U$ is the free energy variation of the system after the new molecule has been added;
4. Draw a random number (*R*) from 0 to 1;
5. Accept if: Probability$>R$, else reject;
6. If the target surface concentration is not yet achieved, restart from step 1, else terminate the procedure.

The surfactants are inserted with a uniform distribution in the $x-y$ plane, and a Gaussian distribution in the z direction perpendicular to the air–water surface obtained by fitting the density profiles of the heads along the *z* direction resulting from molecular dynamics simulations [15, 25]. The counterions are inserted with a uniform distribution in the whole simulation box. We assumed the relative dielectric permittivity of water as $\varepsilon =78.5$. The complete MATLAB^®^ code is reported in Supplementary file 3: Data S2. The free energy variation $\Delta U$ consist of three terms: the charge–charge interaction, the charge–dipole interaction and the dipole–dipole interaction. The following equation is implemented for the charge–charge interaction

15 $$\begin{aligned} U_{0 c c}(r)=-\frac{1}{4 \pi \varepsilon \varepsilon _{0}} \frac{q_{1} q_{2}}{r} \end{aligned}$$

A thermal average of the charge–dipole interaction is implemented in the code, in order to consider the dipole moment fluctuation due to thermal agitation. More in detail, molecular dynamics simulations show that surfactants at the gas–water interface oscillate with a preferential orientation of their aliphatic tail toward the gas phase [15, 67]. Thus, we calculated the charge–dipole interaction as [68]

16 $$\begin{aligned} \left\langle U_\mathrm{c d}(r)\right\rangle \equiv \frac{\int _{0}^{2 \pi } \mathrm{d} \zeta \int _{\vartheta =\theta _{1}}^{\theta _{2}} U_{0}(r) f(\vartheta ) \exp \left( -\frac{U_{0 c d}(r) f_\mathrm{c d}(\vartheta )}{k_{B} T}\right) \sin (\vartheta ) \mathrm{d} \vartheta }{\int _{0}^{2 \pi } d \zeta \int _{\vartheta =\theta _{1}}^{\theta _{2}} \exp \left( -\frac{U_{0 c d}(r) f_\mathrm{c d}(\vartheta )}{k_{B} T}\right) \sin (\vartheta ) \mathrm{d} \vartheta } \end{aligned}$$

where

17 $$\begin{aligned} f_\mathrm{c d}(\vartheta )=\cos (\vartheta ) \end{aligned}$$

and

18 $$\begin{aligned} U_{0 c d}(r)=-\frac{1}{4 \pi \varepsilon \varepsilon _{0}} \frac{q \mu }{r^{2}} \end{aligned}$$

Similarly, thermal average for a single dipole–dipole interaction was implemented as [68]:

19 $$\begin{aligned} \left\langle U_{d d}(r)\right\rangle \equiv \frac{\int _{0}^{\zeta } \mathrm{d} Z \int _{\Theta _{1}=\theta _{1}}^{\theta _{2}} \int _{\Theta _{2}=\theta _{1}}^{\theta _{2}} U_{0 d d}(r) f(\Omega ) \exp \left( -\frac{U_{0 d d}(r) f_{d d}(\Omega )}{k_{B} T}\right) \sin \left( \Theta _{1}\right) \mathrm{d} \Theta _{1} \sin \left( \Theta _{2}\right) \mathrm{d} \Theta _{2}}{\int _{0}^{\zeta } \mathrm{d} Z \int _{\Theta _{1}=\theta _{1}}^{\theta _{2}} \int _{\Theta _{2}=\theta _{1}}^{\theta _{2}} \exp \left( -\frac{U_{0 d d}(r) f_{c d}(\Omega )}{k_{B} T}\right) \sin \left( \Theta _{1}\right) \mathrm{d} \Theta _{1} \sin \left( \Theta _{2}\right) d \Theta _{2}} \quad \end{aligned}$$

where

20 $$\begin{aligned} f_{d d}(\Omega )=f_{d d}\left( \vartheta _{1}, \vartheta _{2}, \zeta \right) =-\left( 2 \cos \left( \vartheta _{1}\right) \cos \left( \vartheta _{2}\right) -\sin \left( \vartheta _{1}\right) \sin \left( \vartheta _{2}\right) \cos (\zeta )\right) \end{aligned}$$

and

21 $$\begin{aligned} U_{0 d d}(r)=-\frac{1}{4 \pi \varepsilon \varepsilon _{0}} \frac{\mu _{1} \mu _{2}}{r^{3}} \end{aligned}$$

For a more detailed definition of the angles, refer to Supplementary file 1: Fig. S20. Periodic boundary conditions were applied along the *x* and *y* directions. In particular, we adopted a near image approximation where a particle *i* interacts with the nearest image of the particle *j* (see Supplementary file 1: Fig. S21) to avoid boundary effects. Convergence analysis was performed for the sodium dodecyl sulfate (SDS) surfactant for three different cubic simulation boxes of lateral size 10, 15 and 20 nm, respectively, and compared with the experimental data [49] (see Supplementary file 1: Fig. S15). A microstate resulting from the Metropolis Monte Carlo algorithm for a $15 \times 15 \times 15$ $\mathrm {nm}$ box is reported in Supplementary file 1: Fig. S22. The input parameters of the simulations are reported in Supplementary file 1: Table S9. After convergence analysis, a simulation box of $15 \times 15 \times 15 \mathrm {~nm}$ was chosen for the simulations of the photosensitizer–catalyst mixtures. In Supplementary file 1: Fig. S22, the electric double layer resulting from the MMC simulations is shown. In particular, the total number of counterions in the bulk is equal to the total number of surfactants on the surface. Indeed, the simulations are run in NVT ensemble, and thus, the number of particles is constant and in every insertion cycle of the algorithm one surfactant and its counterion are inserted. The surface tension was calculated with the test-area method, which was first introduced by Gloor and coworkers [69–71]. We consider the definition of surface tension as the change in the Helmholtz free energy due to a change in the surface area of the system at constant temperature, total volume and total number of particles as

22 $$\begin{aligned} \left. \frac{\mathrm{d} U}{d A}\right| _{T, V, N} \equiv \gamma . \end{aligned}$$

The surface tension is split into three contributions as

23 $$\begin{aligned} \gamma =\gamma _{0}+\gamma _{s}+\gamma ^{\prime }, \end{aligned}$$

with $\gamma _{0}$ being the contribution from the solvent, $\gamma _{s}$ the effect of the surfactants and $\gamma ^{\prime }$ the mutual effects between the surfactants and the solvent. In our case, we consider an implicit solvent representation, and hence, $\gamma ^{\prime }=0$. This leads to the following definition of the surface pressure:

24 $$\begin{aligned} \pi =\gamma _{0}-\left( \gamma _{0}+\gamma _{s}+\gamma ^{\prime }\right) =-\gamma _{s} \quad \end{aligned}$$

where $\gamma _{s}$ is calculated according to Eq. 22.

When applying the Metropolis Monte Carlo method, the sum of the dipole–dipole, charge–dipole and charge–charge interactions among all the particles is computed as

25 $$\begin{aligned} U= & {} \sum _{i=1}^{N_{c}} \sum _{j=i}^{N_{c}}\left( U_{0 c c(i, j)}\right) +\sum _{i=1}^{N_{c}} \sum _{j=1}^{N_{d}}\left( U_{c \mathrm{d}(i, j)}\right) \nonumber \\&+\sum _{i=1}^{N_\mathrm{d}} \sum _{j=i}^{N_\mathrm{d}}\left( U_{\mathrm{d} \mathrm{d}(i, j)}\right) , \quad i \ne j \quad \end{aligned}$$

being $N_{c}$ the total number of charges in the system, and $N_{d}$ the total number of dipoles. According to Eqs. 22 and 25, the surface tension is calculated from the simulations as

26 $$\begin{aligned} \gamma =\left. \left. \frac{\Delta U}{\Delta A}\right| _{T, V, N} \approx \frac{\left( U_{1}-U_{0}\right) }{\left( A_{1}-A_{0}\right) }\right| _{T, V, N}, \end{aligned}$$

where the subscripts 1 and 0 on the energy, *U*, and surface area, *A*, indicate the system before and after the geometrical transformation has been applied. Particularly, the following transformations are considered for each microstate resulting from the Monte Carlo Metropolis simulations:

27 $$\begin{aligned} L_{x, 1} & = L_{x, 0} \sqrt{1+\Delta \mathrm {A}}, \end{aligned}$$

28 $$\begin{aligned} L_{y, 1} & = L_{y, 0} \sqrt{1+\Delta \mathrm {A}}, \end{aligned}$$

29 $$\begin{aligned} L_{z, 1} & = L_{z, 0}(1+\Delta \mathrm {A})^{-1}, \end{aligned}$$

where $L_{x, 0}=L_{y, 0}=L_{z, 0}$ are the dimensions of the simulation box before the geometric transformation, and $L_{x, 1}, L_{y, 1}$ and $L_{z, 1}$ are those after the transformation is applied, as shown in Supplementary file 1: Fig. S23. Here, we adopt the following dimensionless parameter $\Delta \mathrm {A}=5 \times 10^{-4}$ [69]. The surface tension was calculated as an average of eight simulations of the Metropolis Monte Carlo algorithm for each of the analyzed cases.

## Supplementary Information

Below is the link to the electronic supplementary material.

Supplementary file1 (PDF 1972 KB)

Supplementary file2 (ZIP 2426 KB)

Supplementary file3 (ZIP 48 KB)

## Data Availability

The supplementary Figures and Tables are available in the Supplementary file supp-info.pdf.

## Code availability

The codes used in this study are available in the Supplementary files Data S1 and Data S2.
