# Supplementary material for: A novel concept of photosynthetic soft membranes: a numerical study
Source: Discov Nano. 2023 Feb 9;18(1):9. doi: 10.1186/s11671-023-03772-1 (PMC9911585; doi:10.1186/s11671-023-03772-1)
Supplement: Supplementary file 1 — Supplementary file1 (PDF 1972 KB) [file 11671_2023_3772_MOESM1_ESM.pdf]

Supplementary information file for:

A novel concept of photosynthetic soft membranes: A numerical study

Gabriele Falciani,[1] Luca Bergamasco,[1] Shannon A. Bonke,[2] Indraneel Sen,[3] and Eliodoro Chiavazzo[1,*]

[1] Department of Energy, Politecnico di Torino, C.so Duca degli Abruzzi 24 - 10129, Torino, Italy
[2] Yusuf Hamied Department of Chemistry, University of Cambridge, Lensfield Rd, Cambridge CB2 1EW, UK
[3] Department of Chemistry, Uppsala University, Laegerhyddsvaegen 1 - 751 20, Uppsala, Sweden

*Correspondence: eliodoro.chiavazzo@polito.it

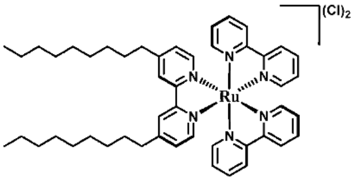

Figure S1. Chemical structure of the alkylated Ruthenium based photosensitizer (Tris(bipyridine)ruthenium(II) chloride) studied in the work of (1)

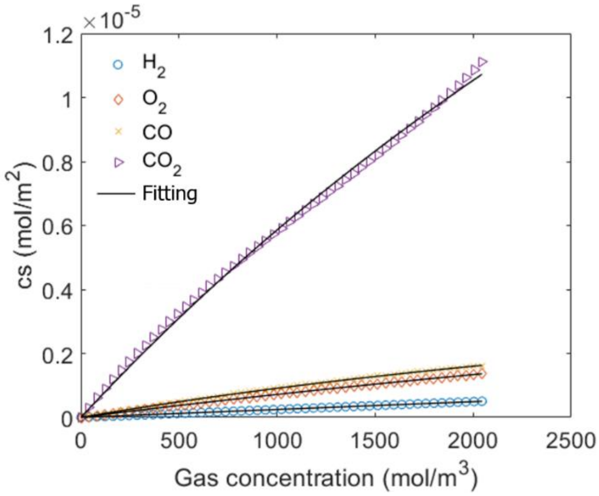

Figure S2. Experimental results for the gas adsorption at the gas-water interface, and best fitting from (2)**.** The surface concentration at the water-air interface as function of the bulk concentration in the gas phase for four gases is reported. Data from (2) (colored markers) are fitted with a Langmuir type model (continuous black line).

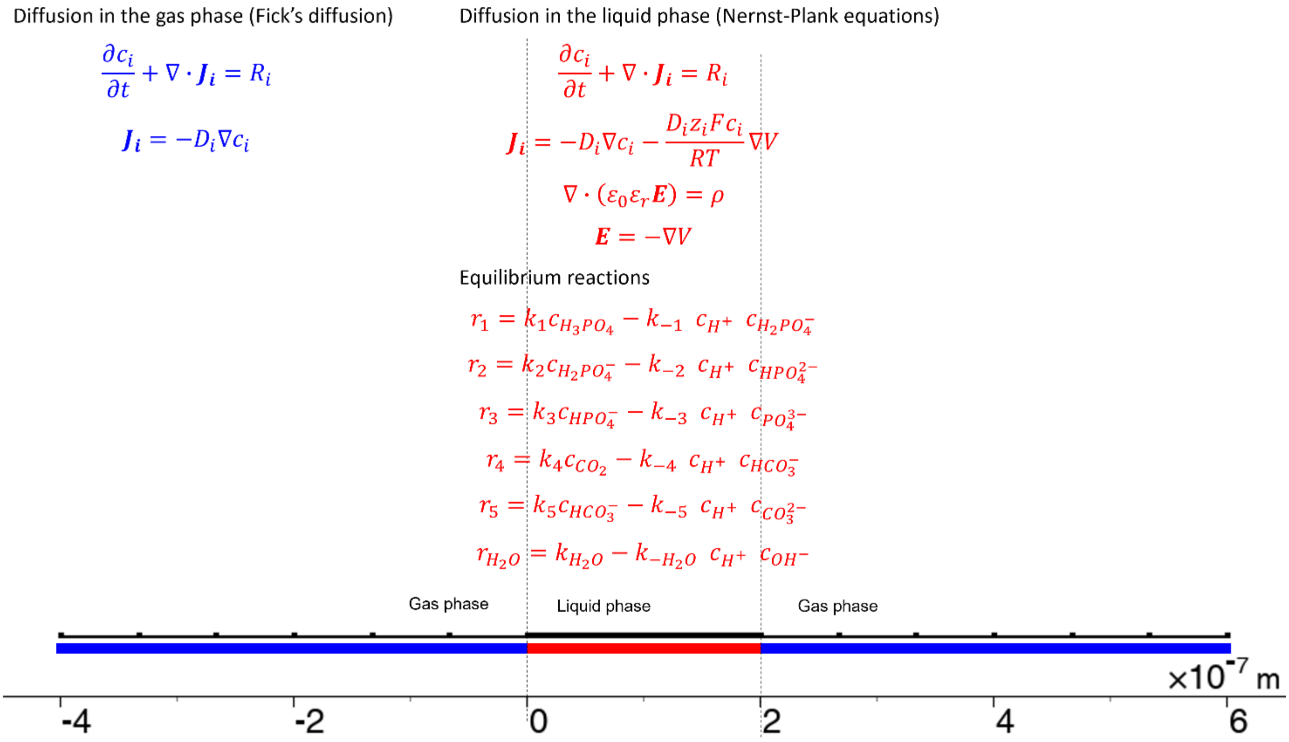

Figure S3. Equations solved in each domain. Fickian diffusion is assumed for each of the species i in the gas phase (depicted in blue). Nerst-Plank equations are solved in the liquid core of the soap film for describing the transport of ionic species. If the species are neutral, Fickian diffusion is assumed. The term R_i takes into account the production and consumption of the i-th species and is related to the chemical equilibrium reactions reported in Table 2 and to the surface reactions in **Figure S3**. In the equilibrium reactions, r is the rate of the

reaction, k the forward reaction constant and k_- the reverse rate constant calculated from the equilibrium constant in Table 2.

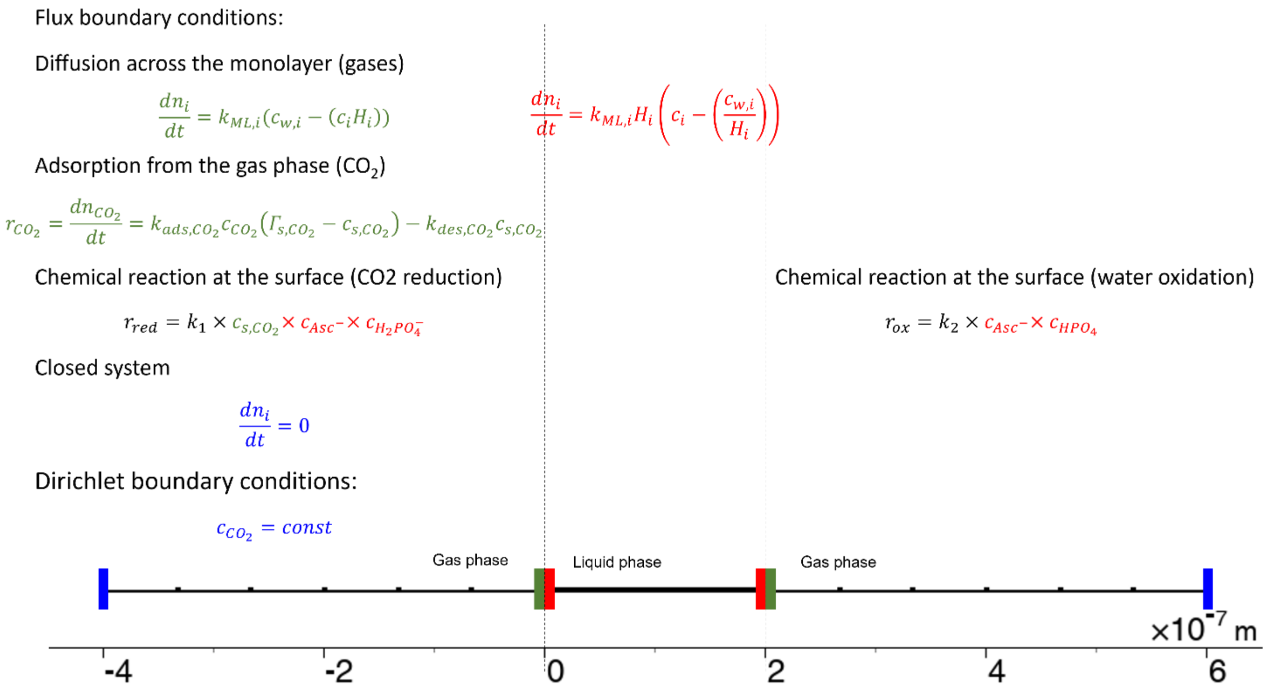

Figure S4. Boundary conditions applied in the continuum model. Neumann boundary conditions were applied to maintain the flux continuity of the permeating gas i at the air-water interface. The solubility of the gas in the water core of the soap film is considered using the Henry's Law. The boundary conditions at the gas-water interface applied in the gas and in the liquid domains are depicted in blue and red, respectively. The adsorption of gases at the interfaces occurs from the gas domain. Here, we focus only on CO_2 since it is the only gaseous reactant as shown in Table 1. At the interface, the gases in the gas phase are assumed to react with the non-gaseous species dissolved in the solution. The same occurs for the products: gaseous products are released in the gas phase and the non-gaseous in solution. This is a reasonable assumption since the diffusion coefficient in the gas phase is higher than in the liquid; thus, the gases will tend to diffuse in the gas phase. Moreover, the gases will solubilize to reach the equilibrium imposed by the Henry's Law. Dirichlet boundary conditions for the CO_2 at the two ends of the simulation domains are applied (depicted in blue), since we are studying the equilibrium conditions and the production of CO and O_2 is relatively small. The gaseous products (CO and O_2) accumulate in the domain without interfering with the simulation. The integral over time and space of the products give the total production as function of time.

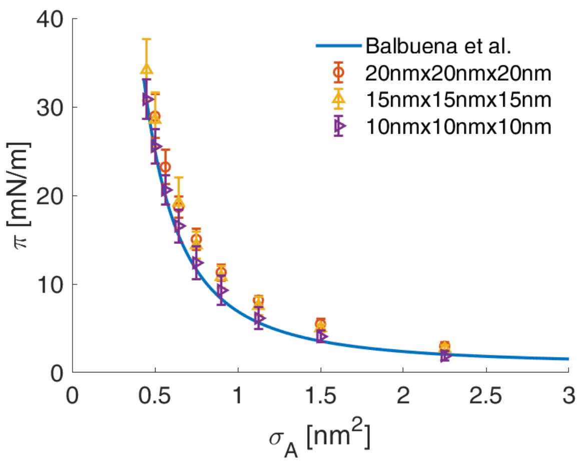

Figure S5. Convergence analysis of the Metropolis Monte Carlo algorithm for different sizes (length, thickness and depth) of the cubic computational box (nm). The results are compared with available data of the surface pressure as a function of the mean area per molecule. This latter is obtained by fitting the

adsorption isotherm for SDS (3) (solid line) using the Gibbs adsorption equation for ionic surfactants, namely $\Gamma = -1/nRT\, d\gamma/d\ln(C)$ being $n = 2$ as SDS is a ionic surfactant, T the temperature, R the universal gas constant, Γ the surface excess, γ the surface tension, and C the SDS bulk concentration. The surface excess is related to the area per molecule σ_A with the Avogadro constant N_A: $\sigma_A = 1/N_A\Gamma$.

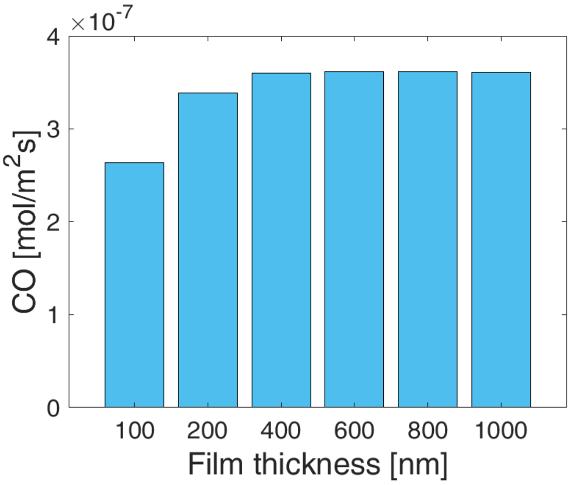

Figure S6. Sensitivity analysis: CO production as function of the film thickness. Maximum molar flux $dn/dt = 3.617 \cdot 10^{-7}\ mol/m^2s$) for a film thickness of 600 nm.

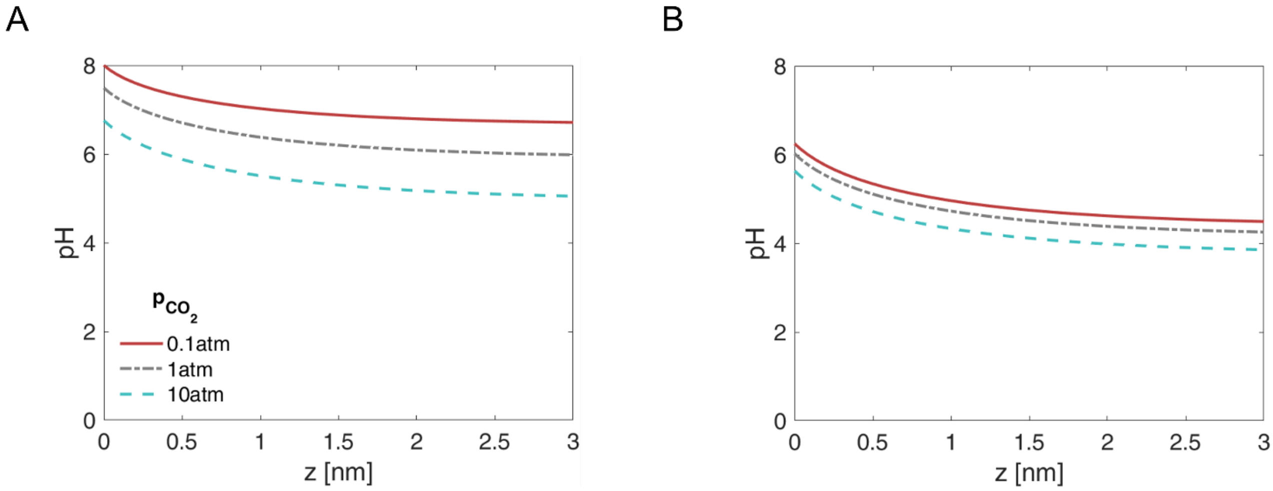

Figure S7. pH close to the surfactant monolayer depending on the partial pressure of the CO_2 at the CO_2 reduction side. Initial concentration of the electron relay $c_{0\ Q} = c_{0\ Q^-} = 20\ mM$. Initial concentration of the buffer $c_{0\ H_2PO_4^-} = c_{0\ HPO_4^{2-}} = 20\ mM$. (A) Reactions are not taking place. (B) Reactions are occurring.

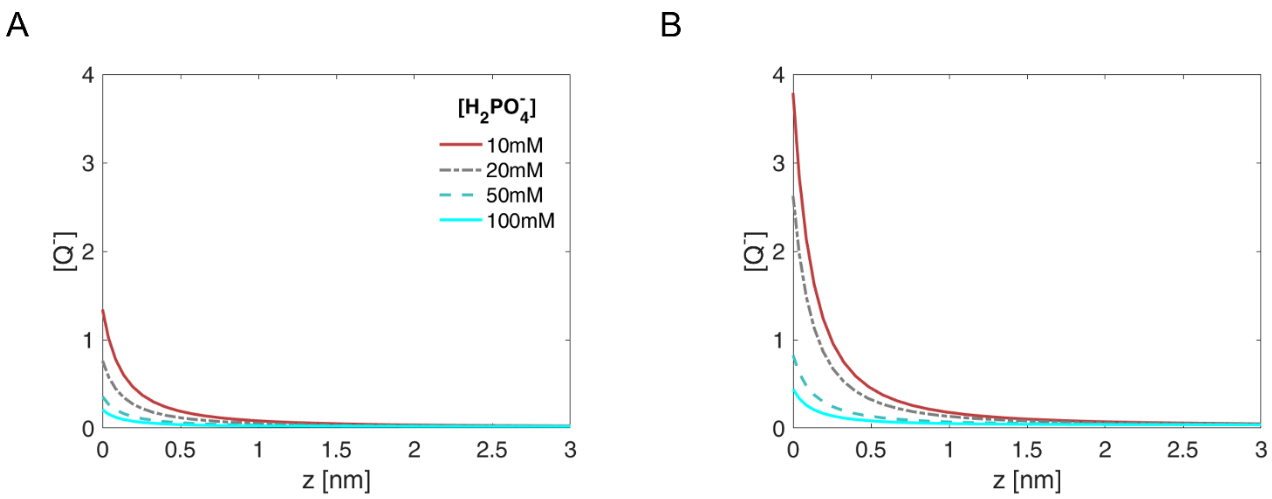

Figure S8. Molar concentration of electron relays Q⁻ close to the surfactant monolayer depending on the initial concentration of the phosphate buffer ($c_{0\,H_2PO_4^-} = c_{0\,HPO_4^{2-}} = 10, 20, 50, 100\ mM$). Initial concentration of the electron relay $c_{0\,Q} = c_{0\,Q^-} = 20\ mM$ at the CO_2 reduction side. (A) Reactions are not taking place. (B) Reactions are occurring.

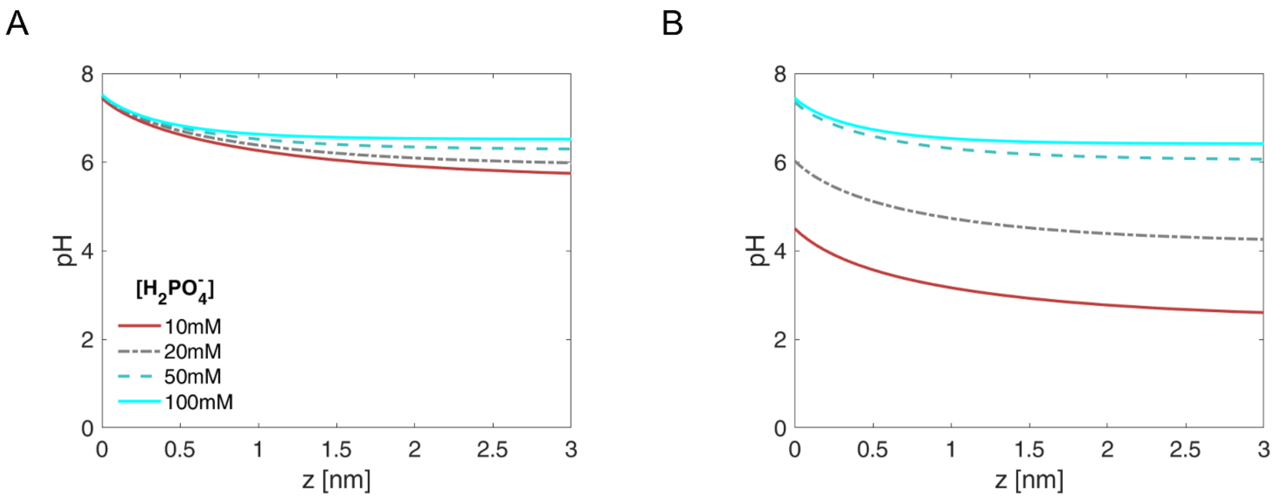

Figure S9. pH close to the surfactant monolayer depending on the initial concentration of the phosphate buffer ($c_{0\,H_2PO_4^-} = c_{0\,HPO_4^{2-}} = 10, 20, 50, 100\ mM$). Initial concentration of the electron relay $c_{0\,Q} = c_{0\,Q^-} = 20\ mM$ at the CO_2 reduction side. (A) Reactions are not taking place. (B) Reactions are occurring.

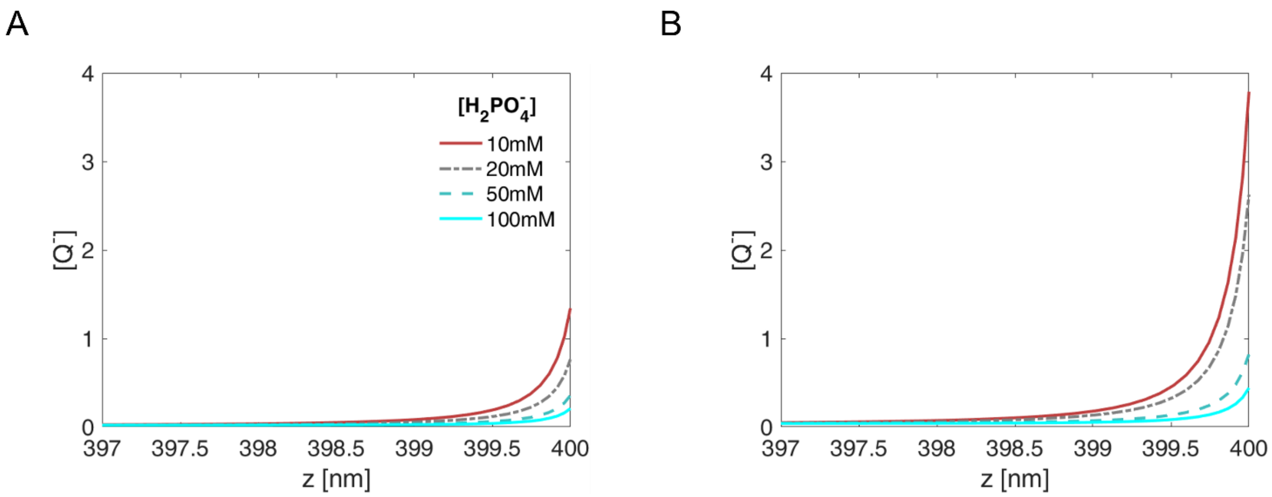

Figure S10. Molar concentration of Q- close to the surfactant monolayer depending on the initial concentration of the phosphate buffer ($c_{0\,H_2PO_4^-} = c_{0\,HPO_4^{2-}} = 10, 20, 50, 100\ mM$). Initial concentration of the electron relay $c_{0\,Q} = c_{0\,Q^-} = 20\ mM$ at the water oxidation side. (A) Reactions are not taking place. (B) Reactions are occurring.

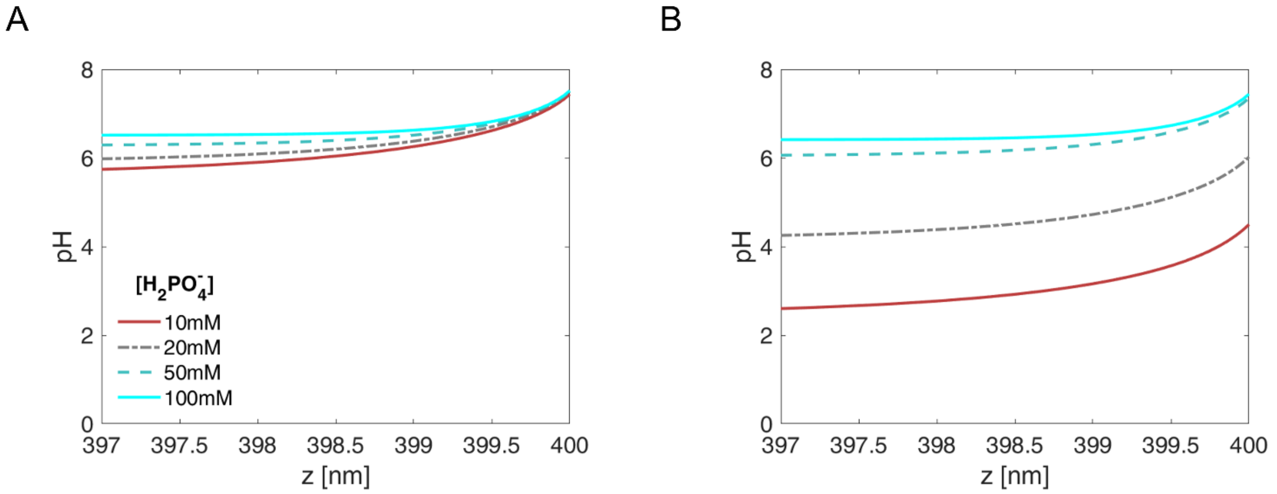

Figure S11. pH close to the surfactant monolayer depending on the initial concentration of phosphate buffer ($c_{0\,H_2PO_4^-} = c_{0\,HPO_4^{2-}} = 10, 20, 50, 100\ mM$). Initial concentration of the electron relay $c_{0\,Q} = c_{0\,Q^-} = 20\ mM$ at the water oxidation side. (A) Reactions are not taking place. (B) Reactions are occurring.

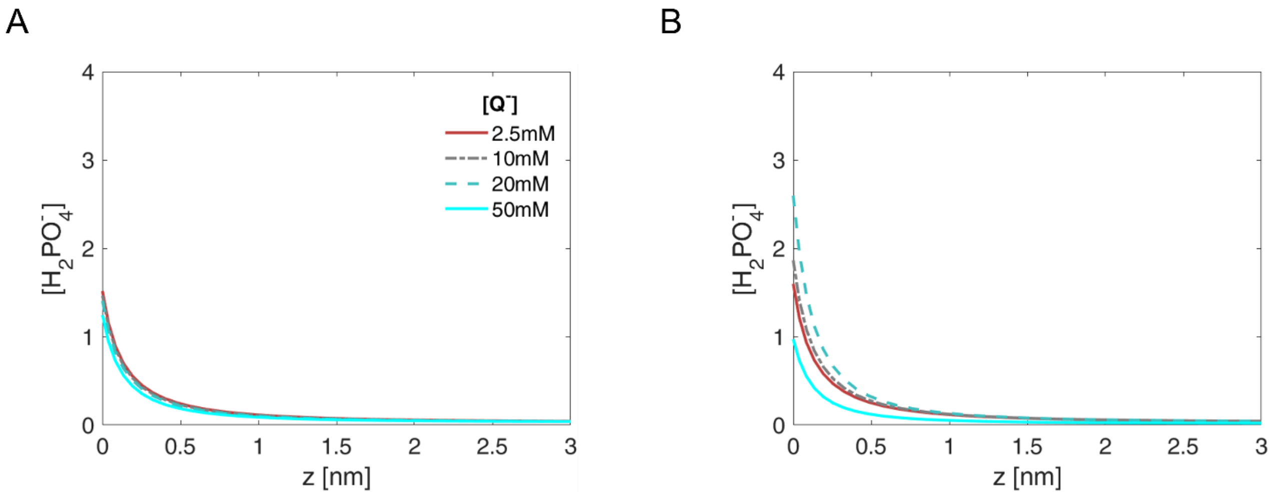

Figure S12. Molar concentration of Q- close to the surfactant monolayer depending on the initial concentration of the electron relay $c_{0\,Q} = c_{0\,Q^-} = 2.5, 10, 20, 50\ mM$. Initial concentration of the buffer $c_{0\,H_2PO_4^-} = c_{0\,HPO_4^{2-}} = 20\ mM$ at the CO_2 reduction side. (A) Reactions are not taking place. (B) Reactions are occurring.

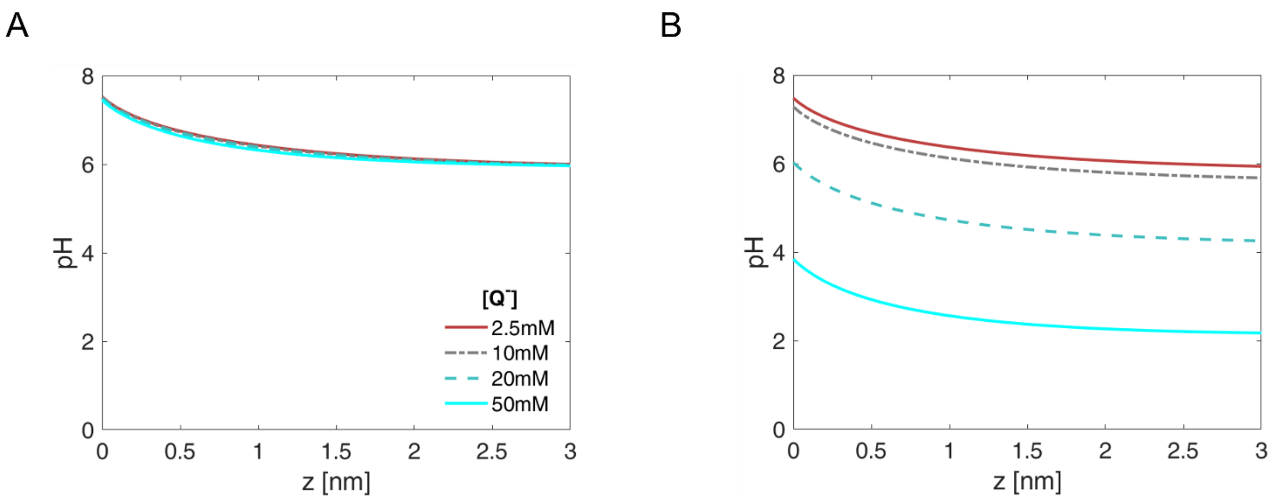

Figure S13. pH close to the surfactant monolayer depending on the initial concentration of the electron relay ($c_{0\,Q} = c_{0\,Q^-} = 2.5, 10, 20, 50\ mM$). Initial concentration of the buffer $c_{0\,H_2PO_4^-} = c_{0\,HPO_4^{2-}} = 20\ mM$ at the CO_2 reduction side. (A) Reactions are not taking place. (B) Reactions are occurring.

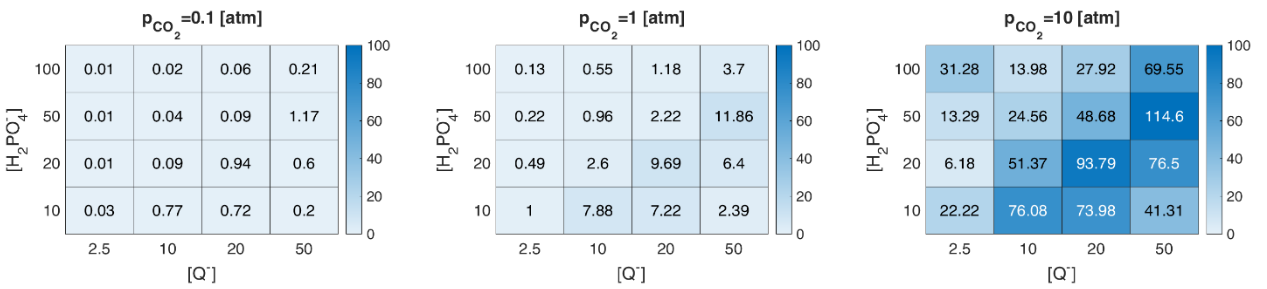

Figure S14. Normalized CO production. The initial molar concentration of the buffer ($c_{0\,H_2PO_4^-} = c_{0\,HPO_4^{2-}} = 10, 20, 50, 100\ mM$), of the electron relays ($c_{0\,Q} = c_{0\,Q^-} = 2.5, 10, 20, 50\ mM$) and the external CO_2 pressure ($p_{CO_2} = 0.1, 1, 10\ atm$) is varied. The flux is normalized to the 10:1 case for $c_{0\,Q} = c_{0\,Q^-} = 2\ mM$, $c_{0\,H_2PO_4^-} = c_{0\,HPO_4^{2-}} = 10\ mM$ and $p_{CO_2} = 1\ atm$ $(dn/dt = 3.599 \cdot 10^{-7}\ mol/m^2s)$. The soap film thickness is 400 nm.

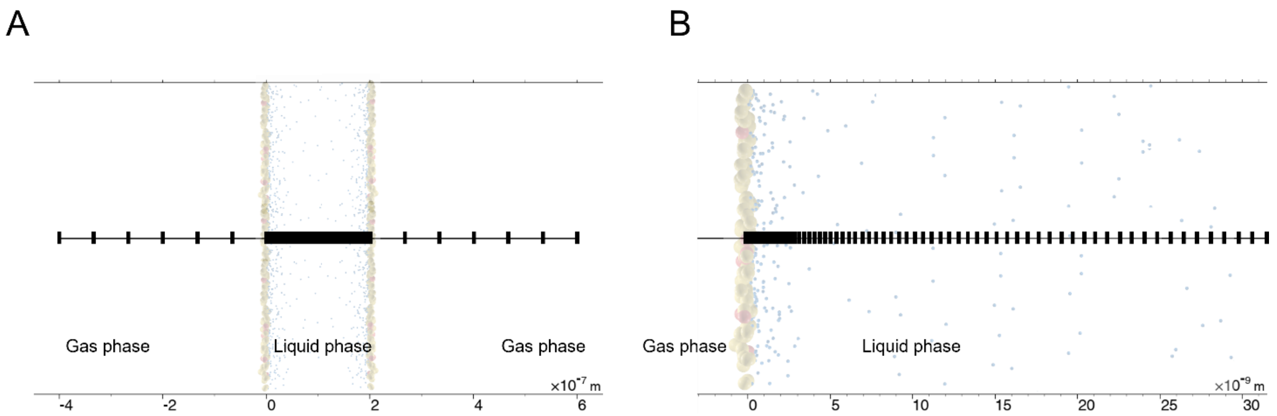

Figure S15. 1D Mesh. (A) Whole 1D domain. (B) Zoom at the gas water interface ($z = 0\ nm$). On the background the surfactant monolayers are depicted to guide the eye.

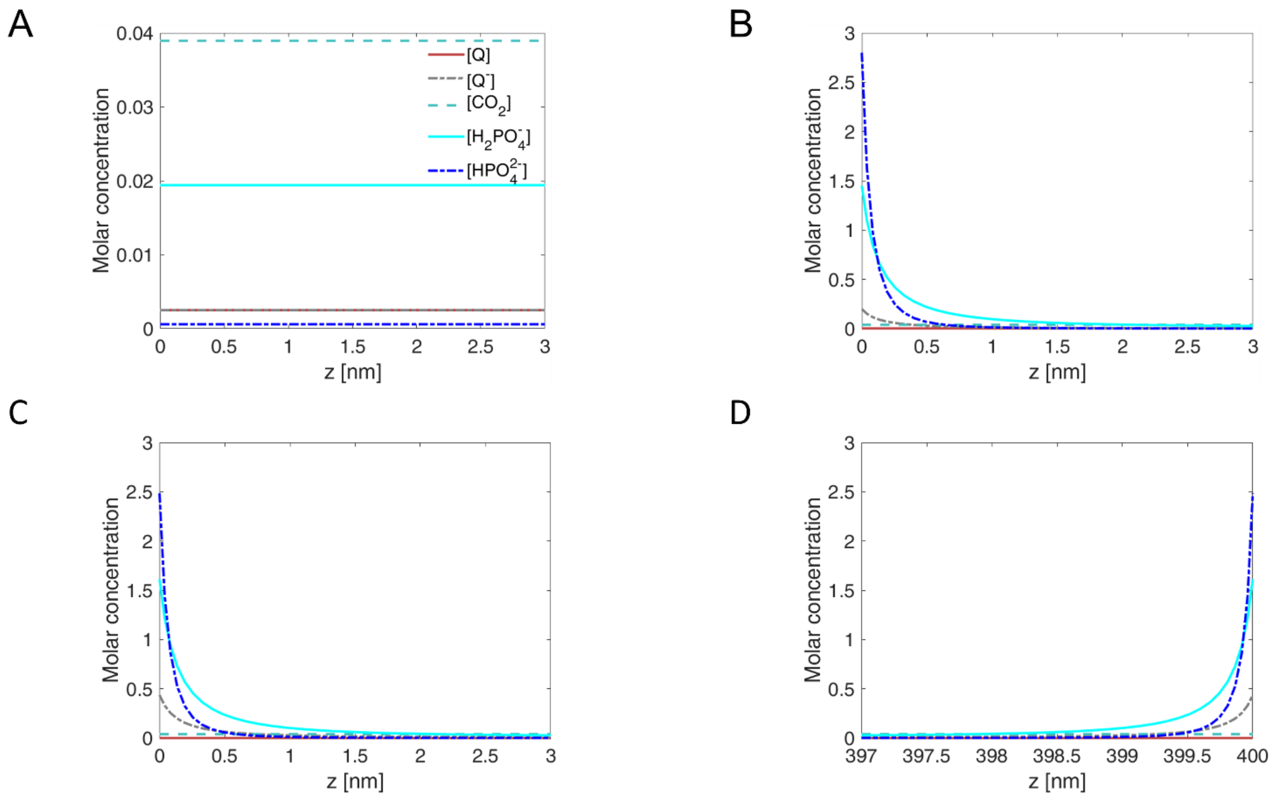

Figure S16. Molar concentration of the reactants in the water core for the following initial conditions: $\boldsymbol{c_{0\,H_2PO_4^-} = c_{0\,HPO_4^{2-}} = 10\,mM,\ \ c_{0\,Q} = c_{0\,Q^-} = 2.5\,mM,\ \ p_{CO_2} = 1\,atm.}$ (A) Concentration profile after the equilibration phase. (B) Concentration profile after the calculation of the electrostatic interactions. (C) Stationary solution once CO and O_2 are being produced on the CO_2 reduction side. (D) Stationary solution once CO and O_2 are being produced on the water oxidation side.

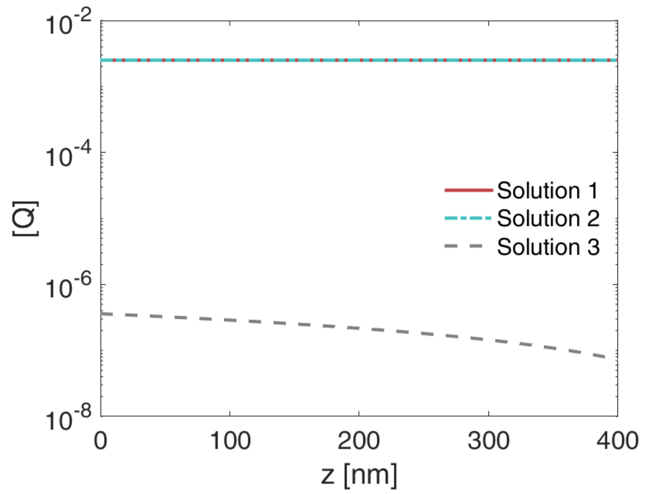

Figure S17. Molar concentration of the neutral electron relay Q in the water core. The following initial conditions were applied: $c_{0\,H_2PO_4^-} = c_{0\,HPO_4^{2-}} = 10$, $c_{0\,Q} = c_{0\,Q^-} = 2.5\,mM$, $p_{CO_2} = 1\,atm$. Solution 1 shows the concentration profile after the equilibration phase, Solution 2 the concentration profile after the calculation of the electrostatic interactions, Solution 3 is the stationary solution once the reactions are producing CO and O_2. The Q are produced from the carbon dioxide reduction side (z = 0 nm) and consumed in the water oxidation side (z = 400 nm).

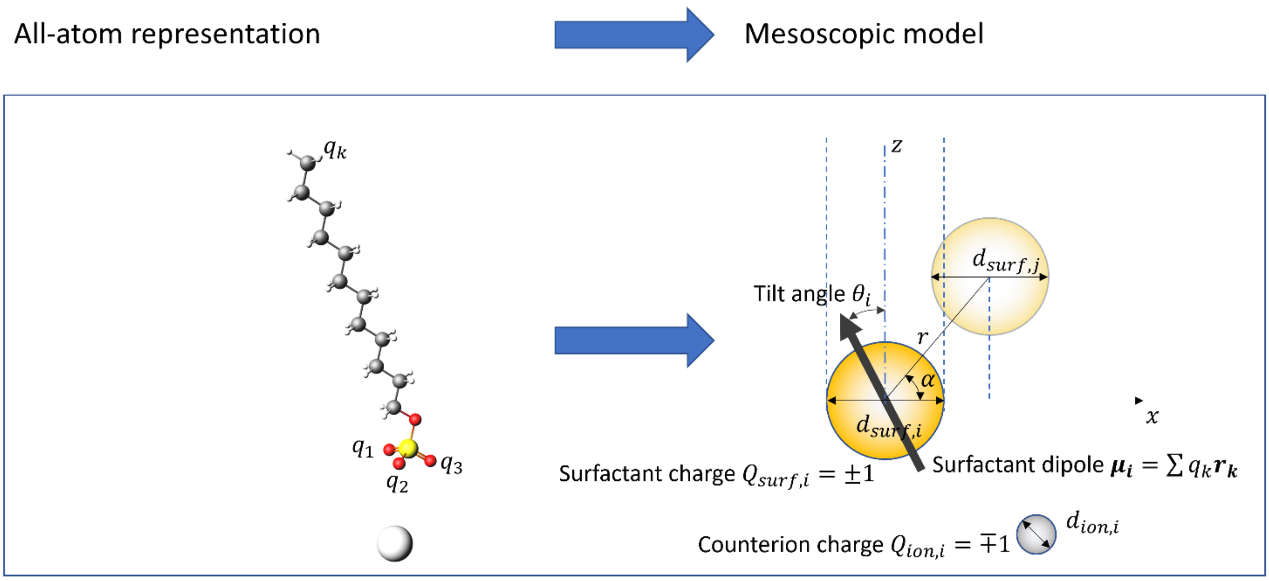

Figure S18. Coarse graining of the surfactant molecule. On the left: the all-atom molecular structure of the surfactant sodium dodecyl sulfate. The spheres in grey and white composing the tail are the carbon and hydrogen atoms. The surfactant head is composed of one sulfur (in yellow) and four oxygen atoms. The sodium counterion is depicted in white. On the right: coarse grained surfactant approximation used in the mesoscopic model. The head is approximated as a sphere of diameter d_{surf} with a charge Q_{surf} and a dipole μ, which oscillates with an angle θ due to thermal agitation around its z axis. The tails are considered with an excluded volume on the sphere, to avoid overlap of the surfactants. The ion is considered as a sphere with diameter d_{ion} and a charge Q_{ion}.

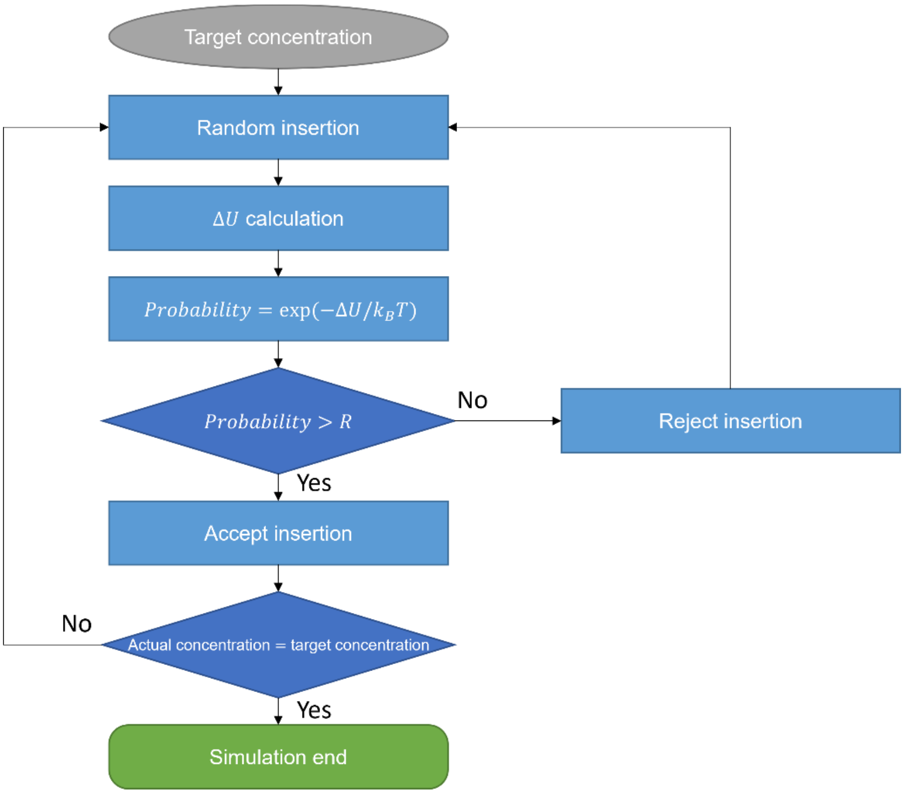

Figure 19. Flow chart of the Metropolis Monte Carlo (MMC) algorithm: 1) Randomly insert one surfactant (with its counterions if the surfactant is ionic); 2) Compute the energy variation of the system due to the particle insertion; 3) Compute the probability of retaining the inserted particles, namely: $Probability = \exp(-\Delta U/k_B T)$, where ΔU is the free energy variation of the system after the new molecule has been added; 4) Draw a random

number (R) from 0 to 1; 5) Accept if: $Probability > R$, else reject; 6) If the target surface concentration is not yet achieved, re-start from step 1, else terminate the procedure.

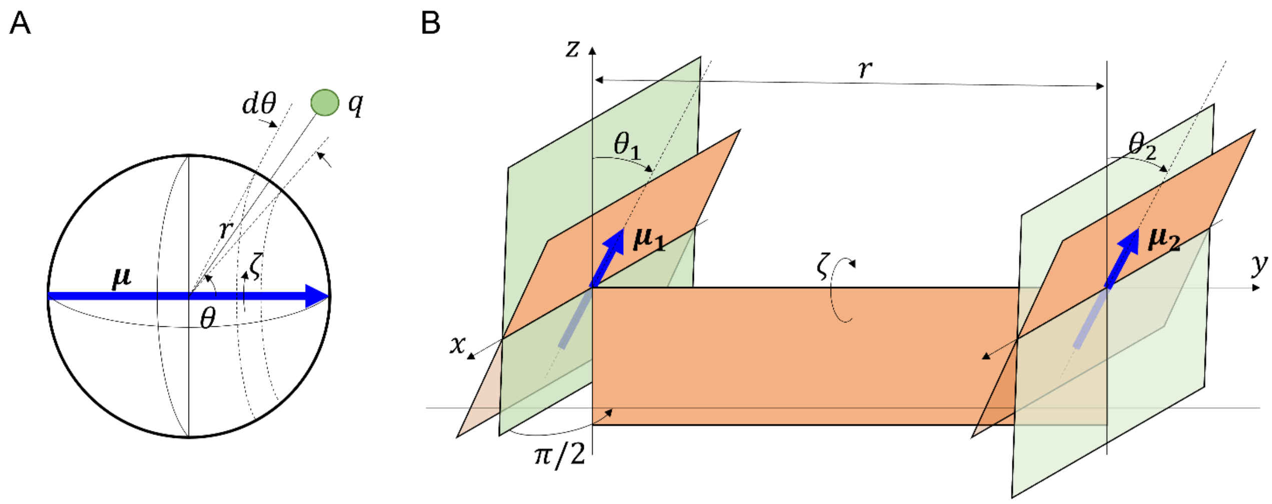

Figure S20. Schematics of the dipole interactions considered. (A) charge dipole interaction. A generic dipole oscillating around its axis with an angle ζ and perpendicular to its axis with an angle θ is depicted as a blue arrow. A point charge distant r from the dipole is depicted in green. (B) dipole-dipole interaction. Two dipoles, $\boldsymbol{\mu_1}$ and $\boldsymbol{\mu_2}$, free to oscillate with an angle θ_1 and θ_2 with respect to the z-axis are represented. The two dipoles are twisted by an angle ζ in the x-z plane and distant r. Image inspired by (4).

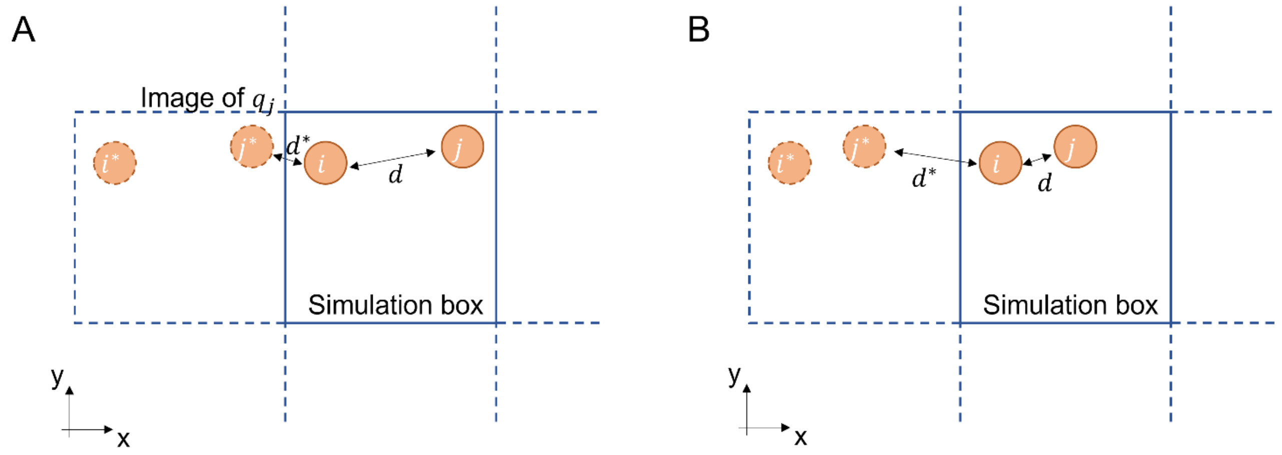

Figure S21. Periodic boundary conditions. A particle i interacts with the nearest image of a particle j: (A) The interaction energy between i and j is calculated at a distance d^*; (B) the interaction energy between i and j is calculated at a distance d.

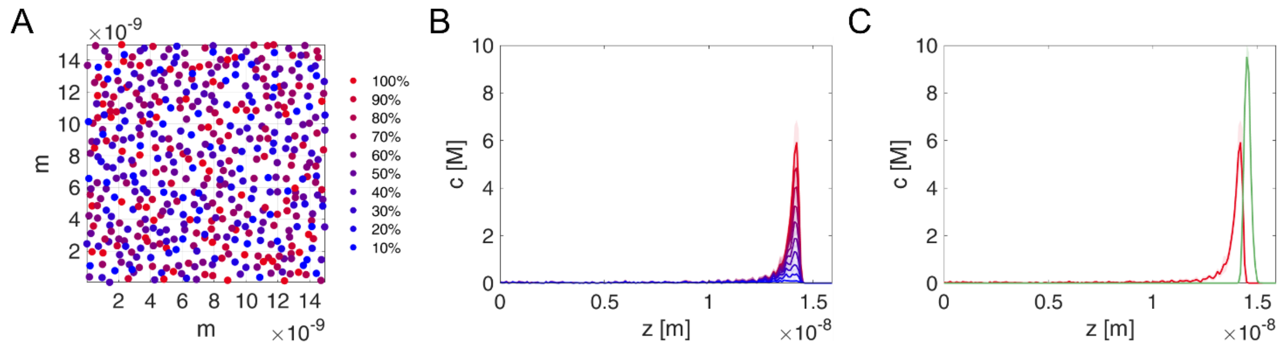

Figure S22. Output of the simulations for sodium dodecyl sulfate (SDS). (A) Top view of the monolayer configuration for a single microstate depending on the surface concentration. (B) Average molar concentration of the ions along the z axis in the bulk (perpendicular axis with respect to the surface) depending on the surface concentration. (C) Average ions (in red) and head-group molar concentration (in green) along the z axis in the bulk for the maximum coverage (100%), corresponding to a surface concentration $\Gamma = 3.69 \cdot 10^{-6}\ mol\ m^{-2}$.

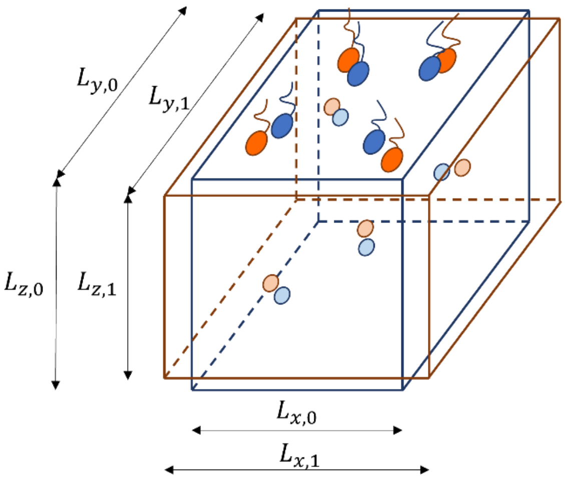

Figure S23. Geometric transformation applied to the simulation box. The subscript 0 and 1 indicate the unperturbed state (shown in blue) and the perturbed state (shown in orange), respectively. The ellipses with a tail are a schematic representation of the surfactants, while the ellipse alone represent the counterions.

Table S1. Diffusion coefficients of the species in solution

Species	Diffusion coefficient $\cdot$ 10^9 $(m^2 s^{-1})$	Reference
O_2	2.01	(5)
CO_2	1.91	(5)
CO	2.03	(6)
Q	1	-
Q^-	1	-
HCO_3^-	1.18	(5)
CO_3^{2-}	0.92	(5)
H_3PO_4	1	(5)
$H_2PO_4^-$	0.96	(5)
HPO_4^{2-}	0.76	(5)
PO_4^{3-}	0.82	(5)
H^+	9.31	(5)
OH^-	5.27	(5)
Na^+	1.33	(5)
Cl^-	2.03	(5)

Table S2. Self-diffusion coefficients of the gaseous species

Species	Diffusion coefficient $\cdot$ $10^5(m^2 s^{-1})$	Reference
O_2	2.09	(7)
CO_2	1.13	(8)
CO	1.9	(9)

Table S3. Henry's law constants

Species	Henry's law constant $(-)$	Reference
O_2	0.0336	(7)
CO_2	0.9520	(8)
CO	0.0256	(9)

Table S4. Monolayer permeability for different gases

Species	Monolayer permeability $(\boldsymbol{m\,s^{-1}})$	Reference
O_2	0.188	(10)
CO_2	0.164	(10)
CO	= O_2	Experimental data not available

Table S5. Fitting parameters for gas adsorption (Langmuir model) at the gas-water interface (see the related Figure S1).

Gas	$\boldsymbol{K_{eq}}$ $(\boldsymbol{m^3/mol})$	$\boldsymbol{\Gamma_s}$ $(\boldsymbol{mol/m^2})$
H_2	$8.22 \cdot 10^{-6}$	$3.04 \cdot 10^{-5}$
O_2	$7.93 \cdot 10^{-5}$	$9.77 \cdot 10^{-6}$
CO	$1.60 \cdot 10^{-4}$	$6.59 \cdot 10^{-6}$
CO_2	$1.26 \cdot 10^{-4}$	$5.24 \cdot 10^{-5}$

Table S6. Characteristics of the photosensitizer (PS) for the mesoscale model

		**Notes**
d_{surf}	0.7 nm	Geometric diameter of the head
d_{ion}	0.18 nm	Geometric diameter of the ion
Q_{surf}	-2e	Charge of the head. e = elementary charge
Q_{ion}	e	Charge of the ion. e = elementary charge, 2 ions per head
μ	-9 D	Surfactant dipole
Cutoff radius for dipole-dipole interactions	5 nm	
Tilt angle	37.1°	
m	-0.4	Mean parameter. Gaussian distribution along z of the heads
σ	0.15	Standard deviation. Gaussian distribution along z of the heads

Table S7. Characteristics for the neutral catalyst C for the mesoscale model

		**Notes**
d_{surf}	0.7 nm	Geometric diameter of the head
d_{ion}		Geometric diameter of the ion
μ	-9 D	Surfactant dipole
Cutoff radius for dipole-dipole interactions	5 nm	
Tilt angle	37.1°	
m	-0.4	Mean parameter. Gaussian distribution along z of the heads
σ	0.15	Standard deviation. Gaussian distribution along z of the heads

Table S8. 1D mesh details. L is the thickness of the soap film and round is a function that approximate the result to the closest integer.

Domain	Number of elements	Element ratio
Gas phases	250*round(L/200e-9)	40
Liquid phase	10*round(L/200e-9)	1

Table S9. Characteristics for the SDS molecule for the mesoscale model

		Notes
d_{surf}	0.4 nm	Geometric diameter of the head
d_{ion}	0.18 nm	Geometric diameter of the ion
Q_{surf}	-e	Charge of the head. e = elementary charge
Q_{ion}	e	Charge of the ion. e = elementary charge
μ	-9 D	Surfactant dipole, best fitting
Cutoff radius for dipole-dipole interactions	5 nm	
Tilt angle	37.1°	(11)
m	-0.4	Mean parameter. Gaussian distribution along z of the heads (12)
σ	0.15	Standard deviation. Gaussian distribution along z of the heads (12)

References

1. **Timounay, Yousra and Pannwitz, Andrea and Klein, David M and Biance, Anne-Laure and Hoefnagel, Marlene E and Sen, Indraneel and Cagna, Alain and Le Merrer, Marie and Bonnet, Sylvestre.** Interfacial Characterization of Ruthenium-Based Amphiphilic Photosensitizers. *Langmuir.* 2022, Vol. 38, 31, pp. 9697--9707.

2. **Massoudi, R and King Jr, AD.** Effect of pressure on the surface tension of water. Adsorption of low molecular weight gases on water at 25. deg. *The Journal of Physical Chemistry.* 1974, Vol. 78, 22, pp. 2262--2266.

3. **Martinez-Balbuena, L and Arteaga-Jimenez, Araceli and Hernandez-Zapata, Ernesto and Marquez-Beltran, Cesar.** Applicability of the Gibbs Adsorption Isotherm to the analysis of experimental surface-tension data for ionic and nonionic surfactants. *Advances in colloid and interface science.* 2017, Vol. 247, pp. 178--184.

4. **Reifenberger, Ronald.** Fundamentals of Atomic Force Microscopy: Part I: Foundations. 2016.

5. **Lide, D. R.** CRC handbook of chemistry and physics. 2004, Vol. 85.

6. **D.L. Wise, G. Houghton.** Diffusion coefficients of neon, krypton, xenon, carbon monoxide and nitric oxide in water at 10-60 C. *Chemical Engineering Science.* 1968, Vol. 23, 10, pp. 1211--1216.

7. **Landolt-Börnstein.** Group IV Physical Chmistry Diffusion of oxygen. *Gases in Gases, Liquids and their Mixtures.* 2007.

8. **Winn.** The temperature dependence of the self-diffusion coefficients of argon, neon, nitrogen, oxygen, carbon dioxide, and methane. *Physical review,.* 1950, Vol. 80, 6, p. 1024.

9. **Amdor, Shuler.** Diffusion Coefficients of the Systems CO–CO and CO–N2. *The Journal of Chemical Physics.* 1963, Vol. 38, pp. 188--192.

10. **Princen, HM and Mason, SG.** The permeability of soap films to gases. *Journal of Colloid Science.* 1965, Vol. 20, 4, pp. 353-375.

11. **Pang, Jinyu and Xu, Guiying.** Comparison of the influence of fluorocarbon and hydrocarbon surfactants on the adsorptions of SDS, DTAB and C12E8 at the air/water interface by MD simulation. *Chemical Physics Letters.* 2012, Vol. 537, pp. 118--125.

12. **Farajzadeh, R and Krastev, R and Zitha, Pacelli LJ.** Foam film permeability: Theory and experiment. *Advances in colloid and interface science.* 2008, Vol. 137, 1, pp. 27-44.
