# Supplementary material for: A novel concept of photosynthetic soft membranes: a numerical study
Source: Discov Nano. 2023 Feb 9;18(1):9. doi: 10.1186/s11671-023-03772-1 (PMC9911585; doi:10.1186/s11671-023-03772-1)
Supplement: Supplementary file 2 — Supplementary file2 (ZIP 2426 KB) [file 11671_2023_3772_MOESM2_ESM.zip › Data_S1/soap_film_comsol.pdf]

Soapfilm

Report date	May 26, 2022, 10:13:26 AM

Contents

1. Global Definitions 3
1.1. Parameters 3
1.2. Shared Properties 6
2. Component 1 7
2.1. Definitions 7
2.2. Geometry 1 9
2.3. Electrostatics 10
2.4. Transport of Diluted Species 28
2.5. Transport of Diluted Species CO2 side 116
2.6. Transport of Diluted Species H2O side 136
2.7. Surface Reactions CO2 reduction 156
2.8. Surface Reactions H2O oxidation 162
2.9. Chemistry CO2 reduction 167
2.10. Chemistry H2O oxidation 221
2.11. Chemistry equilibria 264
2.12. Multiphysics 336
2.13. Mesh 1 341

1 Global Definitions

Date	May 16, 2022, 3:21:49 PM

GLOBAL SETTINGS

Name	Soapfilm.mph
Path	C:\comsol\soapfilm.mph
Version	COMSOL Multiphysics 6.0 (Build: 354)
Unit system	SI

USED PRODUCTS

COMSOL Multiphysics
Chemical Reaction Engineering Module

COMPUTER INFORMATION

CPU	Intel64 Family 6 Model 85 Stepping 4, 16 cores
Operating system	Windows 10

1.1 PARAMETERS

PARAMETERS 1

**Name**	**Expression**	**Value**	**Description**
c0	1[atm]/(R_const*Temp)	40.895 mol/m³	Initial concentration
c0_B	10 [mol/m^3]	10 mol/m³	
c0_ER	2.5 [mol/m^3]	2.5 mol/m³	
D_Clw	2.032e-9 [m^2/s]	2.032E−9 m²/s	lide handbook of chemistry and phyiscs
D_CO	1.9e-5[m^2/s]	1.9E−5 m²/s	
D_CO2	1.35e-5[m^2/s]	1.35E−5 m²/s	
D_CO2w	1.67e-9[m^2/s]	1.67E−9 m²/s	
D_CO3w	0.923E-9 [m^2/s]	9.23E−10 m²/s	lide handbook of chemistry and phyiscs
D_COw	2.03e-9[m^2/s]	2.03E−9 m²/s	
D_H2	1.24e-4[m^2/s]	1.24E−4 m²/s	
D_H2Ow	2e-9[m^2/s]	2E−9 m²/s	
D_H2PO4w	0.959E-9 [m^2/s]	9.59E−10 m²/s	lide handbook of chemistry and phyiscs
D_H2Qw	1e-10[m^2/s]	1E−10 m²/s	
D_H2w	5e-9[m^2/s]	5E−9 m²/s	
D_H3PO4w	1e-9 [m^2/s]	1E−9 m²/s	edwards Diffusion of

Name	Expression	Value	Description
			Aqueous Solutions of Phosphoric Acid
D_HCO3w	1.185E-9 [m^2/s]	1.185E−9 m²/s	lide handbook of chemistry and phyiscs
D_HPO4w	0.759E-9 [m^2/s]	7.59E−10 m²/s	lide handbook of chemistry and phyiscs
D_Hw	9.31e-9 [m^2/s]	9.31E−9 m²/s	lide handbook of chemistry and phyiscs
d_ion	5e-10 [m]	5E−10 m	
D_Naw	1.334e-9 [m^2/s]	1.334E−9 m²/s	lide handbook of chemistry and phyiscs
D_O2	2.09e-5[m^2/s]	2.09E−5 m²/s	
D_O2w	2.01e-9[m^2/s]	2.01E−9 m²/s	
D_OHw	5.27e-9 [m^2/s]	5.27E−9 m²/s	lide handbook of chemistry and phyiscs
D_PO4w	0.824e-9 [m^2/s]	8.24E−10 m²/s	lide handbook of chemistry and phyiscs
D_Qw	1e-10[m^2/s]	1E−10 m²/s	
Dw	1e-9[m^2/s]	1E−9 m²/s	Gas diffusivity
eps_H2O	80	80	
Gamma_CAT	7.3803e-8	7.3803E−8	
Gamma_s	Gamma_sO2	9.77E−6 mol/m²	Active site concentration
Gamma_sCO	6.59e-6 [mol/m^2]	6.59E−6 mol/m²	
Gamma_sCO2	5.24e-5[mol/m^2]	5.24E−5 mol/m²	
Gamma_sH2	3.04e-5 [mol/m^2]	3.04E−5 mol/m²	
Gamma_sO2	9.77e-6 [mol/m^2]	9.77E−6 mol/m²	
H_CO	2.56E-2	0.0256	deliverable
H_CO2	0.952	0.952	
H_O2	0.0328	0.0328	
k_ads	k_adsCO	1.6	
k_adsCO	KH_CO*k_desCO	1.6	
k_adsCO2	KH_CO2*k_desCO2	1.26	
k_adsH2	KH_H2*k_desH2	0.0822	
k_adsO2	KH_O2*k_desO2	0.793	
k_CO	k_H2	0.593 m/s	
k_CO2	0.164[m/s]	0.164 m/s	
K_CO2	10^(-6.3 + 3)	5.0119E−4	Wolf-Gladrow

Name	Expression	Value	Description
K_CO2_OHkf	2.23E3[1/(s*M)]	2.23 $m^3/(s\cdot mol)$	
K_CO2kf	3.71e-2[1/s]	0.0371 1/s	
k_des	k_desCO2	10000	
k_desCO	k_desCO2	10000	
k_desCO2	1e4	10000	
k_desH2	k_desCO2	10000	
k_desO2	k_desCO2	10000	
k_H2	0.593[m/s]	0.593 m/s	
K_H2O	10^(-14 + 6)	1E−8	water dissociation
K_H2PO4	10^(-7.2 + 3)	6.3096E−5	smith critical stability constants
K_H3PO4	10^(-2.14 + 3)	7.2444	smith critical stability constants
K_HCO3	10^(-10.3 + 3)	5.0119E−8	Wolf-Gladrow
K_HCO3_OHkf	6E9[1/(s*M)]	6E6 $m^3/(s\cdot mol)$	
K_HCO3kf	59.44[1/s]	59.44 1/s	
K_HPO4	10^(-12.37 + 3)	4.2658E−10	smith critical stability constants
k_O2	k_H2	0.593 m/s	
k1_CO	H_CO*k_CO	0.015181 m/s	
k1_CO2	H_CO2*k_CO2	0.15613 m/s	
k1_O2	H_O2*k_O2	0.01945 m/s	
kf_CO2	m_CO2*PS_close_CAT*photon_flux*Gamma_CAT	2E−6	
kf_H2O	m_H2O*PS_close_CAT*photon_flux*Gamma_CAT	2E−6	
KH	KH_CO2	1.26E−4	
KH_CO	1.6e-4	1.6E−4	
KH_CO2	1.26e-4	1.26E−4	
KH_H2	8.22e-6	8.22E−6	
KH_O2	7.93E-5	7.93E−5	
ki	((2*c0_ER*N_A_const*e_const^2)/(eps_H2O*epsilon0_const*k_B_const*Temp))^0.5	1.6286E8 1/m	
L	400e-9[m]	4E−7 m	
m_CO2	6.7918	6.7918	
m_H2O	6.7918	6.7918	

Name	Expression	Value	Description
photon_flux	1	1	
PM_Asc	175.12 [g/mol]	0.17512 kg/mol	
PM_Ascm	175.12 [g/mol]	0.17512 kg/mol	
PM_CO	28[g/mol]	0.028 kg/mol	
PM_CO2	44[g/mol]	0.044 kg/mol	
PM_CO3	60 [g/mol]	0.06 kg/mol	
PM_H	1[g/mol]	0.001 kg/mol	
PM_H2O	18[g/mol]	0.018 kg/mol	
PM_H2PO4	97[g/mol]	0.097 kg/mol	
PM_H2Q	110[g/mol]	0.11 kg/mol	
PM_H3PO4	98[g/mol]	0.098 kg/mol	
PM_HCO3	61 [g/mol]	0.061 kg/mol	
PM_HPO4	96[g/mol]	0.096 kg/mol	
PM_HQ	109[g/mol]	0.109 kg/mol	
PM_Na	23[g/mol]	0.023 kg/mol	
PM_O2	32[g/mol]	0.032 kg/mol	
PM_OH	17[g/mol]	0.017 kg/mol	
PM_PO4	95[g/mol]	0.095 kg/mol	
PM_Q	108[g/mol]	0.108 kg/mol	
PS_close_CAT	3.99	3.99	
rho_s	0.01 [C/m^2]	0.01 C/m²	
Temp	298[K]	298 K	
Z_Asc	0	0	
Z_Ascm	-1	−1	

1.2 SHARED PROPERTIES

1.2.1 Default Model Inputs

Tag	cminpt

2 Component 1

Date	Mar 30, 2022, 6:05:05 PM

SETTINGS

Description	Value
Unit system	Same as global system (SI)
Geometry shape function	Automatic

SPATIAL FRAME COORDINATES

First	Second	Third
x	y	z

MATERIAL FRAME COORDINATES

First	Second	Third
X	Y	Z

GEOMETRY FRAME COORDINATES

First	Second	Third
Xg	Yg	Zg

MESH FRAME COORDINATES

First	Second	Third
Xm	Ym	Zm

2.1 DEFINITIONS

2.1.1 Variables

Variables 1

SELECTION

Geometric entity level	Entire model

Name	Expression	Unit	Description
rho_surf	0.142[C/m^2]	C/m^2	
rho_surf_left	rho_surf	C/m^2	
rho_surf_right	rho_surf	C/m^2	

2.1.2 Functions

Step 1

Function name	step1
Function type	Step

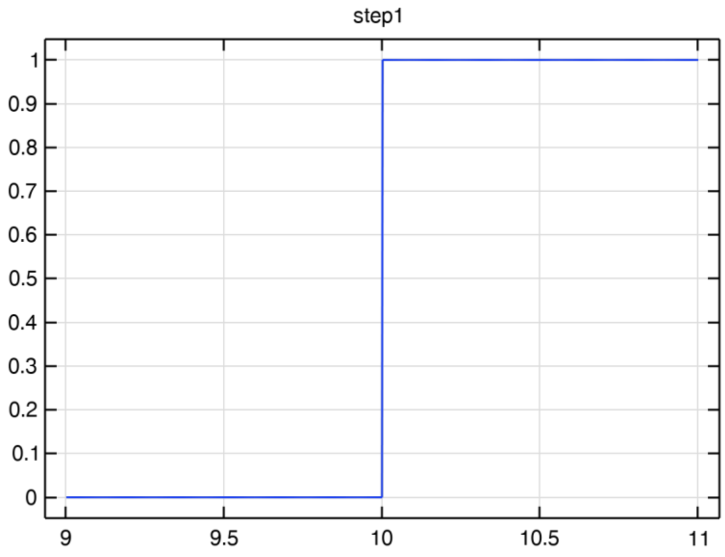

Step 1

PARAMETERS

Description	Value
Location	10 + 1.5e-3
From	0
To	1

SMOOTHING

Description	Value
Size of transition zone	Off

2.1.3 Nonlocal Couplings

Average 1

Coupling type	Average
Operator name	aveop1

SELECTION

Geometric entity level	Domain
Selection	Geometry geom1: Dimension 1: Domain 2

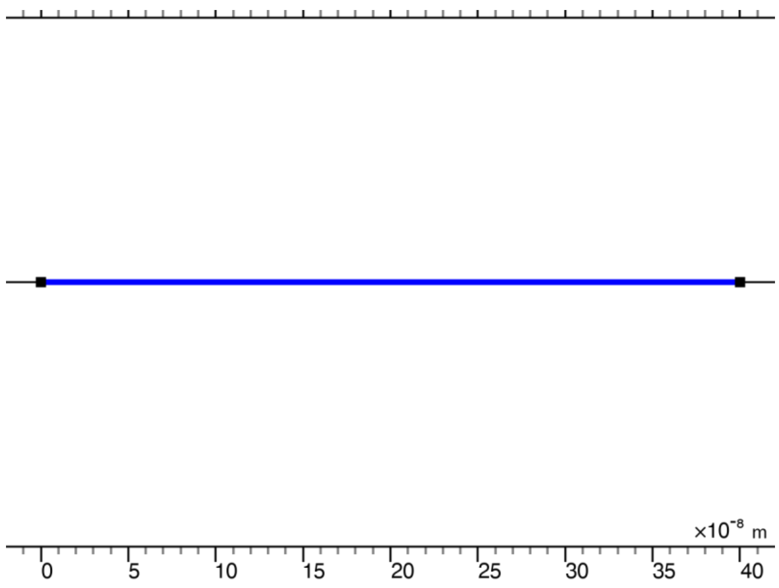

Selection

2.2 GEOMETRY 1

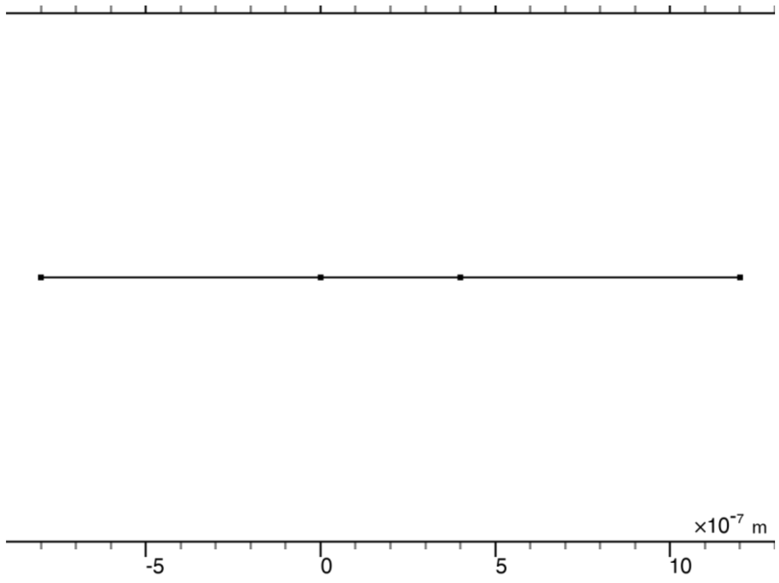

Geometry 1

UNITS

Length unit	m
Angular unit	deg

GEOMETRY STATISTICS

Description	Value
Space dimension	1

Description	Value
Number of domains	3
Number of boundaries	4

2.2.1 Interval 1 (i1)

INTERVAL

Coordinates (m)
0
L

2.2.2 Interval 2 (i2)

INTERVAL

Coordinates (m)
-2*L
0

2.2.3 Interval 3 (i3)

INTERVAL

Coordinates (m)
L
3*L

2.3 ELECTROSTATICS

USED PRODUCTS

COMSOL Multiphysics

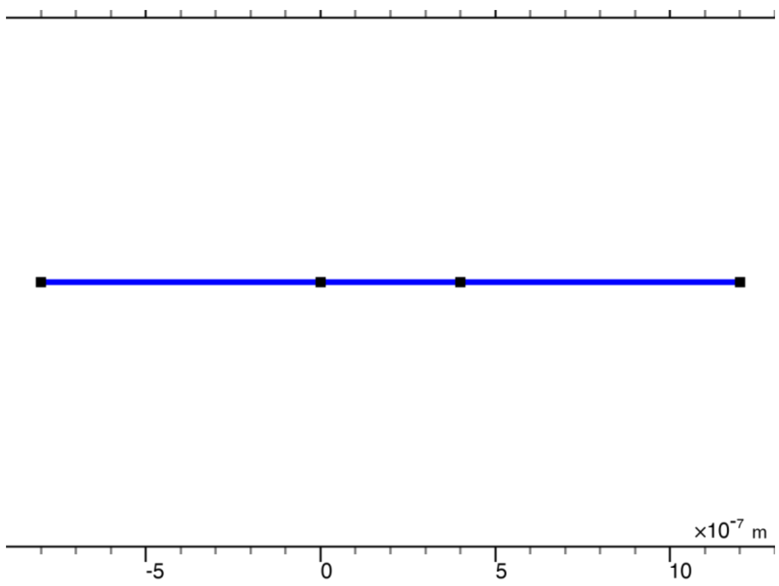

Electrostatics

SELECTION

Geometric entity level	Domain
Selection	Geometry geom1: Dimension 1: All domains

EQUATIONS

$$\nabla \cdot \mathbf{D} = \rho_{\mathrm{v}}$$
$$\mathbf{E} = -\nabla V$$

2.3.1 Interface Settings

Discretization

SETTINGS

**Description**	**Value**
Electric potential	Quadratic

SETTINGS

**Description**	**Value**
Cross-section area	1

Manual Terminal Sweep Settings

SETTINGS

**Description**	**Value**
Use manual terminal sweep	Off
Reference impedance	50[ohm]

2.3.2 Variables

Name	Expression	Unit	Description	Selection	Details
es.d	1	m^2	Cross-section area	Domains 1–3	
es.nx	nx		Normal vector, x component	Boundaries 2–3	
es.ny	0		Normal vector, y component	Boundaries 2–3	
es.nz	0		Normal vector, z component	Boundaries 2–3	
es.nx	unx		Normal vector, x component	Boundary 1	
es.ny	0		Normal vector, y component	Boundary 1	
es.nz	0		Normal vector, z component	Boundary 1	
es.nx	dnx		Normal vector, x component	Boundary 4	
es.ny	0		Normal vector, y component	Boundary 4	
es.nz	0		Normal vector, z component	Boundary 4	
es.nmeshx	nxmesh		Mesh normal vector, x component	Boundaries 2–3	
es.nmeshy	0		Mesh normal vector, y component	Boundaries 2–3	
es.nmeshz	0		Mesh normal vector, z component	Boundaries 2–3	
es.nmeshx	unxmesh		Mesh normal vector, x component	Boundary 1	
es.nmeshy	0		Mesh normal vector, y component	Boundary 1	
es.nmeshz	0		Mesh normal vector, z component	Boundary 1	
es.nmeshx	dnxmesh		Mesh normal vector, x	Boundary 4	

Name	Expression	Unit	Description	Selection	Details
			component		
es.nmeshy	0		Mesh normal vector, y component	Boundary 4	
es.nmeshz	0		Mesh normal vector, z component	Boundary 4	
es.unmeshx	unxmesh		Mesh normal vector, upside, x component	Boundaries 1–4	
es.unmeshy	0		Mesh normal vector, upside, y component	Boundaries 1–4	
es.unmeshz	0		Mesh normal vector, upside, z component	Boundaries 1–4	
es.dnmeshx	dnxmesh		Mesh normal vector, downside, x component	Boundaries 1–4	
es.dnmeshy	0		Mesh normal vector, downside, y component	Boundaries 1–4	
es.dnmeshz	0		Mesh normal vector, downside, z component	Boundaries 1–4	
es.I_sXX	spatial.invF11^2*es.I_sxx*spatial.detF	1	Spatial identity matrix, material frame, XX component	Domains 1–3	
es.I_sYX	spatial.invF11*es.I_syx*spatial.detF	1	Spatial identity matrix, material frame, YX component	Domains 1–3	
es.I_sZX	spatial.invF11*es.I_szx*spatial.detF	1	Spatial identity matrix, material frame, ZX component	Domains 1–3	
es.I_sXY	spatial.invF11*es.I_sxy*spatial.detF	1	Spatial identity matrix, material frame, XY	Domains 1–3	

Name	Expression	Unit	Description	Selection	Details
			component		
es.I_sYY	es.I_syy*spatial.detF	1	Spatial identity matrix, material frame, YY component	Domains 1–3	
es.I_sZY	es.I_szy*spatial.detF	1	Spatial identity matrix, material frame, ZY component	Domains 1–3	
es.I_sXZ	spatial.invF11*es.I_sxz*spatial.detF	1	Spatial identity matrix, material frame, XZ component	Domains 1–3	
es.I_sYZ	es.I_syz*spatial.detF	1	Spatial identity matrix, material frame, YZ component	Domains 1–3	
es.I_sZZ	es.I_szz*spatial.detF	1	Spatial identity matrix, material frame, ZZ component	Domains 1–3	
es.I_sxx	1	1	Spatial identity matrix, xx component	Domains 1–3	
es.I_syx	0	1	Spatial identity matrix, yx component	Domains 1–3	
es.I_szx	0	1	Spatial identity matrix, zx component	Domains 1–3	
es.I_sxy	0	1	Spatial identity matrix, xy component	Domains 1–3	
es.I_syy	1	1	Spatial identity matrix, yy component	Domains 1–3	
es.I_szy	0	1	Spatial identity matrix, zy component	Domains 1–3	
es.I_sxz	0	1	Spatial identity matrix, xz component	Domains 1–3	
es.I_syz	0	1	Spatial identity	Domains 1–3	

Name	Expression	Unit	Description	Selection	Details
			matrix, yz component		
es.I_szz	1	1	Spatial identity matrix, zz component	Domains 1–3	
es.unTx	es.unTex	Pa	Maxwell upward surface stress tensor, x component	Boundaries 1–4	
es.unTy	es.unTey	Pa	Maxwell upward surface stress tensor, y component	Boundaries 1–4	
es.unTz	es.unTez	Pa	Maxwell upward surface stress tensor, z component	Boundaries 1–4	
es.dnTx	es.dnTex	Pa	Maxwell downward surface stress tensor, x component	Boundaries 1–4	
es.dnTy	es.dnTey	Pa	Maxwell downward surface stress tensor, y component	Boundaries 1–4	
es.dnTz	es.dnTez	Pa	Maxwell downward surface stress tensor, z component	Boundaries 1–4	
es.unx	unx		Normal vector up direction, x component	Boundaries 1–4	
es.uny	0		Normal vector up direction, y component	Boundaries 1–4	
es.unz	0		Normal vector up direction, z component	Boundaries 1–4	
es.dnx	dnx		Normal vector down direction, x component	Boundaries 1–4	

Name	Expression	Unit	Description	Selection	Details
es.dny	0		Normal vector down direction, y component	Boundaries 1–4	
es.dnz	0		Normal vector down direction, z component	Boundaries 1–4	
es.unTex	-0.5*es.dnx*(real(up(es.Dx))*real(up(es.Ex))+real(up(es.Dy))*real(up(es.Ey))+real(up(es.Dz))*real(up(es.Ez)))+real(up(es.Dx))*(real(up(es.Ex))*es.dnx+real(up(es.Ey))*es.dny+real(up(es.Ez))*es.dnz)	Pa	Maxwell upward electric surface stress tensor, x component	Boundaries 1–3	
es.unTey	-0.5*es.dny*(real(up(es.Dx))*real(up(es.Ex))+real(up(es.Dy))*real(up(es.Ey))+real(up(es.Dz))*real(up(es.Ez)))+real(up(es.Dy))*(real(up(es.Ex))*es.dnx+real(up(es.Ey))*es.dny+real(up(es.Ez))*es.dnz)	Pa	Maxwell upward electric surface stress tensor, y component	Boundaries 1–3	
es.unTez	-0.5*es.dnz*(real(up(es.Dx))*real(up(es.Ex))+real(up(es.Dy))*real(up(es.Ey))+real(up(es.Dz))*real(up(es.Ez)))+real(up(es.Dz))*(real(up(es.Ex))*es.dnx+real(up(es.Ey))*es.dny+real(up(es.Ez))*es.dnz)	Pa	Maxwell upward electric surface stress tensor, z component	Boundaries 1–3	
es.unTex	0	Pa	Maxwell upward electric surface stress tensor, x component	Boundary 4	
es.unTey	0	Pa	Maxwell upward electric surface stress tensor, y component	Boundary 4	
es.unTez	0	Pa	Maxwell upward electric surface stress tensor, z component	Boundary 4	
es.dnTex	-0.5*es.unx*(real(down(es.Dx))*real(down(es.Ex))+real(dow	Pa	Maxwell downward electric surface	Boundaries 2–4	

Name	Expression	Unit	Description	Selection	Details
	n(es.Dy))*real(down(es.Ey))+real(down(es.Dz))*real(down(es.Ez)))+real(down(es.Dx))*(real(down(es.Ex))*es.unx+real(down(es.Ey))*es.uny+real(down(es.Ez))*es.unz)		stress tensor, x component		
es.dnTey	-0.5*es.uny*(real(down(es.Dx))*real(down(es.Ex))+real(down(es.Dy))*real(down(es.Ey))+real(down(es.Dz))*real(down(es.Ez)))+real(down(es.Dy))*(real(down(es.Ex))*es.unx+real(down(es.Ey))*es.uny+real(down(es.Ez))*es.unz)	Pa	Maxwell downward electric surface stress tensor, y component	Boundaries 2–4	
es.dnTez	-0.5*es.unz*(real(down(es.Dx))*real(down(es.Ex))+real(down(es.Dy))*real(down(es.Ey))+real(down(es.Dz))*real(down(es.Ez)))+real(down(es.Dz))*(real(down(es.Ex))*es.unx+real(down(es.Ey))*es.uny+real(down(es.Ez))*es.unz)	Pa	Maxwell downward electric surface stress tensor, z component	Boundaries 2–4	
es.dnTex	0	Pa	Maxwell downward electric surface stress tensor, x component	Boundary 1	
es.dnTey	0	Pa	Maxwell downward electric surface stress tensor, y component	Boundary 1	
es.dnTez	0	Pa	Maxwell downward electric surface stress tensor, z component	Boundary 1	
es.intWe	es.int_We(es.d*es.dWe)	J	Total electric energy	Global	+ operation
es.zref	50[ohm]	Ω	Reference impedance	Global	

2.3.3 Charge Conservation 1

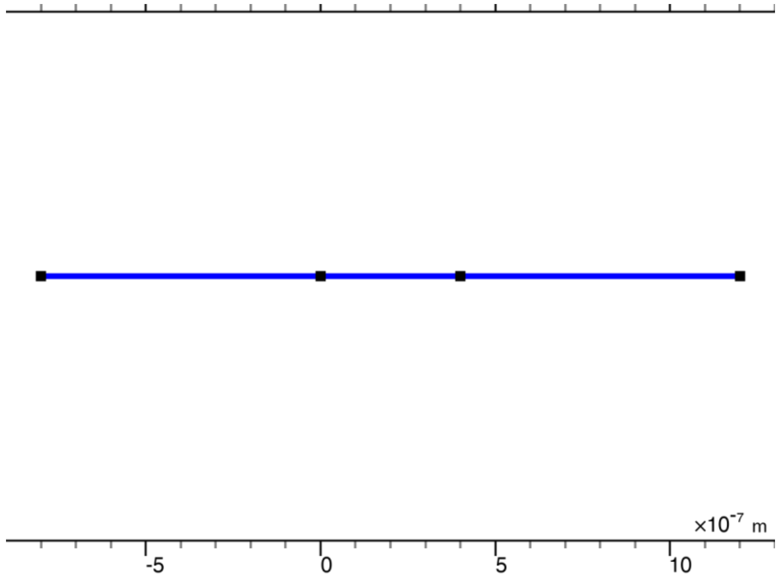

Charge Conservation 1

SELECTION

Geometric entity level	Domain
Selection	Geometry geom1: Dimension 1: All domains

EQUATIONS

$$\mathbf{E} = -\nabla V$$
$$\nabla \cdot (\epsilon_0 \epsilon_r \mathbf{E}) = \rho_v$$

Constitutive Relation D-E

SETTINGS

**Description**	**Value**
Dielectric model	Relative permittivity
Relative permittivity	User defined
Relative permittivity	{{eps_H2O, 0, 0}, {0, eps_H2O, 0}, {0, 0, eps_H2O}}

Coordinate System Selection

SETTINGS

**Description**	**Value**
Coordinate system	Global coordinate system

Model Input

SETTINGS

Description	Value
Temperature	Common model input

Variables

Name	Expression	Unit	Description	Selection	Details
es.nD	0	C/m²	Surface charge density	Boundaries 1–4	+ operation
es.epsilonrxx	eps_H2O	1	Relative permittivity, xx component	Domains 1–3	
es.epsilonryx	0	1	Relative permittivity, yx component	Domains 1–3	
es.epsilonrzx	0	1	Relative permittivity, zx component	Domains 1–3	
es.epsilonrxy	0	1	Relative permittivity, xy component	Domains 1–3	
es.epsilonryy	eps_H2O	1	Relative permittivity, yy component	Domains 1–3	
es.epsilonrzy	0	1	Relative permittivity, zy component	Domains 1–3	
es.epsilonrxz	0	1	Relative permittivity, xz component	Domains 1–3	
es.epsilonryz	0	1	Relative permittivity, yz component	Domains 1–3	
es.epsilonrzz	eps_H2O	1	Relative permittivity, zz component	Domains 1–3	
es.epsilonr_iso	eps_H2O	1	Relative permittivity, isotropic value	Domains 1–3	
es.DrX	0	C/m²	Remanent electric displacement, X component	Domains 1–3	
es.DrY	0	C/m²	Remanent electric displacement, Y component	Domains 1–3	

Name	Expression	Unit	Description	Selection	Details
es.DrZ	0	C/m²	Remanent electric displacement, Z component	Domains 1–3	
es.Dx	epsilon0_const*es.I_sxx*es.Ex+epsilon0_const*es.I_sxy*es.Ey+epsilon0_const*es.I_sxz*es.Ez+es.Px+es.Pex	C/m²	Electric displacement field, x component	Domains 1–3	
es.Dy	epsilon0_const*es.I_syx*es.Ex+epsilon0_const*es.I_syy*es.Ey+epsilon0_const*es.I_syz*es.Ez+es.Py+es.Pey	C/m²	Electric displacement field, y component	Domains 1–3	
es.Dz	epsilon0_const*es.I_szx*es.Ex+epsilon0_const*es.I_szy*es.Ey+epsilon0_const*es.I_szz*es.Ez+es.Pz+es.Pez	C/m²	Electric displacement field, z component	Domains 1–3	
es.Px	epsilon0_const*(es.chixx*es.Ex+es.chixy*es.Ey+es.chixz*es.Ez)	C/m²	Polarization, x component	Domains 1–3	
es.Py	epsilon0_const*(es.chiyx*es.Ex+es.chiyy*es.Ey+es.chiyz*es.Ez)	C/m²	Polarization, y component	Domains 1–3	
es.Pz	epsilon0_const*(es.chizx*es.Ex+es.chizy*es.Ey+es.chizz*es.Ez)	C/m²	Polarization, z component	Domains 1–3	
es.normD	sqrt(realdot(es.Dx,es.Dx)+realdot(es.Dy,es.Dy)+realdot(es.Dz,es.Dz))	C/m²	Electric displacement field norm	Domains 1–3	
es.normP	sqrt(realdot(es.Px,es.Px)+realdot(es.Py,es.Py)+realdot(es.Pz,es.Pz))	C/m²	Polarization norm	Domains 1–3	
es.Pex	0	C/m²	Polarization contribution, x component	Domains 1–3	+ operation
es.Pey	0	C/m²	Polarization contribution, y component	Domains 1–3	+ operation
es.Pez	0	C/m²	Polarization contribution, z component	Domains 1–3	+ operation
es.chixx	-1+es.epsilonrxx	1	Electric	Domains 1–3	

Name	Expression	Unit	Description	Selection	Details
			susceptibility, xx component		
es.chiyx	es.epsilonryx	1	Electric susceptibility, yx component	Domains 1–3	
es.chizx	es.epsilonrzx	1	Electric susceptibility, zx component	Domains 1–3	
es.chixy	es.epsilonrxy	1	Electric susceptibility, xy component	Domains 1–3	
es.chiyy	-1+es.epsilonryy	1	Electric susceptibility, yy component	Domains 1–3	
es.chizy	es.epsilonrzy	1	Electric susceptibility, zy component	Domains 1–3	
es.chixz	es.epsilonrxz	1	Electric susceptibility, xz component	Domains 1–3	
es.chiyz	es.epsilonryz	1	Electric susceptibility, yz component	Domains 1–3	
es.chizz	-1+es.epsilonrzz	1	Electric susceptibility, zz component	Domains 1–3	
es.Ex	-Vx	V/m	Electric field, x component	Domains 1–3	
es.Ey	0	V/m	Electric field, y component	Domains 1–3	
es.Ez	0	V/m	Electric field, z component	Domains 1–3	
es.tEx	-VTx	V/m	Tangential electric field, x component	Boundaries 1–4	
es.tEy	0	V/m	Tangential electric field, y component	Boundaries 1–4	
es.tEz	0	V/m	Tangential electric field, z component	Boundaries 1–4	
es.normE	sqrt(realdot(es.Ex,es.Ex)+realdot(es.Ey,es.Ey)+realdot(es.Ez,es.Ez))	V/m	Electric field norm	Domains 1–3	
es.Jx	es.Jdx	A/m^2	Current density, x	Domains 1–3	+ operation

Name	Expression	Unit	Description	Selection	Details
			component		
es.Jy	es.Jdy	A/m²	Current density, y component	Domains 1–3	+ operation
es.Jz	es.Jdz	A/m²	Current density, z component	Domains 1–3	+ operation
es.JX	spatial.invF11*es.Jdx*spatial.detF	A/m²	Current density, X component	Domains 1–3	+ operation
es.JY	es.Jdy*spatial.detF	A/m²	Current density, Y component	Domains 1–3	+ operation
es.JZ	es.Jdz*spatial.detF	A/m²	Current density, Z component	Domains 1–3	+ operation
es.Jdx	0	A/m²	Displacement current density, x component	Domains 1–3	
es.Jdy	0	A/m²	Displacement current density, y component	Domains 1–3	
es.Jdz	0	A/m²	Displacement current density, z component	Domains 1–3	
es.normJ	sqrt(realdot(es.Jx,es.Jx)+realdot(es.Jy,es.Jy)+realdot(es.Jz,es.Jz))	A/m²	Current density norm	Domains 1–3	
es.ccn1.nJ	es.dnx*up(es.Jx)+es.dny*up(es.Jy)+es.dnz*up(es.Jz)	A/m²	Inward current density	Boundary 1	
es.ccn1.nJ	es.unx*down(es.Jx)+es.uny*down(es.Jy)+es.unz*down(es.Jz)	A/m²	Inward current density	Boundary 4	
es.W	es.We	J/m³	Energy density	Domains 1–3	+ operation
es.dWe	es.We	J/m³	Integrand for total electric energy	Domains 1–3	Meta
es.We	0.5*epsilon0_const*(((es.I_sxx+es.chixx)*es.Ex+(es.I_sxy+es.chixy)*es.Ey+(es.I_sxz+es.chixz)*es.Ez)*es.Ex+((es.I_syx+es.chiyx)*es.Ex+(es.I_syy+es.chiyy)*es.Ey+(es.I_syz+es.chiyz)*es.Ez)*es.Ey+((es.I_szx+es.chizx)*es.Ex+(es.I_szy+es.chizy)*es.Ey+(es.I_sz	J/m³	Electric energy density	Domains 1–3	

Name	Expression	Unit	Description	Selection	Details
	z+es.chizz)*es.Ez)*es.E z)				

Shape functions

Name	Shape function	Unit	Description	Shape frame	Selection
V	Lagrange (Quadratic)	V	Electric potential	Spatial	Domains 1–3
V	Lagrange (Quadratic)	V	Electric potential	Material	Domains 1–3
V	Lagrange (Quadratic)	V	Electric potential	Geometry	Domains 1–3
V	Lagrange (Quadratic)	V	Electric potential	Mesh	Domains 1–3

2.3.4 Zero Charge 1

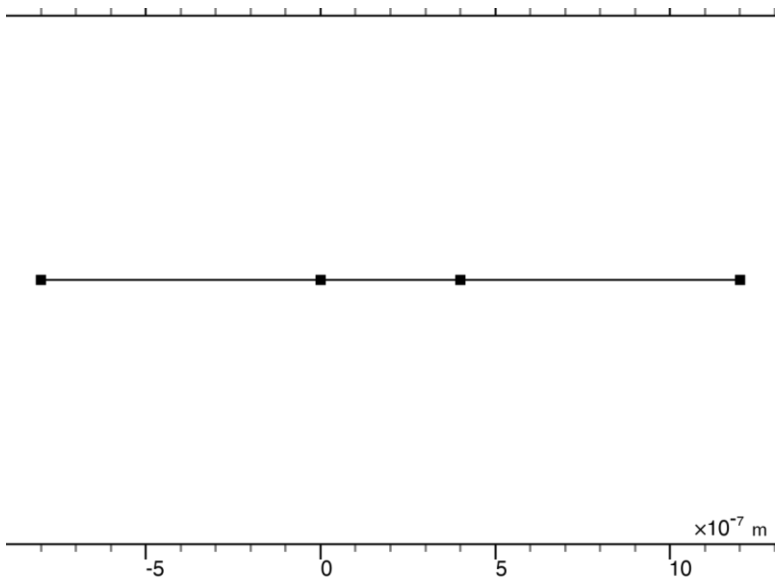

Zero Charge 1

SELECTION

Geometric entity level	Boundary
Selection	Geometry geom1: Dimension 0: All boundaries

EQUATIONS

$$\mathbf{n} \cdot \mathbf{D} = 0$$

Shape functions

Name	Shape function	Unit	Description	Shape frame	Selection	Details
V	Lagrange (Quadratic)	V	Electric potential	Spatial	No boundaries	Slit

Name	Shape function	Unit	Description	Shape frame	Selection	Details
V	Lagrange (Quadratic)	V	Electric potential	Material	No boundaries	Slit
V	Lagrange (Quadratic)	V	Electric potential	Geometry	No boundaries	Slit
V	Lagrange (Quadratic)	V	Electric potential	Mesh	No boundaries	Slit

2.3.5 Initial Values 1

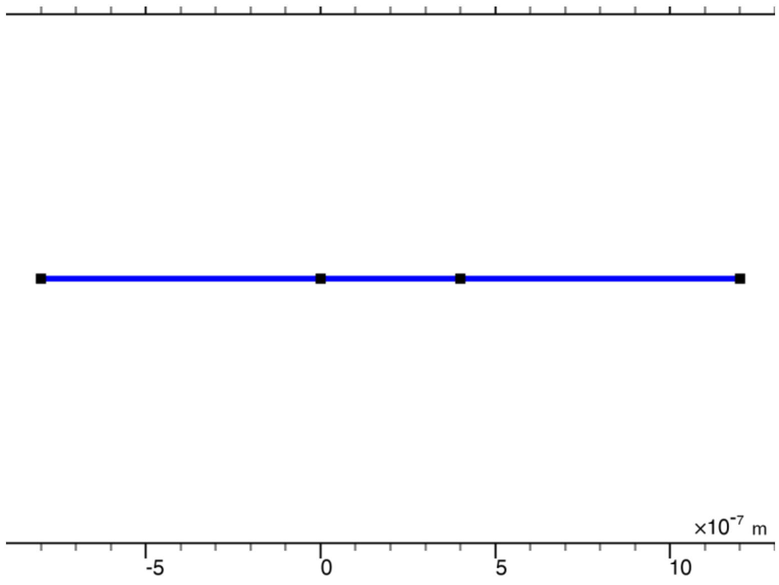

Initial Values 1

SELECTION

Geometric entity level	Domain
Selection	Geometry geom1: Dimension 1: All domains

SETTINGS

Description	Value
Electric potential	0

2.3.6 Surface Charge Density 1

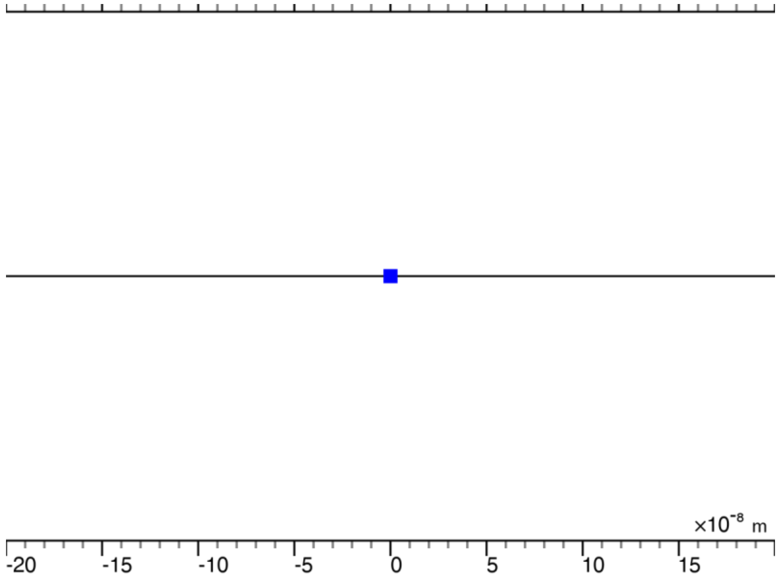

Surface Charge Density 1

SELECTION

Geometric entity level	Boundary
Selection	Geometry geom1: Dimension 0: Boundary 2

EQUATIONS

$$\mathbf{n}\cdot(\mathbf{D}_1 - \mathbf{D}_2) = \rho_s$$

Surface Charge Density

SETTINGS

Description	Value
Surface charge density	rho_surf_left

Coordinate System Selection

SETTINGS

Description	Value
Coordinate system	Global coordinate system

Variables

Name	Expression	Unit	Description	Selection	Details
es.nD	es.sfcd1.rhoqs	C/m^2	Surface charge density	Boundary 2	+ operation
es.sfcd1.rhoqs	rho_surf_left	C/m^2	Surface charge density	Boundary 2	

2.3.7 Surface Charge Density 2

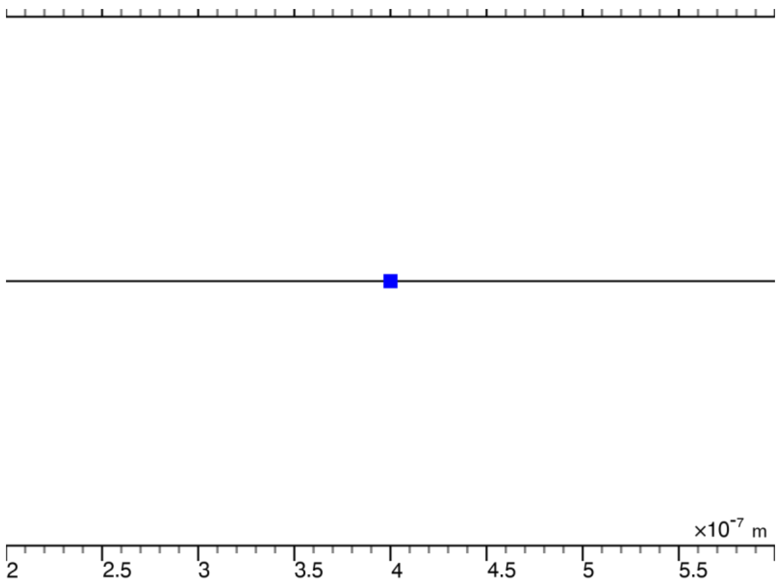

Surface Charge Density 2

SELECTION

Geometric entity level	Boundary
Selection	Geometry geom1: Dimension 0: Boundary 3

EQUATIONS

$$\mathbf{n}\cdot(\mathbf{D}_1 - \mathbf{D}_2) = \rho_s$$

Surface Charge Density

SETTINGS

Description	Value
Surface charge density	rho_surf_right

Coordinate System Selection

SETTINGS

Description	Value
Coordinate system	Global coordinate system

Variables

Name	Expression	Unit	Description	Selection	Details
es.nD	es.sfcd2.rhoqs	C/m²	Surface charge density	Boundary 3	+ operation
es.sfcd2.rhoqs	rho_surf_right	C/m²	Surface charge density	Boundary 3	

2.3.8 Ground 1

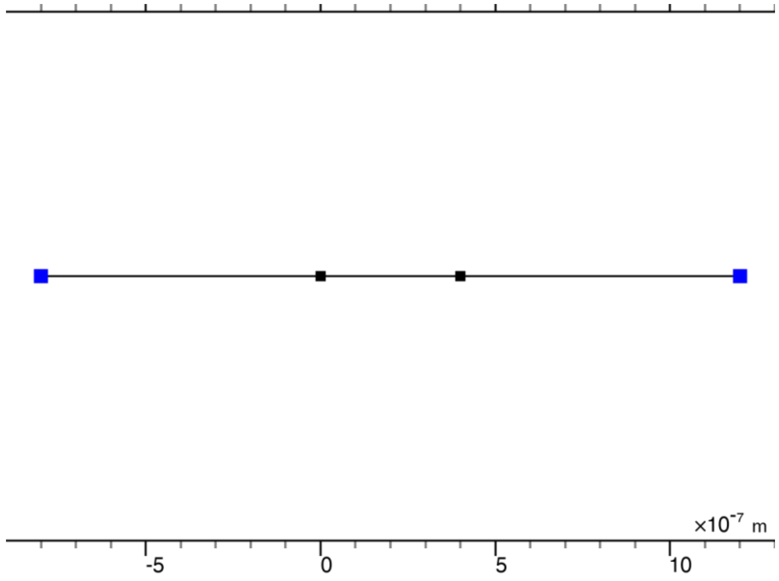

Ground 1

SELECTION

Geometric entity level	Boundary
Selection	Geometry geom1: Dimension 0: Boundaries 1, 4

EQUATIONS

$$V = 0$$

Constraint Settings

SETTINGS

Description	Value
Apply reaction terms on	All physics (symmetric)
Use weak constraints	Off
Constraint method	Elemental

Variables

Name	Expression	Unit	Description	Selection	Details
es.nD	-es.unx*up(es.Dx)-es.uny*up(es.Dy)-es.unz*up(es.Dz)	C/m^2	Surface charge density	Boundary 1	+ operation
es.nD	es.unx*down(es.Dx)+es.uny*down(es.Dy)+es.unz*down(es.Dz)	C/m^2	Surface charge density	Boundary 4	+ operation

2.4 TRANSPORT OF DILUTED SPECIES

USED PRODUCTS

COMSOL Multiphysics
Chemical Reaction Engineering Module

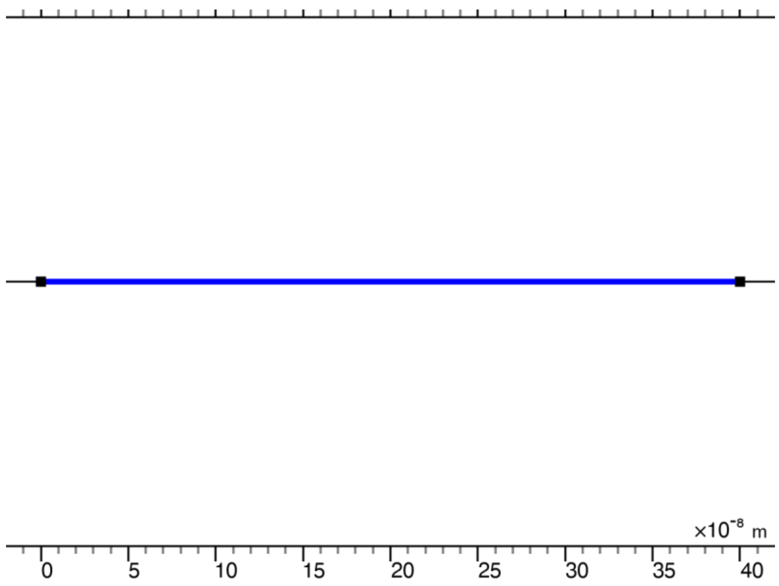

Transport of Diluted Species

SELECTION

Geometric entity level	Domain
Selection	Geometry geom1: Dimension 1: Domain 2

EQUATIONS

$$\frac{\partial c_i}{\partial t} + \nabla \cdot \mathbf{J}_i = R_i$$
$$\mathbf{J}_i = -D_i \nabla c_i - z_i u_{m,i} F c_i \nabla V$$

2.4.1 Interface Settings

Discretization

SETTINGS

Description	Value
Concentration	Linear

SETTINGS

Description	Value
Equation form	Study controlled

Cross-Sectional Area

SETTINGS

Description	Value
Cross-sectional area	1[m^2]

Consistent Stabilization

SETTINGS

Description	Value
Streamline diffusion	On
Crosswind diffusion	On
Equation residual	Approximate residual
Crosswind diffusion type	Do Carmo and Galeão

Inconsistent Stabilization

SETTINGS

Description	Value
Isotropic diffusion	Off

Advanced Settings

SETTINGS

Description	Value
Convective term	Nonconservative form

Transport Mechanisms

SETTINGS

Description	Value
Convection	Off
Migration in electric field	On
Mass transfer in porous media	Off

2.4.2 Variables

Name	Expression	Unit	Description	Selection	Details
tds.Ac	1[m^2]	m^2	Cross-sectional area	Global	
tds.d	tds.Ac	m^2	Out-of-plane geometry extension	Global	
tds.nx	unx	1	Normal vector, x component	Boundary 2	
tds.ny	0	1	Normal vector, y	Boundary 2	

Name	Expression	Unit	Description	Selection	Details
			component		
tds.nz	0	1	Normal vector, z component	Boundary 2	
tds.nx	dnx	1	Normal vector, x component	Boundary 3	
tds.ny	0	1	Normal vector, y component	Boundary 3	
tds.nz	0	1	Normal vector, z component	Boundary 3	
tds.nxmesh	unxmesh	1	Normal vector (mesh), x component	Boundary 2	
tds.nymesh	0	1	Normal vector (mesh), y component	Boundary 2	
tds.nzmesh	0	1	Normal vector (mesh), z component	Boundary 2	
tds.nxmesh	dnxmesh	1	Normal vector (mesh), x component	Boundary 3	
tds.nymesh	0	1	Normal vector (mesh), y component	Boundary 3	
tds.nzmesh	0	1	Normal vector (mesh), z component	Boundary 3	
tds.nxc	-root.nxc/tds.ncLen	1	Normal vector, x component	Boundary 2	
tds.nyc	0	1	Normal vector, y component	Boundary 2	
tds.nzc	0	1	Normal vector, z component	Boundary 2	
tds.nxc	root.nxc/tds.ncLen	1	Normal vector, x component	Boundary 3	
tds.nyc	0	1	Normal vector, y component	Boundary 3	
tds.nzc	0	1	Normal vector, z component	Boundary 3	
tds.R_cAsc	0	mol/(m³·s)	Total rate expression	Domain 2	+ operation
tds.cP_cAsc	0	mol/kg	Concentration species adsorbed to the solid	Domain 2	+ operation
tds.cP_cAsc	0	mol/kg	Concentration species adsorbed to the solid	Boundaries 2–3	+ operation
tds.KP_cAsc	0	m³/kg	Adsorption isotherm,	Domain 2	+ operation

Name	Expression	Unit	Description	Selection	Details
			first concentration derivative		
tds.KP_cAsc	0	m^3/kg	Adsorption isotherm, first concentration derivative	Boundaries 2–3	+ operation
tds.Rads_cAsc	0	$mol/(m^3 \cdot s)$	Total adsorption rate	Domain 2	+ operation
tds.DiT_cAsc	0	m^2/s	Turbulent diffusivity	Domain 2	
tds.cVar_cAsc	cAsc	mol/m^3	Species	Boundaries 2–3	
tds.R_cAscm	0	$mol/(m^3 \cdot s)$	Total rate expression	Domain 2	+ operation
tds.cP_cAscm	0	mol/kg	Concentration species adsorbed to the solid	Domain 2	+ operation
tds.cP_cAscm	0	mol/kg	Concentration species adsorbed to the solid	Boundaries 2–3	+ operation
tds.KP_cAscm	0	m^3/kg	Adsorption isotherm, first concentration derivative	Domain 2	+ operation
tds.KP_cAscm	0	m^3/kg	Adsorption isotherm, first concentration derivative	Boundaries 2–3	+ operation
tds.Rads_cAscm	0	$mol/(m^3 \cdot s)$	Total adsorption rate	Domain 2	+ operation
tds.DiT_cAscm	0	m^2/s	Turbulent diffusivity	Domain 2	
tds.cVar_cAscm	cAscm	mol/m^3	Species	Boundaries 2–3	
tds.R_cCO2	0	$mol/(m^3 \cdot s)$	Total rate expression	Domain 2	+ operation
tds.cP_cCO2	0	mol/kg	Concentration species adsorbed to the solid	Domain 2	+ operation
tds.cP_cCO2	0	mol/kg	Concentration species adsorbed to the solid	Boundaries 2–3	+ operation
tds.KP_cCO2	0	m^3/kg	Adsorption isotherm, first concentration derivative	Domain 2	+ operation
tds.KP_cCO2	0	m^3/kg	Adsorption isotherm, first concentration derivative	Boundaries 2–3	+ operation
tds.Rads_cCO2	0	$mol/(m^3 \cdot s)$	Total adsorption rate	Domain 2	+ operation
tds.DiT_cCO2	0	m^2/s	Turbulent diffusivity	Domain 2	
tds.cVar_cCO2	cCO2	mol/m^3	Species	Boundaries 2–3	

Name	Expression	Unit	Description	Selection	Details
tds.R_cCO	0	mol/(m³·s)	Total rate expression	Domain 2	+ operation
tds.cP_cCO	0	mol/kg	Concentration species adsorbed to the solid	Domain 2	+ operation
tds.cP_cCO	0	mol/kg	Concentration species adsorbed to the solid	Boundaries 2–3	+ operation
tds.KP_cCO	0	m³/kg	Adsorption isotherm, first concentration derivative	Domain 2	+ operation
tds.KP_cCO	0	m³/kg	Adsorption isotherm, first concentration derivative	Boundaries 2–3	+ operation
tds.Rads_cCO	0	mol/(m³·s)	Total adsorption rate	Domain 2	+ operation
tds.DiT_cCO	0	m²/s	Turbulent diffusivity	Domain 2	
tds.cVar_cCO	cCO	mol/m³	Species	Boundaries 2–3	
tds.R_cO2	0	mol/(m³·s)	Total rate expression	Domain 2	+ operation
tds.cP_cO2	0	mol/kg	Concentration species adsorbed to the solid	Domain 2	+ operation
tds.cP_cO2	0	mol/kg	Concentration species adsorbed to the solid	Boundaries 2–3	+ operation
tds.KP_cO2	0	m³/kg	Adsorption isotherm, first concentration derivative	Domain 2	+ operation
tds.KP_cO2	0	m³/kg	Adsorption isotherm, first concentration derivative	Boundaries 2–3	+ operation
tds.Rads_cO2	0	mol/(m³·s)	Total adsorption rate	Domain 2	+ operation
tds.DiT_cO2	0	m²/s	Turbulent diffusivity	Domain 2	
tds.cVar_cO2	cO2	mol/m³	Species	Boundaries 2–3	
tds.R_cH2O	0	mol/(m³·s)	Total rate expression	Domain 2	+ operation
tds.cP_cH2O	0	mol/kg	Concentration species adsorbed to the solid	Domain 2	+ operation
tds.cP_cH2O	0	mol/kg	Concentration species adsorbed to the solid	Boundaries 2–3	+ operation
tds.KP_cH2O	0	m³/kg	Adsorption isotherm, first concentration	Domain 2	+ operation

Name	Expression	Unit	Description	Selection	Details
			derivative		
tds.KP_cH2O	0	m^3/kg	Adsorption isotherm, first concentration derivative	Boundaries 2–3	+ operation
tds.Rads_cH2O	0	$mol/(m^3·s)$	Total adsorption rate	Domain 2	+ operation
tds.DiT_cH2O	0	m^2/s	Turbulent diffusivity	Domain 2	
tds.cVar_cH2O	cH2O	mol/m^3	Species	Boundaries 2–3	
tds.R_cH2PO4	0	$mol/(m^3·s)$	Total rate expression	Domain 2	+ operation
tds.cP_cH2PO4	0	mol/kg	Concentration species adsorbed to the solid	Domain 2	+ operation
tds.cP_cH2PO4	0	mol/kg	Concentration species adsorbed to the solid	Boundaries 2–3	+ operation
tds.KP_cH2PO4	0	m^3/kg	Adsorption isotherm, first concentration derivative	Domain 2	+ operation
tds.KP_cH2PO4	0	m^3/kg	Adsorption isotherm, first concentration derivative	Boundaries 2–3	+ operation
tds.Rads_cH2PO4	0	$mol/(m^3·s)$	Total adsorption rate	Domain 2	+ operation
tds.DiT_cH2PO4	0	m^2/s	Turbulent diffusivity	Domain 2	
tds.cVar_cH2PO4	cH2PO4	mol/m^3	Species	Boundaries 2–3	
tds.R_cHPO4	0	$mol/(m^3·s)$	Total rate expression	Domain 2	+ operation
tds.cP_cHPO4	0	mol/kg	Concentration species adsorbed to the solid	Domain 2	+ operation
tds.cP_cHPO4	0	mol/kg	Concentration species adsorbed to the solid	Boundaries 2–3	+ operation
tds.KP_cHPO4	0	m^3/kg	Adsorption isotherm, first concentration derivative	Domain 2	+ operation
tds.KP_cHPO4	0	m^3/kg	Adsorption isotherm, first concentration derivative	Boundaries 2–3	+ operation
tds.Rads_cHPO4	0	$mol/(m^3·s)$	Total adsorption rate	Domain 2	+ operation

Name	Expression	Unit	Description	Selection	Details
tds.DiT_cHPO4	0	m^2/s	Turbulent diffusivity	Domain 2	
tds.cVar_cHPO 4	cHPO4	mol/m^3	Species	Boundaries 2–3	
tds.R_cNa	0	$mol/(m^3 \cdot s)$	Total rate expression	Domain 2	+ operation
tds.cP_cNa	0	mol/kg	Concentration species adsorbed to the solid	Domain 2	+ operation
tds.cP_cNa	0	mol/kg	Concentration species adsorbed to the solid	Boundaries 2–3	+ operation
tds.KP_cNa	0	m^3/kg	Adsorption isotherm, first concentration derivative	Domain 2	+ operation
tds.KP_cNa	0	m^3/kg	Adsorption isotherm, first concentration derivative	Boundaries 2–3	+ operation
tds.Rads_cNa	0	$mol/(m^3 \cdot s)$	Total adsorption rate	Domain 2	+ operation
tds.DiT_cNa	0	m^2/s	Turbulent diffusivity	Domain 2	
tds.cVar_cNa	cNa	mol/m^3	Species	Boundaries 2–3	
tds.R_cCl	0	$mol/(m^3 \cdot s)$	Total rate expression	Domain 2	+ operation
tds.cP_cCl	0	mol/kg	Concentration species adsorbed to the solid	Domain 2	+ operation
tds.cP_cCl	0	mol/kg	Concentration species adsorbed to the solid	Boundaries 2–3	+ operation
tds.KP_cCl	0	m^3/kg	Adsorption isotherm, first concentration derivative	Domain 2	+ operation
tds.KP_cCl	0	m^3/kg	Adsorption isotherm, first concentration derivative	Boundaries 2–3	+ operation
tds.Rads_cCl	0	$mol/(m^3 \cdot s)$	Total adsorption rate	Domain 2	+ operation
tds.DiT_cCl	0	m^2/s	Turbulent diffusivity	Domain 2	
tds.cVar_cCl	cCl	mol/m^3	Species	Boundaries 2–3	
tds.R_cH3PO4	0	$mol/(m^3 \cdot s)$	Total rate expression	Domain 2	+ operation
tds.cP_cH3PO4	0	mol/kg	Concentration species adsorbed to the solid	Domain 2	+ operation
tds.cP_cH3PO4	0	mol/kg	Concentration species adsorbed to	Boundaries 2–3	+ operation

Name	Expression	Unit	Description	Selection	Details
			the solid		
tds.KP_cH3PO4	0	m^3/kg	Adsorption isotherm, first concentration derivative	Domain 2	+ operation
tds.KP_cH3PO4	0	m^3/kg	Adsorption isotherm, first concentration derivative	Boundaries 2–3	+ operation
tds.Rads_cH3PO4	0	$mol/(m^3·s)$	Total adsorption rate	Domain 2	+ operation
tds.DiT_cH3PO4	0	m^2/s	Turbulent diffusivity	Domain 2	
tds.cVar_cH3PO4	cH3PO4	mol/m^3	Species	Boundaries 2–3	
tds.R_cPO4	0	$mol/(m^3·s)$	Total rate expression	Domain 2	+ operation
tds.cP_cPO4	0	mol/kg	Concentration species adsorbed to the solid	Domain 2	+ operation
tds.cP_cPO4	0	mol/kg	Concentration species adsorbed to the solid	Boundaries 2–3	+ operation
tds.KP_cPO4	0	m^3/kg	Adsorption isotherm, first concentration derivative	Domain 2	+ operation
tds.KP_cPO4	0	m^3/kg	Adsorption isotherm, first concentration derivative	Boundaries 2–3	+ operation
tds.Rads_cPO4	0	$mol/(m^3·s)$	Total adsorption rate	Domain 2	+ operation
tds.DiT_cPO4	0	m^2/s	Turbulent diffusivity	Domain 2	
tds.cVar_cPO4	cPO4	mol/m^3	Species	Boundaries 2–3	
tds.R_cH	0	$mol/(m^3·s)$	Total rate expression	Domain 2	+ operation
tds.cP_cH	0	mol/kg	Concentration species adsorbed to the solid	Domain 2	+ operation
tds.cP_cH	0	mol/kg	Concentration species adsorbed to the solid	Boundaries 2–3	+ operation
tds.KP_cH	0	m^3/kg	Adsorption isotherm, first concentration derivative	Domain 2	+ operation
tds.KP_cH	0	m^3/kg	Adsorption isotherm, first concentration	Boundaries 2–3	+ operation

Name	Expression	Unit	Description	Selection	Details
			derivative		
tds.Rads_cH	0	$mol/(m^3 \cdot s)$	Total adsorption rate	Domain 2	+ operation
tds.DiT_cH	0	m^2/s	Turbulent diffusivity	Domain 2	
tds.cVar_cH	cH	mol/m^3	Species	Boundaries 2–3	
tds.R_cOH	0	$mol/(m^3 \cdot s)$	Total rate expression	Domain 2	+ operation
tds.cP_cOH	0	mol/kg	Concentration species adsorbed to the solid	Domain 2	+ operation
tds.cP_cOH	0	mol/kg	Concentration species adsorbed to the solid	Boundaries 2–3	+ operation
tds.KP_cOH	0	m^3/kg	Adsorption isotherm, first concentration derivative	Domain 2	+ operation
tds.KP_cOH	0	m^3/kg	Adsorption isotherm, first concentration derivative	Boundaries 2–3	+ operation
tds.Rads_cOH	0	$mol/(m^3 \cdot s)$	Total adsorption rate	Domain 2	+ operation
tds.DiT_cOH	0	m^2/s	Turbulent diffusivity	Domain 2	
tds.cVar_cOH	cOH	mol/m^3	Species	Boundaries 2–3	
tds.R_cHCO3	0	$mol/(m^3 \cdot s)$	Total rate expression	Domain 2	+ operation
tds.cP_cHCO3	0	mol/kg	Concentration species adsorbed to the solid	Domain 2	+ operation
tds.cP_cHCO3	0	mol/kg	Concentration species adsorbed to the solid	Boundaries 2–3	+ operation
tds.KP_cHCO3	0	m^3/kg	Adsorption isotherm, first concentration derivative	Domain 2	+ operation
tds.KP_cHCO3	0	m^3/kg	Adsorption isotherm, first concentration derivative	Boundaries 2–3	+ operation
tds.Rads_cHCO3	0	$mol/(m^3 \cdot s)$	Total adsorption rate	Domain 2	+ operation
tds.DiT_cHCO3	0	m^2/s	Turbulent diffusivity	Domain 2	
tds.cVar_cHCO3	cHCO3	mol/m^3	Species	Boundaries 2–3	
tds.R_cCO3	0	$mol/(m^3 \cdot s)$	Total rate expression	Domain 2	+ operation
tds.cP_cCO3	0	mol/kg	Concentration	Domain 2	+ operation

Name	Expression	Unit	Description	Selection	Details
			species adsorbed to the solid		
tds.cP_cCO3	0	mol/kg	Concentration species adsorbed to the solid	Boundaries 2–3	+ operation
tds.KP_cCO3	0	m^3/kg	Adsorption isotherm, first concentration derivative	Domain 2	+ operation
tds.KP_cCO3	0	m^3/kg	Adsorption isotherm, first concentration derivative	Boundaries 2–3	+ operation
tds.Rads_cCO3	0	mol/(m^3·s)	Total adsorption rate	Domain 2	+ operation
tds.DiT_cCO3	0	m^2/s	Turbulent diffusivity	Domain 2	
tds.cVar_cCO3	cCO3	mol/m^3	Species	Boundaries 2–3	
tds.poro	1	1	Porosity	Domain 2	
tds.theta_g	0	1	Gas volume fraction	Domain 2	
tds.theta_l	1	1	Liquid volume fraction	Domain 2	
tds.theta	tds.poro	1	Mobile fluid volume fraction	Domain 2	

2.4.3 Transport Properties 1

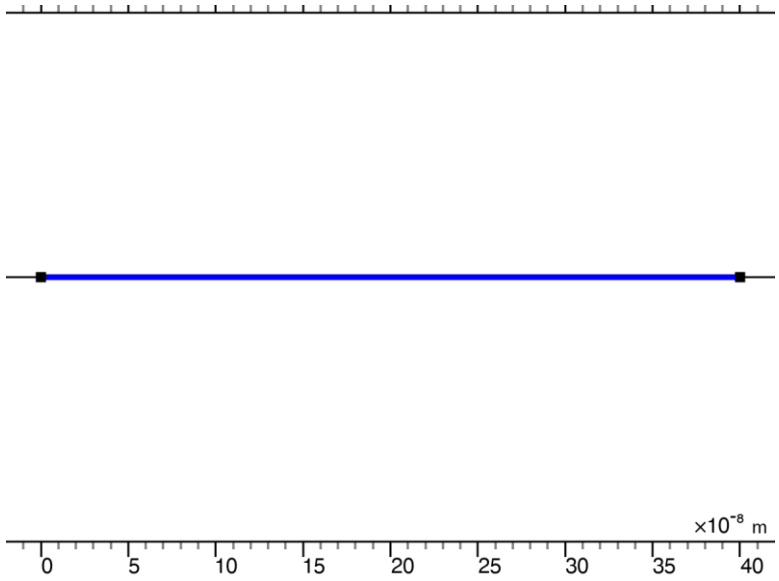

Transport Properties 1

SELECTION

Geometric entity level	Domain

Selection	Geometry geom1: Dimension 1: All domains

EQUATIONS

$$\frac{\partial c_i}{\partial t} + \nabla \cdot \mathbf{J}_i = R_i$$

$$\mathbf{J}_i = -D_i \nabla c_i - z_i u_{m,i} F c_i \nabla V$$

Diffusion

SETTINGS

Description	Value
Source	Material
Material	None
Diffusion coefficient	User defined
Diffusion coefficient	{{1e-9[m^2/s], 0, 0}, {0, 1e-9[m^2/s], 0}, {0, 0, 1e-9[m^2/s]}}
Diffusion coefficient	User defined
Diffusion coefficient	{{1e-9[m^2/s], 0, 0}, {0, 1e-9[m^2/s], 0}, {0, 0, 1e-9[m^2/s]}}
Diffusion coefficient	User defined
Diffusion coefficient	{{D_CO2w, 0, 0}, {0, D_CO2w, 0}, {0, 0, D_CO2w}}
Diffusion coefficient	User defined
Diffusion coefficient	{{D_COw, 0, 0}, {0, D_COw, 0}, {0, 0, D_COw}}
Diffusion coefficient	User defined
Diffusion coefficient	{{D_O2w, 0, 0}, {0, D_O2w, 0}, {0, 0, D_O2w}}
Diffusion coefficient	User defined
Diffusion coefficient	{{1e-9, 0, 0}, {0, 1e-9, 0}, {0, 0, 1e-9}}
Diffusion coefficient	User defined
Diffusion coefficient	{{D_H2PO4w, 0, 0}, {0, D_H2PO4w, 0}, {0, 0, D_H2PO4w}}
Diffusion coefficient	User defined
Diffusion coefficient	{{D_HPO4w, 0, 0}, {0, D_HPO4w, 0}, {0, 0, D_HPO4w}}
Diffusion coefficient	User defined
Diffusion coefficient	{{D_Naw, 0, 0}, {0, D_Naw, 0}, {0, 0, D_Naw}}
Diffusion coefficient	User defined
Diffusion coefficient	{{D_Clw, 0, 0}, {0, D_Clw, 0}, {0, 0, D_Clw}}
Diffusion coefficient	User defined
Diffusion coefficient	{{D_H3PO4w, 0, 0}, {0, D_H3PO4w, 0}, {0, 0, D_H3PO4w}}
Diffusion coefficient	User defined
Diffusion coefficient	{{D_PO4w, 0, 0}, {0, D_PO4w, 0}, {0, 0, D_PO4w}}
Diffusion coefficient	User defined
Diffusion coefficient	{{D_Hw, 0, 0}, {0, D_Hw, 0}, {0, 0, D_Hw}}

Description	Value
Diffusion coefficient	User defined
Diffusion coefficient	{{D_OHw, 0, 0}, {0, D_OHw, 0}, {0, 0, D_OHw}}
Diffusion coefficient	User defined
Diffusion coefficient	{{D_HCO3w, 0, 0}, {0, D_HCO3w, 0}, {0, 0, D_HCO3w}}
Diffusion coefficient	User defined
Diffusion coefficient	{{D_CO3w, 0, 0}, {0, D_CO3w, 0}, {0, 0, D_CO3w}}

Migration in Electric Field

SETTINGS

Description	Value
Mobility	Nernst - Einstein relation
Charge number	{0, -1, 0, 0, 0, 0, -1, -2, 1, -1, 0, -3, 1, -1, -1, -2}

Coordinate System Selection

SETTINGS

Description	Value
Coordinate system	Global coordinate system

Model Input

SETTINGS

Description	Value
Temperature	Common model input

Variables

Name	Expression	Unit	Description	Selection	Details
domflux.cAscx	(tds.dflux_cAscx+tds.mflux_cAscx)*tds.d	mol/s	Domain flux, x component	Domain 2	
domflux.cAscmx	(tds.dflux_cAscmx+tds.mflux_cAscmx)*tds.d	mol/s	Domain flux, x component	Domain 2	
domflux.cCO2x	(tds.dflux_cCO2x+tds.mflux_cCO2x)*tds.d	mol/s	Domain flux, x component	Domain 2	
domflux.cCOx	(tds.dflux_cCOx+tds.mflux_cCOx)*tds.d	mol/s	Domain flux, x component	Domain 2	
domflux.cO2x	(tds.dflux_cO2x+tds.mflux_cO2x)*tds.d	mol/s	Domain flux, x component	Domain 2	
domflux.cH2Ox	(tds.dflux_cH2Ox+tds.mflux_cH2Ox)*tds.	mol/s	Domain flux, x component	Domain 2	

Name	Expression	Unit	Description	Selection	Details
	d				
domflux.cH2PO4x	(tds.dflux_cH2PO4x+tds.mflux_cH2PO4x)*tds.d	mol/s	Domain flux, x component	Domain 2	
domflux.cHPO4x	(tds.dflux_cHPO4x+tds.mflux_cHPO4x)*tds.d	mol/s	Domain flux, x component	Domain 2	
domflux.cNax	(tds.dflux_cNax+tds.mflux_cNax)*tds.d	mol/s	Domain flux, x component	Domain 2	
domflux.cClx	(tds.dflux_cClx+tds.mflux_cClx)*tds.d	mol/s	Domain flux, x component	Domain 2	
domflux.cH3PO4x	(tds.dflux_cH3PO4x+tds.mflux_cH3PO4x)*tds.d	mol/s	Domain flux, x component	Domain 2	
domflux.cPO4x	(tds.dflux_cPO4x+tds.mflux_cPO4x)*tds.d	mol/s	Domain flux, x component	Domain 2	
domflux.cHx	(tds.dflux_cHx+tds.mflux_cHx)*tds.d	mol/s	Domain flux, x component	Domain 2	
domflux.cOHx	(tds.dflux_cOHx+tds.mflux_cOHx)*tds.d	mol/s	Domain flux, x component	Domain 2	
domflux.cHCO3x	(tds.dflux_cHCO3x+tds.mflux_cHCO3x)*tds.d	mol/s	Domain flux, x component	Domain 2	
domflux.cCO3x	(tds.dflux_cCO3x+tds.mflux_cCO3x)*tds.d	mol/s	Domain flux, x component	Domain 2	
tds.ndflux_cAsc	tds.dflux_cAscx*tds.nx+tds.dflux_cAscy*tds.ny+tds.dflux_cAscz*tds.nz	mol/(m²·s)	Normal diffusive flux	Boundaries 2–3	
tds.nmflux_cAsc	tds.mflux_cAscx*tds.nx+tds.mflux_cAscy*tds.ny+tds.mflux_cAscz*tds.nz	mol/(m²·s)	Normal electrophoretic flux	Boundaries 2–3	
tds.ntflux_cAsc	tds.bndFlux_cAsc	mol/(m²·s)	Normal total flux	Boundaries 2–3	
tds.ndflux_cAscm	tds.dflux_cAscmx*tds.nx+tds.dflux_cAscmy*tds.ny+tds.dflux_cAscmz*tds.nz	mol/(m²·s)	Normal diffusive flux	Boundaries 2–3	
tds.nmflux_cAscm	tds.mflux_cAscmx*tds.nx+tds.mflux_cAscmy*tds.ny+tds.mflux	mol/(m²·s)	Normal electrophoretic flux	Boundaries 2–3	

Name	Expression	Unit	Description	Selection	Details
	_cAscmz*tds.nz				
tds.ntflux_cAscm	tds.bndFlux_cAscm	mol/(m²·s)	Normal total flux	Boundaries 2–3	
tds.ndflux_cCO2	tds.dflux_cCO2x*tds.nx+tds.dflux_cCO2y*tds.ny+tds.dflux_cCO2z*tds.nz	mol/(m²·s)	Normal diffusive flux	Boundaries 2–3	
tds.nmflux_cCO2	tds.mflux_cCO2x*tds.nx+tds.mflux_cCO2y*tds.ny+tds.mflux_cCO2z*tds.nz	mol/(m²·s)	Normal electrophoretic flux	Boundaries 2–3	
tds.ntflux_cCO2	tds.bndFlux_cCO2	mol/(m²·s)	Normal total flux	Boundaries 2–3	
tds.ndflux_cCO	tds.dflux_cCOx*tds.nx+tds.dflux_cCOy*tds.ny+tds.dflux_cCOz*tds.nz	mol/(m²·s)	Normal diffusive flux	Boundaries 2–3	
tds.nmflux_cCO	tds.mflux_cCOx*tds.nx+tds.mflux_cCOy*tds.ny+tds.mflux_cCOz*tds.nz	mol/(m²·s)	Normal electrophoretic flux	Boundaries 2–3	
tds.ntflux_cCO	tds.bndFlux_cCO	mol/(m²·s)	Normal total flux	Boundaries 2–3	
tds.ndflux_cO2	tds.dflux_cO2x*tds.nx+tds.dflux_cO2y*tds.ny+tds.dflux_cO2z*tds.nz	mol/(m²·s)	Normal diffusive flux	Boundaries 2–3	
tds.nmflux_cO2	tds.mflux_cO2x*tds.nx+tds.mflux_cO2y*tds.ny+tds.mflux_cO2z*tds.nz	mol/(m²·s)	Normal electrophoretic flux	Boundaries 2–3	
tds.ntflux_cO2	tds.bndFlux_cO2	mol/(m²·s)	Normal total flux	Boundaries 2–3	
tds.ndflux_cH2O	tds.dflux_cH2Ox*tds.nx+tds.dflux_cH2Oy*tds.ny+tds.dflux_cH2Oz*tds.nz	mol/(m²·s)	Normal diffusive flux	Boundaries 2–3	
tds.nmflux_cH2O	tds.mflux_cH2Ox*tds.nx+tds.mflux_cH2Oy*tds.ny+tds.mflux_cH2Oz*tds.nz	mol/(m²·s)	Normal electrophoretic flux	Boundaries 2–3	
tds.ntflux_cH2O	tds.bndFlux_cH2O	mol/(m²·s)	Normal total flux	Boundaries 2–3	
tds.ndflux_cH2PO4	tds.dflux_cH2PO4x*tds.nx+tds.dflux_cH2PO4y*tds.ny+tds.dflux_cH2PO4z*tds.nz	mol/(m²·s)	Normal diffusive flux	Boundaries 2–3	

Name	Expression	Unit	Description	Selection	Details
tds.nmflux_cH2PO4	tds.mflux_cH2PO4x*tds.nx+tds.mflux_cH2PO4y*tds.ny+tds.mflux_cH2PO4z*tds.nz	mol/(m²·s)	Normal electrophoretic flux	Boundaries 2–3	
tds.ntflux_cH2PO4	tds.bndFlux_cH2PO4	mol/(m²·s)	Normal total flux	Boundaries 2–3	
tds.ndflux_cHPO4	tds.dflux_cHPO4x*tds.nx+tds.dflux_cHPO4y*tds.ny+tds.dflux_cHPO4z*tds.nz	mol/(m²·s)	Normal diffusive flux	Boundaries 2–3	
tds.nmflux_cHPO4	tds.mflux_cHPO4x*tds.nx+tds.mflux_cHPO4y*tds.ny+tds.mflux_cHPO4z*tds.nz	mol/(m²·s)	Normal electrophoretic flux	Boundaries 2–3	
tds.ntflux_cHPO4	tds.bndFlux_cHPO4	mol/(m²·s)	Normal total flux	Boundaries 2–3	
tds.ndflux_cNa	tds.dflux_cNax*tds.nx+tds.dflux_cNay*tds.ny+tds.dflux_cNaz*tds.nz	mol/(m²·s)	Normal diffusive flux	Boundaries 2–3	
tds.nmflux_cNa	tds.mflux_cNax*tds.nx+tds.mflux_cNay*tds.ny+tds.mflux_cNaz*tds.nz	mol/(m²·s)	Normal electrophoretic flux	Boundaries 2–3	
tds.ntflux_cNa	tds.bndFlux_cNa	mol/(m²·s)	Normal total flux	Boundaries 2–3	
tds.ndflux_cCl	tds.dflux_cClx*tds.nx+tds.dflux_cCly*tds.ny+tds.dflux_cClz*tds.nz	mol/(m²·s)	Normal diffusive flux	Boundaries 2–3	
tds.nmflux_cCl	tds.mflux_cClx*tds.nx+tds.mflux_cCly*tds.ny+tds.mflux_cClz*tds.nz	mol/(m²·s)	Normal electrophoretic flux	Boundaries 2–3	
tds.ntflux_cCl	tds.bndFlux_cCl	mol/(m²·s)	Normal total flux	Boundaries 2–3	
tds.ndflux_cH3PO4	tds.dflux_cH3PO4x*tds.nx+tds.dflux_cH3PO4y*tds.ny+tds.dflux_cH3PO4z*tds.nz	mol/(m²·s)	Normal diffusive flux	Boundaries 2–3	
tds.nmflux_cH3PO4	tds.mflux_cH3PO4x*tds.nx+tds.mflux_cH3PO4y*tds.ny+tds.mflux_cH3PO4z*tds.nz	mol/(m²·s)	Normal electrophoretic flux	Boundaries 2–3	
tds.ntflux_cH3PO4	tds.bndFlux_cH3PO4	mol/(m²·s)	Normal total flux	Boundaries 2–3	
tds.ndflux_cPO4	tds.dflux_cPO4x*tds.nx+tds.dflux_cPO4y*	mol/(m²·s)	Normal diffusive flux	Boundaries 2–3	

Name	Expression	Unit	Description	Selection	Details
	tds.ny+tds.dflux_cPO4z*tds.nz				
tds.nmflux_cPO4	tds.mflux_cPO4x*tds.nx+tds.mflux_cPO4y*tds.ny+tds.mflux_cPO4z*tds.nz	mol/(m²·s)	Normal electrophoretic flux	Boundaries 2–3	
tds.ntflux_cPO4	tds.bndFlux_cPO4	mol/(m²·s)	Normal total flux	Boundaries 2–3	
tds.ndflux_cH	tds.dflux_cHx*tds.nx+tds.dflux_cHy*tds.ny+tds.dflux_cHz*tds.nz	mol/(m²·s)	Normal diffusive flux	Boundaries 2–3	
tds.nmflux_cH	tds.mflux_cHx*tds.nx+tds.mflux_cHy*tds.ny+tds.mflux_cHz*tds.nz	mol/(m²·s)	Normal electrophoretic flux	Boundaries 2–3	
tds.ntflux_cH	tds.bndFlux_cH	mol/(m²·s)	Normal total flux	Boundaries 2–3	
tds.ndflux_cOH	tds.dflux_cOHx*tds.nx+tds.dflux_cOHy*tds.ny+tds.dflux_cOHz*tds.nz	mol/(m²·s)	Normal diffusive flux	Boundaries 2–3	
tds.nmflux_cOH	tds.mflux_cOHx*tds.nx+tds.mflux_cOHy*tds.ny+tds.mflux_cOHz*tds.nz	mol/(m²·s)	Normal electrophoretic flux	Boundaries 2–3	
tds.ntflux_cOH	tds.bndFlux_cOH	mol/(m²·s)	Normal total flux	Boundaries 2–3	
tds.ndflux_cHCO3	tds.dflux_cHCO3x*tds.nx+tds.dflux_cHCO3y*tds.ny+tds.dflux_cHCO3z*tds.nz	mol/(m²·s)	Normal diffusive flux	Boundaries 2–3	
tds.nmflux_cHCO3	tds.mflux_cHCO3x*tds.nx+tds.mflux_cHCO3y*tds.ny+tds.mflux_cHCO3z*tds.nz	mol/(m²·s)	Normal electrophoretic flux	Boundaries 2–3	
tds.ntflux_cHCO3	tds.bndFlux_cHCO3	mol/(m²·s)	Normal total flux	Boundaries 2–3	
tds.ndflux_cCO3	tds.dflux_cCO3x*tds.nx+tds.dflux_cCO3y*tds.ny+tds.dflux_cCO3z*tds.nz	mol/(m²·s)	Normal diffusive flux	Boundaries 2–3	
tds.nmflux_cCO3	tds.mflux_cCO3x*tds.nx+tds.mflux_cCO3y*tds.ny+tds.mflux_cCO3z*tds.nz	mol/(m²·s)	Normal electrophoretic flux	Boundaries 2–3	
tds.ntflux_cCO3	tds.bndFlux_cCO3	mol/(m²·s)	Normal total flux	Boundaries 2–3	

Name	Expression	Unit	Description	Selection	Details
tds.DF_cAscxx	1.0E-9[m^2/s]	m²/s	Fluid diffusion coefficient, xx component	Domain 2	
tds.DF_cAscyx	0	m²/s	Fluid diffusion coefficient, yx component	Domain 2	
tds.DF_cAsczx	0	m²/s	Fluid diffusion coefficient, zx component	Domain 2	
tds.DF_cAscxy	0	m²/s	Fluid diffusion coefficient, xy component	Domain 2	
tds.DF_cAscyy	1.0E-9[m^2/s]	m²/s	Fluid diffusion coefficient, yy component	Domain 2	
tds.DF_cAsczy	0	m²/s	Fluid diffusion coefficient, zy component	Domain 2	
tds.DF_cAscxz	0	m²/s	Fluid diffusion coefficient, xz component	Domain 2	
tds.DF_cAscyz	0	m²/s	Fluid diffusion coefficient, yz component	Domain 2	
tds.DF_cAsczz	1.0E-9[m^2/s]	m²/s	Fluid diffusion coefficient, zz component	Domain 2	
tds.D_cAscxx	tds.DF_cAscxx+tds.DiT_cAsc	m²/s	Diffusion coefficient, xx component	Domain 2	
tds.D_cAscyx	tds.DF_cAscyx	m²/s	Diffusion coefficient, yx component	Domain 2	
tds.D_cAsczx	tds.DF_cAsczx	m²/s	Diffusion coefficient, zx component	Domain 2	
tds.D_cAscxy	tds.DF_cAscxy	m²/s	Diffusion coefficient, xy component	Domain 2	
tds.D_cAscyy	tds.DF_cAscyy+tds.DiT_cAsc	m²/s	Diffusion coefficient, yy component	Domain 2	
tds.D_cAsczy	tds.DF_cAsczy	m²/s	Diffusion	Domain 2	

Name	Expression	Unit	Description	Selection	Details
			coefficient, zy component		
tds.D_cAscxz	tds.DF_cAscxz	m²/s	Diffusion coefficient, xz component	Domain 2	
tds.D_cAscyz	tds.DF_cAscyz	m²/s	Diffusion coefficient, yz component	Domain 2	
tds.D_cAsczz	tds.DF_cAsczz+tds.DiT_cAsc	m²/s	Diffusion coefficient, zz component	Domain 2	
tds.DF_cAscmxx	1.0E-9[m^2/s]	m²/s	Fluid diffusion coefficient, xx component	Domain 2	
tds.DF_cAscmyx	0	m²/s	Fluid diffusion coefficient, yx component	Domain 2	
tds.DF_cAscmzx	0	m²/s	Fluid diffusion coefficient, zx component	Domain 2	
tds.DF_cAscmxy	0	m²/s	Fluid diffusion coefficient, xy component	Domain 2	
tds.DF_cAscmyy	1.0E-9[m^2/s]	m²/s	Fluid diffusion coefficient, yy component	Domain 2	
tds.DF_cAscmzy	0	m²/s	Fluid diffusion coefficient, zy component	Domain 2	
tds.DF_cAscmxz	0	m²/s	Fluid diffusion coefficient, xz component	Domain 2	
tds.DF_cAscmyz	0	m²/s	Fluid diffusion coefficient, yz component	Domain 2	
tds.DF_cAscmzz	1.0E-9[m^2/s]	m²/s	Fluid diffusion coefficient, zz component	Domain 2	
tds.D_cAscmxx	tds.DF_cAscmxx+tds.DiT_cAscm	m²/s	Diffusion coefficient, xx component	Domain 2	
tds.D_cAscmyx	tds.DF_cAscmyx	m²/s	Diffusion coefficient, yx	Domain 2	

Name	Expression	Unit	Description	Selection	Details
			component		
tds.D_cAscmzx	tds.DF_cAscmzx	m²/s	Diffusion coefficient, zx component	Domain 2	
tds.D_cAscmxy	tds.DF_cAscmxy	m²/s	Diffusion coefficient, xy component	Domain 2	
tds.D_cAscmyy	tds.DF_cAscmyy+tds.DiT_cAscm	m²/s	Diffusion coefficient, yy component	Domain 2	
tds.D_cAscmzy	tds.DF_cAscmzy	m²/s	Diffusion coefficient, zy component	Domain 2	
tds.D_cAscmxz	tds.DF_cAscmxz	m²/s	Diffusion coefficient, xz component	Domain 2	
tds.D_cAscmyz	tds.DF_cAscmyz	m²/s	Diffusion coefficient, yz component	Domain 2	
tds.D_cAscmzz	tds.DF_cAscmzz+tds.DiT_cAscm	m²/s	Diffusion coefficient, zz component	Domain 2	
tds.DF_cCO2xx	D_CO2w	m²/s	Fluid diffusion coefficient, xx component	Domain 2	
tds.DF_cCO2yx	0	m²/s	Fluid diffusion coefficient, yx component	Domain 2	
tds.DF_cCO2zx	0	m²/s	Fluid diffusion coefficient, zx component	Domain 2	
tds.DF_cCO2xy	0	m²/s	Fluid diffusion coefficient, xy component	Domain 2	
tds.DF_cCO2yy	D_CO2w	m²/s	Fluid diffusion coefficient, yy component	Domain 2	
tds.DF_cCO2zy	0	m²/s	Fluid diffusion coefficient, zy component	Domain 2	
tds.DF_cCO2xz	0	m²/s	Fluid diffusion coefficient, xz component	Domain 2	

Name	Expression	Unit	Description	Selection	Details
tds.DF_cCO2yz	0	m²/s	Fluid diffusion coefficient, yz component	Domain 2	
tds.DF_cCO2zz	D_CO2w	m²/s	Fluid diffusion coefficient, zz component	Domain 2	
tds.D_cCO2xx	tds.DF_cCO2xx+tds.DiT_cCO2	m²/s	Diffusion coefficient, xx component	Domain 2	
tds.D_cCO2yx	tds.DF_cCO2yx	m²/s	Diffusion coefficient, yx component	Domain 2	
tds.D_cCO2zx	tds.DF_cCO2zx	m²/s	Diffusion coefficient, zx component	Domain 2	
tds.D_cCO2xy	tds.DF_cCO2xy	m²/s	Diffusion coefficient, xy component	Domain 2	
tds.D_cCO2yy	tds.DF_cCO2yy+tds.DiT_cCO2	m²/s	Diffusion coefficient, yy component	Domain 2	
tds.D_cCO2zy	tds.DF_cCO2zy	m²/s	Diffusion coefficient, zy component	Domain 2	
tds.D_cCO2xz	tds.DF_cCO2xz	m²/s	Diffusion coefficient, xz component	Domain 2	
tds.D_cCO2yz	tds.DF_cCO2yz	m²/s	Diffusion coefficient, yz component	Domain 2	
tds.D_cCO2zz	tds.DF_cCO2zz+tds.DiT_cCO2	m²/s	Diffusion coefficient, zz component	Domain 2	
tds.DF_cCOxx	D_COw	m²/s	Fluid diffusion coefficient, xx component	Domain 2	
tds.DF_cCOyx	0	m²/s	Fluid diffusion coefficient, yx component	Domain 2	
tds.DF_cCOzx	0	m²/s	Fluid diffusion coefficient, zx component	Domain 2	
tds.DF_cCOxy	0	m²/s	Fluid diffusion	Domain 2	

Name	Expression	Unit	Description	Selection	Details
			coefficient, xy component		
tds.DF_cCOyy	D_COw	m^2/s	Fluid diffusion coefficient, yy component	Domain 2	
tds.DF_cCOzy	0	m^2/s	Fluid diffusion coefficient, zy component	Domain 2	
tds.DF_cCOxz	0	m^2/s	Fluid diffusion coefficient, xz component	Domain 2	
tds.DF_cCOyz	0	m^2/s	Fluid diffusion coefficient, yz component	Domain 2	
tds.DF_cCOzz	D_COw	m^2/s	Fluid diffusion coefficient, zz component	Domain 2	
tds.D_cCOxx	tds.DF_cCOxx+tds.DiT_cCO	m^2/s	Diffusion coefficient, xx component	Domain 2	
tds.D_cCOyx	tds.DF_cCOyx	m^2/s	Diffusion coefficient, yx component	Domain 2	
tds.D_cCOzx	tds.DF_cCOzx	m^2/s	Diffusion coefficient, zx component	Domain 2	
tds.D_cCOxy	tds.DF_cCOxy	m^2/s	Diffusion coefficient, xy component	Domain 2	
tds.D_cCOyy	tds.DF_cCOyy+tds.DiT_cCO	m^2/s	Diffusion coefficient, yy component	Domain 2	
tds.D_cCOzy	tds.DF_cCOzy	m^2/s	Diffusion coefficient, zy component	Domain 2	
tds.D_cCOxz	tds.DF_cCOxz	m^2/s	Diffusion coefficient, xz component	Domain 2	
tds.D_cCOyz	tds.DF_cCOyz	m^2/s	Diffusion coefficient, yz component	Domain 2	
tds.D_cCOzz	tds.DF_cCOzz+tds.DiT_cCO	m^2/s	Diffusion coefficient, zz	Domain 2	

Name	Expression	Unit	Description	Selection	Details
			component		
tds.DF_cO2xx	D_O2w	m²/s	Fluid diffusion coefficient, xx component	Domain 2	
tds.DF_cO2yx	0	m²/s	Fluid diffusion coefficient, yx component	Domain 2	
tds.DF_cO2zx	0	m²/s	Fluid diffusion coefficient, zx component	Domain 2	
tds.DF_cO2xy	0	m²/s	Fluid diffusion coefficient, xy component	Domain 2	
tds.DF_cO2yy	D_O2w	m²/s	Fluid diffusion coefficient, yy component	Domain 2	
tds.DF_cO2zy	0	m²/s	Fluid diffusion coefficient, zy component	Domain 2	
tds.DF_cO2xz	0	m²/s	Fluid diffusion coefficient, xz component	Domain 2	
tds.DF_cO2yz	0	m²/s	Fluid diffusion coefficient, yz component	Domain 2	
tds.DF_cO2zz	D_O2w	m²/s	Fluid diffusion coefficient, zz component	Domain 2	
tds.D_cO2xx	tds.DF_cO2xx+tds.DiT_cO2	m²/s	Diffusion coefficient, xx component	Domain 2	
tds.D_cO2yx	tds.DF_cO2yx	m²/s	Diffusion coefficient, yx component	Domain 2	
tds.D_cO2zx	tds.DF_cO2zx	m²/s	Diffusion coefficient, zx component	Domain 2	
tds.D_cO2xy	tds.DF_cO2xy	m²/s	Diffusion coefficient, xy component	Domain 2	
tds.D_cO2yy	tds.DF_cO2yy+tds.DiT_cO2	m²/s	Diffusion coefficient, yy component	Domain 2	

Name	Expression	Unit	Description	Selection	Details
tds.D_cO2zy	tds.DF_cO2zy	m²/s	Diffusion coefficient, zy component	Domain 2	
tds.D_cO2xz	tds.DF_cO2xz	m²/s	Diffusion coefficient, xz component	Domain 2	
tds.D_cO2yz	tds.DF_cO2yz	m²/s	Diffusion coefficient, yz component	Domain 2	
tds.D_cO2zz	tds.DF_cO2zz+tds.DiT_cO2	m²/s	Diffusion coefficient, zz component	Domain 2	
tds.DF_cH2Oxx	1.0E-9	m²/s	Fluid diffusion coefficient, xx component	Domain 2	
tds.DF_cH2Oyx	0	m²/s	Fluid diffusion coefficient, yx component	Domain 2	
tds.DF_cH2Ozx	0	m²/s	Fluid diffusion coefficient, zx component	Domain 2	
tds.DF_cH2Oxy	0	m²/s	Fluid diffusion coefficient, xy component	Domain 2	
tds.DF_cH2Oyy	1.0E-9	m²/s	Fluid diffusion coefficient, yy component	Domain 2	
tds.DF_cH2Ozy	0	m²/s	Fluid diffusion coefficient, zy component	Domain 2	
tds.DF_cH2Oxz	0	m²/s	Fluid diffusion coefficient, xz component	Domain 2	
tds.DF_cH2Oyz	0	m²/s	Fluid diffusion coefficient, yz component	Domain 2	
tds.DF_cH2Ozz	1.0E-9	m²/s	Fluid diffusion coefficient, zz component	Domain 2	
tds.D_cH2Oxx	tds.DF_cH2Oxx+tds.DiT_cH2O	m²/s	Diffusion coefficient, xx component	Domain 2	
tds.D_cH2Oyx	tds.DF_cH2Oyx	m²/s	Diffusion	Domain 2	

Name	Expression	Unit	Description	Selection	Details
			coefficient, yx component		
tds.D_cH2Ozx	tds.DF_cH2Ozx	m^2/s	Diffusion coefficient, zx component	Domain 2	
tds.D_cH2Oxy	tds.DF_cH2Oxy	m^2/s	Diffusion coefficient, xy component	Domain 2	
tds.D_cH2Oyy	tds.DF_cH2Oyy+tds.DiT_cH2O	m^2/s	Diffusion coefficient, yy component	Domain 2	
tds.D_cH2Ozy	tds.DF_cH2Ozy	m^2/s	Diffusion coefficient, zy component	Domain 2	
tds.D_cH2Oxz	tds.DF_cH2Oxz	m^2/s	Diffusion coefficient, xz component	Domain 2	
tds.D_cH2Oyz	tds.DF_cH2Oyz	m^2/s	Diffusion coefficient, yz component	Domain 2	
tds.D_cH2Ozz	tds.DF_cH2Ozz+tds.DiT_cH2O	m^2/s	Diffusion coefficient, zz component	Domain 2	
tds.DF_cH2PO4xx	D_H2PO4w	m^2/s	Fluid diffusion coefficient, xx component	Domain 2	
tds.DF_cH2PO4yx	0	m^2/s	Fluid diffusion coefficient, yx component	Domain 2	
tds.DF_cH2PO4zx	0	m^2/s	Fluid diffusion coefficient, zx component	Domain 2	
tds.DF_cH2PO4xy	0	m^2/s	Fluid diffusion coefficient, xy component	Domain 2	
tds.DF_cH2PO4yy	D_H2PO4w	m^2/s	Fluid diffusion coefficient, yy component	Domain 2	
tds.DF_cH2PO4zy	0	m^2/s	Fluid diffusion coefficient, zy component	Domain 2	
tds.DF_cH2PO4xz	0	m^2/s	Fluid diffusion coefficient, xz	Domain 2	

Name	Expression	Unit	Description	Selection	Details
			component		
tds.DF_cH2PO4yz	0	m²/s	Fluid diffusion coefficient, yz component	Domain 2	
tds.DF_cH2PO4zz	D_H2PO4w	m²/s	Fluid diffusion coefficient, zz component	Domain 2	
tds.D_cH2PO4xx	tds.DF_cH2PO4xx+tds.DiT_cH2PO4	m²/s	Diffusion coefficient, xx component	Domain 2	
tds.D_cH2PO4yx	tds.DF_cH2PO4yx	m²/s	Diffusion coefficient, yx component	Domain 2	
tds.D_cH2PO4zx	tds.DF_cH2PO4zx	m²/s	Diffusion coefficient, zx component	Domain 2	
tds.D_cH2PO4xy	tds.DF_cH2PO4xy	m²/s	Diffusion coefficient, xy component	Domain 2	
tds.D_cH2PO4yy	tds.DF_cH2PO4yy+tds.DiT_cH2PO4	m²/s	Diffusion coefficient, yy component	Domain 2	
tds.D_cH2PO4zy	tds.DF_cH2PO4zy	m²/s	Diffusion coefficient, zy component	Domain 2	
tds.D_cH2PO4xz	tds.DF_cH2PO4xz	m²/s	Diffusion coefficient, xz component	Domain 2	
tds.D_cH2PO4yz	tds.DF_cH2PO4yz	m²/s	Diffusion coefficient, yz component	Domain 2	
tds.D_cH2PO4zz	tds.DF_cH2PO4zz+tds.DiT_cH2PO4	m²/s	Diffusion coefficient, zz component	Domain 2	
tds.DF_cHPO4xx	D_HPO4w	m²/s	Fluid diffusion coefficient, xx component	Domain 2	
tds.DF_cHPO4yx	0	m²/s	Fluid diffusion coefficient, yx component	Domain 2	
tds.DF_cHPO4zx	0	m²/s	Fluid diffusion coefficient, zx component	Domain 2	

Name	Expression	Unit	Description	Selection	Details
tds.DF_cHPO4xy	0	m²/s	Fluid diffusion coefficient, xy component	Domain 2	
tds.DF_cHPO4yy	D_HPO4w	m²/s	Fluid diffusion coefficient, yy component	Domain 2	
tds.DF_cHPO4zy	0	m²/s	Fluid diffusion coefficient, zy component	Domain 2	
tds.DF_cHPO4xz	0	m²/s	Fluid diffusion coefficient, xz component	Domain 2	
tds.DF_cHPO4yz	0	m²/s	Fluid diffusion coefficient, yz component	Domain 2	
tds.DF_cHPO4zz	D_HPO4w	m²/s	Fluid diffusion coefficient, zz component	Domain 2	
tds.D_cHPO4xx	tds.DF_cHPO4xx+tds.DiT_cHPO4	m²/s	Diffusion coefficient, xx component	Domain 2	
tds.D_cHPO4yx	tds.DF_cHPO4yx	m²/s	Diffusion coefficient, yx component	Domain 2	
tds.D_cHPO4zx	tds.DF_cHPO4zx	m²/s	Diffusion coefficient, zx component	Domain 2	
tds.D_cHPO4xy	tds.DF_cHPO4xy	m²/s	Diffusion coefficient, xy component	Domain 2	
tds.D_cHPO4yy	tds.DF_cHPO4yy+tds.DiT_cHPO4	m²/s	Diffusion coefficient, yy component	Domain 2	
tds.D_cHPO4zy	tds.DF_cHPO4zy	m²/s	Diffusion coefficient, zy component	Domain 2	
tds.D_cHPO4xz	tds.DF_cHPO4xz	m²/s	Diffusion coefficient, xz component	Domain 2	
tds.D_cHPO4yz	tds.DF_cHPO4yz	m²/s	Diffusion coefficient, yz component	Domain 2	
tds.D_cHPO4zz	tds.DF_cHPO4zz+tds	m²/s	Diffusion	Domain 2	

Name	Expression	Unit	Description	Selection	Details
	.DiT_cHPO4		coefficient, zz component		
tds.DF_cNaxx	D_Naw	m²/s	Fluid diffusion coefficient, xx component	Domain 2	
tds.DF_cNayx	0	m²/s	Fluid diffusion coefficient, yx component	Domain 2	
tds.DF_cNazx	0	m²/s	Fluid diffusion coefficient, zx component	Domain 2	
tds.DF_cNaxy	0	m²/s	Fluid diffusion coefficient, xy component	Domain 2	
tds.DF_cNayy	D_Naw	m²/s	Fluid diffusion coefficient, yy component	Domain 2	
tds.DF_cNazy	0	m²/s	Fluid diffusion coefficient, zy component	Domain 2	
tds.DF_cNaxz	0	m²/s	Fluid diffusion coefficient, xz component	Domain 2	
tds.DF_cNayz	0	m²/s	Fluid diffusion coefficient, yz component	Domain 2	
tds.DF_cNazz	D_Naw	m²/s	Fluid diffusion coefficient, zz component	Domain 2	
tds.D_cNaxx	tds.DF_cNaxx+tds.DiT_cNa	m²/s	Diffusion coefficient, xx component	Domain 2	
tds.D_cNayx	tds.DF_cNayx	m²/s	Diffusion coefficient, yx component	Domain 2	
tds.D_cNazx	tds.DF_cNazx	m²/s	Diffusion coefficient, zx component	Domain 2	
tds.D_cNaxy	tds.DF_cNaxy	m²/s	Diffusion coefficient, xy component	Domain 2	
tds.D_cNayy	tds.DF_cNayy+tds.DiT_cNa	m²/s	Diffusion coefficient, yy	Domain 2	

Name	Expression	Unit	Description	Selection	Details
			component		
tds.D_cNazy	tds.DF_cNazy	m²/s	Diffusion coefficient, zy component	Domain 2	
tds.D_cNaxz	tds.DF_cNaxz	m²/s	Diffusion coefficient, xz component	Domain 2	
tds.D_cNayz	tds.DF_cNayz	m²/s	Diffusion coefficient, yz component	Domain 2	
tds.D_cNazz	tds.DF_cNazz+tds.DiT_cNa	m²/s	Diffusion coefficient, zz component	Domain 2	
tds.DF_cClxx	D_Clw	m²/s	Fluid diffusion coefficient, xx component	Domain 2	
tds.DF_cClyx	0	m²/s	Fluid diffusion coefficient, yx component	Domain 2	
tds.DF_cClzx	0	m²/s	Fluid diffusion coefficient, zx component	Domain 2	
tds.DF_cClxy	0	m²/s	Fluid diffusion coefficient, xy component	Domain 2	
tds.DF_cClyy	D_Clw	m²/s	Fluid diffusion coefficient, yy component	Domain 2	
tds.DF_cClzy	0	m²/s	Fluid diffusion coefficient, zy component	Domain 2	
tds.DF_cClxz	0	m²/s	Fluid diffusion coefficient, xz component	Domain 2	
tds.DF_cClyz	0	m²/s	Fluid diffusion coefficient, yz component	Domain 2	
tds.DF_cClzz	D_Clw	m²/s	Fluid diffusion coefficient, zz component	Domain 2	
tds.D_cClxx	tds.DF_cClxx+tds.DiT_cCl	m²/s	Diffusion coefficient, xx component	Domain 2	

Name	Expression	Unit	Description	Selection	Details
tds.D_cClyx	tds.DF_cClyx	m^2/s	Diffusion coefficient, yx component	Domain 2	
tds.D_cClzx	tds.DF_cClzx	m^2/s	Diffusion coefficient, zx component	Domain 2	
tds.D_cClxy	tds.DF_cClxy	m^2/s	Diffusion coefficient, xy component	Domain 2	
tds.D_cClyy	tds.DF_cClyy+tds.DiT_cCl	m^2/s	Diffusion coefficient, yy component	Domain 2	
tds.D_cClzy	tds.DF_cClzy	m^2/s	Diffusion coefficient, zy component	Domain 2	
tds.D_cClxz	tds.DF_cClxz	m^2/s	Diffusion coefficient, xz component	Domain 2	
tds.D_cClyz	tds.DF_cClyz	m^2/s	Diffusion coefficient, yz component	Domain 2	
tds.D_cClzz	tds.DF_cClzz+tds.DiT_cCl	m^2/s	Diffusion coefficient, zz component	Domain 2	
tds.DF_cH3PO4xx	D_H3PO4w	m^2/s	Fluid diffusion coefficient, xx component	Domain 2	
tds.DF_cH3PO4yx	0	m^2/s	Fluid diffusion coefficient, yx component	Domain 2	
tds.DF_cH3PO4zx	0	m^2/s	Fluid diffusion coefficient, zx component	Domain 2	
tds.DF_cH3PO4xy	0	m^2/s	Fluid diffusion coefficient, xy component	Domain 2	
tds.DF_cH3PO4yy	D_H3PO4w	m^2/s	Fluid diffusion coefficient, yy component	Domain 2	
tds.DF_cH3PO4zy	0	m^2/s	Fluid diffusion coefficient, zy component	Domain 2	
tds.DF_cH3PO4xz	0	m^2/s	Fluid diffusion	Domain 2	

Name	Expression	Unit	Description	Selection	Details
			coefficient, xz component		
tds.DF_cH3PO4yz	0	m^2/s	Fluid diffusion coefficient, yz component	Domain 2	
tds.DF_cH3PO4zz	D_H3PO4w	m^2/s	Fluid diffusion coefficient, zz component	Domain 2	
tds.D_cH3PO4xx	tds.DF_cH3PO4xx+tds.DiT_cH3PO4	m^2/s	Diffusion coefficient, xx component	Domain 2	
tds.D_cH3PO4yx	tds.DF_cH3PO4yx	m^2/s	Diffusion coefficient, yx component	Domain 2	
tds.D_cH3PO4zx	tds.DF_cH3PO4zx	m^2/s	Diffusion coefficient, zx component	Domain 2	
tds.D_cH3PO4xy	tds.DF_cH3PO4xy	m^2/s	Diffusion coefficient, xy component	Domain 2	
tds.D_cH3PO4yy	tds.DF_cH3PO4yy+tds.DiT_cH3PO4	m^2/s	Diffusion coefficient, yy component	Domain 2	
tds.D_cH3PO4zy	tds.DF_cH3PO4zy	m^2/s	Diffusion coefficient, zy component	Domain 2	
tds.D_cH3PO4xz	tds.DF_cH3PO4xz	m^2/s	Diffusion coefficient, xz component	Domain 2	
tds.D_cH3PO4yz	tds.DF_cH3PO4yz	m^2/s	Diffusion coefficient, yz component	Domain 2	
tds.D_cH3PO4zz	tds.DF_cH3PO4zz+tds.DiT_cH3PO4	m^2/s	Diffusion coefficient, zz component	Domain 2	
tds.DF_cPO4xx	D_PO4w	m^2/s	Fluid diffusion coefficient, xx component	Domain 2	
tds.DF_cPO4yx	0	m^2/s	Fluid diffusion coefficient, yx component	Domain 2	
tds.DF_cPO4zx	0	m^2/s	Fluid diffusion coefficient, zx	Domain 2	

Name	Expression	Unit	Description	Selection	Details
			component		
tds.DF_cPO4xy	0	m^2/s	Fluid diffusion coefficient, xy component	Domain 2	
tds.DF_cPO4yy	D_PO4w	m^2/s	Fluid diffusion coefficient, yy component	Domain 2	
tds.DF_cPO4zy	0	m^2/s	Fluid diffusion coefficient, zy component	Domain 2	
tds.DF_cPO4xz	0	m^2/s	Fluid diffusion coefficient, xz component	Domain 2	
tds.DF_cPO4yz	0	m^2/s	Fluid diffusion coefficient, yz component	Domain 2	
tds.DF_cPO4zz	D_PO4w	m^2/s	Fluid diffusion coefficient, zz component	Domain 2	
tds.D_cPO4xx	tds.DF_cPO4xx+tds.DiT_cPO4	m^2/s	Diffusion coefficient, xx component	Domain 2	
tds.D_cPO4yx	tds.DF_cPO4yx	m^2/s	Diffusion coefficient, yx component	Domain 2	
tds.D_cPO4zx	tds.DF_cPO4zx	m^2/s	Diffusion coefficient, zx component	Domain 2	
tds.D_cPO4xy	tds.DF_cPO4xy	m^2/s	Diffusion coefficient, xy component	Domain 2	
tds.D_cPO4yy	tds.DF_cPO4yy+tds.DiT_cPO4	m^2/s	Diffusion coefficient, yy component	Domain 2	
tds.D_cPO4zy	tds.DF_cPO4zy	m^2/s	Diffusion coefficient, zy component	Domain 2	
tds.D_cPO4xz	tds.DF_cPO4xz	m^2/s	Diffusion coefficient, xz component	Domain 2	
tds.D_cPO4yz	tds.DF_cPO4yz	m^2/s	Diffusion coefficient, yz component	Domain 2	

Name	Expression	Unit	Description	Selection	Details
tds.D_cPO4zz	tds.DF_cPO4zz+tds.DiT_cPO4	m^2/s	Diffusion coefficient, zz component	Domain 2	
tds.DF_cHxx	D_Hw	m^2/s	Fluid diffusion coefficient, xx component	Domain 2	
tds.DF_cHyx	0	m^2/s	Fluid diffusion coefficient, yx component	Domain 2	
tds.DF_cHzx	0	m^2/s	Fluid diffusion coefficient, zx component	Domain 2	
tds.DF_cHxy	0	m^2/s	Fluid diffusion coefficient, xy component	Domain 2	
tds.DF_cHyy	D_Hw	m^2/s	Fluid diffusion coefficient, yy component	Domain 2	
tds.DF_cHzy	0	m^2/s	Fluid diffusion coefficient, zy component	Domain 2	
tds.DF_cHxz	0	m^2/s	Fluid diffusion coefficient, xz component	Domain 2	
tds.DF_cHyz	0	m^2/s	Fluid diffusion coefficient, yz component	Domain 2	
tds.DF_cHzz	D_Hw	m^2/s	Fluid diffusion coefficient, zz component	Domain 2	
tds.D_cHxx	tds.DF_cHxx+tds.DiT_cH	m^2/s	Diffusion coefficient, xx component	Domain 2	
tds.D_cHyx	tds.DF_cHyx	m^2/s	Diffusion coefficient, yx component	Domain 2	
tds.D_cHzx	tds.DF_cHzx	m^2/s	Diffusion coefficient, zx component	Domain 2	
tds.D_cHxy	tds.DF_cHxy	m^2/s	Diffusion coefficient, xy component	Domain 2	
tds.D_cHyy	tds.DF_cHyy+tds.DiT	m^2/s	Diffusion	Domain 2	

Name	Expression	Unit	Description	Selection	Details
	_cH		coefficient, yy component		
tds.D_cHzy	tds.DF_cHzy	m^2/s	Diffusion coefficient, zy component	Domain 2	
tds.D_cHxz	tds.DF_cHxz	m^2/s	Diffusion coefficient, xz component	Domain 2	
tds.D_cHyz	tds.DF_cHyz	m^2/s	Diffusion coefficient, yz component	Domain 2	
tds.D_cHzz	tds.DF_cHzz+tds.DiT_cH	m^2/s	Diffusion coefficient, zz component	Domain 2	
tds.DF_cOHxx	D_OHw	m^2/s	Fluid diffusion coefficient, xx component	Domain 2	
tds.DF_cOHyx	0	m^2/s	Fluid diffusion coefficient, yx component	Domain 2	
tds.DF_cOHzx	0	m^2/s	Fluid diffusion coefficient, zx component	Domain 2	
tds.DF_cOHxy	0	m^2/s	Fluid diffusion coefficient, xy component	Domain 2	
tds.DF_cOHyy	D_OHw	m^2/s	Fluid diffusion coefficient, yy component	Domain 2	
tds.DF_cOHzy	0	m^2/s	Fluid diffusion coefficient, zy component	Domain 2	
tds.DF_cOHxz	0	m^2/s	Fluid diffusion coefficient, xz component	Domain 2	
tds.DF_cOHyz	0	m^2/s	Fluid diffusion coefficient, yz component	Domain 2	
tds.DF_cOHzz	D_OHw	m^2/s	Fluid diffusion coefficient, zz component	Domain 2	
tds.D_cOHxx	tds.DF_cOHxx+tds.DiT_cOH	m^2/s	Diffusion coefficient, xx	Domain 2	

Name	Expression	Unit	Description	Selection	Details
			component		
tds.D_cOHyx	tds.DF_cOHyx	m²/s	Diffusion coefficient, yx component	Domain 2	
tds.D_cOHzx	tds.DF_cOHzx	m²/s	Diffusion coefficient, zx component	Domain 2	
tds.D_cOHxy	tds.DF_cOHxy	m²/s	Diffusion coefficient, xy component	Domain 2	
tds.D_cOHyy	tds.DF_cOHyy+tds.DiT_cOH	m²/s	Diffusion coefficient, yy component	Domain 2	
tds.D_cOHzy	tds.DF_cOHzy	m²/s	Diffusion coefficient, zy component	Domain 2	
tds.D_cOHxz	tds.DF_cOHxz	m²/s	Diffusion coefficient, xz component	Domain 2	
tds.D_cOHyz	tds.DF_cOHyz	m²/s	Diffusion coefficient, yz component	Domain 2	
tds.D_cOHzz	tds.DF_cOHzz+tds.DiT_cOH	m²/s	Diffusion coefficient, zz component	Domain 2	
tds.DF_cHCO3xx	D_HCO3w	m²/s	Fluid diffusion coefficient, xx component	Domain 2	
tds.DF_cHCO3yx	0	m²/s	Fluid diffusion coefficient, yx component	Domain 2	
tds.DF_cHCO3zx	0	m²/s	Fluid diffusion coefficient, zx component	Domain 2	
tds.DF_cHCO3xy	0	m²/s	Fluid diffusion coefficient, xy component	Domain 2	
tds.DF_cHCO3yy	D_HCO3w	m²/s	Fluid diffusion coefficient, yy component	Domain 2	
tds.DF_cHCO3zy	0	m²/s	Fluid diffusion coefficient, zy component	Domain 2	

Name	Expression	Unit	Description	Selection	Details
tds.DF_cHCO3xz	0	m²/s	Fluid diffusion coefficient, xz component	Domain 2	
tds.DF_cHCO3yz	0	m²/s	Fluid diffusion coefficient, yz component	Domain 2	
tds.DF_cHCO3zz	D_HCO3w	m²/s	Fluid diffusion coefficient, zz component	Domain 2	
tds.D_cHCO3xx	tds.DF_cHCO3xx+tds.DiT_cHCO3	m²/s	Diffusion coefficient, xx component	Domain 2	
tds.D_cHCO3yx	tds.DF_cHCO3yx	m²/s	Diffusion coefficient, yx component	Domain 2	
tds.D_cHCO3zx	tds.DF_cHCO3zx	m²/s	Diffusion coefficient, zx component	Domain 2	
tds.D_cHCO3xy	tds.DF_cHCO3xy	m²/s	Diffusion coefficient, xy component	Domain 2	
tds.D_cHCO3yy	tds.DF_cHCO3yy+tds.DiT_cHCO3	m²/s	Diffusion coefficient, yy component	Domain 2	
tds.D_cHCO3zy	tds.DF_cHCO3zy	m²/s	Diffusion coefficient, zy component	Domain 2	
tds.D_cHCO3xz	tds.DF_cHCO3xz	m²/s	Diffusion coefficient, xz component	Domain 2	
tds.D_cHCO3yz	tds.DF_cHCO3yz	m²/s	Diffusion coefficient, yz component	Domain 2	
tds.D_cHCO3zz	tds.DF_cHCO3zz+tds.DiT_cHCO3	m²/s	Diffusion coefficient, zz component	Domain 2	
tds.DF_cCO3xx	D_CO3w	m²/s	Fluid diffusion coefficient, xx component	Domain 2	
tds.DF_cCO3yx	0	m²/s	Fluid diffusion coefficient, yx component	Domain 2	
tds.DF_cCO3zx	0	m²/s	Fluid diffusion	Domain 2	

Name	Expression	Unit	Description	Selection	Details
			coefficient, zx component		
tds.DF_cCO3xy	0	m²/s	Fluid diffusion coefficient, xy component	Domain 2	
tds.DF_cCO3yy	D_CO3w	m²/s	Fluid diffusion coefficient, yy component	Domain 2	
tds.DF_cCO3zy	0	m²/s	Fluid diffusion coefficient, zy component	Domain 2	
tds.DF_cCO3xz	0	m²/s	Fluid diffusion coefficient, xz component	Domain 2	
tds.DF_cCO3yz	0	m²/s	Fluid diffusion coefficient, yz component	Domain 2	
tds.DF_cCO3zz	D_CO3w	m²/s	Fluid diffusion coefficient, zz component	Domain 2	
tds.D_cCO3xx	tds.DF_cCO3xx+tds.DiT_cCO3	m²/s	Diffusion coefficient, xx component	Domain 2	
tds.D_cCO3yx	tds.DF_cCO3yx	m²/s	Diffusion coefficient, yx component	Domain 2	
tds.D_cCO3zx	tds.DF_cCO3zx	m²/s	Diffusion coefficient, zx component	Domain 2	
tds.D_cCO3xy	tds.DF_cCO3xy	m²/s	Diffusion coefficient, xy component	Domain 2	
tds.D_cCO3yy	tds.DF_cCO3yy+tds.DiT_cCO3	m²/s	Diffusion coefficient, yy component	Domain 2	
tds.D_cCO3zy	tds.DF_cCO3zy	m²/s	Diffusion coefficient, zy component	Domain 2	
tds.D_cCO3xz	tds.DF_cCO3xz	m²/s	Diffusion coefficient, xz component	Domain 2	
tds.D_cCO3yz	tds.DF_cCO3yz	m²/s	Diffusion coefficient, yz	Domain 2	

Name	Expression	Unit	Description	Selection	Details
			component		
tds.D_cCO3zz	tds.DF_cCO3zz+tds.DiT_cCO3	m^2/s	Diffusion coefficient, zz component	Domain 2	
tds.Dav_cAsc	tds.D_cAscxx	m^2/s	Average diffusion coefficient	Domain 2	
tds.Dav_cAscm	tds.D_cAscmxx	m^2/s	Average diffusion coefficient	Domain 2	
tds.Dav_cCO2	tds.D_cCO2xx	m^2/s	Average diffusion coefficient	Domain 2	
tds.Dav_cCO	tds.D_cCOxx	m^2/s	Average diffusion coefficient	Domain 2	
tds.Dav_cO2	tds.D_cO2xx	m^2/s	Average diffusion coefficient	Domain 2	
tds.Dav_cH2O	tds.D_cH2Oxx	m^2/s	Average diffusion coefficient	Domain 2	
tds.Dav_cH2PO4	tds.D_cH2PO4xx	m^2/s	Average diffusion coefficient	Domain 2	
tds.Dav_cHPO4	tds.D_cHPO4xx	m^2/s	Average diffusion coefficient	Domain 2	
tds.Dav_cNa	tds.D_cNaxx	m^2/s	Average diffusion coefficient	Domain 2	
tds.Dav_cCl	tds.D_cClxx	m^2/s	Average diffusion coefficient	Domain 2	
tds.Dav_cH3PO4	tds.D_cH3PO4xx	m^2/s	Average diffusion coefficient	Domain 2	
tds.Dav_cPO4	tds.D_cPO4xx	m^2/s	Average diffusion coefficient	Domain 2	
tds.Dav_cH	tds.D_cHxx	m^2/s	Average diffusion coefficient	Domain 2	
tds.Dav_cOH	tds.D_cOHxx	m^2/s	Average diffusion coefficient	Domain 2	
tds.Dav_cHCO3	tds.D_cHCO3xx	m^2/s	Average diffusion coefficient	Domain 2	
tds.Dav_cCO3	tds.D_cCO3xx	m^2/s	Average diffusion coefficient	Domain 2	
tds.tflux_cAscx	tds.dflux_cAscx+tds.mflux_cAscx	$mol/(m^2 \cdot s)$	Total flux, x component	Domain 2	+ operation
tds.tflux_cAscy	tds.dflux_cAscy+tds.mflux_cAscy	$mol/(m^2 \cdot s)$	Total flux, y component	Domain 2	+ operation
tds.tflux_cAscz	tds.dflux_cAscz+tds.	$mol/(m^2 \cdot s)$	Total flux, z	Domain 2	+ operation

Name	Expression	Unit	Description	Selection	Details
	mflux_cAscz		component		
tds.dfluxMag_cAsc	sqrt(tds.dflux_cAscx^2+tds.dflux_cAscy^2+tds.dflux_cAscz^2)	mol/(m²·s)	Diffusive flux magnitude	Domain 2	
tds.tfluxMag_cAsc	sqrt(tds.tflux_cAscx^2+tds.tflux_cAscy^2+tds.tflux_cAscz^2)	mol/(m²·s)	Total flux magnitude	Domain 2	
tds.dpflux_cAscx	0	mol/(m²·s)	Dispersive flux, x component	Domain 2	
tds.dpflux_cAscy	0	mol/(m²·s)	Dispersive flux, y component	Domain 2	
tds.dpflux_cAscz	0	mol/(m²·s)	Dispersive flux, z component	Domain 2	
tds.mflux_cAscx	-tds.z_cAsc*F_const*cAsc*tds.um_cAscxx*d(tds.V,x)	mol/(m²·s)	Electrophoretic flux, x component	Domain 2	
tds.mflux_cAscy	-tds.z_cAsc*F_const*cAsc*tds.um_cAscyx*d(tds.V,x)	mol/(m²·s)	Electrophoretic flux, y component	Domain 2	
tds.mflux_cAscz	-tds.z_cAsc*F_const*cAsc*tds.um_cAsczx*d(tds.V,x)	mol/(m²·s)	Electrophoretic flux, z component	Domain 2	
tds.mfluxMag_cAsc	sqrt(tds.mflux_cAscx^2+tds.mflux_cAscy^2+tds.mflux_cAscz^2)	mol/(m²·s)	Electrophoretic flux magnitude	Domain 2	
tds.tflux_cAscmx	tds.dflux_cAscmx+tds.mflux_cAscmx	mol/(m²·s)	Total flux, x component	Domain 2	+ operation
tds.tflux_cAscmy	tds.dflux_cAscmy+tds.mflux_cAscmy	mol/(m²·s)	Total flux, y component	Domain 2	+ operation
tds.tflux_cAscmz	tds.dflux_cAscmz+tds.mflux_cAscmz	mol/(m²·s)	Total flux, z component	Domain 2	+ operation
tds.dfluxMag_cAscm	sqrt(tds.dflux_cAscmx^2+tds.dflux_cAscmy^2+tds.dflux_cAscmz^2)	mol/(m²·s)	Diffusive flux magnitude	Domain 2	
tds.tfluxMag_cAscm	sqrt(tds.tflux_cAscmx^2+tds.tflux_cAscmy^2+tds.tflux_cAsc	mol/(m²·s)	Total flux magnitude	Domain 2	

Name	Expression	Unit	Description	Selection	Details
	mz^2)				
tds.dpflux_cAscmx	0	mol/(m²·s)	Dispersive flux, x component	Domain 2	
tds.dpflux_cAscmy	0	mol/(m²·s)	Dispersive flux, y component	Domain 2	
tds.dpflux_cAscmz	0	mol/(m²·s)	Dispersive flux, z component	Domain 2	
tds.mflux_cAscmx	-tds.z_cAscm*F_const*cAscm*tds.um_cAscmxx*d(tds.V,x)	mol/(m²·s)	Electrophoretic flux, x component	Domain 2	
tds.mflux_cAscmy	-tds.z_cAscm*F_const*cAscm*tds.um_cAscmyx*d(tds.V,x)	mol/(m²·s)	Electrophoretic flux, y component	Domain 2	
tds.mflux_cAscmz	-tds.z_cAscm*F_const*cAscm*tds.um_cAscmzx*d(tds.V,x)	mol/(m²·s)	Electrophoretic flux, z component	Domain 2	
tds.mfluxMag_cAscm	sqrt(tds.mflux_cAscmx^2+tds.mflux_cAscmy^2+tds.mflux_cAscmz^2)	mol/(m²·s)	Electrophoretic flux magnitude	Domain 2	
tds.tflux_cCO2x	tds.dflux_cCO2x+tds.mflux_cCO2x	mol/(m²·s)	Total flux, x component	Domain 2	+ operation
tds.tflux_cCO2y	tds.dflux_cCO2y+tds.mflux_cCO2y	mol/(m²·s)	Total flux, y component	Domain 2	+ operation
tds.tflux_cCO2z	tds.dflux_cCO2z+tds.mflux_cCO2z	mol/(m²·s)	Total flux, z component	Domain 2	+ operation
tds.dfluxMag_cCO2	sqrt(tds.dflux_cCO2x^2+tds.dflux_cCO2y^2+tds.dflux_cCO2z^2)	mol/(m²·s)	Diffusive flux magnitude	Domain 2	
tds.tfluxMag_cCO2	sqrt(tds.tflux_cCO2x^2+tds.tflux_cCO2y^2+tds.tflux_cCO2z^2)	mol/(m²·s)	Total flux magnitude	Domain 2	
tds.dpflux_cCO2x	0	mol/(m²·s)	Dispersive flux, x component	Domain 2	
tds.dpflux_cCO2y	0	mol/(m²·s)	Dispersive flux, y component	Domain 2	
tds.dpflux_cCO2z	0	mol/(m²·s)	Dispersive flux, z	Domain 2	

Name	Expression	Unit	Description	Selection	Details
			component		
tds.mflux_cCO2x	-tds.z_cCO2*F_const*cCO2*tds.um_cCO2xx*d(tds.V,x)	mol/(m²·s)	Electrophoretic flux, x component	Domain 2	
tds.mflux_cCO2y	-tds.z_cCO2*F_const*cCO2*tds.um_cCO2yx*d(tds.V,x)	mol/(m²·s)	Electrophoretic flux, y component	Domain 2	
tds.mflux_cCO2z	-tds.z_cCO2*F_const*cCO2*tds.um_cCO2zx*d(tds.V,x)	mol/(m²·s)	Electrophoretic flux, z component	Domain 2	
tds.mfluxMag_cCO2	sqrt(tds.mflux_cCO2x^2+tds.mflux_cCO2y^2+tds.mflux_cCO2z^2)	mol/(m²·s)	Electrophoretic flux magnitude	Domain 2	
tds.tflux_cCOx	tds.dflux_cCOx+tds.mflux_cCOx	mol/(m²·s)	Total flux, x component	Domain 2	+ operation
tds.tflux_cCOy	tds.dflux_cCOy+tds.mflux_cCOy	mol/(m²·s)	Total flux, y component	Domain 2	+ operation
tds.tflux_cCOz	tds.dflux_cCOz+tds.mflux_cCOz	mol/(m²·s)	Total flux, z component	Domain 2	+ operation
tds.dfluxMag_cCO	sqrt(tds.dflux_cCOx^2+tds.dflux_cCOy^2+tds.dflux_cCOz^2)	mol/(m²·s)	Diffusive flux magnitude	Domain 2	
tds.tfluxMag_cCO	sqrt(tds.tflux_cCOx^2+tds.tflux_cCOy^2+tds.tflux_cCOz^2)	mol/(m²·s)	Total flux magnitude	Domain 2	
tds.dpflux_cCOx	0	mol/(m²·s)	Dispersive flux, x component	Domain 2	
tds.dpflux_cCOy	0	mol/(m²·s)	Dispersive flux, y component	Domain 2	
tds.dpflux_cCOz	0	mol/(m²·s)	Dispersive flux, z component	Domain 2	
tds.mflux_cCOx	-tds.z_cCO*F_const*cCO*tds.um_cCOxx*d(tds.V,x)	mol/(m²·s)	Electrophoretic flux, x component	Domain 2	
tds.mflux_cCOy	-tds.z_cCO*F_const*cCO*tds.um_cCOyx*d(tds.V,x)	mol/(m²·s)	Electrophoretic flux, y component	Domain 2	

Name	Expression	Unit	Description	Selection	Details
tds.mflux_cCOz	-tds.z_cCO*F_const*cCO*tds.um_cCOzx*d(tds.V,x)	mol/(m²·s)	Electrophoretic flux, z component	Domain 2	
tds.mfluxMag_cCO	sqrt(tds.mflux_cCOx^2+tds.mflux_cCOy^2+tds.mflux_cCOz^2)	mol/(m²·s)	Electrophoretic flux magnitude	Domain 2	
tds.tflux_cO2x	tds.dflux_cO2x+tds.mflux_cO2x	mol/(m²·s)	Total flux, x component	Domain 2	+ operation
tds.tflux_cO2y	tds.dflux_cO2y+tds.mflux_cO2y	mol/(m²·s)	Total flux, y component	Domain 2	+ operation
tds.tflux_cO2z	tds.dflux_cO2z+tds.mflux_cO2z	mol/(m²·s)	Total flux, z component	Domain 2	+ operation
tds.dfluxMag_cO2	sqrt(tds.dflux_cO2x^2+tds.dflux_cO2y^2+tds.dflux_cO2z^2)	mol/(m²·s)	Diffusive flux magnitude	Domain 2	
tds.tfluxMag_cO2	sqrt(tds.tflux_cO2x^2+tds.tflux_cO2y^2+tds.tflux_cO2z^2)	mol/(m²·s)	Total flux magnitude	Domain 2	
tds.dpflux_cO2x	0	mol/(m²·s)	Dispersive flux, x component	Domain 2	
tds.dpflux_cO2y	0	mol/(m²·s)	Dispersive flux, y component	Domain 2	
tds.dpflux_cO2z	0	mol/(m²·s)	Dispersive flux, z component	Domain 2	
tds.mflux_cO2x	-tds.z_cO2*F_const*cO2*tds.um_cO2xx*d(tds.V,x)	mol/(m²·s)	Electrophoretic flux, x component	Domain 2	
tds.mflux_cO2y	-tds.z_cO2*F_const*cO2*tds.um_cO2yx*d(tds.V,x)	mol/(m²·s)	Electrophoretic flux, y component	Domain 2	
tds.mflux_cO2z	-tds.z_cO2*F_const*cO2*tds.um_cO2zx*d(tds.V,x)	mol/(m²·s)	Electrophoretic flux, z component	Domain 2	
tds.mfluxMag_cO2	sqrt(tds.mflux_cO2x^2+tds.mflux_cO2y^2+tds.mflux_cO2z^2)	mol/(m²·s)	Electrophoretic flux magnitude	Domain 2	
tds.tflux_cH2Ox	tds.dflux_cH2Ox+tds	mol/(m²·s)	Total flux, x	Domain 2	+ operation

Name	Expression	Unit	Description	Selection	Details
	.mflux_cH2Ox		component		
tds.tflux_cH2Oy	tds.dflux_cH2Oy+tds.mflux_cH2Oy	mol/(m²·s)	Total flux, y component	Domain 2	+ operation
tds.tflux_cH2Oz	tds.dflux_cH2Oz+tds.mflux_cH2Oz	mol/(m²·s)	Total flux, z component	Domain 2	+ operation
tds.dfluxMag_cH2O	sqrt(tds.dflux_cH2Ox^2+tds.dflux_cH2Oy^2+tds.dflux_cH2Oz^2)	mol/(m²·s)	Diffusive flux magnitude	Domain 2	
tds.tfluxMag_cH2O	sqrt(tds.tflux_cH2Ox^2+tds.tflux_cH2Oy^2+tds.tflux_cH2Oz^2)	mol/(m²·s)	Total flux magnitude	Domain 2	
tds.dpflux_cH2Ox	0	mol/(m²·s)	Dispersive flux, x component	Domain 2	
tds.dpflux_cH2Oy	0	mol/(m²·s)	Dispersive flux, y component	Domain 2	
tds.dpflux_cH2Oz	0	mol/(m²·s)	Dispersive flux, z component	Domain 2	
tds.mflux_cH2Ox	-tds.z_cH2O*F_const*cH2O*tds.um_cH2Oxx*d(tds.V,x)	mol/(m²·s)	Electrophoretic flux, x component	Domain 2	
tds.mflux_cH2Oy	-tds.z_cH2O*F_const*cH2O*tds.um_cH2Oyx*d(tds.V,x)	mol/(m²·s)	Electrophoretic flux, y component	Domain 2	
tds.mflux_cH2Oz	-tds.z_cH2O*F_const*cH2O*tds.um_cH2Ozx*d(tds.V,x)	mol/(m²·s)	Electrophoretic flux, z component	Domain 2	
tds.mfluxMag_cH2O	sqrt(tds.mflux_cH2Ox^2+tds.mflux_cH2Oy^2+tds.mflux_cH2Oz^2)	mol/(m²·s)	Electrophoretic flux magnitude	Domain 2	
tds.tflux_cH2PO4x	tds.dflux_cH2PO4x+tds.mflux_cH2PO4x	mol/(m²·s)	Total flux, x component	Domain 2	+ operation
tds.tflux_cH2PO4y	tds.dflux_cH2PO4y+tds.mflux_cH2PO4y	mol/(m²·s)	Total flux, y component	Domain 2	+ operation
tds.tflux_cH2PO4z	tds.dflux_cH2PO4z+tds.mflux_cH2PO4z	mol/(m²·s)	Total flux, z component	Domain 2	+ operation
tds.dfluxMag_cH2P	sqrt(tds.dflux_cH2PO4x^2+tds.dflux_cH	mol/(m²·s)	Diffusive flux	Domain 2	

Name	Expression	Unit	Description	Selection	Details
O4	2PO4y^2+tds.dflux_cH2PO4z^2)		magnitude		
tds.tfluxMag_cH2PO4	sqrt(tds.tflux_cH2PO4x^2+tds.tflux_cH2PO4y^2+tds.tflux_cH2PO4z^2)	mol/(m²·s)	Total flux magnitude	Domain 2	
tds.dpflux_cH2PO4x	0	mol/(m²·s)	Dispersive flux, x component	Domain 2	
tds.dpflux_cH2PO4y	0	mol/(m²·s)	Dispersive flux, y component	Domain 2	
tds.dpflux_cH2PO4z	0	mol/(m²·s)	Dispersive flux, z component	Domain 2	
tds.mflux_cH2PO4x	-tds.z_cH2PO4*F_const*cH2PO4*tds.um_cH2PO4xx*d(tds.V,x)	mol/(m²·s)	Electrophoretic flux, x component	Domain 2	
tds.mflux_cH2PO4y	-tds.z_cH2PO4*F_const*cH2PO4*tds.um_cH2PO4yx*d(tds.V,x)	mol/(m²·s)	Electrophoretic flux, y component	Domain 2	
tds.mflux_cH2PO4z	-tds.z_cH2PO4*F_const*cH2PO4*tds.um_cH2PO4zx*d(tds.V,x)	mol/(m²·s)	Electrophoretic flux, z component	Domain 2	
tds.mfluxMag_cH2PO4	sqrt(tds.mflux_cH2PO4x^2+tds.mflux_cH2PO4y^2+tds.mflux_cH2PO4z^2)	mol/(m²·s)	Electrophoretic flux magnitude	Domain 2	
tds.tflux_cHPO4x	tds.dflux_cHPO4x+tds.mflux_cHPO4x	mol/(m²·s)	Total flux, x component	Domain 2	+ operation
tds.tflux_cHPO4y	tds.dflux_cHPO4y+tds.mflux_cHPO4y	mol/(m²·s)	Total flux, y component	Domain 2	+ operation
tds.tflux_cHPO4z	tds.dflux_cHPO4z+tds.mflux_cHPO4z	mol/(m²·s)	Total flux, z component	Domain 2	+ operation
tds.dfluxMag_cHPO4	sqrt(tds.dflux_cHPO4x^2+tds.dflux_cHPO4y^2+tds.dflux_cHPO4z^2)	mol/(m²·s)	Diffusive flux magnitude	Domain 2	
tds.tfluxMag_cHPO4	sqrt(tds.tflux_cHPO4x^2+tds.tflux_cHPO4y^2+tds.tflux_cHPO4z^2)	mol/(m²·s)	Total flux magnitude	Domain 2	
tds.dpflux_cHPO4x	0	mol/(m²·s)	Dispersive flux, x	Domain 2	

Name	Expression	Unit	Description	Selection	Details
			component		
tds.dpflux_cHPO4y	0	mol/(m²·s)	Dispersive flux, y component	Domain 2	
tds.dpflux_cHPO4z	0	mol/(m²·s)	Dispersive flux, z component	Domain 2	
tds.mflux_cHPO4x	-tds.z_cHPO4*F_const*cHPO4*tds.um_cHPO4xx*d(tds.V,x)	mol/(m²·s)	Electrophoretic flux, x component	Domain 2	
tds.mflux_cHPO4y	-tds.z_cHPO4*F_const*cHPO4*tds.um_cHPO4yx*d(tds.V,x)	mol/(m²·s)	Electrophoretic flux, y component	Domain 2	
tds.mflux_cHPO4z	-tds.z_cHPO4*F_const*cHPO4*tds.um_cHPO4zx*d(tds.V,x)	mol/(m²·s)	Electrophoretic flux, z component	Domain 2	
tds.mfluxMag_cHPO4	sqrt(tds.mflux_cHPO4x^2+tds.mflux_cHPO4y^2+tds.mflux_cHPO4z^2)	mol/(m²·s)	Electrophoretic flux magnitude	Domain 2	
tds.tflux_cNax	tds.dflux_cNax+tds.mflux_cNax	mol/(m²·s)	Total flux, x component	Domain 2	+ operation
tds.tflux_cNay	tds.dflux_cNay+tds.mflux_cNay	mol/(m²·s)	Total flux, y component	Domain 2	+ operation
tds.tflux_cNaz	tds.dflux_cNaz+tds.mflux_cNaz	mol/(m²·s)	Total flux, z component	Domain 2	+ operation
tds.dfluxMag_cNa	sqrt(tds.dflux_cNax^2+tds.dflux_cNay^2+tds.dflux_cNaz^2)	mol/(m²·s)	Diffusive flux magnitude	Domain 2	
tds.tfluxMag_cNa	sqrt(tds.tflux_cNax^2+tds.tflux_cNay^2+tds.tflux_cNaz^2)	mol/(m²·s)	Total flux magnitude	Domain 2	
tds.dpflux_cNax	0	mol/(m²·s)	Dispersive flux, x component	Domain 2	
tds.dpflux_cNay	0	mol/(m²·s)	Dispersive flux, y component	Domain 2	
tds.dpflux_cNaz	0	mol/(m²·s)	Dispersive flux, z component	Domain 2	
tds.mflux_cNax	-tds.z_cNa*F_const*cNa*tds.um_cNaxx*d(	mol/(m²·s)	Electrophoretic flux, x component	Domain 2	

Name	Expression	Unit	Description	Selection	Details
	tds.V,x)				
tds.mflux_cNay	-tds.z_cNa*F_const*cNa*tds.um_cNayx*d(tds.V,x)	mol/(m²·s)	Electrophoretic flux, y component	Domain 2	
tds.mflux_cNaz	-tds.z_cNa*F_const*cNa*tds.um_cNazx*d(tds.V,x)	mol/(m²·s)	Electrophoretic flux, z component	Domain 2	
tds.mfluxMag_cNa	sqrt(tds.mflux_cNax^2+tds.mflux_cNay^2+tds.mflux_cNaz^2)	mol/(m²·s)	Electrophoretic flux magnitude	Domain 2	
tds.tflux_cClx	tds.dflux_cClx+tds.mflux_cClx	mol/(m²·s)	Total flux, x component	Domain 2	+ operation
tds.tflux_cCly	tds.dflux_cCly+tds.mflux_cCly	mol/(m²·s)	Total flux, y component	Domain 2	+ operation
tds.tflux_cClz	tds.dflux_cClz+tds.mflux_cClz	mol/(m²·s)	Total flux, z component	Domain 2	+ operation
tds.dfluxMag_cCl	sqrt(tds.dflux_cClx^2+tds.dflux_cCly^2+tds.dflux_cClz^2)	mol/(m²·s)	Diffusive flux magnitude	Domain 2	
tds.tfluxMag_cCl	sqrt(tds.tflux_cClx^2+tds.tflux_cCly^2+tds.tflux_cClz^2)	mol/(m²·s)	Total flux magnitude	Domain 2	
tds.dpflux_cClx	0	mol/(m²·s)	Dispersive flux, x component	Domain 2	
tds.dpflux_cCly	0	mol/(m²·s)	Dispersive flux, y component	Domain 2	
tds.dpflux_cClz	0	mol/(m²·s)	Dispersive flux, z component	Domain 2	
tds.mflux_cClx	-tds.z_cCl*F_const*cCl*tds.um_cClxx*d(tds.V,x)	mol/(m²·s)	Electrophoretic flux, x component	Domain 2	
tds.mflux_cCly	-tds.z_cCl*F_const*cCl*tds.um_cClyx*d(tds.V,x)	mol/(m²·s)	Electrophoretic flux, y component	Domain 2	
tds.mflux_cClz	-tds.z_cCl*F_const*cCl*tds.um_cClzx*d(tds.V,x)	mol/(m²·s)	Electrophoretic flux, z component	Domain 2	

Name	Expression	Unit	Description	Selection	Details
tds.mfluxMag_cCl	sqrt(tds.mflux_cClx^2+tds.mflux_cCly^2+tds.mflux_cClz^2)	mol/(m²·s)	Electrophoretic flux magnitude	Domain 2	
tds.tflux_cH3PO4x	tds.dflux_cH3PO4x+tds.mflux_cH3PO4x	mol/(m²·s)	Total flux, x component	Domain 2	+ operation
tds.tflux_cH3PO4y	tds.dflux_cH3PO4y+tds.mflux_cH3PO4y	mol/(m²·s)	Total flux, y component	Domain 2	+ operation
tds.tflux_cH3PO4z	tds.dflux_cH3PO4z+tds.mflux_cH3PO4z	mol/(m²·s)	Total flux, z component	Domain 2	+ operation
tds.dfluxMag_cH3PO4	sqrt(tds.dflux_cH3PO4x^2+tds.dflux_cH3PO4y^2+tds.dflux_cH3PO4z^2)	mol/(m²·s)	Diffusive flux magnitude	Domain 2	
tds.tfluxMag_cH3PO4	sqrt(tds.tflux_cH3PO4x^2+tds.tflux_cH3PO4y^2+tds.tflux_cH3PO4z^2)	mol/(m²·s)	Total flux magnitude	Domain 2	
tds.dpflux_cH3PO4x	0	mol/(m²·s)	Dispersive flux, x component	Domain 2	
tds.dpflux_cH3PO4y	0	mol/(m²·s)	Dispersive flux, y component	Domain 2	
tds.dpflux_cH3PO4z	0	mol/(m²·s)	Dispersive flux, z component	Domain 2	
tds.mflux_cH3PO4x	-tds.z_cH3PO4*F_const*cH3PO4*tds.um_cH3PO4xx*d(tds.V,x)	mol/(m²·s)	Electrophoretic flux, x component	Domain 2	
tds.mflux_cH3PO4y	-tds.z_cH3PO4*F_const*cH3PO4*tds.um_cH3PO4yx*d(tds.V,x)	mol/(m²·s)	Electrophoretic flux, y component	Domain 2	
tds.mflux_cH3PO4z	-tds.z_cH3PO4*F_const*cH3PO4*tds.um_cH3PO4zx*d(tds.V,x)	mol/(m²·s)	Electrophoretic flux, z component	Domain 2	
tds.mfluxMag_cH3PO4	sqrt(tds.mflux_cH3PO4x^2+tds.mflux_cH3PO4y^2+tds.mflux_cH3PO4z^2)	mol/(m²·s)	Electrophoretic flux magnitude	Domain 2	
tds.tflux_cPO4x	tds.dflux_cPO4x+tds.mflux_cPO4x	mol/(m²·s)	Total flux, x component	Domain 2	+ operation
tds.tflux_cPO4y	tds.dflux_cPO4y+tds.mflux_cPO4y	mol/(m²·s)	Total flux, y component	Domain 2	+ operation

Name	Expression	Unit	Description	Selection	Details
tds.tflux_cPO4z	tds.dflux_cPO4z+tds.mflux_cPO4z	mol/(m²·s)	Total flux, z component	Domain 2	+ operation
tds.dfluxMag_cPO4	sqrt(tds.dflux_cPO4x^2+tds.dflux_cPO4y^2+tds.dflux_cPO4z^2)	mol/(m²·s)	Diffusive flux magnitude	Domain 2	
tds.tfluxMag_cPO4	sqrt(tds.tflux_cPO4x^2+tds.tflux_cPO4y^2+tds.tflux_cPO4z^2)	mol/(m²·s)	Total flux magnitude	Domain 2	
tds.dpflux_cPO4x	0	mol/(m²·s)	Dispersive flux, x component	Domain 2	
tds.dpflux_cPO4y	0	mol/(m²·s)	Dispersive flux, y component	Domain 2	
tds.dpflux_cPO4z	0	mol/(m²·s)	Dispersive flux, z component	Domain 2	
tds.mflux_cPO4x	-tds.z_cPO4*F_const*cPO4*tds.um_cPO4xx*d(tds.V,x)	mol/(m²·s)	Electrophoretic flux, x component	Domain 2	
tds.mflux_cPO4y	-tds.z_cPO4*F_const*cPO4*tds.um_cPO4yx*d(tds.V,x)	mol/(m²·s)	Electrophoretic flux, y component	Domain 2	
tds.mflux_cPO4z	-tds.z_cPO4*F_const*cPO4*tds.um_cPO4zx*d(tds.V,x)	mol/(m²·s)	Electrophoretic flux, z component	Domain 2	
tds.mfluxMag_cPO4	sqrt(tds.mflux_cPO4x^2+tds.mflux_cPO4y^2+tds.mflux_cPO4z^2)	mol/(m²·s)	Electrophoretic flux magnitude	Domain 2	
tds.tflux_cHx	tds.dflux_cHx+tds.mflux_cHx	mol/(m²·s)	Total flux, x component	Domain 2	+ operation
tds.tflux_cHy	tds.dflux_cHy+tds.mflux_cHy	mol/(m²·s)	Total flux, y component	Domain 2	+ operation
tds.tflux_cHz	tds.dflux_cHz+tds.mflux_cHz	mol/(m²·s)	Total flux, z component	Domain 2	+ operation
tds.dfluxMag_cH	sqrt(tds.dflux_cHx^2+tds.dflux_cHy^2+tds.dflux_cHz^2)	mol/(m²·s)	Diffusive flux magnitude	Domain 2	
tds.tfluxMag_cH	sqrt(tds.tflux_cHx^2+tds.tflux_cHy^2+td	mol/(m²·s)	Total flux magnitude	Domain 2	

Name	Expression	Unit	Description	Selection	Details
	s.tflux_cHz^2)				
tds.dpflux_cHx	0	mol/(m²·s)	Dispersive flux, x component	Domain 2	
tds.dpflux_cHy	0	mol/(m²·s)	Dispersive flux, y component	Domain 2	
tds.dpflux_cHz	0	mol/(m²·s)	Dispersive flux, z component	Domain 2	
tds.mflux_cHx	-tds.z_cH*F_const*cH*tds.um_cHxx*d(tds.V,x)	mol/(m²·s)	Electrophoretic flux, x component	Domain 2	
tds.mflux_cHy	-tds.z_cH*F_const*cH*tds.um_cHyx*d(tds.V,x)	mol/(m²·s)	Electrophoretic flux, y component	Domain 2	
tds.mflux_cHz	-tds.z_cH*F_const*cH*tds.um_cHzx*d(tds.V,x)	mol/(m²·s)	Electrophoretic flux, z component	Domain 2	
tds.mfluxMag_cH	sqrt(tds.mflux_cHx^2+tds.mflux_cHy^2+tds.mflux_cHz^2)	mol/(m²·s)	Electrophoretic flux magnitude	Domain 2	
tds.tflux_cOHx	tds.dflux_cOHx+tds.mflux_cOHx	mol/(m²·s)	Total flux, x component	Domain 2	+ operation
tds.tflux_cOHy	tds.dflux_cOHy+tds.mflux_cOHy	mol/(m²·s)	Total flux, y component	Domain 2	+ operation
tds.tflux_cOHz	tds.dflux_cOHz+tds.mflux_cOHz	mol/(m²·s)	Total flux, z component	Domain 2	+ operation
tds.dfluxMag_cOH	sqrt(tds.dflux_cOHx^2+tds.dflux_cOHy^2+tds.dflux_cOHz^2)	mol/(m²·s)	Diffusive flux magnitude	Domain 2	
tds.tfluxMag_cOH	sqrt(tds.tflux_cOHx^2+tds.tflux_cOHy^2+tds.tflux_cOHz^2)	mol/(m²·s)	Total flux magnitude	Domain 2	
tds.dpflux_cOHx	0	mol/(m²·s)	Dispersive flux, x component	Domain 2	
tds.dpflux_cOHy	0	mol/(m²·s)	Dispersive flux, y component	Domain 2	
tds.dpflux_cOHz	0	mol/(m²·s)	Dispersive flux, z component	Domain 2	
tds.mflux_cOHx	-	mol/(m²·s)	Electrophoretic	Domain 2	

Name	Expression	Unit	Description	Selection	Details
	tds.z_cOH*F_const*cOH*tds.um_cOHxx*d(tds.V,x)		flux, x component		
tds.mflux_cOHy	-tds.z_cOH*F_const*cOH*tds.um_cOHyx*d(tds.V,x)	mol/(m²·s)	Electrophoretic flux, y component	Domain 2	
tds.mflux_cOHz	-tds.z_cOH*F_const*cOH*tds.um_cOHzx*d(tds.V,x)	mol/(m²·s)	Electrophoretic flux, z component	Domain 2	
tds.mfluxMag_cOH	sqrt(tds.mflux_cOHx^2+tds.mflux_cOHy^2+tds.mflux_cOHz^2)	mol/(m²·s)	Electrophoretic flux magnitude	Domain 2	
tds.tflux_cHCO3x	tds.dflux_cHCO3x+tds.mflux_cHCO3x	mol/(m²·s)	Total flux, x component	Domain 2	+ operation
tds.tflux_cHCO3y	tds.dflux_cHCO3y+tds.mflux_cHCO3y	mol/(m²·s)	Total flux, y component	Domain 2	+ operation
tds.tflux_cHCO3z	tds.dflux_cHCO3z+tds.mflux_cHCO3z	mol/(m²·s)	Total flux, z component	Domain 2	+ operation
tds.dfluxMag_cHCO3	sqrt(tds.dflux_cHCO3x^2+tds.dflux_cHCO3y^2+tds.dflux_cHCO3z^2)	mol/(m²·s)	Diffusive flux magnitude	Domain 2	
tds.tfluxMag_cHCO3	sqrt(tds.tflux_cHCO3x^2+tds.tflux_cHCO3y^2+tds.tflux_cHCO3z^2)	mol/(m²·s)	Total flux magnitude	Domain 2	
tds.dpflux_cHCO3x	0	mol/(m²·s)	Dispersive flux, x component	Domain 2	
tds.dpflux_cHCO3y	0	mol/(m²·s)	Dispersive flux, y component	Domain 2	
tds.dpflux_cHCO3z	0	mol/(m²·s)	Dispersive flux, z component	Domain 2	
tds.mflux_cHCO3x	-tds.z_cHCO3*F_const*cHCO3*tds.um_cHCO3xx*d(tds.V,x)	mol/(m²·s)	Electrophoretic flux, x component	Domain 2	
tds.mflux_cHCO3y	-tds.z_cHCO3*F_const*cHCO3*tds.um_cHCO3yx*d(tds.V,x)	mol/(m²·s)	Electrophoretic flux, y component	Domain 2	

Name	Expression	Unit	Description	Selection	Details
tds.mflux_cHCO3z	-tds.z_cHCO3*F_const*cHCO3*tds.um_cHCO3zx*d(tds.V,x)	mol/(m²·s)	Electrophoretic flux, z component	Domain 2	
tds.mfluxMag_cHCO3	sqrt(tds.mflux_cHCO3x^2+tds.mflux_cHCO3y^2+tds.mflux_cHCO3z^2)	mol/(m²·s)	Electrophoretic flux magnitude	Domain 2	
tds.tflux_cCO3x	tds.dflux_cCO3x+tds.mflux_cCO3x	mol/(m²·s)	Total flux, x component	Domain 2	+ operation
tds.tflux_cCO3y	tds.dflux_cCO3y+tds.mflux_cCO3y	mol/(m²·s)	Total flux, y component	Domain 2	+ operation
tds.tflux_cCO3z	tds.dflux_cCO3z+tds.mflux_cCO3z	mol/(m²·s)	Total flux, z component	Domain 2	+ operation
tds.dfluxMag_cCO3	sqrt(tds.dflux_cCO3x^2+tds.dflux_cCO3y^2+tds.dflux_cCO3z^2)	mol/(m²·s)	Diffusive flux magnitude	Domain 2	
tds.tfluxMag_cCO3	sqrt(tds.tflux_cCO3x^2+tds.tflux_cCO3y^2+tds.tflux_cCO3z^2)	mol/(m²·s)	Total flux magnitude	Domain 2	
tds.dpflux_cCO3x	0	mol/(m²·s)	Dispersive flux, x component	Domain 2	
tds.dpflux_cCO3y	0	mol/(m²·s)	Dispersive flux, y component	Domain 2	
tds.dpflux_cCO3z	0	mol/(m²·s)	Dispersive flux, z component	Domain 2	
tds.mflux_cCO3x	-tds.z_cCO3*F_const*cCO3*tds.um_cCO3xx*d(tds.V,x)	mol/(m²·s)	Electrophoretic flux, x component	Domain 2	
tds.mflux_cCO3y	-tds.z_cCO3*F_const*cCO3*tds.um_cCO3yx*d(tds.V,x)	mol/(m²·s)	Electrophoretic flux, y component	Domain 2	
tds.mflux_cCO3z	-tds.z_cCO3*F_const*cCO3*tds.um_cCO3zx*d(tds.V,x)	mol/(m²·s)	Electrophoretic flux, z component	Domain 2	
tds.mfluxMag_cCO3	sqrt(tds.mflux_cCO3x^2+tds.mflux_cCO3y^2+tds.mflux_cC	mol/(m²·s)	Electrophoretic flux magnitude	Domain 2	

Name	Expression	Unit	Description	Selection	Details
	O3z^2)				
tds.dflux_cAscx	-tds.D_cAscxx*cAscx	mol/(m²·s)	Diffusive flux, x component	Domain 2	+ operation
tds.dflux_cAscy	-tds.D_cAscyx*cAscx	mol/(m²·s)	Diffusive flux, y component	Domain 2	+ operation
tds.dflux_cAscz	-tds.D_cAsczx*cAscx	mol/(m²·s)	Diffusive flux, z component	Domain 2	+ operation
tds.grad_cAscx	cAscx	mol/m⁴	Concentration gradient, x component	Domain 2	
tds.grad_cAscy	0	mol/m⁴	Concentration gradient, y component	Domain 2	
tds.grad_cAscz	0	mol/m⁴	Concentration gradient, z component	Domain 2	
tds.dflux_cAscmx	-tds.D_cAscmxx*cAscmx	mol/(m²·s)	Diffusive flux, x component	Domain 2	+ operation
tds.dflux_cAscmy	-tds.D_cAscmyx*cAscmx	mol/(m²·s)	Diffusive flux, y component	Domain 2	+ operation
tds.dflux_cAscmz	-tds.D_cAscmzx*cAscmx	mol/(m²·s)	Diffusive flux, z component	Domain 2	+ operation
tds.grad_cAscmx	cAscmx	mol/m⁴	Concentration gradient, x component	Domain 2	
tds.grad_cAscmy	0	mol/m⁴	Concentration gradient, y component	Domain 2	
tds.grad_cAscmz	0	mol/m⁴	Concentration gradient, z component	Domain 2	
tds.dflux_cCO2x	-tds.D_cCO2xx*cCO2x	mol/(m²·s)	Diffusive flux, x component	Domain 2	+ operation
tds.dflux_cCO2y	-tds.D_cCO2yx*cCO2x	mol/(m²·s)	Diffusive flux, y component	Domain 2	+ operation
tds.dflux_cCO2z	-tds.D_cCO2zx*cCO2	mol/(m²·s)	Diffusive flux, z component	Domain 2	+ operation

Name	Expression	Unit	Description	Selection	Details
	x				
tds.grad_cCO2x	cCO2x	mol/m⁴	Concentration gradient, x component	Domain 2	
tds.grad_cCO2y	0	mol/m⁴	Concentration gradient, y component	Domain 2	
tds.grad_cCO2z	0	mol/m⁴	Concentration gradient, z component	Domain 2	
tds.dflux_cCOx	-tds.D_cCOxx*cCOx	mol/(m²·s)	Diffusive flux, x component	Domain 2	+ operation
tds.dflux_cCOy	-tds.D_cCOyx*cCOx	mol/(m²·s)	Diffusive flux, y component	Domain 2	+ operation
tds.dflux_cCOz	-tds.D_cCOzx*cCOx	mol/(m²·s)	Diffusive flux, z component	Domain 2	+ operation
tds.grad_cCOx	cCOx	mol/m⁴	Concentration gradient, x component	Domain 2	
tds.grad_cCOy	0	mol/m⁴	Concentration gradient, y component	Domain 2	
tds.grad_cCOz	0	mol/m⁴	Concentration gradient, z component	Domain 2	
tds.dflux_cO2x	-tds.D_cO2xx*cO2x	mol/(m²·s)	Diffusive flux, x component	Domain 2	+ operation
tds.dflux_cO2y	-tds.D_cO2yx*cO2x	mol/(m²·s)	Diffusive flux, y component	Domain 2	+ operation
tds.dflux_cO2z	-tds.D_cO2zx*cO2x	mol/(m²·s)	Diffusive flux, z component	Domain 2	+ operation
tds.grad_cO2x	cO2x	mol/m⁴	Concentration gradient, x component	Domain 2	
tds.grad_cO2y	0	mol/m⁴	Concentration gradient, y component	Domain 2	
tds.grad_cO2z	0	mol/m⁴	Concentration gradient, z component	Domain 2	
tds.dflux_cH2Ox	- tds.D_cH2Oxx*cH2O	mol/(m²·s)	Diffusive flux, x component	Domain 2	+ operation

Name	Expression	Unit	Description	Selection	Details
	x				
tds.dflux_cH2Oy	-tds.D_cH2Oyx*cH2Ox	mol/(m²·s)	Diffusive flux, y component	Domain 2	+ operation
tds.dflux_cH2Oz	-tds.D_cH2Ozx*cH2Ox	mol/(m²·s)	Diffusive flux, z component	Domain 2	+ operation
tds.grad_cH2Ox	cH2Ox	mol/m⁴	Concentration gradient, x component	Domain 2	
tds.grad_cH2Oy	0	mol/m⁴	Concentration gradient, y component	Domain 2	
tds.grad_cH2Oz	0	mol/m⁴	Concentration gradient, z component	Domain 2	
tds.dflux_cH2PO4x	-tds.D_cH2PO4xx*cH2PO4x	mol/(m²·s)	Diffusive flux, x component	Domain 2	+ operation
tds.dflux_cH2PO4y	-tds.D_cH2PO4yx*cH2PO4x	mol/(m²·s)	Diffusive flux, y component	Domain 2	+ operation
tds.dflux_cH2PO4z	-tds.D_cH2PO4zx*cH2PO4x	mol/(m²·s)	Diffusive flux, z component	Domain 2	+ operation
tds.grad_cH2PO4x	cH2PO4x	mol/m⁴	Concentration gradient, x component	Domain 2	
tds.grad_cH2PO4y	0	mol/m⁴	Concentration gradient, y component	Domain 2	
tds.grad_cH2PO4z	0	mol/m⁴	Concentration gradient, z component	Domain 2	
tds.dflux_cHPO4x	-tds.D_cHPO4xx*cHPO4x	mol/(m²·s)	Diffusive flux, x component	Domain 2	+ operation
tds.dflux_cHPO4y	-tds.D_cHPO4yx*cHPO4x	mol/(m²·s)	Diffusive flux, y component	Domain 2	+ operation
tds.dflux_cHPO4z	-tds.D_cHPO4zx*cHPO4x	mol/(m²·s)	Diffusive flux, z component	Domain 2	+ operation

Name	Expression	Unit	Description	Selection	Details
tds.grad_cHPO4x	cHPO4x	mol/m⁴	Concentration gradient, x component	Domain 2	
tds.grad_cHPO4y	0	mol/m⁴	Concentration gradient, y component	Domain 2	
tds.grad_cHPO4z	0	mol/m⁴	Concentration gradient, z component	Domain 2	
tds.dflux_cNax	-tds.D_cNaxx*cNax	mol/(m²·s)	Diffusive flux, x component	Domain 2	+ operation
tds.dflux_cNay	-tds.D_cNayx*cNax	mol/(m²·s)	Diffusive flux, y component	Domain 2	+ operation
tds.dflux_cNaz	-tds.D_cNazx*cNax	mol/(m²·s)	Diffusive flux, z component	Domain 2	+ operation
tds.grad_cNax	cNax	mol/m⁴	Concentration gradient, x component	Domain 2	
tds.grad_cNay	0	mol/m⁴	Concentration gradient, y component	Domain 2	
tds.grad_cNaz	0	mol/m⁴	Concentration gradient, z component	Domain 2	
tds.dflux_cClx	-tds.D_cClxx*cClx	mol/(m²·s)	Diffusive flux, x component	Domain 2	+ operation
tds.dflux_cCly	-tds.D_cClyx*cClx	mol/(m²·s)	Diffusive flux, y component	Domain 2	+ operation
tds.dflux_cClz	-tds.D_cClzx*cClx	mol/(m²·s)	Diffusive flux, z component	Domain 2	+ operation
tds.grad_cClx	cClx	mol/m⁴	Concentration gradient, x component	Domain 2	
tds.grad_cCly	0	mol/m⁴	Concentration gradient, y component	Domain 2	
tds.grad_cClz	0	mol/m⁴	Concentration gradient, z component	Domain 2	
tds.dflux_cH3PO4x	-tds.D_cH3PO4xx*cH3PO4x	mol/(m²·s)	Diffusive flux, x component	Domain 2	+ operation

Name	Expression	Unit	Description	Selection	Details
tds.dflux_cH3PO4y	-tds.D_cH3PO4yx*cH3PO4x	mol/(m²·s)	Diffusive flux, y component	Domain 2	+ operation
tds.dflux_cH3PO4z	-tds.D_cH3PO4zx*cH3PO4x	mol/(m²·s)	Diffusive flux, z component	Domain 2	+ operation
tds.grad_cH3PO4x	cH3PO4x	mol/m⁴	Concentration gradient, x component	Domain 2	
tds.grad_cH3PO4y	0	mol/m⁴	Concentration gradient, y component	Domain 2	
tds.grad_cH3PO4z	0	mol/m⁴	Concentration gradient, z component	Domain 2	
tds.dflux_cPO4x	-tds.D_cPO4xx*cPO4x	mol/(m²·s)	Diffusive flux, x component	Domain 2	+ operation
tds.dflux_cPO4y	-tds.D_cPO4yx*cPO4x	mol/(m²·s)	Diffusive flux, y component	Domain 2	+ operation
tds.dflux_cPO4z	-tds.D_cPO4zx*cPO4x	mol/(m²·s)	Diffusive flux, z component	Domain 2	+ operation
tds.grad_cPO4x	cPO4x	mol/m⁴	Concentration gradient, x component	Domain 2	
tds.grad_cPO4y	0	mol/m⁴	Concentration gradient, y component	Domain 2	
tds.grad_cPO4z	0	mol/m⁴	Concentration gradient, z component	Domain 2	
tds.dflux_cHx	-tds.D_cHxx*cHx	mol/(m²·s)	Diffusive flux, x component	Domain 2	+ operation
tds.dflux_cHy	-tds.D_cHyx*cHx	mol/(m²·s)	Diffusive flux, y component	Domain 2	+ operation
tds.dflux_cHz	-tds.D_cHzx*cHx	mol/(m²·s)	Diffusive flux, z component	Domain 2	+ operation
tds.grad_cHx	cHx	mol/m⁴	Concentration gradient, x component	Domain 2	
tds.grad_cHy	0	mol/m⁴	Concentration gradient, y component	Domain 2	

Name	Expression	Unit	Description	Selection	Details
tds.grad_cHz	0	mol/m^4	Concentration gradient, z component	Domain 2	
tds.dflux_cOHx	-tds.D_cOHxx*cOHx	$mol/(m^2 \cdot s)$	Diffusive flux, x component	Domain 2	+ operation
tds.dflux_cOHy	-tds.D_cOHyx*cOHx	$mol/(m^2 \cdot s)$	Diffusive flux, y component	Domain 2	+ operation
tds.dflux_cOHz	-tds.D_cOHzx*cOHx	$mol/(m^2 \cdot s)$	Diffusive flux, z component	Domain 2	+ operation
tds.grad_cOHx	cOHx	mol/m^4	Concentration gradient, x component	Domain 2	
tds.grad_cOHy	0	mol/m^4	Concentration gradient, y component	Domain 2	
tds.grad_cOHz	0	mol/m^4	Concentration gradient, z component	Domain 2	
tds.dflux_cHCO3x	-tds.D_cHCO3xx*cHCO3x	$mol/(m^2 \cdot s)$	Diffusive flux, x component	Domain 2	+ operation
tds.dflux_cHCO3y	-tds.D_cHCO3yx*cHCO3x	$mol/(m^2 \cdot s)$	Diffusive flux, y component	Domain 2	+ operation
tds.dflux_cHCO3z	-tds.D_cHCO3zx*cHCO3x	$mol/(m^2 \cdot s)$	Diffusive flux, z component	Domain 2	+ operation
tds.grad_cHCO3x	cHCO3x	mol/m^4	Concentration gradient, x component	Domain 2	
tds.grad_cHCO3y	0	mol/m^4	Concentration gradient, y component	Domain 2	
tds.grad_cHCO3z	0	mol/m^4	Concentration gradient, z component	Domain 2	
tds.dflux_cCO3x	-tds.D_cCO3xx*cCO3x	$mol/(m^2 \cdot s)$	Diffusive flux, x component	Domain 2	+ operation
tds.dflux_cCO3y	-tds.D_cCO3yx*cCO3x	$mol/(m^2 \cdot s)$	Diffusive flux, y component	Domain 2	+ operation

Name	Expression	Unit	Description	Selection	Details
tds.dflux_cCO3z	-tds.D_cCO3zx*cCO3x	mol/(m²·s)	Diffusive flux, z component	Domain 2	+ operation
tds.grad_cCO3x	cCO3x	mol/m⁴	Concentration gradient, x component	Domain 2	
tds.grad_cCO3y	0	mol/m⁴	Concentration gradient, y component	Domain 2	
tds.grad_cCO3z	0	mol/m⁴	Concentration gradient, z component	Domain 2	
tds.um_cAscxx	tds.D_cAscxx/(R_const*tds.T)	s·mol/kg	Mobility, xx component	Domain 2	
tds.um_cAscyx	tds.D_cAscyx/(R_const*tds.T)	s·mol/kg	Mobility, yx component	Domain 2	
tds.um_cAsczx	tds.D_cAsczx/(R_const*tds.T)	s·mol/kg	Mobility, zx component	Domain 2	
tds.um_cAscxy	tds.D_cAscxy/(R_const*tds.T)	s·mol/kg	Mobility, xy component	Domain 2	
tds.um_cAscyy	tds.D_cAscyy/(R_const*tds.T)	s·mol/kg	Mobility, yy component	Domain 2	
tds.um_cAsczy	tds.D_cAsczy/(R_const*tds.T)	s·mol/kg	Mobility, zy component	Domain 2	
tds.um_cAscxz	tds.D_cAscxz/(R_const*tds.T)	s·mol/kg	Mobility, xz component	Domain 2	
tds.um_cAscyz	tds.D_cAscyz/(R_const*tds.T)	s·mol/kg	Mobility, yz component	Domain 2	
tds.um_cAsczz	tds.D_cAsczz/(R_const*tds.T)	s·mol/kg	Mobility, zz component	Domain 2	
tds.z_cAsc	0	1	Charge number	Domain 2	
tds.um_cAscmxx	tds.D_cAscmxx/(R_const*tds.T)	s·mol/kg	Mobility, xx component	Domain 2	
tds.um_cAscmyx	tds.D_cAscmyx/(R_const*tds.T)	s·mol/kg	Mobility, yx component	Domain 2	
tds.um_cAscmzx	tds.D_cAscmzx/(R_const*tds.T)	s·mol/kg	Mobility, zx component	Domain 2	
tds.um_cAscmxy	tds.D_cAscmxy/(R_const*tds.T)	s·mol/kg	Mobility, xy component	Domain 2	
tds.um_cAscmyy	tds.D_cAscmyy/(R_const*tds.T)	s·mol/kg	Mobility, yy component	Domain 2	

Name	Expression	Unit	Description	Selection	Details
tds.um_cAscmzy	tds.D_cAscmzy/(R_const*tds.T)	s·mol/kg	Mobility, zy component	Domain 2	
tds.um_cAscmxz	tds.D_cAscmxz/(R_const*tds.T)	s·mol/kg	Mobility, xz component	Domain 2	
tds.um_cAscmyz	tds.D_cAscmyz/(R_const*tds.T)	s·mol/kg	Mobility, yz component	Domain 2	
tds.um_cAscmzz	tds.D_cAscmzz/(R_const*tds.T)	s·mol/kg	Mobility, zz component	Domain 2	
tds.z_cAscm	-1	1	Charge number	Domain 2	
tds.um_cCO2xx	tds.D_cCO2xx/(R_const*tds.T)	s·mol/kg	Mobility, xx component	Domain 2	
tds.um_cCO2yx	tds.D_cCO2yx/(R_const*tds.T)	s·mol/kg	Mobility, yx component	Domain 2	
tds.um_cCO2zx	tds.D_cCO2zx/(R_const*tds.T)	s·mol/kg	Mobility, zx component	Domain 2	
tds.um_cCO2xy	tds.D_cCO2xy/(R_const*tds.T)	s·mol/kg	Mobility, xy component	Domain 2	
tds.um_cCO2yy	tds.D_cCO2yy/(R_const*tds.T)	s·mol/kg	Mobility, yy component	Domain 2	
tds.um_cCO2zy	tds.D_cCO2zy/(R_const*tds.T)	s·mol/kg	Mobility, zy component	Domain 2	
tds.um_cCO2xz	tds.D_cCO2xz/(R_const*tds.T)	s·mol/kg	Mobility, xz component	Domain 2	
tds.um_cCO2yz	tds.D_cCO2yz/(R_const*tds.T)	s·mol/kg	Mobility, yz component	Domain 2	
tds.um_cCO2zz	tds.D_cCO2zz/(R_const*tds.T)	s·mol/kg	Mobility, zz component	Domain 2	
tds.z_cCO2	0	1	Charge number	Domain 2	
tds.um_cCOxx	tds.D_cCOxx/(R_const*tds.T)	s·mol/kg	Mobility, xx component	Domain 2	
tds.um_cCOyx	tds.D_cCOyx/(R_const*tds.T)	s·mol/kg	Mobility, yx component	Domain 2	
tds.um_cCOzx	tds.D_cCOzx/(R_const*tds.T)	s·mol/kg	Mobility, zx component	Domain 2	
tds.um_cCOxy	tds.D_cCOxy/(R_const*tds.T)	s·mol/kg	Mobility, xy component	Domain 2	
tds.um_cCOyy	tds.D_cCOyy/(R_const*tds.T)	s·mol/kg	Mobility, yy component	Domain 2	
tds.um_cCOzy	tds.D_cCOzy/(R_const*tds.T)	s·mol/kg	Mobility, zy component	Domain 2	

Name	Expression	Unit	Description	Selection	Details
tds.um_cCOxz	tds.D_cCOxz/(R_const*tds.T)	s·mol/kg	Mobility, xz component	Domain 2	
tds.um_cCOyz	tds.D_cCOyz/(R_const*tds.T)	s·mol/kg	Mobility, yz component	Domain 2	
tds.um_cCOzz	tds.D_cCOzz/(R_const*tds.T)	s·mol/kg	Mobility, zz component	Domain 2	
tds.z_cCO	0	1	Charge number	Domain 2	
tds.um_cO2xx	tds.D_cO2xx/(R_const*tds.T)	s·mol/kg	Mobility, xx component	Domain 2	
tds.um_cO2yx	tds.D_cO2yx/(R_const*tds.T)	s·mol/kg	Mobility, yx component	Domain 2	
tds.um_cO2zx	tds.D_cO2zx/(R_const*tds.T)	s·mol/kg	Mobility, zx component	Domain 2	
tds.um_cO2xy	tds.D_cO2xy/(R_const*tds.T)	s·mol/kg	Mobility, xy component	Domain 2	
tds.um_cO2yy	tds.D_cO2yy/(R_const*tds.T)	s·mol/kg	Mobility, yy component	Domain 2	
tds.um_cO2zy	tds.D_cO2zy/(R_const*tds.T)	s·mol/kg	Mobility, zy component	Domain 2	
tds.um_cO2xz	tds.D_cO2xz/(R_const*tds.T)	s·mol/kg	Mobility, xz component	Domain 2	
tds.um_cO2yz	tds.D_cO2yz/(R_const*tds.T)	s·mol/kg	Mobility, yz component	Domain 2	
tds.um_cO2zz	tds.D_cO2zz/(R_const*tds.T)	s·mol/kg	Mobility, zz component	Domain 2	
tds.z_cO2	0	1	Charge number	Domain 2	
tds.um_cH2Oxx	tds.D_cH2Oxx/(R_const*tds.T)	s·mol/kg	Mobility, xx component	Domain 2	
tds.um_cH2Oyx	tds.D_cH2Oyx/(R_const*tds.T)	s·mol/kg	Mobility, yx component	Domain 2	
tds.um_cH2Ozx	tds.D_cH2Ozx/(R_const*tds.T)	s·mol/kg	Mobility, zx component	Domain 2	
tds.um_cH2Oxy	tds.D_cH2Oxy/(R_const*tds.T)	s·mol/kg	Mobility, xy component	Domain 2	
tds.um_cH2Oyy	tds.D_cH2Oyy/(R_const*tds.T)	s·mol/kg	Mobility, yy component	Domain 2	
tds.um_cH2Ozy	tds.D_cH2Ozy/(R_const*tds.T)	s·mol/kg	Mobility, zy component	Domain 2	
tds.um_cH2Oxz	tds.D_cH2Oxz/(R_const*tds.T)	s·mol/kg	Mobility, xz component	Domain 2	

Name	Expression	Unit	Description	Selection	Details
tds.um_cH2Oyz	tds.D_cH2Oyz/(R_const*tds.T)	s·mol/kg	Mobility, yz component	Domain 2	
tds.um_cH2Ozz	tds.D_cH2Ozz/(R_const*tds.T)	s·mol/kg	Mobility, zz component	Domain 2	
tds.z_cH2O	0	1	Charge number	Domain 2	
tds.um_cH2PO4xx	tds.D_cH2PO4xx/(R_const*tds.T)	s·mol/kg	Mobility, xx component	Domain 2	
tds.um_cH2PO4yx	tds.D_cH2PO4yx/(R_const*tds.T)	s·mol/kg	Mobility, yx component	Domain 2	
tds.um_cH2PO4zx	tds.D_cH2PO4zx/(R_const*tds.T)	s·mol/kg	Mobility, zx component	Domain 2	
tds.um_cH2PO4xy	tds.D_cH2PO4xy/(R_const*tds.T)	s·mol/kg	Mobility, xy component	Domain 2	
tds.um_cH2PO4yy	tds.D_cH2PO4yy/(R_const*tds.T)	s·mol/kg	Mobility, yy component	Domain 2	
tds.um_cH2PO4zy	tds.D_cH2PO4zy/(R_const*tds.T)	s·mol/kg	Mobility, zy component	Domain 2	
tds.um_cH2PO4xz	tds.D_cH2PO4xz/(R_const*tds.T)	s·mol/kg	Mobility, xz component	Domain 2	
tds.um_cH2PO4yz	tds.D_cH2PO4yz/(R_const*tds.T)	s·mol/kg	Mobility, yz component	Domain 2	
tds.um_cH2PO4zz	tds.D_cH2PO4zz/(R_const*tds.T)	s·mol/kg	Mobility, zz component	Domain 2	
tds.z_cH2PO4	-1	1	Charge number	Domain 2	
tds.um_cHPO4xx	tds.D_cHPO4xx/(R_const*tds.T)	s·mol/kg	Mobility, xx component	Domain 2	
tds.um_cHPO4yx	tds.D_cHPO4yx/(R_const*tds.T)	s·mol/kg	Mobility, yx component	Domain 2	
tds.um_cHPO4zx	tds.D_cHPO4zx/(R_const*tds.T)	s·mol/kg	Mobility, zx component	Domain 2	
tds.um_cHPO4xy	tds.D_cHPO4xy/(R_const*tds.T)	s·mol/kg	Mobility, xy component	Domain 2	
tds.um_cHPO4yy	tds.D_cHPO4yy/(R_const*tds.T)	s·mol/kg	Mobility, yy component	Domain 2	
tds.um_cHPO4zy	tds.D_cHPO4zy/(R_const*tds.T)	s·mol/kg	Mobility, zy component	Domain 2	
tds.um_cHPO4xz	tds.D_cHPO4xz/(R_const*tds.T)	s·mol/kg	Mobility, xz component	Domain 2	
tds.um_cHPO4yz	tds.D_cHPO4yz/(R_const*tds.T)	s·mol/kg	Mobility, yz component	Domain 2	

Name	Expression	Unit	Description	Selection	Details
tds.um_cHPO4zz	tds.D_cHPO4zz/(R_const*tds.T)	s·mol/kg	Mobility, zz component	Domain 2	
tds.z_cHPO4	-2	1	Charge number	Domain 2	
tds.um_cNaxx	tds.D_cNaxx/(R_const*tds.T)	s·mol/kg	Mobility, xx component	Domain 2	
tds.um_cNayx	tds.D_cNayx/(R_const*tds.T)	s·mol/kg	Mobility, yx component	Domain 2	
tds.um_cNazx	tds.D_cNazx/(R_const*tds.T)	s·mol/kg	Mobility, zx component	Domain 2	
tds.um_cNaxy	tds.D_cNaxy/(R_const*tds.T)	s·mol/kg	Mobility, xy component	Domain 2	
tds.um_cNayy	tds.D_cNayy/(R_const*tds.T)	s·mol/kg	Mobility, yy component	Domain 2	
tds.um_cNazy	tds.D_cNazy/(R_const*tds.T)	s·mol/kg	Mobility, zy component	Domain 2	
tds.um_cNaxz	tds.D_cNaxz/(R_const*tds.T)	s·mol/kg	Mobility, xz component	Domain 2	
tds.um_cNayz	tds.D_cNayz/(R_const*tds.T)	s·mol/kg	Mobility, yz component	Domain 2	
tds.um_cNazz	tds.D_cNazz/(R_const*tds.T)	s·mol/kg	Mobility, zz component	Domain 2	
tds.z_cNa	1	1	Charge number	Domain 2	
tds.um_cClxx	tds.D_cClxx/(R_const*tds.T)	s·mol/kg	Mobility, xx component	Domain 2	
tds.um_cClyx	tds.D_cClyx/(R_const*tds.T)	s·mol/kg	Mobility, yx component	Domain 2	
tds.um_cClzx	tds.D_cClzx/(R_const*tds.T)	s·mol/kg	Mobility, zx component	Domain 2	
tds.um_cClxy	tds.D_cClxy/(R_const*tds.T)	s·mol/kg	Mobility, xy component	Domain 2	
tds.um_cClyy	tds.D_cClyy/(R_const*tds.T)	s·mol/kg	Mobility, yy component	Domain 2	
tds.um_cClzy	tds.D_cClzy/(R_const*tds.T)	s·mol/kg	Mobility, zy component	Domain 2	
tds.um_cClxz	tds.D_cClxz/(R_const*tds.T)	s·mol/kg	Mobility, xz component	Domain 2	
tds.um_cClyz	tds.D_cClyz/(R_const*tds.T)	s·mol/kg	Mobility, yz component	Domain 2	
tds.um_cClzz	tds.D_cClzz/(R_const*tds.T)	s·mol/kg	Mobility, zz component	Domain 2	

Name	Expression	Unit	Description	Selection	Details
tds.z_cCl	-1	1	Charge number	Domain 2	
tds.um_cH3PO4xx	tds.D_cH3PO4xx/(R_const*tds.T)	s·mol/kg	Mobility, xx component	Domain 2	
tds.um_cH3PO4yx	tds.D_cH3PO4yx/(R_const*tds.T)	s·mol/kg	Mobility, yx component	Domain 2	
tds.um_cH3PO4zx	tds.D_cH3PO4zx/(R_const*tds.T)	s·mol/kg	Mobility, zx component	Domain 2	
tds.um_cH3PO4xy	tds.D_cH3PO4xy/(R_const*tds.T)	s·mol/kg	Mobility, xy component	Domain 2	
tds.um_cH3PO4yy	tds.D_cH3PO4yy/(R_const*tds.T)	s·mol/kg	Mobility, yy component	Domain 2	
tds.um_cH3PO4zy	tds.D_cH3PO4zy/(R_const*tds.T)	s·mol/kg	Mobility, zy component	Domain 2	
tds.um_cH3PO4xz	tds.D_cH3PO4xz/(R_const*tds.T)	s·mol/kg	Mobility, xz component	Domain 2	
tds.um_cH3PO4yz	tds.D_cH3PO4yz/(R_const*tds.T)	s·mol/kg	Mobility, yz component	Domain 2	
tds.um_cH3PO4zz	tds.D_cH3PO4zz/(R_const*tds.T)	s·mol/kg	Mobility, zz component	Domain 2	
tds.z_cH3PO4	0	1	Charge number	Domain 2	
tds.um_cPO4xx	tds.D_cPO4xx/(R_const*tds.T)	s·mol/kg	Mobility, xx component	Domain 2	
tds.um_cPO4yx	tds.D_cPO4yx/(R_const*tds.T)	s·mol/kg	Mobility, yx component	Domain 2	
tds.um_cPO4zx	tds.D_cPO4zx/(R_const*tds.T)	s·mol/kg	Mobility, zx component	Domain 2	
tds.um_cPO4xy	tds.D_cPO4xy/(R_const*tds.T)	s·mol/kg	Mobility, xy component	Domain 2	
tds.um_cPO4yy	tds.D_cPO4yy/(R_const*tds.T)	s·mol/kg	Mobility, yy component	Domain 2	
tds.um_cPO4zy	tds.D_cPO4zy/(R_const*tds.T)	s·mol/kg	Mobility, zy component	Domain 2	
tds.um_cPO4xz	tds.D_cPO4xz/(R_const*tds.T)	s·mol/kg	Mobility, xz component	Domain 2	
tds.um_cPO4yz	tds.D_cPO4yz/(R_const*tds.T)	s·mol/kg	Mobility, yz component	Domain 2	
tds.um_cPO4zz	tds.D_cPO4zz/(R_const*tds.T)	s·mol/kg	Mobility, zz component	Domain 2	
tds.z_cPO4	-3	1	Charge number	Domain 2	
tds.um_cHxx	tds.D_cHxx/(R_const	s·mol/kg	Mobility, xx	Domain 2	

Name	Expression	Unit	Description	Selection	Details
	*tds.T)		component		
tds.um_cHyx	tds.D_cHyx/(R_const *tds.T)	s·mol/kg	Mobility, yx component	Domain 2	
tds.um_cHzx	tds.D_cHzx/(R_const *tds.T)	s·mol/kg	Mobility, zx component	Domain 2	
tds.um_cHxy	tds.D_cHxy/(R_const *tds.T)	s·mol/kg	Mobility, xy component	Domain 2	
tds.um_cHyy	tds.D_cHyy/(R_const *tds.T)	s·mol/kg	Mobility, yy component	Domain 2	
tds.um_cHzy	tds.D_cHzy/(R_const *tds.T)	s·mol/kg	Mobility, zy component	Domain 2	
tds.um_cHxz	tds.D_cHxz/(R_const *tds.T)	s·mol/kg	Mobility, xz component	Domain 2	
tds.um_cHyz	tds.D_cHyz/(R_const *tds.T)	s·mol/kg	Mobility, yz component	Domain 2	
tds.um_cHzz	tds.D_cHzz/(R_const *tds.T)	s·mol/kg	Mobility, zz component	Domain 2	
tds.z_cH	1	1	Charge number	Domain 2	
tds.um_cOHxx	tds.D_cOHxx/(R_const*tds.T)	s·mol/kg	Mobility, xx component	Domain 2	
tds.um_cOHyx	tds.D_cOHyx/(R_const*tds.T)	s·mol/kg	Mobility, yx component	Domain 2	
tds.um_cOHzx	tds.D_cOHzx/(R_const*tds.T)	s·mol/kg	Mobility, zx component	Domain 2	
tds.um_cOHxy	tds.D_cOHxy/(R_const*tds.T)	s·mol/kg	Mobility, xy component	Domain 2	
tds.um_cOHyy	tds.D_cOHyy/(R_const*tds.T)	s·mol/kg	Mobility, yy component	Domain 2	
tds.um_cOHzy	tds.D_cOHzy/(R_const*tds.T)	s·mol/kg	Mobility, zy component	Domain 2	
tds.um_cOHxz	tds.D_cOHxz/(R_const*tds.T)	s·mol/kg	Mobility, xz component	Domain 2	
tds.um_cOHyz	tds.D_cOHyz/(R_const*tds.T)	s·mol/kg	Mobility, yz component	Domain 2	
tds.um_cOHzz	tds.D_cOHzz/(R_const*tds.T)	s·mol/kg	Mobility, zz component	Domain 2	
tds.z_cOH	-1	1	Charge number	Domain 2	
tds.um_cHCO3xx	tds.D_cHCO3xx/(R_const*tds.T)	s·mol/kg	Mobility, xx component	Domain 2	
tds.um_cHCO3yx	tds.D_cHCO3yx/(R_c	s·mol/kg	Mobility, yx	Domain 2	

Name	Expression	Unit	Description	Selection	Details
	onst*tds.T)		component		
tds.um_cHCO3zx	tds.D_cHCO3zx/(R_const*tds.T)	s·mol/kg	Mobility, zx component	Domain 2	
tds.um_cHCO3xy	tds.D_cHCO3xy/(R_const*tds.T)	s·mol/kg	Mobility, xy component	Domain 2	
tds.um_cHCO3yy	tds.D_cHCO3yy/(R_const*tds.T)	s·mol/kg	Mobility, yy component	Domain 2	
tds.um_cHCO3zy	tds.D_cHCO3zy/(R_const*tds.T)	s·mol/kg	Mobility, zy component	Domain 2	
tds.um_cHCO3xz	tds.D_cHCO3xz/(R_const*tds.T)	s·mol/kg	Mobility, xz component	Domain 2	
tds.um_cHCO3yz	tds.D_cHCO3yz/(R_const*tds.T)	s·mol/kg	Mobility, yz component	Domain 2	
tds.um_cHCO3zz	tds.D_cHCO3zz/(R_const*tds.T)	s·mol/kg	Mobility, zz component	Domain 2	
tds.z_cHCO3	-1	1	Charge number	Domain 2	
tds.um_cCO3xx	tds.D_cCO3xx/(R_const*tds.T)	s·mol/kg	Mobility, xx component	Domain 2	
tds.um_cCO3yx	tds.D_cCO3yx/(R_const*tds.T)	s·mol/kg	Mobility, yx component	Domain 2	
tds.um_cCO3zx	tds.D_cCO3zx/(R_const*tds.T)	s·mol/kg	Mobility, zx component	Domain 2	
tds.um_cCO3xy	tds.D_cCO3xy/(R_const*tds.T)	s·mol/kg	Mobility, xy component	Domain 2	
tds.um_cCO3yy	tds.D_cCO3yy/(R_const*tds.T)	s·mol/kg	Mobility, yy component	Domain 2	
tds.um_cCO3zy	tds.D_cCO3zy/(R_const*tds.T)	s·mol/kg	Mobility, zy component	Domain 2	
tds.um_cCO3xz	tds.D_cCO3xz/(R_const*tds.T)	s·mol/kg	Mobility, xz component	Domain 2	
tds.um_cCO3yz	tds.D_cCO3yz/(R_const*tds.T)	s·mol/kg	Mobility, yz component	Domain 2	
tds.um_cCO3zz	tds.D_cCO3zz/(R_const*tds.T)	s·mol/kg	Mobility, zz component	Domain 2	
tds.z_cCO3	-2	1	Charge number	Domain 2	
tds.V	model.input.V	V	Electric potential	Domain 2	Meta
tds.T	tds.cdm1.minput_temperature	K	Temperature	Domain 2	
tds.bndFlux_cAsc	-uflux_spatial(cAsc)/t	mol/(m²·s)	Boundary flux	Boundary 2	Meta

Name	Expression	Unit	Description	Selection	Details
	ds.d				
tds.bndFlux_cAsc	-dflux_spatial(cAsc)/tds.d	mol/(m²·s)	Boundary flux	Boundary 3	Meta
tds.bndFlux_cAscm	-uflux_spatial(cAscm)/tds.d	mol/(m²·s)	Boundary flux	Boundary 2	Meta
tds.bndFlux_cAscm	-dflux_spatial(cAscm)/tds.d	mol/(m²·s)	Boundary flux	Boundary 3	Meta
tds.bndFlux_cCO2	-uflux_spatial(cCO2)/tds.d	mol/(m²·s)	Boundary flux	Boundary 2	Meta
tds.bndFlux_cCO2	-dflux_spatial(cCO2)/tds.d	mol/(m²·s)	Boundary flux	Boundary 3	Meta
tds.bndFlux_cCO	-uflux_spatial(cCO)/tds.d	mol/(m²·s)	Boundary flux	Boundary 2	Meta
tds.bndFlux_cCO	-dflux_spatial(cCO)/tds.d	mol/(m²·s)	Boundary flux	Boundary 3	Meta
tds.bndFlux_cO2	-uflux_spatial(cO2)/tds.d	mol/(m²·s)	Boundary flux	Boundary 2	Meta
tds.bndFlux_cO2	-dflux_spatial(cO2)/tds.d	mol/(m²·s)	Boundary flux	Boundary 3	Meta
tds.bndFlux_cH2O	-uflux_spatial(cH2O)/tds.d	mol/(m²·s)	Boundary flux	Boundary 2	Meta
tds.bndFlux_cH2O	-dflux_spatial(cH2O)/tds.d	mol/(m²·s)	Boundary flux	Boundary 3	Meta
tds.bndFlux_cH2PO4	-uflux_spatial(cH2PO4)/tds.d	mol/(m²·s)	Boundary flux	Boundary 2	Meta
tds.bndFlux_cH2PO4	-dflux_spatial(cH2PO4)/tds.d	mol/(m²·s)	Boundary flux	Boundary 3	Meta
tds.bndFlux_cHPO4	-uflux_spatial(cHPO4)/tds.d	mol/(m²·s)	Boundary flux	Boundary 2	Meta

Name	Expression	Unit	Description	Selection	Details
tds.bndFlux_cHPO4	-dflux_spatial(cHPO4)/tds.d	mol/(m²·s)	Boundary flux	Boundary 3	Meta
tds.bndFlux_cNa	-uflux_spatial(cNa)/tds.d	mol/(m²·s)	Boundary flux	Boundary 2	Meta
tds.bndFlux_cNa	-dflux_spatial(cNa)/tds.d	mol/(m²·s)	Boundary flux	Boundary 3	Meta
tds.bndFlux_cCl	-uflux_spatial(cCl)/tds.d	mol/(m²·s)	Boundary flux	Boundary 2	Meta
tds.bndFlux_cCl	-dflux_spatial(cCl)/tds.d	mol/(m²·s)	Boundary flux	Boundary 3	Meta
tds.bndFlux_cH3PO4	-uflux_spatial(cH3PO4)/tds.d	mol/(m²·s)	Boundary flux	Boundary 2	Meta
tds.bndFlux_cH3PO4	-dflux_spatial(cH3PO4)/tds.d	mol/(m²·s)	Boundary flux	Boundary 3	Meta
tds.bndFlux_cPO4	-uflux_spatial(cPO4)/tds.d	mol/(m²·s)	Boundary flux	Boundary 2	Meta
tds.bndFlux_cPO4	-dflux_spatial(cPO4)/tds.d	mol/(m²·s)	Boundary flux	Boundary 3	Meta
tds.bndFlux_cH	-uflux_spatial(cH)/tds.d	mol/(m²·s)	Boundary flux	Boundary 2	Meta
tds.bndFlux_cH	-dflux_spatial(cH)/tds.d	mol/(m²·s)	Boundary flux	Boundary 3	Meta
tds.bndFlux_cOH	-uflux_spatial(cOH)/tds.d	mol/(m²·s)	Boundary flux	Boundary 2	Meta
tds.bndFlux_cOH	-dflux_spatial(cOH)/tds.d	mol/(m²·s)	Boundary flux	Boundary 3	Meta
tds.bndFlux_cHCO3	-uflux_spatial(cHCO3)/tds.d	mol/(m²·s)	Boundary flux	Boundary 2	Meta
tds.bndFlux_cHCO3	-	mol/(m²·s)	Boundary flux	Boundary 3	Meta

Name	Expression	Unit	Description	Selection	Details
	dflux_spatial(cHCO3)/tds.d				
tds.bndFlux_cCO3	-uflux_spatial(cCO3)/tds.d	mol/(m²·s)	Boundary flux	Boundary 2	Meta
tds.bndFlux_cCO3	-dflux_spatial(cCO3)/tds.d	mol/(m²·s)	Boundary flux	Boundary 3	Meta
tds.Res_cAsc	d(cAsc,t)+d(-cAsc*tds.z_cAsc*tds.um_cAscxx*F_const*d(tds.V,x),x)-tds.R_cAsc	mol/(m³·s)	Equation residual	Domain 2	
tds.Rlin_cAsc	0		Linear source term coefficient	Domain 2	
tds.Res_cAscm	d(cAscm,t)+d(-cAscm*tds.z_cAscm*tds.um_cAscmxx*F_const*d(tds.V,x),x)-tds.R_cAscm	mol/(m³·s)	Equation residual	Domain 2	
tds.Rlin_cAscm	0		Linear source term coefficient	Domain 2	
tds.Res_cCO2	d(cCO2,t)+d(-cCO2*tds.z_cCO2*tds.um_cCO2xx*F_const*d(tds.V,x),x)-tds.R_cCO2	mol/(m³·s)	Equation residual	Domain 2	
tds.Rlin_cCO2	0		Linear source term coefficient	Domain 2	
tds.Res_cCO	d(cCO,t)+d(-cCO*tds.z_cCO*tds.um_cCOxx*F_const*d(tds.V,x),x)-tds.R_cCO	mol/(m³·s)	Equation residual	Domain 2	
tds.Rlin_cCO	0		Linear source term coefficient	Domain 2	
tds.Res_cO2	d(cO2,t)+d(-cO2*tds.z_cO2*tds.um_cO2xx*F_const*d(tds.V,x),x)-tds.R_cO2	mol/(m³·s)	Equation residual	Domain 2	
tds.Rlin_cO2	0		Linear source term coefficient	Domain 2	
tds.Res_cH2O	d(cH2O,t)+d(-cH2O*tds.z_cH2O*td	mol/(m³·s)	Equation residual	Domain 2	

Name	Expression	Unit	Description	Selection	Details
	s.um_cH2Oxx*F_const*d(tds.V,x),x)-tds.R_cH2O				
tds.Rlin_cH2O	0		Linear source term coefficient	Domain 2	
tds.Res_cH2PO4	d(cH2PO4,t)+d(-cH2PO4*tds.z_cH2PO4*tds.um_cH2PO4xx*F_const*d(tds.V,x),x)-tds.R_cH2PO4	mol/(m^3·s)	Equation residual	Domain 2	
tds.Rlin_cH2PO4	0		Linear source term coefficient	Domain 2	
tds.Res_cHPO4	d(cHPO4,t)+d(-cHPO4*tds.z_cHPO4*tds.um_cHPO4xx*F_const*d(tds.V,x),x)-tds.R_cHPO4	mol/(m^3·s)	Equation residual	Domain 2	
tds.Rlin_cHPO4	0		Linear source term coefficient	Domain 2	
tds.Res_cNa	d(cNa,t)+d(-cNa*tds.z_cNa*tds.um_cNaxx*F_const*d(tds.V,x),x)-tds.R_cNa	mol/(m^3·s)	Equation residual	Domain 2	
tds.Rlin_cNa	0		Linear source term coefficient	Domain 2	
tds.Res_cCl	d(cCl,t)+d(-cCl*tds.z_cCl*tds.um_cClxx*F_const*d(tds.V,x),x)-tds.R_cCl	mol/(m^3·s)	Equation residual	Domain 2	
tds.Rlin_cCl	0		Linear source term coefficient	Domain 2	
tds.Res_cH3PO4	d(cH3PO4,t)+d(-cH3PO4*tds.z_cH3PO4*tds.um_cH3PO4xx*F_const*d(tds.V,x),x)-tds.R_cH3PO4	mol/(m^3·s)	Equation residual	Domain 2	
tds.Rlin_cH3PO4	0		Linear source term coefficient	Domain 2	
tds.Res_cPO4	d(cPO4,t)+d(-cPO4*tds.z_cPO4*tds.um_cPO4xx*F_const*d(tds.V,x),x)-tds.R_cPO4	mol/(m^3·s)	Equation residual	Domain 2	

Name	Expression	Unit	Description	Selection	Details
tds.Rlin_cPO4	0		Linear source term coefficient	Domain 2	
tds.Res_cH	d(cH,t)+d(-cH*tds.z_cH*tds.um_cHxx*F_const*d(tds.V,x),x)-tds.R_cH	mol/(m³·s)	Equation residual	Domain 2	
tds.Rlin_cH	0		Linear source term coefficient	Domain 2	
tds.Res_cOH	d(cOH,t)+d(-cOH*tds.z_cOH*tds.um_cOHxx*F_const*d(tds.V,x),x)-tds.R_cOH	mol/(m³·s)	Equation residual	Domain 2	
tds.Rlin_cOH	0		Linear source term coefficient	Domain 2	
tds.Res_cHCO3	d(cHCO3,t)+d(-cHCO3*tds.z_cHCO3*tds.um_cHCO3xx*F_const*d(tds.V,x),x)-tds.R_cHCO3	mol/(m³·s)	Equation residual	Domain 2	
tds.Rlin_cHCO3	0		Linear source term coefficient	Domain 2	
tds.Res_cCO3	d(cCO3,t)+d(-cCO3*tds.z_cCO3*tds.um_cCO3xx*F_const*d(tds.V,x),x)-tds.R_cCO3	mol/(m³·s)	Equation residual	Domain 2	
tds.Rlin_cCO3	0		Linear source term coefficient	Domain 2	

Shape functions

Name	Shape function	Unit	Description	Shape frame	Selection
cAsc	Lagrange (Linear)	mol/m³	Concentration	Spatial	Domain 2
cAscm	Lagrange (Linear)	mol/m³	Concentration	Spatial	Domain 2
cCO2	Lagrange (Linear)	mol/m³	Concentration	Spatial	Domain 2
cCO	Lagrange (Linear)	mol/m³	Concentration	Spatial	Domain 2
cO2	Lagrange (Linear)	mol/m³	Concentration	Spatial	Domain 2
cH2O	Lagrange (Linear)	mol/m³	Concentration	Spatial	Domain 2
cH2PO4	Lagrange (Linear)	mol/m³	Concentration	Spatial	Domain 2
cHPO4	Lagrange (Linear)	mol/m³	Concentration	Spatial	Domain 2
cNa	Lagrange (Linear)	mol/m³	Concentration	Spatial	Domain 2

Name	Shape function	Unit	Description	Shape frame	Selection
cCl	Lagrange (Linear)	mol/m^3	Concentration	Spatial	Domain 2
cH3PO4	Lagrange (Linear)	mol/m^3	Concentration	Spatial	Domain 2
cPO4	Lagrange (Linear)	mol/m^3	Concentration	Spatial	Domain 2
cH	Lagrange (Linear)	mol/m^3	Concentration	Spatial	Domain 2
cOH	Lagrange (Linear)	mol/m^3	Concentration	Spatial	Domain 2
cHCO3	Lagrange (Linear)	mol/m^3	Concentration	Spatial	Domain 2
cCO3	Lagrange (Linear)	mol/m^3	Concentration	Spatial	Domain 2

Weak Expressions

Weak expression	Integration order	Integration frame	Selection
(-cAsct*test(cAsc)+tds.dflux_cAscx*test(cAscx))*tds.d	2	Spatial	Domain 2
(-cAscmt*test(cAscm)+tds.dflux_cAscmx*test(cAscmx))*tds.d	2	Spatial	Domain 2
(-cCO2t*test(cCO2)+tds.dflux_cCO2x*test(cCO2x))*tds.d	2	Spatial	Domain 2
(-cCOt*test(cCO)+tds.dflux_cCOx*test(cCOx))*tds.d	2	Spatial	Domain 2
(-cO2t*test(cO2)+tds.dflux_cO2x*test(cO2x))*tds.d	2	Spatial	Domain 2
(-cH2Ot*test(cH2O)+tds.dflux_cH2Ox*test(cH2Ox))*tds.d	2	Spatial	Domain 2
(-cH2PO4t*test(cH2PO4)+tds.dflux_cH2PO4x*test(cH2PO4x))*tds.d	2	Spatial	Domain 2
(-cHPO4t*test(cHPO4)+tds.dflux_cHPO4x*test(cHPO4x))*tds.d	2	Spatial	Domain 2
(-cNat*test(cNa)+tds.dflux_cNax*test(cNax))*tds.d	2	Spatial	Domain 2
(-cClt*test(cCl)+tds.dflux_cClx*test(cClx))*tds.d	2	Spatial	Domain 2
(-	2	Spatial	Domain 2

Weak expression	Integration order	Integration frame	Selection
cH3PO4t*test(cH3PO4)+tds.dflux_cH3PO4x*test(cH3PO4x))*tds.d			
(-cPO4t*test(cPO4)+tds.dflux_cPO4x*test(cPO4x))*tds.d	2	Spatial	Domain 2
(-cHt*test(cH)+tds.dflux_cHx*test(cHx))*tds.d	2	Spatial	Domain 2
(-cOHt*test(cOH)+tds.dflux_cOHx*test(cOHx))*tds.d	2	Spatial	Domain 2
(-cHCO3t*test(cHCO3)+tds.dflux_cHCO3x*test(cHCO3x))*tds.d	2	Spatial	Domain 2
(-cCO3t*test(cCO3)+tds.dflux_cCO3x*test(cCO3x))*tds.d	2	Spatial	Domain 2
-tds.z_cAsc*F_const*cAsc*tds.um_cAscxx*d(tds.V,x)*test(cAscx)*tds.d	2	Spatial	Domain 2
-tds.z_cAscm*F_const*cAscm*tds.um_cAscmxx*d(tds.V,x)*test(cAscmx)*tds.d	2	Spatial	Domain 2
-tds.z_cCO2*F_const*cCO2*tds.um_cCO2xx*d(tds.V,x)*test(cCO2x)*tds.d	2	Spatial	Domain 2
-tds.z_cCO*F_const*cCO*tds.um_cCOxx*d(tds.V,x)*test(cCOx)*tds.d	2	Spatial	Domain 2
-tds.z_cO2*F_const*cO2*tds.um_cO2xx*d(tds.V,x)*test(cO2x)*tds.d	2	Spatial	Domain 2
-tds.z_cH2O*F_const*cH2O*tds.um_cH2Oxx*d(tds.V,x)*test(cH2Ox)*tds.d	2	Spatial	Domain 2
-tds.z_cH2PO4*F_const*cH2PO4*tds.um_cH2PO4xx*d(tds.V,x)*test(cH2PO4x)*tds.d	2	Spatial	Domain 2
-tds.z_cHPO4*F_const*cHPO4*tds.um_cHPO4xx*d(tds.V,x)*test(cHPO4x)*tds.d	2	Spatial	Domain 2
-tds.z_cNa*F_const*cNa*tds.um_cNaxx*d(t	2	Spatial	Domain 2

Weak expression	Integration order	Integration frame	Selection
ds.V,x)*test(cNax)*tds.d			
-tds.z_cCl*F_const*cCl*tds.um_cClxx*d(tds.V,x)*test(cClx)*tds.d	2	Spatial	Domain 2
-tds.z_cH3PO4*F_const*cH3PO4*tds.um_cH3PO4xx*d(tds.V,x)*test(cH3PO4x)*tds.d	2	Spatial	Domain 2
-tds.z_cPO4*F_const*cPO4*tds.um_cPO4xx*d(tds.V,x)*test(cPO4x)*tds.d	2	Spatial	Domain 2
-tds.z_cH*F_const*cH*tds.um_cHxx*d(tds.V,x)*test(cHx)*tds.d	2	Spatial	Domain 2
-tds.z_cOH*F_const*cOH*tds.um_cOHxx*d(tds.V,x)*test(cOHx)*tds.d	2	Spatial	Domain 2
-tds.z_cHCO3*F_const*cHCO3*tds.um_cHCO3xx*d(tds.V,x)*test(cHCO3x)*tds.d	2	Spatial	Domain 2
-tds.z_cCO3*F_const*cCO3*tds.um_cCO3xx*d(tds.V,x)*test(cCO3x)*tds.d	2	Spatial	Domain 2
tds.streamline*(isScalingSystemDomain==0)*tds.d	2	Spatial	Domain 2
tds.crosswind*(isScalingSystemDomain==0)*tds.d	4	Spatial	Domain 2

2.4.4 No Flux 1

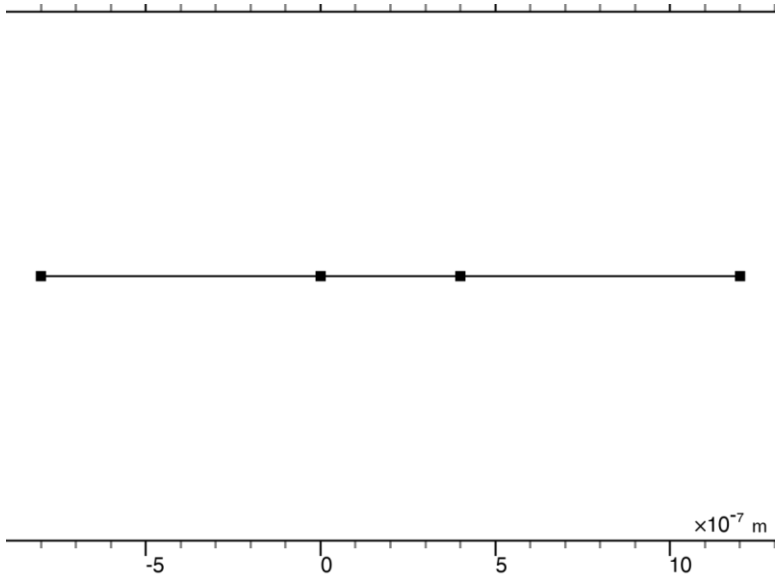

No Flux 1

SELECTION

Geometric entity level	Boundary
Selection	Geometry geom1: Dimension 0: All boundaries

EQUATIONS

$$-\mathbf{n}\cdot\mathbf{J}_i=0$$

2.4.5 Initial Values 1

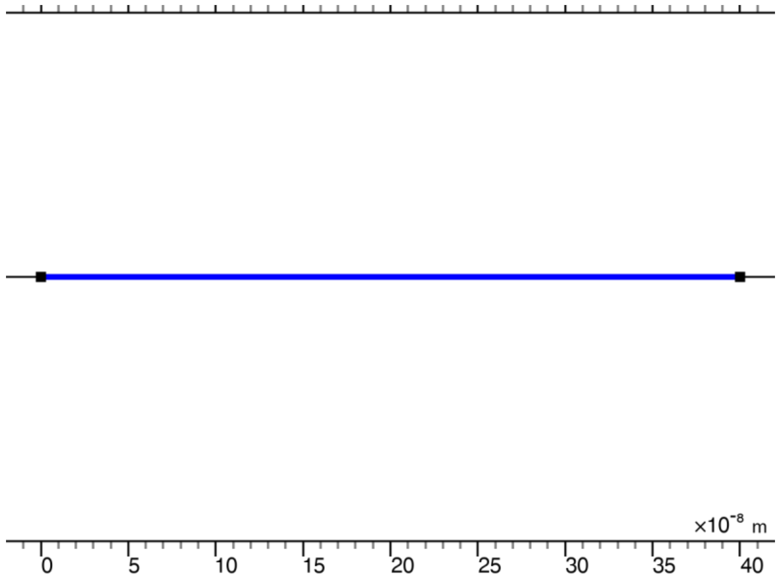

Initial Values 1

SELECTION

Geometric entity level	Domain
Selection	Geometry geom1: Dimension 1: All domains

Initial Values

SETTINGS

Description	Value
Concentration	{c0_ER, c0_ER, c0*H_CO2, 1e-10*H_CO, 1e-10*H_O2, 1, c0_B, c0_B, c0_ER + K_H2O/K_H2PO4 + c0_B + c0_B*2 + 3*c0_B*K_HPO4/K_H2PO4 - K_H2PO4, (rho_surf_left + rho_surf_right)/L/(N_A_const*e_const), c0_B*K_H2PO4/K_H3PO4, c0_B*K_HPO4/K_H2PO4, K_H2PO4, K_H2O/K_H2PO4, 0, 0}

Variables

Name	Expression	Unit	Description	Selection	Details
tds.c0_cAsc	c0_ER	mol/m³	Concentration	Domain 2	+ operation
tds.c0_cAscm	c0_ER	mol/m³	Concentration	Domain 2	+ operation
tds.c0_cCO2	c0*H_CO2	mol/m³	Concentration	Domain 2	+ operation
tds.c0_cCO	1.0E-10*H_CO	mol/m³	Concentration	Domain 2	+ operation
tds.c0_cO2	1.0E-10*H_O2	mol/m³	Concentration	Domain 2	+ operation
tds.c0_cH2O	1	mol/m³	Concentration	Domain 2	+ operation
tds.c0_cH2PO4	c0_B	mol/m³	Concentration	Domain 2	+ operation
tds.c0_cHPO4	c0_B	mol/m³	Concentration	Domain 2	+ operation
tds.c0_cNa	c0_ER+K_H2O/K_H2PO4+3*c0_B+3*c0_B*K_HPO4/K_H2PO4-K_H2PO4	mol/m³	Concentration	Domain 2	+ operation
tds.c0_cCl	(rho_surf_left+rho_surf_right)/(L*N_A_const*e_const)	mol/m³	Concentration	Domain 2	+ operation
tds.c0_cH3PO4	c0_B*K_H2PO4/K_H3PO4	mol/m³	Concentration	Domain 2	+ operation
tds.c0_cPO4	c0_B*K_HPO4/K_H2PO4	mol/m³	Concentration	Domain 2	+ operation
tds.c0_cH	K_H2PO4	mol/m³	Concentration	Domain 2	+ operation

Name	Expression	Unit	Description	Selection	Details
tds.c0_cOH	K_H2O/K_H2PO4	mol/m³	Concentration	Domain 2	+ operation
tds.c0_cHCO3	0	mol/m³	Concentration	Domain 2	+ operation
tds.c0_cCO3	0	mol/m³	Concentration	Domain 2	+ operation

2.4.6 Surface Reactions CO2 reduction

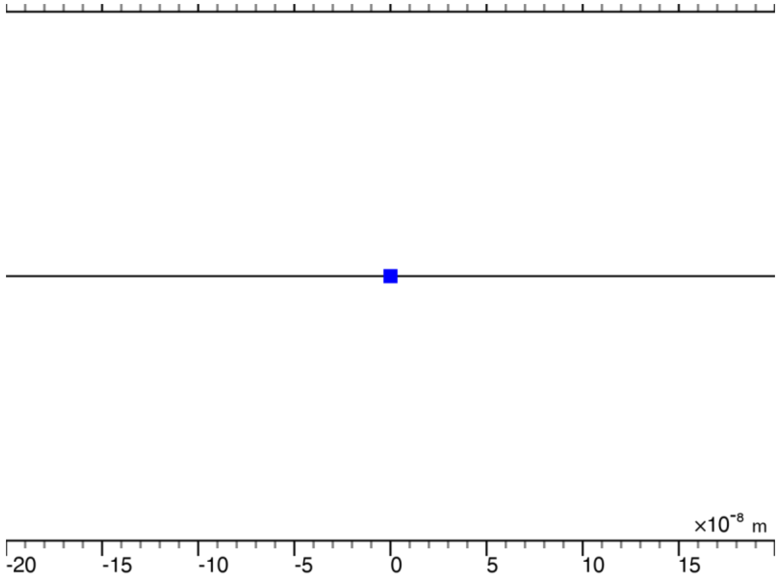

Surface Reactions CO2 reduction

SELECTION

Geometric entity level	Boundary
Selection	Geometry geom1: Dimension 0: Boundary 2

EQUATIONS

$$-\mathbf{n}\cdot\mathbf{J}_i = J_{0,i}$$

Surface Reaction Rate

SETTINGS

Description	Value
Surface reaction rate	Surface reaction rate for species Asc (chem)
Surface reaction rate	Surface reaction rate for species Ascm (chem)
Surface reaction rate	User defined
Surface reaction rate	0
Surface reaction rate	User defined

Description	Value
Surface reaction rate	0
Surface reaction rate	User defined
Surface reaction rate	0
Surface reaction rate	Surface reaction rate for species H2O (chem)
Surface reaction rate	Surface reaction rate for species H2PO4 (chem)
Surface reaction rate	Surface reaction rate for species HPO4 (chem)
Surface reaction rate	User defined
Surface reaction rate	0
Surface reaction rate	User defined
Surface reaction rate	0
Surface reaction rate	User defined
Surface reaction rate	0
Surface reaction rate	User defined
Surface reaction rate	0
Surface reaction rate	User defined
Surface reaction rate	0
Surface reaction rate	User defined
Surface reaction rate	0
Surface reaction rate	User defined
Surface reaction rate	0
Surface reaction rate	User defined
Surface reaction rate	0

Variables

Name	Expression	Unit	Description	Selection	Details
tds.J0_cAsc	model.input.J0_cAsc	mol/(m²·s)	Surface reaction rate	Boundary 2	Meta, + operation
tds.J0_cAscm	model.input.J0_cAscm	mol/(m²·s)	Surface reaction rate	Boundary 2	Meta, + operation
tds.J0_cCO2	model.input.J0_cCO2	mol/(m²·s)	Surface reaction rate	Boundary 2	Meta, + operation
tds.J0_cCO	model.input.J0_cCO	mol/(m²·s)	Surface reaction rate	Boundary 2	Meta, + operation
tds.J0_cO2	model.input.J0_cO2	mol/(m²·s)	Surface reaction rate	Boundary 2	Meta, + operation
tds.J0_cH2O	model.input.J0_cH2O	mol/(m²·s)	Surface reaction rate	Boundary 2	Meta, + operation

Name	Expression	Unit	Description	Selection	Details
tds.J0_cH2PO4	model.input.J0_cH2PO4	mol/(m²·s)	Surface reaction rate	Boundary 2	Meta, + operation
tds.J0_cHPO4	model.input.J0_cHPO4	mol/(m²·s)	Surface reaction rate	Boundary 2	Meta, + operation
tds.J0_cNa	model.input.J0_cNa	mol/(m²·s)	Surface reaction rate	Boundary 2	Meta, + operation
tds.J0_cCl	model.input.J0_cCl	mol/(m²·s)	Surface reaction rate	Boundary 2	Meta, + operation
tds.J0_cH3PO4	model.input.J0_cH3PO4	mol/(m²·s)	Surface reaction rate	Boundary 2	Meta, + operation
tds.J0_cPO4	model.input.J0_cPO4	mol/(m²·s)	Surface reaction rate	Boundary 2	Meta, + operation
tds.J0_cH	model.input.J0_cH	mol/(m²·s)	Surface reaction rate	Boundary 2	Meta, + operation
tds.J0_cOH	model.input.J0_cOH	mol/(m²·s)	Surface reaction rate	Boundary 2	Meta, + operation
tds.J0_cHCO3	model.input.J0_cHCO3	mol/(m²·s)	Surface reaction rate	Boundary 2	Meta, + operation
tds.J0_cCO3	model.input.J0_cCO3	mol/(m²·s)	Surface reaction rate	Boundary 2	Meta, + operation

Weak Expressions

Weak expression	Integration order	Integration frame	Selection
tds.J0_cAsc*test(cAsc)*tds.d	2	Spatial	Boundary 2
tds.J0_cAscm*test(cAscm)*tds.d	2	Spatial	Boundary 2
tds.J0_cCO2*test(cCO2)*tds.d	2	Spatial	Boundary 2
tds.J0_cCO*test(cCO)*tds.d	2	Spatial	Boundary 2
tds.J0_cO2*test(cO2)*tds.d	2	Spatial	Boundary 2
tds.J0_cH2O*test(cH2O)*tds.d	2	Spatial	Boundary 2
tds.J0_cH2PO4*test(cH2PO4)*tds.d	2	Spatial	Boundary 2
tds.J0_cHPO4*test(cHPO4)*tds.d	2	Spatial	Boundary 2
tds.J0_cNa*test(cNa)*tds.d	2	Spatial	Boundary 2
tds.J0_cCl*test(cCl)*tds.d	2	Spatial	Boundary 2
tds.J0_cH3PO4*test(cH3PO4)*tds.d	2	Spatial	Boundary 2
tds.J0_cPO4*test(cPO4)*tds.d	2	Spatial	Boundary 2
tds.J0_cH*test(cH)*tds.d	2	Spatial	Boundary 2
tds.J0_cOH*test(cOH)*tds.d	2	Spatial	Boundary 2
tds.J0_cHCO3*test(cHCO3)*tds.d	2	Spatial	Boundary 2

Weak expression	Integration order	Integration frame	Selection
tds.J0_cCO3*test(cCO3)*tds.d	2	Spatial	Boundary 2

2.4.7 Surface Reactions H2O oxidation

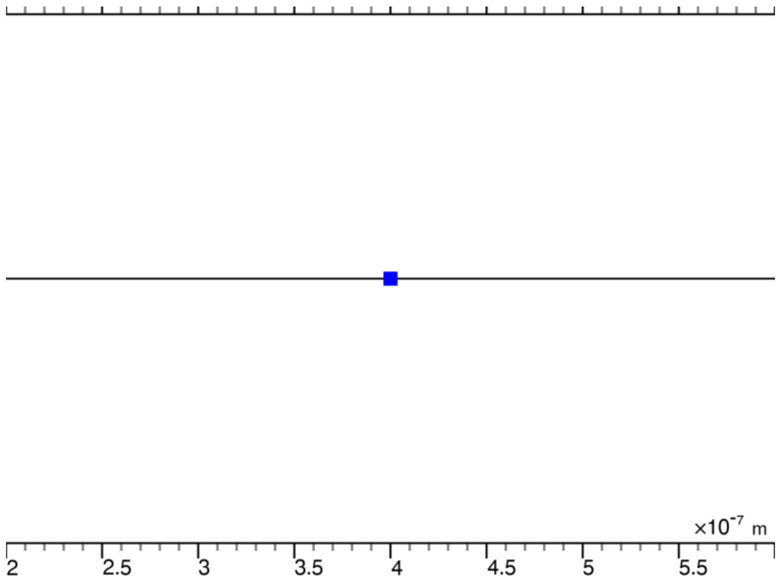

Surface Reactions H2O oxidation

SELECTION

Geometric entity level	Boundary
Selection	Geometry geom1: Dimension 0: Boundary 3

EQUATIONS

$$-\mathbf{n}\cdot\mathbf{J}_i = J_{0,i}$$

Surface Reaction Rate

SETTINGS

Description	Value
Surface reaction rate	Surface reaction rate for species Asc (chem2)
Surface reaction rate	Surface reaction rate for species Ascm (chem2)
Surface reaction rate	User defined
Surface reaction rate	0
Surface reaction rate	User defined
Surface reaction rate	0
Surface reaction rate	User defined
Surface reaction rate	0
Surface reaction rate	Surface reaction rate for species H2O (chem2)

Description	Value
Surface reaction rate	Surface reaction rate for species H2PO4 (chem2)
Surface reaction rate	Surface reaction rate for species HPO4 (chem2)
Surface reaction rate	User defined
Surface reaction rate	0
Surface reaction rate	User defined
Surface reaction rate	0
Surface reaction rate	User defined
Surface reaction rate	0
Surface reaction rate	User defined
Surface reaction rate	0
Surface reaction rate	User defined
Surface reaction rate	0
Surface reaction rate	User defined
Surface reaction rate	0
Surface reaction rate	User defined
Surface reaction rate	0
Surface reaction rate	User defined
Surface reaction rate	0

Variables

Name	Expression	Unit	Description	Selection	Details
tds.J0_cAsc	model.input.J0_cAsc	mol/(m²·s)	Surface reaction rate	Boundary 3	Meta, + operation
tds.J0_cAscm	model.input.J0_cAscm	mol/(m²·s)	Surface reaction rate	Boundary 3	Meta, + operation
tds.J0_cCO2	model.input.J0_cCO2	mol/(m²·s)	Surface reaction rate	Boundary 3	Meta, + operation
tds.J0_cCO	model.input.J0_cCO	mol/(m²·s)	Surface reaction rate	Boundary 3	Meta, + operation
tds.J0_cO2	model.input.J0_cO2	mol/(m²·s)	Surface reaction rate	Boundary 3	Meta, + operation
tds.J0_cH2O	model.input.J0_cH2O	mol/(m²·s)	Surface reaction rate	Boundary 3	Meta, + operation
tds.J0_cH2PO4	model.input.J0_cH2PO4	mol/(m²·s)	Surface reaction rate	Boundary 3	Meta, + operation
tds.J0_cHPO4	model.input.J0_cHPO4	mol/(m²·s)	Surface reaction rate	Boundary 3	Meta, + operation
tds.J0_cNa	model.input.J0_cNa	mol/(m²·s)	Surface reaction	Boundary 3	Meta,

Name	Expression	Unit	Description	Selection	Details
			rate		+ operation
tds.J0_cCl	model.input.J0_cCl	mol/(m²·s)	Surface reaction rate	Boundary 3	Meta, + operation
tds.J0_cH3PO4	model.input.J0_cH3PO4	mol/(m²·s)	Surface reaction rate	Boundary 3	Meta, + operation
tds.J0_cPO4	model.input.J0_cPO4	mol/(m²·s)	Surface reaction rate	Boundary 3	Meta, + operation
tds.J0_cH	model.input.J0_cH	mol/(m²·s)	Surface reaction rate	Boundary 3	Meta, + operation
tds.J0_cOH	model.input.J0_cOH	mol/(m²·s)	Surface reaction rate	Boundary 3	Meta, + operation
tds.J0_cHCO3	model.input.J0_cHCO3	mol/(m²·s)	Surface reaction rate	Boundary 3	Meta, + operation
tds.J0_cCO3	model.input.J0_cCO3	mol/(m²·s)	Surface reaction rate	Boundary 3	Meta, + operation

Weak Expressions

Weak expression	Integration order	Integration frame	Selection
tds.J0_cAsc*test(cAsc)*tds.d	2	Spatial	Boundary 3
tds.J0_cAscm*test(cAscm)*tds.d	2	Spatial	Boundary 3
tds.J0_cCO2*test(cCO2)*tds.d	2	Spatial	Boundary 3
tds.J0_cCO*test(cCO)*tds.d	2	Spatial	Boundary 3
tds.J0_cO2*test(cO2)*tds.d	2	Spatial	Boundary 3
tds.J0_cH2O*test(cH2O)*tds.d	2	Spatial	Boundary 3
tds.J0_cH2PO4*test(cH2PO4)*tds.d	2	Spatial	Boundary 3
tds.J0_cHPO4*test(cHPO4)*tds.d	2	Spatial	Boundary 3
tds.J0_cNa*test(cNa)*tds.d	2	Spatial	Boundary 3
tds.J0_cCl*test(cCl)*tds.d	2	Spatial	Boundary 3
tds.J0_cH3PO4*test(cH3PO4)*tds.d	2	Spatial	Boundary 3
tds.J0_cPO4*test(cPO4)*tds.d	2	Spatial	Boundary 3
tds.J0_cH*test(cH)*tds.d	2	Spatial	Boundary 3
tds.J0_cOH*test(cOH)*tds.d	2	Spatial	Boundary 3
tds.J0_cHCO3*test(cHCO3)*tds.d	2	Spatial	Boundary 3
tds.J0_cCO3*test(cCO3)*tds.d	2	Spatial	Boundary 3

2.4.8 Flux 1 vsH2O

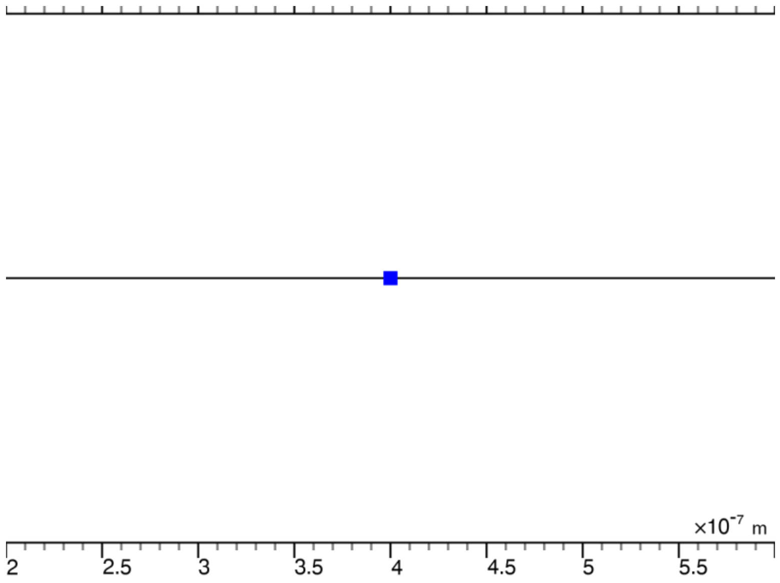

Flux 1 vsH2O

SELECTION

Geometric entity level	Boundary
Selection	Geometry geom1: Dimension 0: Boundary 3

EQUATIONS

$$-\mathbf{n}\cdot\mathbf{J}_i = J_{0,i}$$

Inward Flux

SETTINGS

Description	Value
Flux type	General inward flux
Species cAsc	Off
Species cAscm	Off
Species cCO2	On
Species cCO	On
Species cO2	On
Species cH2O	Off
Species cH2PO4	Off
Species cHPO4	Off
Species cNa	Off
Species cCl	Off
Species cH3PO4	Off

Description	Value
Species cPO4	Off
Species cH	Off
Species cOH	Off
Species cHCO3	Off
Species cCO3	Off

Variables

Name	Expression	Unit	Description	Selection
tds.fl1.nmflow_cAsc	tds.fl1.int(tds.ntflux_cAsc)*tds.d	mol/s	Normal molar flow rate	Global
tds.fl1.nmflow_cAscm	tds.fl1.int(tds.ntflux_cAscm)*tds.d	mol/s	Normal molar flow rate	Global
tds.fl1.nmflow_cCO2	tds.fl1.int(tds.ntflux_cCO2)*tds.d	mol/s	Normal molar flow rate	Global
tds.fl1.nmflow_cCO	tds.fl1.int(tds.ntflux_cCO)*tds.d	mol/s	Normal molar flow rate	Global
tds.fl1.nmflow_cO2	tds.fl1.int(tds.ntflux_cO2)*tds.d	mol/s	Normal molar flow rate	Global
tds.fl1.nmflow_cH2O	tds.fl1.int(tds.ntflux_cH2O)*tds.d	mol/s	Normal molar flow rate	Global
tds.fl1.nmflow_cH2PO4	tds.fl1.int(tds.ntflux_cH2PO4)*tds.d	mol/s	Normal molar flow rate	Global
tds.fl1.nmflow_cHPO4	tds.fl1.int(tds.ntflux_cHPO4)*tds.d	mol/s	Normal molar flow rate	Global
tds.fl1.nmflow_cNa	tds.fl1.int(tds.ntflux_cNa)*tds.d	mol/s	Normal molar flow rate	Global
tds.fl1.nmflow_cCl	tds.fl1.int(tds.ntflux_cCl)*tds.d	mol/s	Normal molar flow rate	Global
tds.fl1.nmflow_cH3PO4	tds.fl1.int(tds.ntflux_cH3PO4)*tds.d	mol/s	Normal molar flow rate	Global
tds.fl1.nmflow_cPO4	tds.fl1.int(tds.ntflux_cPO4)*tds.d	mol/s	Normal molar flow rate	Global
tds.fl1.nmflow_cH	tds.fl1.int(tds.ntflux_cH)*tds.d	mol/s	Normal molar flow rate	Global
tds.fl1.nmflow_cOH	tds.fl1.int(tds.ntflux_cOH)*tds.d	mol/s	Normal molar flow rate	Global
tds.fl1.nmflow_cHCO3	tds.fl1.int(tds.ntflux_cHCO3)*tds.d	mol/s	Normal molar flow rate	Global
tds.fl1.nmflow_cCO3	tds.fl1.int(tds.ntflux_cCO3)*tds.d	mol/s	Normal molar flow rate	Global

Weak Expressions

Weak expression	Integration order	Integration frame	Selection
0	2	Spatial	Boundary 3
0	2	Spatial	Boundary 3
k1_CO2*(ca2_CO2-cCO2/H_CO2)*test(cCO2)*tds.d	2	Spatial	Boundary 3
k1_CO*(ca2_CO-cCO/H_CO)*test(cCO)*tds.d	2	Spatial	Boundary 3
k1_O2*(ca2_O2-cO2/H_O2)*test(cO2)*tds.d	2	Spatial	Boundary 3
0	2	Spatial	Boundary 3
0	2	Spatial	Boundary 3
0	2	Spatial	Boundary 3
0	2	Spatial	Boundary 3
0	2	Spatial	Boundary 3
0	2	Spatial	Boundary 3
0	2	Spatial	Boundary 3
0	2	Spatial	Boundary 3
0	2	Spatial	Boundary 3
0	2	Spatial	Boundary 3
0	2	Spatial	Boundary 3

2.4.9 Flux 2 vsCO2

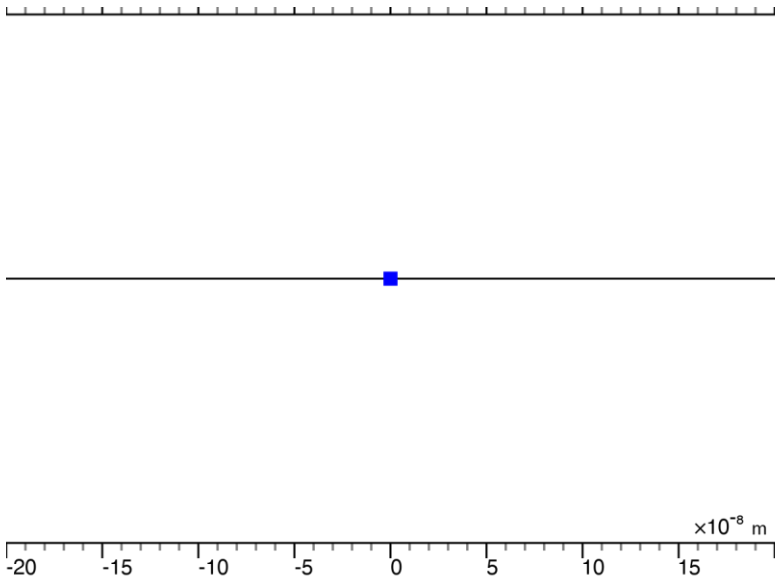

Flux 2 vsCO2

SELECTION

Geometric entity level	Boundary
Selection	Geometry geom1: Dimension 0: Boundary 2

EQUATIONS

$$-\mathbf{n}\cdot\mathbf{J}_i = J_{0,i}$$

Inward Flux

SETTINGS

Description	Value
Flux type	General inward flux
Species cAsc	Off
Species cAscm	Off
Species cCO2	On
Species cCO	On
Species cO2	On
Species cH2O	Off
Species cH2PO4	Off
Species cHPO4	Off
Species cNa	Off
Species cCl	Off
Species cH3PO4	Off
Species cPO4	Off
Species cH	Off
Species cOH	Off
Species cHCO3	Off
Species cCO3	Off

Variables

Name	Expression	Unit	Description	Selection
tds.fl2.nmflow_cAsc	tds.fl2.int(tds.ntflux_cAsc)*tds.d	mol/s	Normal molar flow rate	Global
tds.fl2.nmflow_cAscm	tds.fl2.int(tds.ntflux_cAscm)*tds.d	mol/s	Normal molar flow rate	Global
tds.fl2.nmflow_cCO2	tds.fl2.int(tds.ntflux_cCO2)*tds.d	mol/s	Normal molar flow rate	Global
tds.fl2.nmflow_cCO	tds.fl2.int(tds.ntflux_cCO)*tds.d	mol/s	Normal molar flow rate	Global
tds.fl2.nmflow_cO2	tds.fl2.int(tds.ntflux_cO2)*tds.d	mol/s	Normal molar	Global

Name	Expression	Unit	Description	Selection
			flow rate	
tds.fl2.nmflow_cH2O	tds.fl2.int(tds.ntflux_cH2O)*tds.d	mol/s	Normal molar flow rate	Global
tds.fl2.nmflow_cH2PO4	tds.fl2.int(tds.ntflux_cH2PO4)*tds.d	mol/s	Normal molar flow rate	Global
tds.fl2.nmflow_cHPO4	tds.fl2.int(tds.ntflux_cHPO4)*tds.d	mol/s	Normal molar flow rate	Global
tds.fl2.nmflow_cNa	tds.fl2.int(tds.ntflux_cNa)*tds.d	mol/s	Normal molar flow rate	Global
tds.fl2.nmflow_cCl	tds.fl2.int(tds.ntflux_cCl)*tds.d	mol/s	Normal molar flow rate	Global
tds.fl2.nmflow_cH3PO4	tds.fl2.int(tds.ntflux_cH3PO4)*tds.d	mol/s	Normal molar flow rate	Global
tds.fl2.nmflow_cPO4	tds.fl2.int(tds.ntflux_cPO4)*tds.d	mol/s	Normal molar flow rate	Global
tds.fl2.nmflow_cH	tds.fl2.int(tds.ntflux_cH)*tds.d	mol/s	Normal molar flow rate	Global
tds.fl2.nmflow_cOH	tds.fl2.int(tds.ntflux_cOH)*tds.d	mol/s	Normal molar flow rate	Global
tds.fl2.nmflow_cHCO3	tds.fl2.int(tds.ntflux_cHCO3)*tds.d	mol/s	Normal molar flow rate	Global
tds.fl2.nmflow_cCO3	tds.fl2.int(tds.ntflux_cCO3)*tds.d	mol/s	Normal molar flow rate	Global

Weak Expressions

Weak expression	Integration order	Integration frame	Selection
0	2	Spatial	Boundary 2
0	2	Spatial	Boundary 2
k1_CO2*(ca1_CO2-cCO2/H_CO2)*test(cCO2)*tds.d	2	Spatial	Boundary 2
k1_CO*(ca1_CO-cCO/H_CO)*test(cCO)*tds.d	2	Spatial	Boundary 2
k1_O2*(ca1_O2-cO2/H_O2)*test(cO2)*tds.d	2	Spatial	Boundary 2
0	2	Spatial	Boundary 2
0	2	Spatial	Boundary 2
0	2	Spatial	Boundary 2
0	2	Spatial	Boundary 2
0	2	Spatial	Boundary 2

Weak expression	Integration order	Integration frame	Selection
0	2	Spatial	Boundary 2
0	2	Spatial	Boundary 2
0	2	Spatial	Boundary 2
0	2	Spatial	Boundary 2
0	2	Spatial	Boundary 2
0	2	Spatial	Boundary 2

2.4.10 Reactions 1

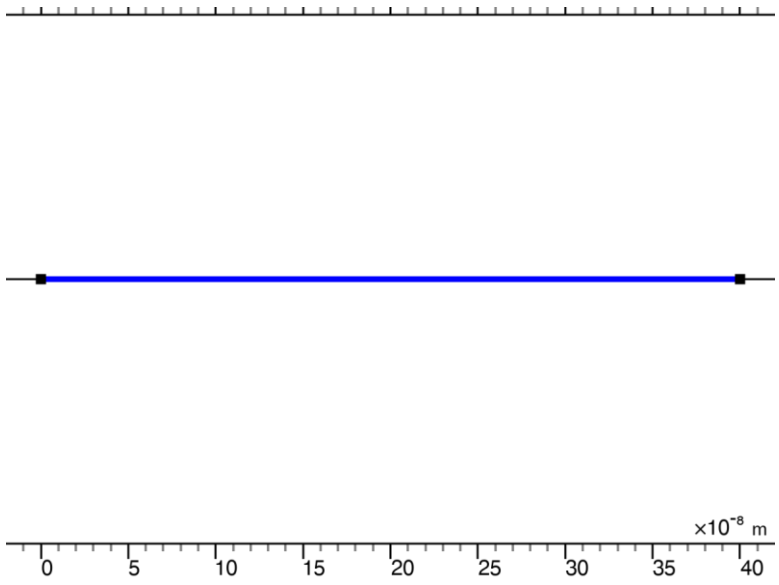

Reactions 1

SELECTION

Geometric entity level	Domain
Selection	Geometry geom1: Dimension 1: Domain 2

EQUATIONS

$$\frac{\partial c_i}{\partial t} + \nabla \cdot \mathbf{J}_i = R_i + S_i$$

Reaction Rates

SETTINGS

Description	Value
Total rate expression	User defined
Total rate expression	0
Total rate expression	User defined

Description	Value
Total rate expression	0
Total rate expression	Reaction rate for species CO2 (chem3)
Total rate expression	User defined
Total rate expression	0
Total rate expression	User defined
Total rate expression	0
Total rate expression	Reaction rate for species H2O (chem3)
Total rate expression	Reaction rate for species H2PO4 (chem3)
Total rate expression	Reaction rate for species HPO4 (chem3)
Total rate expression	User defined
Total rate expression	0
Total rate expression	User defined
Total rate expression	0
Total rate expression	Reaction rate for species H3PO4 (chem3)
Total rate expression	Reaction rate for species PO4 (chem3)
Total rate expression	Reaction rate for species H (chem3)
Total rate expression	Reaction rate for species OH (chem3)
Total rate expression	Reaction rate for species HCO3 (chem3)
Total rate expression	Reaction rate for species CO3 (chem3)

Variables

Name	Expression	Unit	Description	Selection	Details
tds.R_cAsc	tds.reac1.R_cAsc	$mol/(m^3 \cdot s)$	Total rate expression	Domain 2	+ operation
tds.R_cAscm	tds.reac1.R_cAscm	$mol/(m^3 \cdot s)$	Total rate expression	Domain 2	+ operation
tds.R_cCO2	tds.reac1.R_cCO2	$mol/(m^3 \cdot s)$	Total rate expression	Domain 2	+ operation
tds.R_cCO	tds.reac1.R_cCO	$mol/(m^3 \cdot s)$	Total rate expression	Domain 2	+ operation
tds.R_cO2	tds.reac1.R_cO2	$mol/(m^3 \cdot s)$	Total rate expression	Domain 2	+ operation
tds.R_cH2O	tds.reac1.R_cH2O	$mol/(m^3 \cdot s)$	Total rate expression	Domain 2	+ operation
tds.R_cH2PO4	tds.reac1.R_cH2PO4	$mol/(m^3 \cdot s)$	Total rate expression	Domain 2	+ operation
tds.R_cHPO4	tds.reac1.R_cHPO4	$mol/(m^3 \cdot s)$	Total rate expression	Domain 2	+ operation

Name	Expression	Unit	Description	Selection	Details
tds.R_cNa	tds.reac1.R_cNa	mol/(m³·s)	Total rate expression	Domain 2	+ operation
tds.R_cCl	tds.reac1.R_cCl	mol/(m³·s)	Total rate expression	Domain 2	+ operation
tds.R_cH3PO4	tds.reac1.R_cH3PO4	mol/(m³·s)	Total rate expression	Domain 2	+ operation
tds.R_cPO4	tds.reac1.R_cPO4	mol/(m³·s)	Total rate expression	Domain 2	+ operation
tds.R_cH	tds.reac1.R_cH	mol/(m³·s)	Total rate expression	Domain 2	+ operation
tds.R_cOH	tds.reac1.R_cOH	mol/(m³·s)	Total rate expression	Domain 2	+ operation
tds.R_cHCO3	tds.reac1.R_cHCO3	mol/(m³·s)	Total rate expression	Domain 2	+ operation
tds.R_cCO3	tds.reac1.R_cCO3	mol/(m³·s)	Total rate expression	Domain 2	+ operation
tds.reac1.R_cAsc	model.input.R_cAsc	mol/(m³·s)	Total rate expression	Domain 2	Meta
tds.reac1.R_cAscm	model.input.R_cAscm	mol/(m³·s)	Total rate expression	Domain 2	Meta
tds.reac1.R_cCO2	model.input.R_cCO2	mol/(m³·s)	Total rate expression	Domain 2	Meta
tds.reac1.R_cCO	model.input.R_cCO	mol/(m³·s)	Total rate expression	Domain 2	Meta
tds.reac1.R_cO2	model.input.R_cO2	mol/(m³·s)	Total rate expression	Domain 2	Meta
tds.reac1.R_cH2O	model.input.R_cH2O	mol/(m³·s)	Total rate expression	Domain 2	Meta
tds.reac1.R_cH2PO4	model.input.R_cH2PO4	mol/(m³·s)	Total rate expression	Domain 2	Meta
tds.reac1.R_cHPO4	model.input.R_cHPO4	mol/(m³·s)	Total rate expression	Domain 2	Meta
tds.reac1.R_cNa	model.input.R_cNa	mol/(m³·s)	Total rate expression	Domain 2	Meta
tds.reac1.R_cCl	model.input.R_cCl	mol/(m³·s)	Total rate expression	Domain 2	Meta
tds.reac1.R_cH3PO4	model.input.R_cH3PO4	mol/(m³·s)	Total rate expression	Domain 2	Meta
tds.reac1.R_cPO4	model.input.R_cPO4	mol/(m³·s)	Total rate expression	Domain 2	Meta
tds.reac1.R_cH	model.input.R_cH	mol/(m³·s)	Total rate	Domain 2	Meta

Name	Expression	Unit	Description	Selection	Details
			expression		
tds.reac1.R_cOH	model.input.R_cOH	mol/(m³·s)	Total rate expression	Domain 2	Meta
tds.reac1.R_cHCO3	model.input.R_cHCO3	mol/(m³·s)	Total rate expression	Domain 2	Meta
tds.reac1.R_cCO3	model.input.R_cCO3	mol/(m³·s)	Total rate expression	Domain 2	Meta

Weak Expressions

Weak expression	Integration order	Integration frame	Selection
tds.reac1.R_cAsc*test(cAsc)*tds.d	2	Spatial	Domain 2
tds.reac1.R_cAscm*test(cAscm)*tds.d	2	Spatial	Domain 2
tds.reac1.R_cCO2*test(cCO2)*tds.d	2	Spatial	Domain 2
tds.reac1.R_cCO*test(cCO)*tds.d	2	Spatial	Domain 2
tds.reac1.R_cO2*test(cO2)*tds.d	2	Spatial	Domain 2
tds.reac1.R_cH2O*test(cH2O)*tds.d	2	Spatial	Domain 2
tds.reac1.R_cH2PO4*test(cH2PO4)*tds.d	2	Spatial	Domain 2
tds.reac1.R_cHPO4*test(cHPO4)*tds.d	2	Spatial	Domain 2
tds.reac1.R_cNa*test(cNa)*tds.d	2	Spatial	Domain 2
tds.reac1.R_cCl*test(cCl)*tds.d	2	Spatial	Domain 2
tds.reac1.R_cH3PO4*test(cH3PO4)*tds.d	2	Spatial	Domain 2
tds.reac1.R_cPO4*test(cPO4)*tds.d	2	Spatial	Domain 2
tds.reac1.R_cH*test(cH)*tds.d	2	Spatial	Domain 2
tds.reac1.R_cOH*test(cOH)*tds.d	2	Spatial	Domain 2
tds.reac1.R_cHCO3*test(cHCO3)*tds.d	2	Spatial	Domain 2
tds.reac1.R_cCO3*test(cCO3)*tds.d	2	Spatial	Domain 2

2.5 TRANSPORT OF DILUTED SPECIES CO2 SIDE

USED PRODUCTS

COMSOL Multiphysics
Chemical Reaction Engineering Module

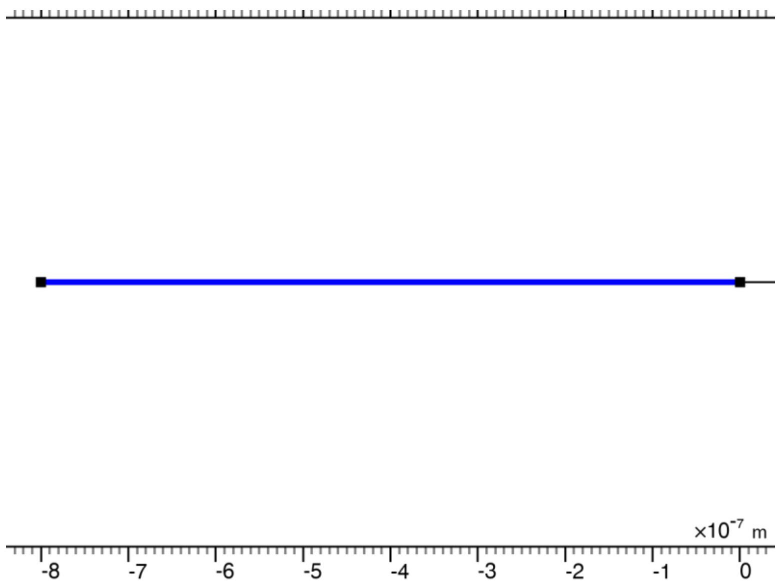

Transport of Diluted Species CO2 side

SELECTION

Geometric entity level	Domain
Selection	Geometry geom1: Dimension 1: Domain 1

EQUATIONS

$$\frac{\partial c_i}{\partial t} + \nabla \cdot \mathbf{J}_i = R_i$$
$$\mathbf{J}_i = -D_i \nabla c_i$$

2.5.1 Interface Settings

Discretization

SETTINGS

Description	Value
Concentration	Linear

SETTINGS

Description	Value
Equation form	Study controlled

Cross-Sectional Area

SETTINGS

Description	Value
Cross-sectional area	1[m^2]

Consistent Stabilization

SETTINGS

Description	Value
Streamline diffusion	On
Crosswind diffusion	On
Equation residual	Approximate residual
Crosswind diffusion type	Do Carmo and Galeão

Advanced Settings

SETTINGS

Description	Value
Convective term	Nonconservative form

Transport Mechanisms

SETTINGS

Description	Value
Convection	Off
Migration in electric field	Off
Mass transfer in porous media	Off

2.5.2 Variables

Name	Expression	Unit	Description	Selection	Details
tds2.Ac	1[m^2]	m^2	Cross-sectional area	Global	
tds2.d	tds2.Ac	m^2	Out-of-plane geometry extension	Global	
tds2.nx	unx	1	Normal vector, x component	Boundary 1	
tds2.ny	0	1	Normal vector, y component	Boundary 1	
tds2.nz	0	1	Normal vector, z component	Boundary 1	
tds2.nx	dnx	1	Normal vector, x component	Boundary 2	
tds2.ny	0	1	Normal vector, y component	Boundary 2	
tds2.nz	0	1	Normal vector, z component	Boundary 2	
tds2.nxmesh	unxmesh	1	Normal vector (mesh), x	Boundary 1	

Name	Expression	Unit	Description	Selection	Details
			component		
tds2.nymesh	0	1	Normal vector (mesh), y component	Boundary 1	
tds2.nzmesh	0	1	Normal vector (mesh), z component	Boundary 1	
tds2.nxmesh	dnxmesh	1	Normal vector (mesh), x component	Boundary 2	
tds2.nymesh	0	1	Normal vector (mesh), y component	Boundary 2	
tds2.nzmesh	0	1	Normal vector (mesh), z component	Boundary 2	
tds2.nxc	-root.nxc/tds2.ncLen	1	Normal vector, x component	Boundary 1	
tds2.nyc	0	1	Normal vector, y component	Boundary 1	
tds2.nzc	0	1	Normal vector, z component	Boundary 1	
tds2.nxc	root.nxc/tds2.ncLen	1	Normal vector, x component	Boundary 2	
tds2.nyc	0	1	Normal vector, y component	Boundary 2	
tds2.nzc	0	1	Normal vector, z component	Boundary 2	
tds2.R_ca1_O2	0	$mol/(m^3\cdot s)$	Total rate expression	Domain 1	+ operation
tds2.cP_ca1_O2	0	mol/kg	Concentration species adsorbed to the solid	Domain 1	+ operation
tds2.cP_ca1_O2	0	mol/kg	Concentration species adsorbed to the solid	Boundaries 1–2	+ operation
tds2.KP_ca1_O2	0	m^3/kg	Adsorption isotherm, first concentration derivative	Domain 1	+ operation
tds2.KP_ca1_O2	0	m^3/kg	Adsorption isotherm, first concentration	Boundaries 1–2	+ operation

Name	Expression	Unit	Description	Selection	Details
			derivative		
tds2.Rads_ca1_O2	0	mol/(m³·s)	Total adsorption rate	Domain 1	+ operation
tds2.DiT_ca1_O2	0	m²/s	Turbulent diffusivity	Domain 1	
tds2.cVar_ca1_O2	ca1_O2	mol/m³	Species	Boundaries 1–2	
tds2.R_ca1_CO2	0	mol/(m³·s)	Total rate expression	Domain 1	+ operation
tds2.cP_ca1_CO2	0	mol/kg	Concentration species adsorbed to the solid	Domain 1	+ operation
tds2.cP_ca1_CO2	0	mol/kg	Concentration species adsorbed to the solid	Boundaries 1–2	+ operation
tds2.KP_ca1_CO2	0	m³/kg	Adsorption isotherm, first concentration derivative	Domain 1	+ operation
tds2.KP_ca1_CO2	0	m³/kg	Adsorption isotherm, first concentration derivative	Boundaries 1–2	+ operation
tds2.Rads_ca1_CO2	0	mol/(m³·s)	Total adsorption rate	Domain 1	+ operation
tds2.DiT_ca1_CO2	0	m²/s	Turbulent diffusivity	Domain 1	
tds2.cVar_ca1_CO2	ca1_CO2	mol/m³	Species	Boundaries 1–2	
tds2.R_ca1_CO	0	mol/(m³·s)	Total rate expression	Domain 1	+ operation
tds2.cP_ca1_CO	0	mol/kg	Concentration species adsorbed to the solid	Domain 1	+ operation
tds2.cP_ca1_CO	0	mol/kg	Concentration species adsorbed to the solid	Boundaries 1–2	+ operation
tds2.KP_ca1_CO	0	m³/kg	Adsorption isotherm, first concentration derivative	Domain 1	+ operation
tds2.KP_ca1_CO	0	m³/kg	Adsorption isotherm, first concentration derivative	Boundaries 1–2	+ operation

Name	Expression	Unit	Description	Selection	Details
tds2.Rads_ca1_CO	0	mol/(m³·s)	Total adsorption rate	Domain 1	+ operation
tds2.DiT_ca1_CO	0	m²/s	Turbulent diffusivity	Domain 1	
tds2.cVar_ca1_CO	ca1_CO	mol/m³	Species	Boundaries 1–2	
tds2.poro	1	1	Porosity	Domain 1	
tds2.theta_g	0	1	Gas volume fraction	Domain 1	
tds2.theta_l	1	1	Liquid volume fraction	Domain 1	
tds2.theta	tds2.poro	1	Mobile fluid volume fraction	Domain 1	

2.5.3 Transport Properties 1

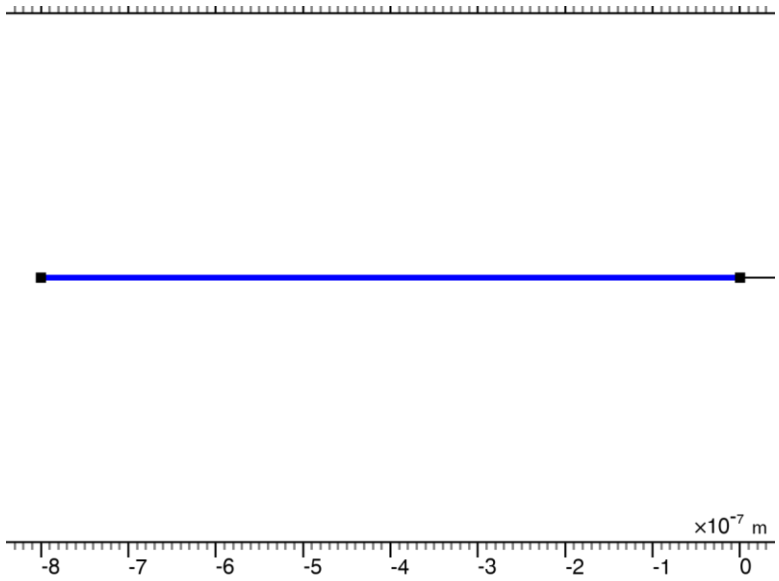

Transport Properties 1

SELECTION

Geometric entity level	Domain
Selection	Geometry geom1: Dimension 1: All domains

EQUATIONS

$$\frac{\partial c_i}{\partial t} + \nabla \cdot \mathbf{J}_i = R_i$$

$$\mathbf{J}_i = -D_i \nabla c_i$$

Diffusion

SETTINGS

Description	Value
Source	Material
Material	None
Diffusion coefficient	User defined
Diffusion coefficient	{{D_O2, 0, 0}, {0, D_O2, 0}, {0, 0, D_O2}}
Diffusion coefficient	User defined
Diffusion coefficient	{{D_CO2, 0, 0}, {0, D_CO2, 0}, {0, 0, D_CO2}}
Diffusion coefficient	User defined
Diffusion coefficient	{{D_CO, 0, 0}, {0, D_CO, 0}, {0, 0, D_CO}}

Coordinate System Selection

SETTINGS

Description	Value
Coordinate system	Global coordinate system

Model Input

SETTINGS

Description	Value
Temperature	Common model input

Variables

Name	Expression	Unit	Description	Selection	Details
domflux.ca1_O2x	tds2.dflux_ca1_O2x*tds2.d	mol/s	Domain flux, x component	Domain 1	
domflux.ca1_CO2x	tds2.dflux_ca1_CO2x*tds2.d	mol/s	Domain flux, x component	Domain 1	
domflux.ca1_COx	tds2.dflux_ca1_COx*tds2.d	mol/s	Domain flux, x component	Domain 1	
tds2.ndflux_ca1_O2	tds2.bndFlux_ca1_O2	mol/(m²·s)	Normal diffusive flux	Boundaries 1–2	
tds2.ntflux_ca1_O2	tds2.bndFlux_ca1_O2	mol/(m²·s)	Normal total flux	Boundaries 1–2	
tds2.ndflux_ca1_CO2	tds2.bndFlux_ca1_CO2	mol/(m²·s)	Normal diffusive flux	Boundaries 1–2	
tds2.ntflux_ca1_CO2	tds2.bndFlux_ca1_CO2	mol/(m²·s)	Normal total flux	Boundaries 1–2	
tds2.ndflux_ca1_CO	tds2.bndFlux_ca1_CO	mol/(m²·s)	Normal diffusive flux	Boundaries 1–2	
tds2.ntflux_ca1_CO	tds2.bndFlux_ca1_CO	mol/(m²·s)	Normal total flux	Boundaries 1–2	

Name	Expression	Unit	Description	Selection	Details
tds2.bndFlux_ca1_CO2	-uflux_spatial(ca1_CO2)/tds2.d	mol/(m²·s)	Boundary flux	Boundary 1	Meta
tds2.bndFlux_ca1_CO2	-dflux_spatial(ca1_CO2)/tds2.d	mol/(m²·s)	Boundary flux	Boundary 2	Meta
tds2.DF_ca1_O2xx	D_O2	m²/s	Fluid diffusion coefficient, xx component	Domain 1	
tds2.DF_ca1_O2yx	0	m²/s	Fluid diffusion coefficient, yx component	Domain 1	
tds2.DF_ca1_O2zx	0	m²/s	Fluid diffusion coefficient, zx component	Domain 1	
tds2.DF_ca1_O2xy	0	m²/s	Fluid diffusion coefficient, xy component	Domain 1	
tds2.DF_ca1_O2yy	D_O2	m²/s	Fluid diffusion coefficient, yy component	Domain 1	
tds2.DF_ca1_O2zy	0	m²/s	Fluid diffusion coefficient, zy component	Domain 1	
tds2.DF_ca1_O2xz	0	m²/s	Fluid diffusion coefficient, xz component	Domain 1	
tds2.DF_ca1_O2yz	0	m²/s	Fluid diffusion coefficient, yz component	Domain 1	
tds2.DF_ca1_O2zz	D_O2	m²/s	Fluid diffusion coefficient, zz component	Domain 1	
tds2.D_ca1_O2xx	tds2.DF_ca1_O2xx+tds2.DiT_ca1_O2	m²/s	Diffusion coefficient, xx component	Domain 1	
tds2.D_ca1_O2yx	tds2.DF_ca1_O2yx	m²/s	Diffusion coefficient, yx component	Domain 1	
tds2.D_ca1_O2zx	tds2.DF_ca1_O2zx	m²/s	Diffusion coefficient, zx component	Domain 1	
tds2.D_ca1_O2xy	tds2.DF_ca1_O2xy	m²/s	Diffusion	Domain 1	

Name	Expression	Unit	Description	Selection	Details
			coefficient, xy component		
tds2.D_ca1_O2yy	tds2.DF_ca1_O2yy+tds2.DiT_ca1_O2	m²/s	Diffusion coefficient, yy component	Domain 1	
tds2.D_ca1_O2zy	tds2.DF_ca1_O2zy	m²/s	Diffusion coefficient, zy component	Domain 1	
tds2.D_ca1_O2xz	tds2.DF_ca1_O2xz	m²/s	Diffusion coefficient, xz component	Domain 1	
tds2.D_ca1_O2yz	tds2.DF_ca1_O2yz	m²/s	Diffusion coefficient, yz component	Domain 1	
tds2.D_ca1_O2zz	tds2.DF_ca1_O2zz+tds2.DiT_ca1_O2	m²/s	Diffusion coefficient, zz component	Domain 1	
tds2.DF_ca1_CO2xx	D_CO2	m²/s	Fluid diffusion coefficient, xx component	Domain 1	
tds2.DF_ca1_CO2yx	0	m²/s	Fluid diffusion coefficient, yx component	Domain 1	
tds2.DF_ca1_CO2zx	0	m²/s	Fluid diffusion coefficient, zx component	Domain 1	
tds2.DF_ca1_CO2xy	0	m²/s	Fluid diffusion coefficient, xy component	Domain 1	
tds2.DF_ca1_CO2yy	D_CO2	m²/s	Fluid diffusion coefficient, yy component	Domain 1	
tds2.DF_ca1_CO2zy	0	m²/s	Fluid diffusion coefficient, zy component	Domain 1	
tds2.DF_ca1_CO2xz	0	m²/s	Fluid diffusion coefficient, xz component	Domain 1	
tds2.DF_ca1_CO2yz	0	m²/s	Fluid diffusion coefficient, yz component	Domain 1	
tds2.DF_ca1_CO2zz	D_CO2	m²/s	Fluid diffusion coefficient, zz	Domain 1	

Name	Expression	Unit	Description	Selection	Details
			component		
tds2.D_ca1_CO2xx	tds2.DF_ca1_CO2xx +tds2.DiT_ca1_CO2	m^2/s	Diffusion coefficient, xx component	Domain 1	
tds2.D_ca1_CO2yx	tds2.DF_ca1_CO2yx	m^2/s	Diffusion coefficient, yx component	Domain 1	
tds2.D_ca1_CO2zx	tds2.DF_ca1_CO2zx	m^2/s	Diffusion coefficient, zx component	Domain 1	
tds2.D_ca1_CO2xy	tds2.DF_ca1_CO2xy	m^2/s	Diffusion coefficient, xy component	Domain 1	
tds2.D_ca1_CO2yy	tds2.DF_ca1_CO2yy +tds2.DiT_ca1_CO2	m^2/s	Diffusion coefficient, yy component	Domain 1	
tds2.D_ca1_CO2zy	tds2.DF_ca1_CO2zy	m^2/s	Diffusion coefficient, zy component	Domain 1	
tds2.D_ca1_CO2xz	tds2.DF_ca1_CO2xz	m^2/s	Diffusion coefficient, xz component	Domain 1	
tds2.D_ca1_CO2yz	tds2.DF_ca1_CO2yz	m^2/s	Diffusion coefficient, yz component	Domain 1	
tds2.D_ca1_CO2zz	tds2.DF_ca1_CO2zz +tds2.DiT_ca1_CO2	m^2/s	Diffusion coefficient, zz component	Domain 1	
tds2.DF_ca1_COxx	D_CO	m^2/s	Fluid diffusion coefficient, xx component	Domain 1	
tds2.DF_ca1_COyx	0	m^2/s	Fluid diffusion coefficient, yx component	Domain 1	
tds2.DF_ca1_COzx	0	m^2/s	Fluid diffusion coefficient, zx component	Domain 1	
tds2.DF_ca1_COxy	0	m^2/s	Fluid diffusion coefficient, xy component	Domain 1	
tds2.DF_ca1_COyy	D_CO	m^2/s	Fluid diffusion coefficient, yy component	Domain 1	

Name	Expression	Unit	Description	Selection	Details
tds2.DF_ca1_COzy	0	m²/s	Fluid diffusion coefficient, zy component	Domain 1	
tds2.DF_ca1_COxz	0	m²/s	Fluid diffusion coefficient, xz component	Domain 1	
tds2.DF_ca1_COyz	0	m²/s	Fluid diffusion coefficient, yz component	Domain 1	
tds2.DF_ca1_COzz	D_CO	m²/s	Fluid diffusion coefficient, zz component	Domain 1	
tds2.D_ca1_COxx	tds2.DF_ca1_COxx+ tds2.DiT_ca1_CO	m²/s	Diffusion coefficient, xx component	Domain 1	
tds2.D_ca1_COyx	tds2.DF_ca1_COyx	m²/s	Diffusion coefficient, yx component	Domain 1	
tds2.D_ca1_COzx	tds2.DF_ca1_COzx	m²/s	Diffusion coefficient, zx component	Domain 1	
tds2.D_ca1_COxy	tds2.DF_ca1_COxy	m²/s	Diffusion coefficient, xy component	Domain 1	
tds2.D_ca1_COyy	tds2.DF_ca1_COyy+ tds2.DiT_ca1_CO	m²/s	Diffusion coefficient, yy component	Domain 1	
tds2.D_ca1_COzy	tds2.DF_ca1_COzy	m²/s	Diffusion coefficient, zy component	Domain 1	
tds2.D_ca1_COxz	tds2.DF_ca1_COxz	m²/s	Diffusion coefficient, xz component	Domain 1	
tds2.D_ca1_COyz	tds2.DF_ca1_COyz	m²/s	Diffusion coefficient, yz component	Domain 1	
tds2.D_ca1_COzz	tds2.DF_ca1_COzz+tds2.DiT_ca1_CO	m²/s	Diffusion coefficient, zz component	Domain 1	
tds2.Dav_ca1_O2	tds2.D_ca1_O2xx	m²/s	Average diffusion coefficient	Domain 1	
tds2.Dav_ca1_CO2	tds2.D_ca1_CO2xx	m²/s	Average diffusion coefficient	Domain 1	

Name	Expression	Unit	Description	Selection	Details
tds2.Dav_ca1_CO	tds2.D_ca1_COxx	m²/s	Average diffusion coefficient	Domain 1	
tds2.tflux_ca1_O2x	tds2.dflux_ca1_O2x	mol/(m²·s)	Total flux, x component	Domain 1	+ operation
tds2.tflux_ca1_O2y	tds2.dflux_ca1_O2y	mol/(m²·s)	Total flux, y component	Domain 1	+ operation
tds2.tflux_ca1_O2z	tds2.dflux_ca1_O2z	mol/(m²·s)	Total flux, z component	Domain 1	+ operation
tds2.dfluxMag_ca1_O2	sqrt(tds2.dflux_ca1_O2x^2+tds2.dflux_ca1_O2y^2+tds2.dflux_ca1_O2z^2)	mol/(m²·s)	Diffusive flux magnitude	Domain 1	
tds2.tfluxMag_ca1_O2	sqrt(tds2.tflux_ca1_O2x^2+tds2.tflux_ca1_O2y^2+tds2.tflux_ca1_O2z^2)	mol/(m²·s)	Total flux magnitude	Domain 1	
tds2.dpflux_ca1_O2x	0	mol/(m²·s)	Dispersive flux, x component	Domain 1	
tds2.dpflux_ca1_O2y	0	mol/(m²·s)	Dispersive flux, y component	Domain 1	
tds2.dpflux_ca1_O2z	0	mol/(m²·s)	Dispersive flux, z component	Domain 1	
tds2.tflux_ca1_CO2x	tds2.dflux_ca1_CO2x	mol/(m²·s)	Total flux, x component	Domain 1	+ operation
tds2.tflux_ca1_CO2y	tds2.dflux_ca1_CO2y	mol/(m²·s)	Total flux, y component	Domain 1	+ operation
tds2.tflux_ca1_CO2z	tds2.dflux_ca1_CO2z	mol/(m²·s)	Total flux, z component	Domain 1	+ operation
tds2.dfluxMag_ca1_CO2	sqrt(tds2.dflux_ca1_CO2x^2+tds2.dflux_ca1_CO2y^2+tds2.dflux_ca1_CO2z^2)	mol/(m²·s)	Diffusive flux magnitude	Domain 1	
tds2.tfluxMag_ca1_CO2	sqrt(tds2.tflux_ca1_CO2x^2+tds2.tflux_ca1_CO2y^2+tds2.tflux_ca1_CO2z^2)	mol/(m²·s)	Total flux magnitude	Domain 1	
tds2.dpflux_ca1_CO2x	0	mol/(m²·s)	Dispersive flux, x component	Domain 1	
tds2.dpflux_ca1_CO2y	0	mol/(m²·s)	Dispersive flux, y component	Domain 1	
tds2.dpflux_ca1_CO2z	0	mol/(m²·s)	Dispersive flux, z component	Domain 1	

Name	Expression	Unit	Description	Selection	Details
tds2.tflux_ca1_COx	tds2.dflux_ca1_COx	mol/(m²·s)	Total flux, x component	Domain 1	+ operation
tds2.tflux_ca1_COy	tds2.dflux_ca1_COy	mol/(m²·s)	Total flux, y component	Domain 1	+ operation
tds2.tflux_ca1_COz	tds2.dflux_ca1_COz	mol/(m²·s)	Total flux, z component	Domain 1	+ operation
tds2.dfluxMag_ca1_CO	sqrt(tds2.dflux_ca1_COx^2+tds2.dflux_ca1_COy^2+tds2.dflux_ca1_COz^2)	mol/(m²·s)	Diffusive flux magnitude	Domain 1	
tds2.tfluxMag_ca1_CO	sqrt(tds2.tflux_ca1_COx^2+tds2.tflux_ca1_COy^2+tds2.tflux_ca1_COz^2)	mol/(m²·s)	Total flux magnitude	Domain 1	
tds2.dpflux_ca1_COx	0	mol/(m²·s)	Dispersive flux, x component	Domain 1	
tds2.dpflux_ca1_COy	0	mol/(m²·s)	Dispersive flux, y component	Domain 1	
tds2.dpflux_ca1_COz	0	mol/(m²·s)	Dispersive flux, z component	Domain 1	
tds2.dflux_ca1_O2x	-tds2.D_ca1_O2xx*ca1_O2x	mol/(m²·s)	Diffusive flux, x component	Domain 1	+ operation
tds2.dflux_ca1_O2y	-tds2.D_ca1_O2yx*ca1_O2x	mol/(m²·s)	Diffusive flux, y component	Domain 1	+ operation
tds2.dflux_ca1_O2z	-tds2.D_ca1_O2zx*ca1_O2x	mol/(m²·s)	Diffusive flux, z component	Domain 1	+ operation
tds2.grad_ca1_O2x	ca1_O2x	mol/m⁴	Concentration gradient, x component	Domain 1	
tds2.grad_ca1_O2y	0	mol/m⁴	Concentration gradient, y component	Domain 1	
tds2.grad_ca1_O2z	0	mol/m⁴	Concentration gradient, z component	Domain 1	
tds2.dflux_ca1_CO2x	-tds2.D_ca1_CO2xx*ca1_CO2x	mol/(m²·s)	Diffusive flux, x component	Domain 1	+ operation
tds2.dflux_ca1_CO2	-	mol/(m²·s)	Diffusive flux, y	Domain 1	+ operation

Name	Expression	Unit	Description	Selection	Details
y	tds2.D_ca1_CO2yx*ca1_CO2x		component		
tds2.dflux_ca1_CO2z	-tds2.D_ca1_CO2zx*ca1_CO2x	mol/(m²·s)	Diffusive flux, z component	Domain 1	+ operation
tds2.grad_ca1_CO2x	ca1_CO2x	mol/m⁴	Concentration gradient, x component	Domain 1	
tds2.grad_ca1_CO2y	0	mol/m⁴	Concentration gradient, y component	Domain 1	
tds2.grad_ca1_CO2z	0	mol/m⁴	Concentration gradient, z component	Domain 1	
tds2.dflux_ca1_COx	-tds2.D_ca1_COxx*ca1_COx	mol/(m²·s)	Diffusive flux, x component	Domain 1	+ operation
tds2.dflux_ca1_COy	-tds2.D_ca1_COyx*ca1_COx	mol/(m²·s)	Diffusive flux, y component	Domain 1	+ operation
tds2.dflux_ca1_COz	-tds2.D_ca1_COzx*ca1_COx	mol/(m²·s)	Diffusive flux, z component	Domain 1	+ operation
tds2.grad_ca1_COx	ca1_COx	mol/m⁴	Concentration gradient, x component	Domain 1	
tds2.grad_ca1_COy	0	mol/m⁴	Concentration gradient, y component	Domain 1	
tds2.grad_ca1_COz	0	mol/m⁴	Concentration gradient, z component	Domain 1	
tds2.bndFlux_ca1_O2	-uflux_spatial(ca1_O2)/tds2.d	mol/(m²·s)	Boundary flux	Boundary 1	Meta
tds2.bndFlux_ca1_O2	-dflux_spatial(ca1_O2)/tds2.d	mol/(m²·s)	Boundary flux	Boundary 2	Meta
tds2.bndFlux_ca1_CO	-uflux_spatial(ca1_CO)/tds2.d	mol/(m²·s)	Boundary flux	Boundary 1	Meta
tds2.bndFlux_ca1_CO	-dflux_spatial(ca1_C	mol/(m²·s)	Boundary flux	Boundary 2	Meta

Name	Expression	Unit	Description	Selection	Details
	O)/tds2.d				
tds2.Res_ca1_O2	d(ca1_O2,t)-tds2.R_ca1_O2	mol/(m³·s)	Equation residual	Domain 1	
tds2.Rlin_ca1_O2	0		Linear source term coefficient	Domain 1	
tds2.Res_ca1_CO2	d(ca1_CO2,t)-tds2.R_ca1_CO2	mol/(m³·s)	Equation residual	Domain 1	
tds2.Rlin_ca1_CO2	0		Linear source term coefficient	Domain 1	
tds2.Res_ca1_CO	d(ca1_CO,t)-tds2.R_ca1_CO	mol/(m³·s)	Equation residual	Domain 1	
tds2.Rlin_ca1_CO	0		Linear source term coefficient	Domain 1	

Shape functions

Name	Shape function	Unit	Description	Shape frame	Selection
ca1_O2	Lagrange (Linear)	mol/m³	Concentration	Spatial	Domain 1
ca1_CO2	Lagrange (Linear)	mol/m³	Concentration	Spatial	Domain 1
ca1_CO	Lagrange (Linear)	mol/m³	Concentration	Spatial	Domain 1

Weak Expressions

Weak expression	Integration order	Integration frame	Selection
(-ca1_O2t*test(ca1_O2)+tds2.dflux_ca1_O2x*test(ca1_O2x))*tds2.d	2	Spatial	Domain 1
(-ca1_CO2t*test(ca1_CO2)+tds2.dflux_ca1_CO2x*test(ca1_CO2x))*tds2.d	2	Spatial	Domain 1
(-ca1_COt*test(ca1_CO)+tds2.dflux_ca1_COx*test(ca1_COx))*tds2.d	2	Spatial	Domain 1
tds2.streamline*(isScalingSystemDomain==0)*tds2.d	2	Spatial	Domain 1
tds2.crosswind*(isScalingSystemDomain==0)*tds2.d	4	Spatial	Domain 1

2.5.4 No Flux 1

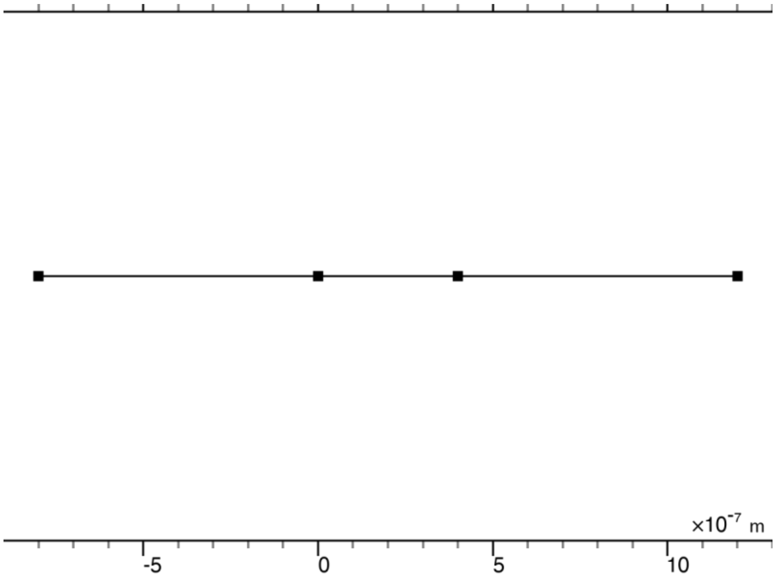

No Flux 1

SELECTION

Geometric entity level	Boundary
Selection	Geometry geom1: Dimension 0: All boundaries

EQUATIONS

$$-\mathbf{n}\cdot\mathbf{J}_i=0$$

2.5.5 Initial Values 1

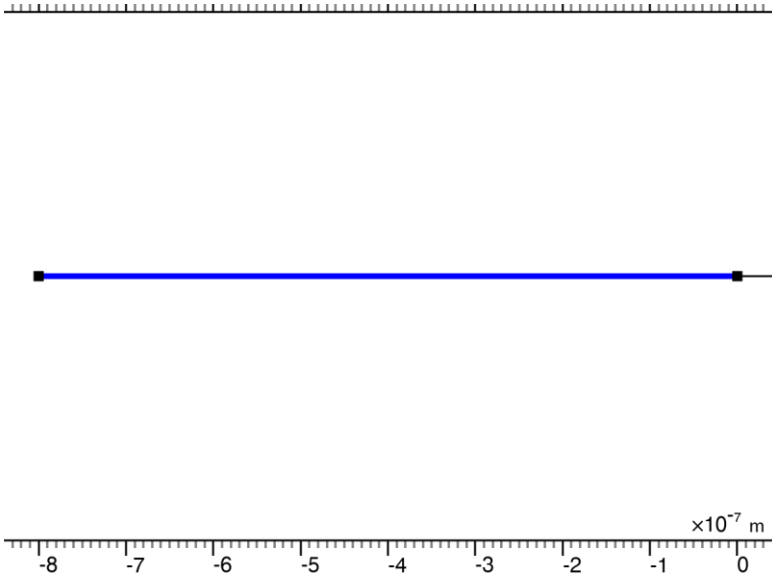

Initial Values 1

SELECTION

Geometric entity level	Domain
Selection	Geometry geom1: Dimension 1: All domains

Initial Values

SETTINGS

Description	Value
Concentration	{1e-10, c0, 1e-10}

Variables

Name	Expression	Unit	Description	Selection	Details
tds2.c0_ca1_O2	1.0E-10	mol/m³	Concentration	Domain 1	+ operation
tds2.c0_ca1_CO2	c0	mol/m³	Concentration	Domain 1	+ operation
tds2.c0_ca1_CO	1.0E-10	mol/m³	Concentration	Domain 1	+ operation

2.5.6 Flux 1

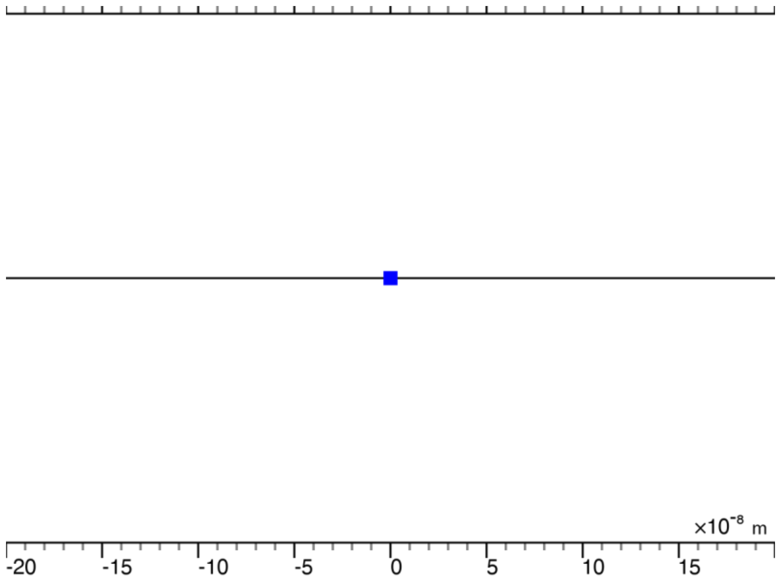

Flux 1

SELECTION

Geometric entity level	Boundary
Selection	Geometry geom1: Dimension 0: Boundary 2

EQUATIONS

$$-\mathbf{n}\cdot\mathbf{J}_i = J_{0,i}$$

Inward Flux

SETTINGS

Description	Value
Flux type	General inward flux
Species ca1_O2	On
Species ca1_CO2	On
Species ca1_CO	On
	{k_O2*(cO2 - (ca1_O2*H_O2)), k_CO2*(cCO2 - (ca1_CO2*H_CO2)), k_CO*(cCO - (ca1_CO*H_CO))}

Variables

Name	Expression	Unit	Description	Selection
tds2.fl1.nmflow_ca1_O2	tds2.fl1.int(tds2.ntflux_ca1_O2)*tds2.d	mol/s	Normal molar flow rate	Global
tds2.fl1.nmflow_ca1_CO2	tds2.fl1.int(tds2.ntflux_ca1_CO2)*tds2.d	mol/s	Normal molar flow rate	Global
tds2.fl1.nmflow_ca1_CO	tds2.fl1.int(tds2.ntflux_ca1_CO)*tds2.d	mol/s	Normal molar flow rate	Global

Weak Expressions

Weak expression	Integration order	Integration frame	Selection
k_O2*(cO2-ca1_O2*H_O2)*test(ca1_O2)*tds2.d	2	Spatial	Boundary 2
k_CO2*(cCO2-ca1_CO2*H_CO2)*test(ca1_CO2)*tds2.d	2	Spatial	Boundary 2
k_CO*(cCO-ca1_CO*H_CO)*test(ca1_CO)*tds2.d	2	Spatial	Boundary 2

2.5.7 Concentration 1

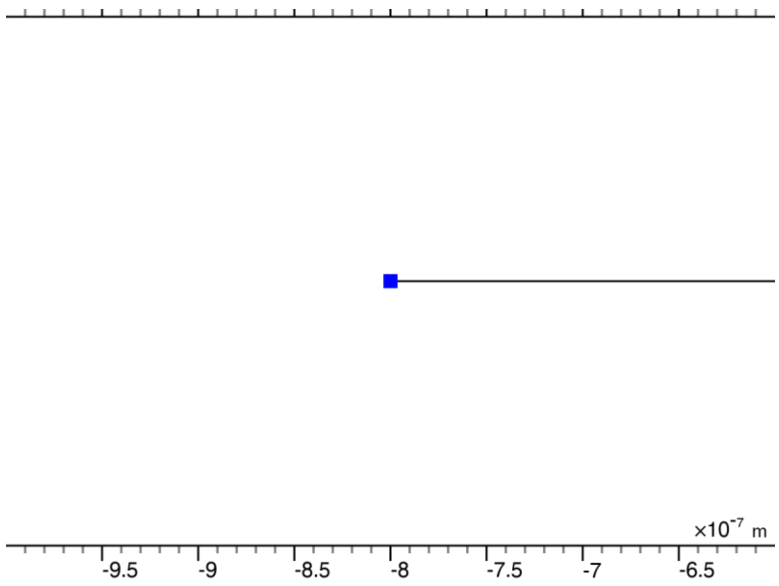

Concentration 1

SELECTION

Geometric entity level	Boundary
Selection	Geometry geom1: Dimension 0: Boundary 1

EQUATIONS

$$c_i = c_{0,j}$$

SETTINGS

**Description**	**Value**
Concentration	{0, c0, 0}

Concentration

SETTINGS

**Description**	**Value**
Species ca1_O2	Off
Species ca1_CO2	On
Species ca1_CO	Off

Constraint Settings

SETTINGS

**Description**	**Value**
Apply reaction terms on	All physics (symmetric)
Use weak constraints	Off

Description	Value
Constraint method	Elemental

Variables

Name	Expression	Unit	Description	Selection	Details
tds2.c0_ca1_CO2	c0	mol/m^3	Concentration	Boundary 1	+ operation
tds2.conc1.nmflow_ca1_O2	tds2.conc1.int(tds2.ntflux_ca1_O2)*tds2.d	mol/s	Normal molar flow rate	Global	
tds2.conc1.nmflow_ca1_CO2	tds2.conc1.int(tds2.ntflux_ca1_CO2)*tds2.d	mol/s	Normal molar flow rate	Global	
tds2.conc1.nmflow_ca1_CO	tds2.conc1.int(tds2.ntflux_ca1_CO)*tds2.d	mol/s	Normal molar flow rate	Global	

Constraints

Constraint	Constraint force	Shape function	Selection	Details
0	0	Lagrange (Linear)	Boundary 1	Elemental
-tds2.cVar_ca1_CO2+tds2.c0_ca1_CO2	test(-tds2.cVar_ca1_CO2+tds2.c0_ca1_CO2)	Lagrange (Linear)	Boundary 1	Elemental
0	0	Lagrange (Linear)	Boundary 1	Elemental

2.5.8 Surface Reactions 1

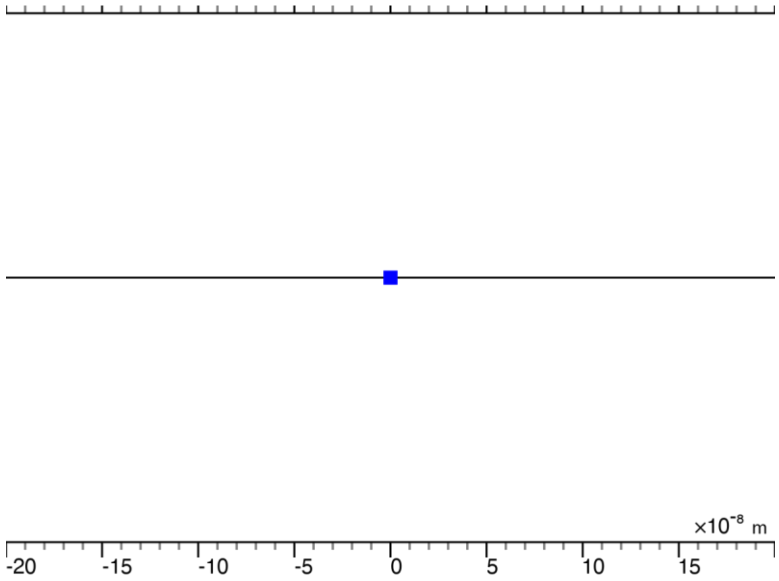

Surface Reactions 1

SELECTION

Geometric entity level	Boundary
Selection	Geometry geom1: Dimension 0: Boundary 2

EQUATIONS

$$-\mathbf{n}\cdot\mathbf{J}_i = J_{0,i}$$

Surface Reaction Rate

SETTINGS

Description	Value
Surface reaction rate	User defined
Surface reaction rate	0
Surface reaction rate	Surface reaction rate for species CO2 (chem)
Surface reaction rate	Surface reaction rate for species CO (chem)

Variables

Name	Expression	Unit	Description	Selection	Details
tds2.J0_ca1_O2	model.input.J0_ca1_O2	mol/(m²·s)	Surface reaction rate	Boundary 2	Meta, + operation
tds2.J0_ca1_CO2	model.input.J0_ca1_CO2	mol/(m²·s)	Surface reaction rate	Boundary 2	Meta, + operation
tds2.J0_ca1_CO	model.input.J0_ca1_CO	mol/(m²·s)	Surface reaction rate	Boundary 2	Meta, + operation

Weak Expressions

Weak expression	Integration order	Integration frame	Selection
tds2.J0_ca1_O2*test(ca1_O2)*tds2.d	2	Spatial	Boundary 2
tds2.J0_ca1_CO2*test(ca1_CO2)*tds2.d	2	Spatial	Boundary 2
tds2.J0_ca1_CO*test(ca1_CO)*tds2.d	2	Spatial	Boundary 2

2.6 TRANSPORT OF DILUTED SPECIES H2O SIDE

USED PRODUCTS

COMSOL Multiphysics
Chemical Reaction Engineering Module

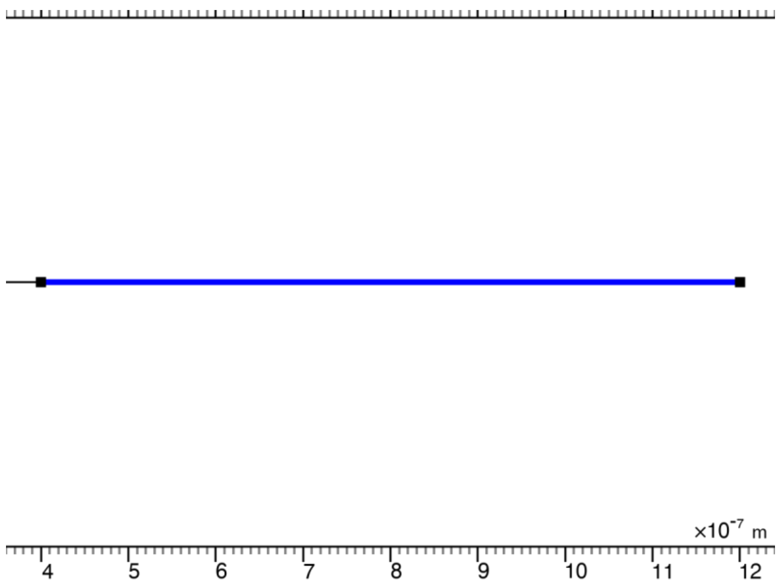

Transport of Diluted Species H2O side

SELECTION

Geometric entity level	Domain
Selection	Geometry geom1: Dimension 1: Domain 3

EQUATIONS

$$\frac{\partial c_i}{\partial t} + \nabla \cdot \mathbf{J}_i = R_i$$
$$\mathbf{J}_i = -D_i \nabla c_i$$

2.6.1 Interface Settings

Discretization

SETTINGS

Description	Value
Concentration	Linear

SETTINGS

Description	Value
Equation form	Study controlled

Cross-Sectional Area

SETTINGS

Description	Value
Cross-sectional area	1[m^2]

Consistent Stabilization

SETTINGS

Description	Value
Streamline diffusion	On
Crosswind diffusion	On
Equation residual	Approximate residual
Crosswind diffusion type	Do Carmo and Galeão

Advanced Settings

SETTINGS

Description	Value
Convective term	Nonconservative form

Transport Mechanisms

SETTINGS

Description	Value
Convection	Off
Migration in electric field	Off
Mass transfer in porous media	Off

2.6.2 Variables

Name	Expression	Unit	Description	Selection	Details
tds3.Ac	1[m^2]	m^2	Cross-sectional area	Global	
tds3.d	tds3.Ac	m^2	Out-of-plane geometry extension	Global	
tds3.nx	unx	1	Normal vector, x component	Boundary 3	
tds3.ny	0	1	Normal vector, y component	Boundary 3	
tds3.nz	0	1	Normal vector, z component	Boundary 3	
tds3.nx	dnx	1	Normal vector, x component	Boundary 4	
tds3.ny	0	1	Normal vector, y component	Boundary 4	
tds3.nz	0	1	Normal vector, z component	Boundary 4	
tds3.nxmesh	unxmesh	1	Normal vector (mesh), x	Boundary 3	

Name	Expression	Unit	Description	Selection	Details
			component		
tds3.nymesh	0	1	Normal vector (mesh), y component	Boundary 3	
tds3.nzmesh	0	1	Normal vector (mesh), z component	Boundary 3	
tds3.nxmesh	dnxmesh	1	Normal vector (mesh), x component	Boundary 4	
tds3.nymesh	0	1	Normal vector (mesh), y component	Boundary 4	
tds3.nzmesh	0	1	Normal vector (mesh), z component	Boundary 4	
tds3.nxc	-root.nxc/tds3.ncLen	1	Normal vector, x component	Boundary 3	
tds3.nyc	0	1	Normal vector, y component	Boundary 3	
tds3.nzc	0	1	Normal vector, z component	Boundary 3	
tds3.nxc	root.nxc/tds3.ncLen	1	Normal vector, x component	Boundary 4	
tds3.nyc	0	1	Normal vector, y component	Boundary 4	
tds3.nzc	0	1	Normal vector, z component	Boundary 4	
tds3.R_ca2_O2	0	mol/(m^3·s)	Total rate expression	Domain 3	+ operation
tds3.cP_ca2_O2	0	mol/kg	Concentration species adsorbed to the solid	Domain 3	+ operation
tds3.cP_ca2_O2	0	mol/kg	Concentration species adsorbed to the solid	Boundaries 3–4	+ operation
tds3.KP_ca2_O2	0	m^3/kg	Adsorption isotherm, first concentration derivative	Domain 3	+ operation
tds3.KP_ca2_O2	0	m^3/kg	Adsorption isotherm, first concentration	Boundaries 3–4	+ operation

Name	Expression	Unit	Description	Selection	Details
			derivative		
tds3.Rads_ca2_O2	0	mol/(m³·s)	Total adsorption rate	Domain 3	+ operation
tds3.DiT_ca2_O2	0	m²/s	Turbulent diffusivity	Domain 3	
tds3.cVar_ca2_O2	ca2_O2	mol/m³	Species	Boundaries 3–4	
tds3.R_ca2_CO2	0	mol/(m³·s)	Total rate expression	Domain 3	+ operation
tds3.cP_ca2_CO2	0	mol/kg	Concentration species adsorbed to the solid	Domain 3	+ operation
tds3.cP_ca2_CO2	0	mol/kg	Concentration species adsorbed to the solid	Boundaries 3–4	+ operation
tds3.KP_ca2_CO2	0	m³/kg	Adsorption isotherm, first concentration derivative	Domain 3	+ operation
tds3.KP_ca2_CO2	0	m³/kg	Adsorption isotherm, first concentration derivative	Boundaries 3–4	+ operation
tds3.Rads_ca2_CO2	0	mol/(m³·s)	Total adsorption rate	Domain 3	+ operation
tds3.DiT_ca2_CO2	0	m²/s	Turbulent diffusivity	Domain 3	
tds3.cVar_ca2_CO2	ca2_CO2	mol/m³	Species	Boundaries 3–4	
tds3.R_ca2_CO	0	mol/(m³·s)	Total rate expression	Domain 3	+ operation
tds3.cP_ca2_CO	0	mol/kg	Concentration species adsorbed to the solid	Domain 3	+ operation
tds3.cP_ca2_CO	0	mol/kg	Concentration species adsorbed to the solid	Boundaries 3–4	+ operation
tds3.KP_ca2_CO	0	m³/kg	Adsorption isotherm, first concentration derivative	Domain 3	+ operation
tds3.KP_ca2_CO	0	m³/kg	Adsorption isotherm, first concentration derivative	Boundaries 3–4	+ operation

Name	Expression	Unit	Description	Selection	Details
tds3.Rads_ca2_CO	0	$mol/(m^3 \cdot s)$	Total adsorption rate	Domain 3	+ operation
tds3.DiT_ca2_CO	0	m^2/s	Turbulent diffusivity	Domain 3	
tds3.cVar_ca2_CO	ca2_CO	mol/m^3	Species	Boundaries 3–4	
tds3.poro	1	1	Porosity	Domain 3	
tds3.theta_g	0	1	Gas volume fraction	Domain 3	
tds3.theta_l	1	1	Liquid volume fraction	Domain 3	
tds3.theta	tds3.poro	1	Mobile fluid volume fraction	Domain 3	

2.6.3 Transport Properties 1

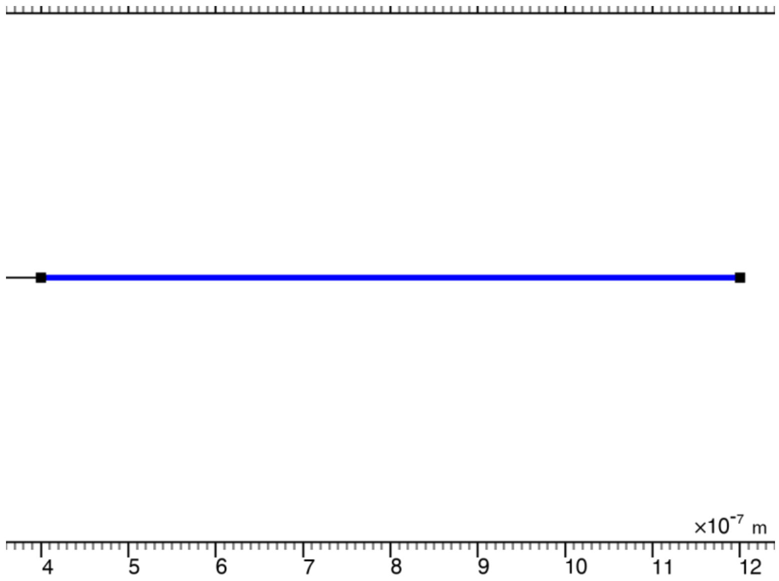

Transport Properties 1

SELECTION

Geometric entity level	Domain
Selection	Geometry geom1: Dimension 1: All domains

EQUATIONS

$$\frac{\partial c_i}{\partial t} + \nabla \cdot \mathbf{J}_i = R_i$$

$$\mathbf{J}_i = -D_i \nabla c_i$$

Diffusion

SETTINGS

Description	Value
Source	Material
Material	None
Diffusion coefficient	User defined
Diffusion coefficient	{{D_O2, 0, 0}, {0, D_O2, 0}, {0, 0, D_O2}}
Diffusion coefficient	User defined
Diffusion coefficient	{{D_CO2, 0, 0}, {0, D_CO2, 0}, {0, 0, D_CO2}}
Diffusion coefficient	User defined
Diffusion coefficient	{{D_CO, 0, 0}, {0, D_CO, 0}, {0, 0, D_CO}}

Coordinate System Selection

SETTINGS

Description	Value
Coordinate system	Global coordinate system

Model Input

SETTINGS

Description	Value
Temperature	Common model input

Variables

Name	Expression	Unit	Description	Selection	Details
domflux.ca2_O2x	tds3.dflux_ca2_O2x*tds3.d	mol/s	Domain flux, x component	Domain 3	
domflux.ca2_CO2x	tds3.dflux_ca2_CO2x*tds3.d	mol/s	Domain flux, x component	Domain 3	
domflux.ca2_COx	tds3.dflux_ca2_COx*tds3.d	mol/s	Domain flux, x component	Domain 3	
tds3.ndflux_ca2_O2	tds3.bndFlux_ca2_O2	mol/(m²·s)	Normal diffusive flux	Boundaries 3–4	
tds3.ntflux_ca2_O2	tds3.bndFlux_ca2_O2	mol/(m²·s)	Normal total flux	Boundaries 3–4	
tds3.ndflux_ca2_CO2	tds3.bndFlux_ca2_CO2	mol/(m²·s)	Normal diffusive flux	Boundaries 3–4	
tds3.ntflux_ca2_CO2	tds3.bndFlux_ca2_CO2	mol/(m²·s)	Normal total flux	Boundaries 3–4	
tds3.ndflux_ca2_CO	tds3.bndFlux_ca2_CO	mol/(m²·s)	Normal diffusive flux	Boundaries 3–4	
tds3.ntflux_ca2_CO	tds3.bndFlux_ca2_CO	mol/(m²·s)	Normal total flux	Boundaries 3–4	

Name	Expression	Unit	Description	Selection	Details
tds3.bndFlux_ca2_CO2	-uflux_spatial(ca2_CO2)/tds3.d	mol/(m²·s)	Boundary flux	Boundary 3	Meta
tds3.bndFlux_ca2_CO2	-dflux_spatial(ca2_CO2)/tds3.d	mol/(m²·s)	Boundary flux	Boundary 4	Meta
tds3.DF_ca2_O2xx	D_O2	m²/s	Fluid diffusion coefficient, xx component	Domain 3	
tds3.DF_ca2_O2yx	0	m²/s	Fluid diffusion coefficient, yx component	Domain 3	
tds3.DF_ca2_O2zx	0	m²/s	Fluid diffusion coefficient, zx component	Domain 3	
tds3.DF_ca2_O2xy	0	m²/s	Fluid diffusion coefficient, xy component	Domain 3	
tds3.DF_ca2_O2yy	D_O2	m²/s	Fluid diffusion coefficient, yy component	Domain 3	
tds3.DF_ca2_O2zy	0	m²/s	Fluid diffusion coefficient, zy component	Domain 3	
tds3.DF_ca2_O2xz	0	m²/s	Fluid diffusion coefficient, xz component	Domain 3	
tds3.DF_ca2_O2yz	0	m²/s	Fluid diffusion coefficient, yz component	Domain 3	
tds3.DF_ca2_O2zz	D_O2	m²/s	Fluid diffusion coefficient, zz component	Domain 3	
tds3.D_ca2_O2xx	tds3.DF_ca2_O2xx+tds3.DiT_ca2_O2	m²/s	Diffusion coefficient, xx component	Domain 3	
tds3.D_ca2_O2yx	tds3.DF_ca2_O2yx	m²/s	Diffusion coefficient, yx component	Domain 3	
tds3.D_ca2_O2zx	tds3.DF_ca2_O2zx	m²/s	Diffusion coefficient, zx component	Domain 3	
tds3.D_ca2_O2xy	tds3.DF_ca2_O2xy	m²/s	Diffusion	Domain 3	

Name	Expression	Unit	Description	Selection	Details
			coefficient, xy component		
tds3.D_ca2_O2yy	tds3.DF_ca2_O2yy+tds3.DiT_ca2_O2	m^2/s	Diffusion coefficient, yy component	Domain 3	
tds3.D_ca2_O2zy	tds3.DF_ca2_O2zy	m^2/s	Diffusion coefficient, zy component	Domain 3	
tds3.D_ca2_O2xz	tds3.DF_ca2_O2xz	m^2/s	Diffusion coefficient, xz component	Domain 3	
tds3.D_ca2_O2yz	tds3.DF_ca2_O2yz	m^2/s	Diffusion coefficient, yz component	Domain 3	
tds3.D_ca2_O2zz	tds3.DF_ca2_O2zz+tds3.DiT_ca2_O2	m^2/s	Diffusion coefficient, zz component	Domain 3	
tds3.DF_ca2_CO2xx	D_CO2	m^2/s	Fluid diffusion coefficient, xx component	Domain 3	
tds3.DF_ca2_CO2yx	0	m^2/s	Fluid diffusion coefficient, yx component	Domain 3	
tds3.DF_ca2_CO2zx	0	m^2/s	Fluid diffusion coefficient, zx component	Domain 3	
tds3.DF_ca2_CO2xy	0	m^2/s	Fluid diffusion coefficient, xy component	Domain 3	
tds3.DF_ca2_CO2yy	D_CO2	m^2/s	Fluid diffusion coefficient, yy component	Domain 3	
tds3.DF_ca2_CO2zy	0	m^2/s	Fluid diffusion coefficient, zy component	Domain 3	
tds3.DF_ca2_CO2xz	0	m^2/s	Fluid diffusion coefficient, xz component	Domain 3	
tds3.DF_ca2_CO2yz	0	m^2/s	Fluid diffusion coefficient, yz component	Domain 3	
tds3.DF_ca2_CO2zz	D_CO2	m^2/s	Fluid diffusion coefficient, zz	Domain 3	

Name	Expression	Unit	Description	Selection	Details
			component		
tds3.D_ca2_CO2xx	tds3.DF_ca2_CO2xx +tds3.DiT_ca2_CO2	m²/s	Diffusion coefficient, xx component	Domain 3	
tds3.D_ca2_CO2yx	tds3.DF_ca2_CO2yx	m²/s	Diffusion coefficient, yx component	Domain 3	
tds3.D_ca2_CO2zx	tds3.DF_ca2_CO2zx	m²/s	Diffusion coefficient, zx component	Domain 3	
tds3.D_ca2_CO2xy	tds3.DF_ca2_CO2xy	m²/s	Diffusion coefficient, xy component	Domain 3	
tds3.D_ca2_CO2yy	tds3.DF_ca2_CO2yy +tds3.DiT_ca2_CO2	m²/s	Diffusion coefficient, yy component	Domain 3	
tds3.D_ca2_CO2zy	tds3.DF_ca2_CO2zy	m²/s	Diffusion coefficient, zy component	Domain 3	
tds3.D_ca2_CO2xz	tds3.DF_ca2_CO2xz	m²/s	Diffusion coefficient, xz component	Domain 3	
tds3.D_ca2_CO2yz	tds3.DF_ca2_CO2yz	m²/s	Diffusion coefficient, yz component	Domain 3	
tds3.D_ca2_CO2zz	tds3.DF_ca2_CO2zz +tds3.DiT_ca2_CO2	m²/s	Diffusion coefficient, zz component	Domain 3	
tds3.DF_ca2_COxx	D_CO	m²/s	Fluid diffusion coefficient, xx component	Domain 3	
tds3.DF_ca2_COyx	0	m²/s	Fluid diffusion coefficient, yx component	Domain 3	
tds3.DF_ca2_COzx	0	m²/s	Fluid diffusion coefficient, zx component	Domain 3	
tds3.DF_ca2_COxy	0	m²/s	Fluid diffusion coefficient, xy component	Domain 3	
tds3.DF_ca2_COyy	D_CO	m²/s	Fluid diffusion coefficient, yy component	Domain 3	

Name	Expression	Unit	Description	Selection	Details
tds3.DF_ca2_COzy	0	m^2/s	Fluid diffusion coefficient, zy component	Domain 3	
tds3.DF_ca2_COxz	0	m^2/s	Fluid diffusion coefficient, xz component	Domain 3	
tds3.DF_ca2_COyz	0	m^2/s	Fluid diffusion coefficient, yz component	Domain 3	
tds3.DF_ca2_COzz	D_CO	m^2/s	Fluid diffusion coefficient, zz component	Domain 3	
tds3.D_ca2_COxx	tds3.DF_ca2_COxx+ tds3.DiT_ca2_CO	m^2/s	Diffusion coefficient, xx component	Domain 3	
tds3.D_ca2_COyx	tds3.DF_ca2_COyx	m^2/s	Diffusion coefficient, yx component	Domain 3	
tds3.D_ca2_COzx	tds3.DF_ca2_COzx	m^2/s	Diffusion coefficient, zx component	Domain 3	
tds3.D_ca2_COxy	tds3.DF_ca2_COxy	m^2/s	Diffusion coefficient, xy component	Domain 3	
tds3.D_ca2_COyy	tds3.DF_ca2_COyy+ tds3.DiT_ca2_CO	m^2/s	Diffusion coefficient, yy component	Domain 3	
tds3.D_ca2_COzy	tds3.DF_ca2_COzy	m^2/s	Diffusion coefficient, zy component	Domain 3	
tds3.D_ca2_COxz	tds3.DF_ca2_COxz	m^2/s	Diffusion coefficient, xz component	Domain 3	
tds3.D_ca2_COyz	tds3.DF_ca2_COyz	m^2/s	Diffusion coefficient, yz component	Domain 3	
tds3.D_ca2_COzz	tds3.DF_ca2_COzz+t ds3.DiT_ca2_CO	m^2/s	Diffusion coefficient, zz component	Domain 3	
tds3.Dav_ca2_O2	tds3.D_ca2_O2xx	m^2/s	Average diffusion coefficient	Domain 3	
tds3.Dav_ca2_CO2	tds3.D_ca2_CO2xx	m^2/s	Average diffusion coefficient	Domain 3	

Name	Expression	Unit	Description	Selection	Details
tds3.Dav_ca2_CO	tds3.D_ca2_COxx	m^2/s	Average diffusion coefficient	Domain 3	
tds3.tflux_ca2_O2x	tds3.dflux_ca2_O2x	$mol/(m^2 \cdot s)$	Total flux, x component	Domain 3	+ operation
tds3.tflux_ca2_O2y	tds3.dflux_ca2_O2y	$mol/(m^2 \cdot s)$	Total flux, y component	Domain 3	+ operation
tds3.tflux_ca2_O2z	tds3.dflux_ca2_O2z	$mol/(m^2 \cdot s)$	Total flux, z component	Domain 3	+ operation
tds3.dfluxMag_ca2_O2	sqrt(tds3.dflux_ca2_O2x^2+tds3.dflux_ca2_O2y^2+tds3.dflux_ca2_O2z^2)	$mol/(m^2 \cdot s)$	Diffusive flux magnitude	Domain 3	
tds3.tfluxMag_ca2_O2	sqrt(tds3.tflux_ca2_O2x^2+tds3.tflux_ca2_O2y^2+tds3.tflux_ca2_O2z^2)	$mol/(m^2 \cdot s)$	Total flux magnitude	Domain 3	
tds3.dpflux_ca2_O2x	0	$mol/(m^2 \cdot s)$	Dispersive flux, x component	Domain 3	
tds3.dpflux_ca2_O2y	0	$mol/(m^2 \cdot s)$	Dispersive flux, y component	Domain 3	
tds3.dpflux_ca2_O2z	0	$mol/(m^2 \cdot s)$	Dispersive flux, z component	Domain 3	
tds3.tflux_ca2_CO2x	tds3.dflux_ca2_CO2x	$mol/(m^2 \cdot s)$	Total flux, x component	Domain 3	+ operation
tds3.tflux_ca2_CO2y	tds3.dflux_ca2_CO2y	$mol/(m^2 \cdot s)$	Total flux, y component	Domain 3	+ operation
tds3.tflux_ca2_CO2z	tds3.dflux_ca2_CO2z	$mol/(m^2 \cdot s)$	Total flux, z component	Domain 3	+ operation
tds3.dfluxMag_ca2_CO2	sqrt(tds3.dflux_ca2_CO2x^2+tds3.dflux_ca2_CO2y^2+tds3.dflux_ca2_CO2z^2)	$mol/(m^2 \cdot s)$	Diffusive flux magnitude	Domain 3	
tds3.tfluxMag_ca2_CO2	sqrt(tds3.tflux_ca2_CO2x^2+tds3.tflux_ca2_CO2y^2+tds3.tflux_ca2_CO2z^2)	$mol/(m^2 \cdot s)$	Total flux magnitude	Domain 3	
tds3.dpflux_ca2_CO2x	0	$mol/(m^2 \cdot s)$	Dispersive flux, x component	Domain 3	
tds3.dpflux_ca2_CO2y	0	$mol/(m^2 \cdot s)$	Dispersive flux, y component	Domain 3	
tds3.dpflux_ca2_CO2z	0	$mol/(m^2 \cdot s)$	Dispersive flux, z component	Domain 3	

Name	Expression	Unit	Description	Selection	Details
tds3.tflux_ca2_COx	tds3.dflux_ca2_COx	mol/(m²·s)	Total flux, x component	Domain 3	+ operation
tds3.tflux_ca2_COy	tds3.dflux_ca2_COy	mol/(m²·s)	Total flux, y component	Domain 3	+ operation
tds3.tflux_ca2_COz	tds3.dflux_ca2_COz	mol/(m²·s)	Total flux, z component	Domain 3	+ operation
tds3.dfluxMag_ca2_CO	sqrt(tds3.dflux_ca2_COx^2+tds3.dflux_ca2_COy^2+tds3.dflux_ca2_COz^2)	mol/(m²·s)	Diffusive flux magnitude	Domain 3	
tds3.tfluxMag_ca2_CO	sqrt(tds3.tflux_ca2_COx^2+tds3.tflux_ca2_COy^2+tds3.tflux_ca2_COz^2)	mol/(m²·s)	Total flux magnitude	Domain 3	
tds3.dpflux_ca2_COx	0	mol/(m²·s)	Dispersive flux, x component	Domain 3	
tds3.dpflux_ca2_COy	0	mol/(m²·s)	Dispersive flux, y component	Domain 3	
tds3.dpflux_ca2_COz	0	mol/(m²·s)	Dispersive flux, z component	Domain 3	
tds3.dflux_ca2_O2x	-tds3.D_ca2_O2xx*ca2_O2x	mol/(m²·s)	Diffusive flux, x component	Domain 3	+ operation
tds3.dflux_ca2_O2y	-tds3.D_ca2_O2yx*ca2_O2x	mol/(m²·s)	Diffusive flux, y component	Domain 3	+ operation
tds3.dflux_ca2_O2z	-tds3.D_ca2_O2zx*ca2_O2x	mol/(m²·s)	Diffusive flux, z component	Domain 3	+ operation
tds3.grad_ca2_O2x	ca2_O2x	mol/m⁴	Concentration gradient, x component	Domain 3	
tds3.grad_ca2_O2y	0	mol/m⁴	Concentration gradient, y component	Domain 3	
tds3.grad_ca2_O2z	0	mol/m⁴	Concentration gradient, z component	Domain 3	
tds3.dflux_ca2_CO2x	-tds3.D_ca2_CO2xx*ca2_CO2x	mol/(m²·s)	Diffusive flux, x component	Domain 3	+ operation
tds3.dflux_ca2_CO2	-	mol/(m²·s)	Diffusive flux, y	Domain 3	+ operation

Name	Expression	Unit	Description	Selection	Details
y	tds3.D_ca2_CO2yx*ca2_CO2x		component		
tds3.dflux_ca2_CO2z	-tds3.D_ca2_CO2zx*ca2_CO2x	mol/(m²·s)	Diffusive flux, z component	Domain 3	+ operation
tds3.grad_ca2_CO2x	ca2_CO2x	mol/m⁴	Concentration gradient, x component	Domain 3	
tds3.grad_ca2_CO2y	0	mol/m⁴	Concentration gradient, y component	Domain 3	
tds3.grad_ca2_CO2z	0	mol/m⁴	Concentration gradient, z component	Domain 3	
tds3.dflux_ca2_COx	-tds3.D_ca2_COxx*ca2_COx	mol/(m²·s)	Diffusive flux, x component	Domain 3	+ operation
tds3.dflux_ca2_COy	-tds3.D_ca2_COyx*ca2_COx	mol/(m²·s)	Diffusive flux, y component	Domain 3	+ operation
tds3.dflux_ca2_COz	-tds3.D_ca2_COzx*ca2_COx	mol/(m²·s)	Diffusive flux, z component	Domain 3	+ operation
tds3.grad_ca2_COx	ca2_COx	mol/m⁴	Concentration gradient, x component	Domain 3	
tds3.grad_ca2_COy	0	mol/m⁴	Concentration gradient, y component	Domain 3	
tds3.grad_ca2_COz	0	mol/m⁴	Concentration gradient, z component	Domain 3	
tds3.bndFlux_ca2_O2	-uflux_spatial(ca2_O2)/tds3.d	mol/(m²·s)	Boundary flux	Boundary 3	Meta
tds3.bndFlux_ca2_O2	-dflux_spatial(ca2_O2)/tds3.d	mol/(m²·s)	Boundary flux	Boundary 4	Meta
tds3.bndFlux_ca2_CO	-uflux_spatial(ca2_CO)/tds3.d	mol/(m²·s)	Boundary flux	Boundary 3	Meta
tds3.bndFlux_ca2_CO	-dflux_spatial(ca2_C	mol/(m²·s)	Boundary flux	Boundary 4	Meta

Name	Expression	Unit	Description	Selection	Details
	O)/tds3.d				
tds3.Res_ca2_O2	d(ca2_O2,t)-tds3.R_ca2_O2	mol/(m³·s)	Equation residual	Domain 3	
tds3.Rlin_ca2_O2	0		Linear source term coefficient	Domain 3	
tds3.Res_ca2_CO2	d(ca2_CO2,t)-tds3.R_ca2_CO2	mol/(m³·s)	Equation residual	Domain 3	
tds3.Rlin_ca2_CO2	0		Linear source term coefficient	Domain 3	
tds3.Res_ca2_CO	d(ca2_CO,t)-tds3.R_ca2_CO	mol/(m³·s)	Equation residual	Domain 3	
tds3.Rlin_ca2_CO	0		Linear source term coefficient	Domain 3	

Shape functions

Name	Shape function	Unit	Description	Shape frame	Selection
ca2_O2	Lagrange (Linear)	mol/m³	Concentration	Spatial	Domain 3
ca2_CO2	Lagrange (Linear)	mol/m³	Concentration	Spatial	Domain 3
ca2_CO	Lagrange (Linear)	mol/m³	Concentration	Spatial	Domain 3

Weak Expressions

Weak expression	Integration order	Integration frame	Selection
(-ca2_O2t*test(ca2_O2)+tds3.dflux_ca2_O2x*test(ca2_O2x))*tds3.d	2	Spatial	Domain 3
(-ca2_CO2t*test(ca2_CO2)+tds3.dflux_ca2_CO2x*test(ca2_CO2x))*tds3.d	2	Spatial	Domain 3
(-ca2_COt*test(ca2_CO)+tds3.dflux_ca2_COx*test(ca2_COx))*tds3.d	2	Spatial	Domain 3
tds3.streamline*(isScalingSystemDomain==0)*tds3.d	2	Spatial	Domain 3
tds3.crosswind*(isScalingSystemDomain==0)*tds3.d	4	Spatial	Domain 3

2.6.4 No Flux 1

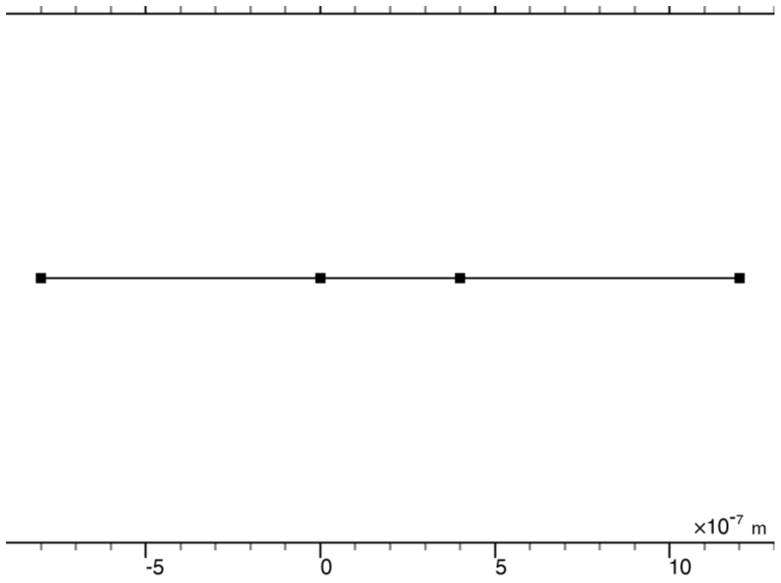

No Flux 1

SELECTION

Geometric entity level	Boundary
Selection	Geometry geom1: Dimension 0: All boundaries

EQUATIONS

$$-\mathbf{n}\cdot\mathbf{J}_i=0$$

2.6.5 Initial Values 1

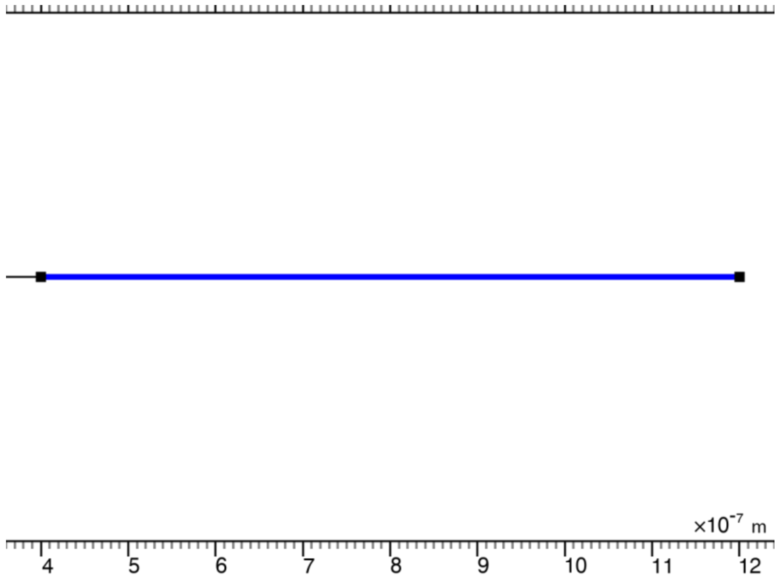

Initial Values 1

SELECTION

Geometric entity level	Domain
Selection	Geometry geom1: Dimension 1: All domains

Initial Values

SETTINGS

Description	Value
Concentration	{1e-10, c0, 1e-10}

Variables

Name	Expression	Unit	Description	Selection	Details
tds3.c0_ca2_O2	1.0E-10	mol/m³	Concentration	Domain 3	+ operation
tds3.c0_ca2_CO2	c0	mol/m³	Concentration	Domain 3	+ operation
tds3.c0_ca2_CO	1.0E-10	mol/m³	Concentration	Domain 3	+ operation

2.6.6 Flux 1

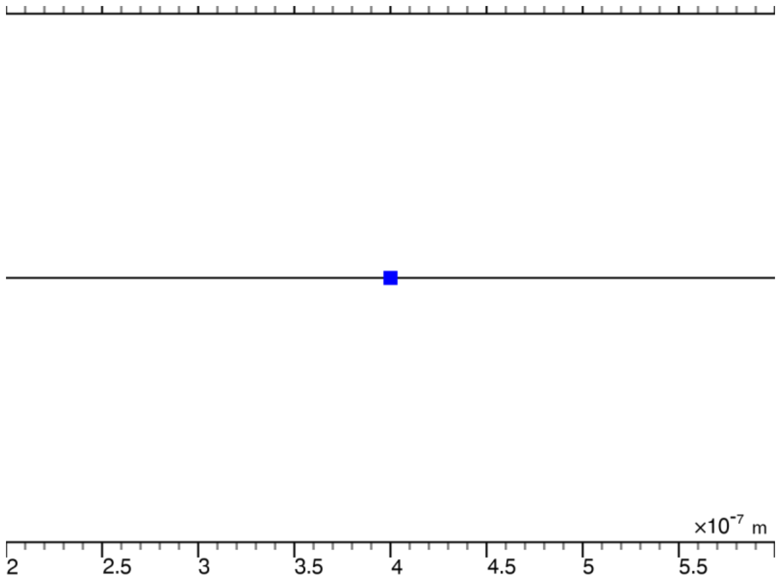

Flux 1

SELECTION

Geometric entity level	Boundary
Selection	Geometry geom1: Dimension 0: Boundary 3

EQUATIONS

$$-\mathbf{n}\cdot\mathbf{J}_i = J_{0,i}$$

Inward Flux

SETTINGS

Description	Value
Flux type	General inward flux
Species ca2_O2	On
Species ca2_CO2	On
Species ca2_CO	On
	{k_O2*(cO2 - (ca2_O2*H_O2)), k_CO2*(cCO2 - (ca2_CO2*H_CO2)), k_CO*(cCO - (ca2_CO*H_CO))}

Variables

Name	Expression	Unit	Description	Selection
tds3.fl1.nmflow_ca2_O2	tds3.fl1.int(tds3.ntflux_ca2_O2)*tds3.d	mol/s	Normal molar flow rate	Global
tds3.fl1.nmflow_ca2_CO2	tds3.fl1.int(tds3.ntflux_ca2_CO2)*tds3.d	mol/s	Normal molar flow rate	Global
tds3.fl1.nmflow_ca2_CO	tds3.fl1.int(tds3.ntflux_ca2_CO)*tds3.d	mol/s	Normal molar flow rate	Global

Weak Expressions

Weak expression	Integration order	Integration frame	Selection
k_O2*(cO2-ca2_O2*H_O2)*test(ca2_O2)*tds3.d	2	Spatial	Boundary 3
k_CO2*(cCO2-ca2_CO2*H_CO2)*test(ca2_CO2)*tds3.d	2	Spatial	Boundary 3
k_CO*(cCO-ca2_CO*H_CO)*test(ca2_CO)*tds3.d	2	Spatial	Boundary 3

2.6.7 Concentration 1

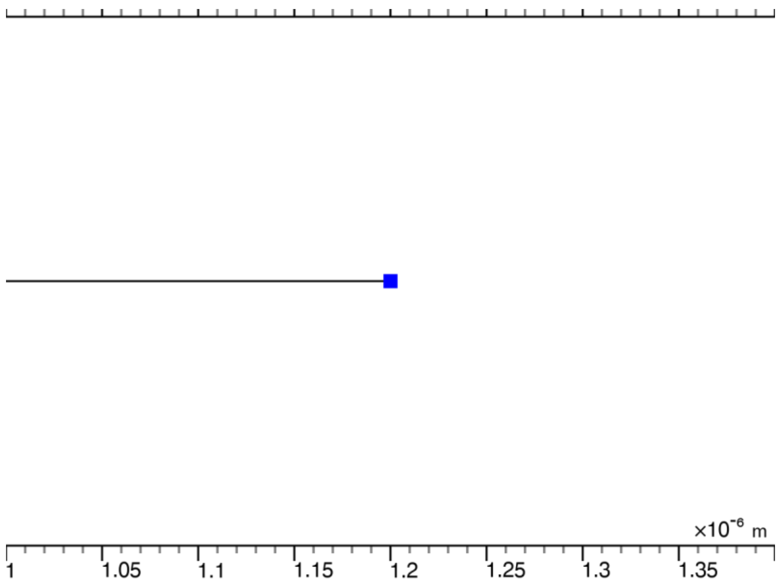

Concentration 1

SELECTION

Geometric entity level	Boundary
Selection	Geometry geom1: Dimension 0: Boundary 4

EQUATIONS

$$c_i = c_{0,j}$$

SETTINGS

Description	Value
Concentration	{0, c0, 0}

Concentration

SETTINGS

Description	Value
Species ca2_O2	Off
Species ca2_CO2	On
Species ca2_CO	Off

Constraint Settings

SETTINGS

Description	Value
Apply reaction terms on	All physics (symmetric)
Use weak constraints	Off

Description	Value
Constraint method	Elemental

Variables

Name	Expression	Unit	Description	Selection	Details
tds3.c0_ca2_CO2	c0	mol/m^3	Concentration	Boundary 4	+ operation
tds3.conc1.nmflow_ca2_O2	tds3.conc1.int(tds3.ntflux_ca2_O2)*tds3.d	mol/s	Normal molar flow rate	Global	
tds3.conc1.nmflow_ca2_CO2	tds3.conc1.int(tds3.ntflux_ca2_CO2)*tds3.d	mol/s	Normal molar flow rate	Global	
tds3.conc1.nmflow_ca2_CO	tds3.conc1.int(tds3.ntflux_ca2_CO)*tds3.d	mol/s	Normal molar flow rate	Global	

Constraints

Constraint	Constraint force	Shape function	Selection	Details
0	0	Lagrange (Linear)	Boundary 4	Elemental
-tds3.cVar_ca2_CO2+tds3.c0_ca2_CO2	test(-tds3.cVar_ca2_CO2+tds3.c0_ca2_CO2)	Lagrange (Linear)	Boundary 4	Elemental
0	0	Lagrange (Linear)	Boundary 4	Elemental

2.6.8 Surface Reactions 1

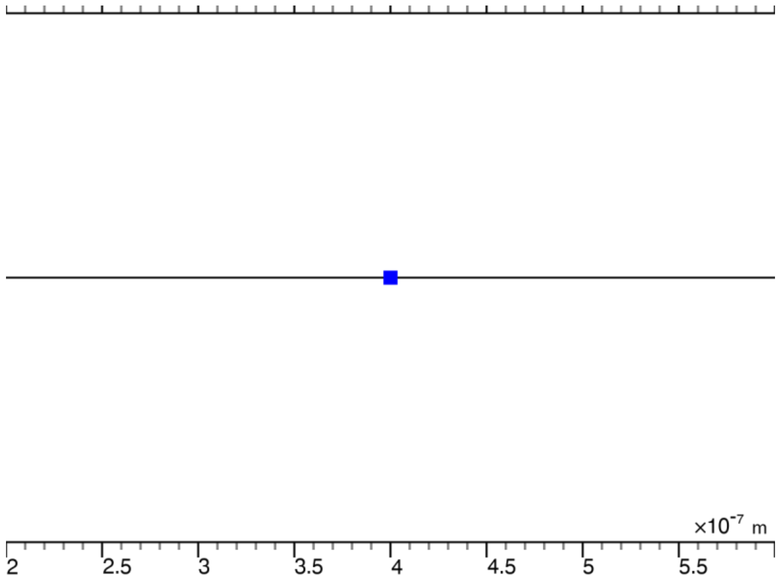

Surface Reactions 1

SELECTION

Geometric entity level	Boundary
Selection	Geometry geom1: Dimension 0: Boundary 3

EQUATIONS

$$-\mathbf{n}\cdot\mathbf{J}_i = J_{0,i}$$

Surface Reaction Rate

SETTINGS

Description	Value
Surface reaction rate	Surface reaction rate for species O2 (chem2)
Surface reaction rate	User defined
Surface reaction rate	0
Surface reaction rate	User defined
Surface reaction rate	0

Variables

Name	Expression	Unit	Description	Selection	Details
tds3.J0_ca2_O2	model.input.J0_ca2_O2	mol/(m²·s)	Surface reaction rate	Boundary 3	Meta, + operation
tds3.J0_ca2_CO2	model.input.J0_ca2_CO2	mol/(m²·s)	Surface reaction rate	Boundary 3	Meta, + operation
tds3.J0_ca2_CO	model.input.J0_ca2_CO	mol/(m²·s)	Surface reaction rate	Boundary 3	Meta, + operation

Weak Expressions

Weak expression	Integration order	Integration frame	Selection
tds3.J0_ca2_O2*test(ca2_O2)*tds3.d	2	Spatial	Boundary 3
tds3.J0_ca2_CO2*test(ca2_CO2)*tds3.d	2	Spatial	Boundary 3
tds3.J0_ca2_CO*test(ca2_CO)*tds3.d	2	Spatial	Boundary 3

2.7 SURFACE REACTIONS CO2 REDUCTION

USED PRODUCTS

COMSOL Multiphysics
Chemical Reaction Engineering Module

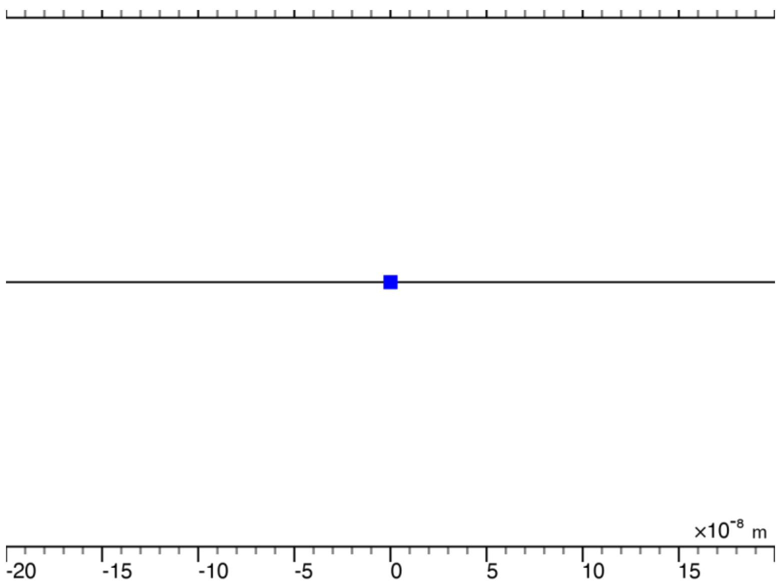

Surface Reactions CO2 reduction

SELECTION

Geometric entity level	Boundary
Selection	Geometry geom1: Dimension 0: Boundary 2

EQUATIONS

$$\frac{\partial c_{s,i}}{\partial t} = R_{s,i}$$

$$\theta_i = \frac{c_{s,i}\sigma_i}{\Gamma_s}$$

$$\frac{\partial c_{b,i}}{\partial t} = R_{b,i}$$

2.7.1 Interface Settings

Discretization

SETTINGS

Description	Value
Surface concentration	Linear
Bulk concentration	Linear

SETTINGS

Description	Value
Equation form	Study controlled

2.7.2 Surface Properties 1

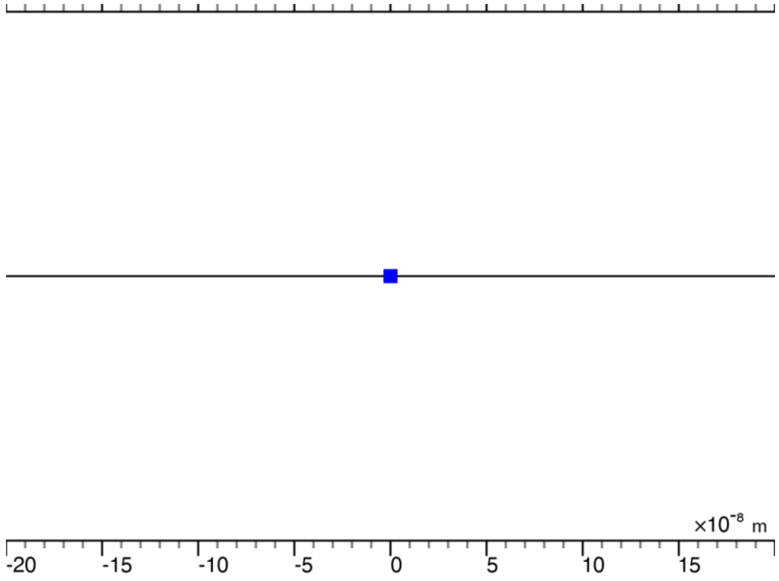

Surface Properties 1

SELECTION

Geometric entity level	Boundary
Selection	Geometry geom1: Dimension 0: All boundaries

EQUATIONS

$$\frac{\partial c_{s,j}}{\partial t} = R_{s,j}$$

$$\theta_i = \frac{c_{s,j}\sigma_i}{\Gamma_s}$$

Sites

SETTINGS

Description	Value
Density of sites	2e-5[mol/m^2]
Site occupancy number	{1, 1}

Variables

Name	Expression	Unit	Description	Selection	Details
sr.Rs_csCO2	0	mol/(m²·s)	Reaction rate for surface species	Boundary 2	+ operation
sr.Rs_csSr	0	mol/(m²·s)	Reaction rate for surface species	Boundary 2	+ operation

Name	Expression	Unit	Description	Selection	Details
sr.Gamma	2.0E-5[mol/m^2]	mol/m²	Density of sites	Boundary 2	
sr.Sigma_csCO2	1	1	Site occupancy number	Boundary 2	
sr.Sigma_csSr	1	1	Site occupancy number	Boundary 2	
sr.theta_i_csCO2	max(eps^2,sr.Sigma_csCO2*csCO2/sr.Gamma)	1	Surface coverage	Boundary 2	
sr.theta_i0	sr.theta_i_csCO2+sr.theta_i_csSr	1	Surface coverage	Boundary 2	+ operation
sr.theta_i_csSr	max(eps^2,sr.Sigma_csSr*csSr/sr.Gamma)	1	Surface coverage	Boundary 2	
sr.theta_free	max(eps^2,min(1,1-sr.theta_i0))	1	Fraction of free sites	Boundary 2	

Shape functions

Name	Shape function	Unit	Description	Shape frame	Selection
csCO2	Lagrange (Linear)	mol/m²	Surface concentration	Spatial	Boundary 2
csCO2	Lagrange (Linear)	mol/m²	Surface concentration	Spatial	Boundary 2
csSr	Lagrange (Linear)	mol/m²	Surface concentration	Spatial	Boundary 2
csSr	Lagrange (Linear)	mol/m²	Surface concentration	Spatial	Boundary 2

Weak Expressions

Weak expression	Integration order	Integration frame	Selection
-d(csCO2,t)*test(csCO2)	2	Spatial	Boundary 2
-d(csSr,t)*test(csSr)	2	Spatial	Boundary 2

2.7.3 Initial Values 1

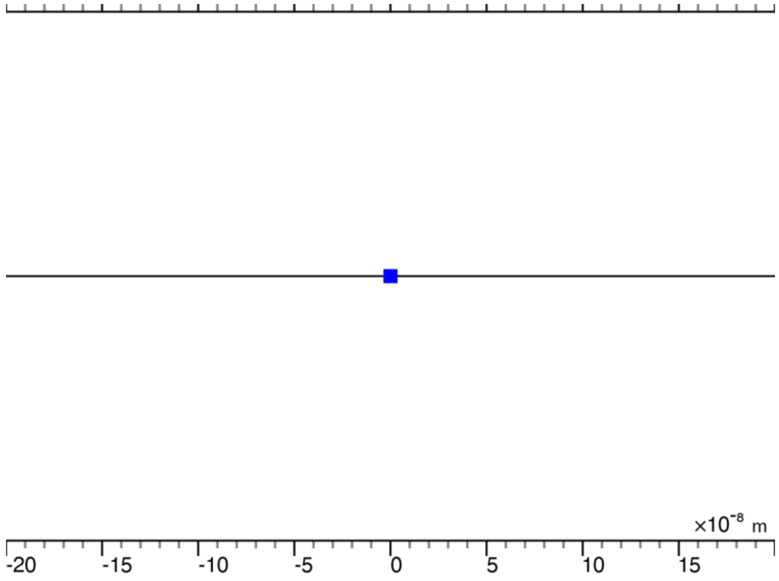

Initial Values 1

SELECTION

Geometric entity level	Boundary
Selection	Geometry geom1: Dimension 0: All boundaries

Initial Values

SETTINGS

Description	Value
Surface concentration	{Gamma_sCO2*(k_adsCO2/k_desCO2)*c0/(1 + (k_adsCO2/k_desCO2)*c0), 1}

2.7.4 Reactions 1

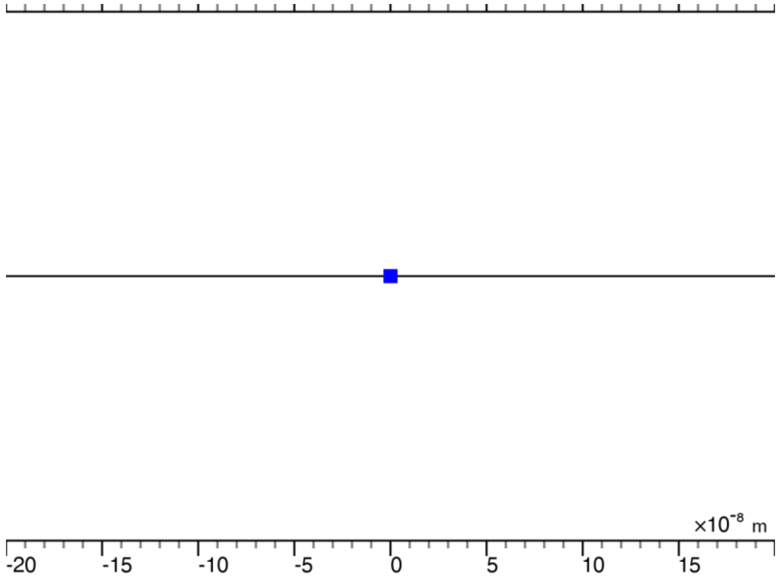

Reactions 1

SELECTION

Geometric entity level	Boundary
Selection	Geometry geom1: Dimension 0: Boundary 2

EQUATIONS

$$\frac{\partial c_{s,i}}{\partial t} = R_{s,i}$$

Reaction Rate for Surface Species

SETTINGS

Description	Value
Reaction rate for surface species	Surface reaction rate for surface species CO2_surf (chem)
Reaction rate for surface species	User defined
Reaction rate for surface species	0

Variables

Name	Expression	Unit	Description	Selection	Details
sr.Rs_csCO2	model.input.Rs_csCO2	mol/(m²·s)	Reaction rate for surface species	Boundary 2	Meta, + operation
sr.Rs_csSr	model.input.Rs_csSr	mol/(m²·s)	Reaction rate for surface species	Boundary 2	Meta, + operation

Weak Expressions

Weak expression	Integration order	Integration frame	Selection
model.input.Rs_csCO2*test(csCO2)	2	Spatial	Boundary 2
model.input.Rs_csSr*test(csSr)	2	Spatial	Boundary 2

2.8 SURFACE REACTIONS H2O OXIDATION

USED PRODUCTS

COMSOL Multiphysics
Chemical Reaction Engineering Module

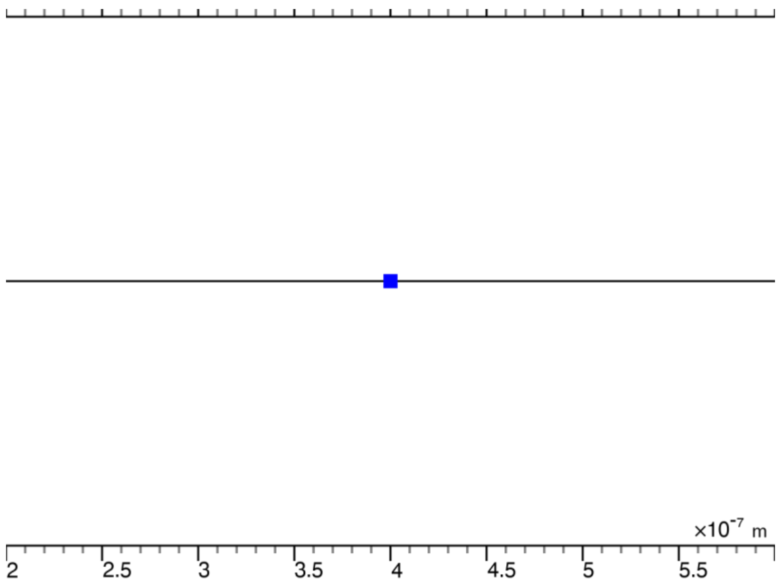

Surface Reactions H2O oxidation

SELECTION

Geometric entity level	Boundary
Selection	Geometry geom1: Dimension 0: Boundary 3

EQUATIONS

$$\frac{\partial c_{s,i}}{\partial t} = R_{s,i}$$

$$\theta_i = \frac{c_{s,i}\sigma_i}{\Gamma_s}$$

$$\frac{\partial c_{b,i}}{\partial t} = R_{b,i}$$

2.8.1 Interface Settings

Discretization

SETTINGS

Description	Value
Surface concentration	Linear
Bulk concentration	Linear

SETTINGS

Description	Value
Equation form	Study controlled

2.8.2 Surface Properties 1

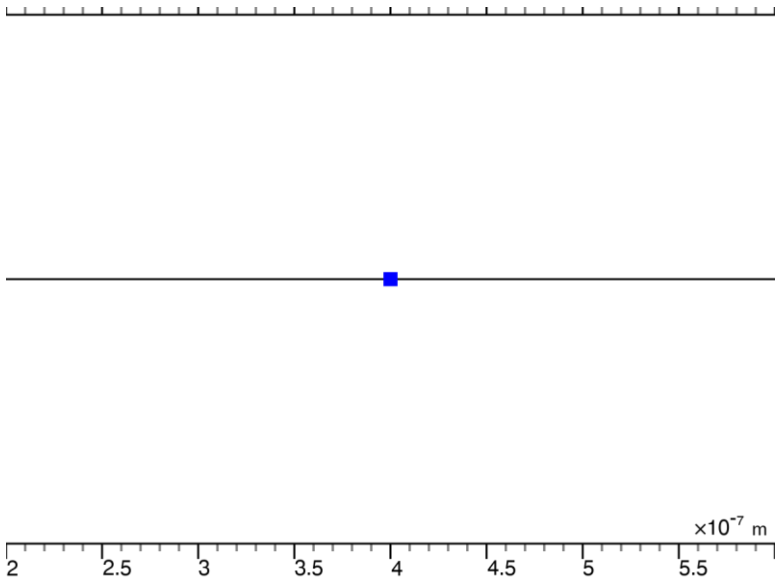

Surface Properties 1

SELECTION

Geometric entity level	Boundary
Selection	Geometry geom1: Dimension 0: All boundaries

EQUATIONS

$$\frac{\partial c_{s,j}}{\partial t} = R_{s,j}$$

$$\theta_i = \frac{c_{s,j}\sigma_i}{\Gamma_s}$$

Sites

SETTINGS

Description	Value
Density of sites	2e-5[mol/m^2]
Site occupancy number	1

Variables

Name	Expression	Unit	Description	Selection	Details
sr2.Rs_csSo	0	mol/(m²·s)	Reaction rate for surface species	Boundary 3	+ operation
sr2.Gamma	2.0E-5[mol/m^2]	mol/m²	Density of sites	Boundary 3	
sr2.Sigma_csSo	1	1	Site occupancy number	Boundary 3	
sr2.theta_i_csSo	max(eps^2,sr2.Sigma_csSo*csSo/sr2.Gamma)	1	Surface coverage	Boundary 3	
sr2.theta_i0	sr2.theta_i_csSo	1	Surface coverage	Boundary 3	+ operation
sr2.theta_free	max(eps^2,min(1,1-sr2.theta_i0))	1	Fraction of free sites	Boundary 3	

Shape functions

Name	Shape function	Unit	Description	Shape frame	Selection
csSo	Lagrange (Linear)	mol/m²	Surface concentration	Spatial	Boundary 3

Weak Expressions

Weak expression	Integration order	Integration frame	Selection
-d(csSo,t)*test(csSo)	2	Spatial	Boundary 3

2.8.3 Initial Values 1

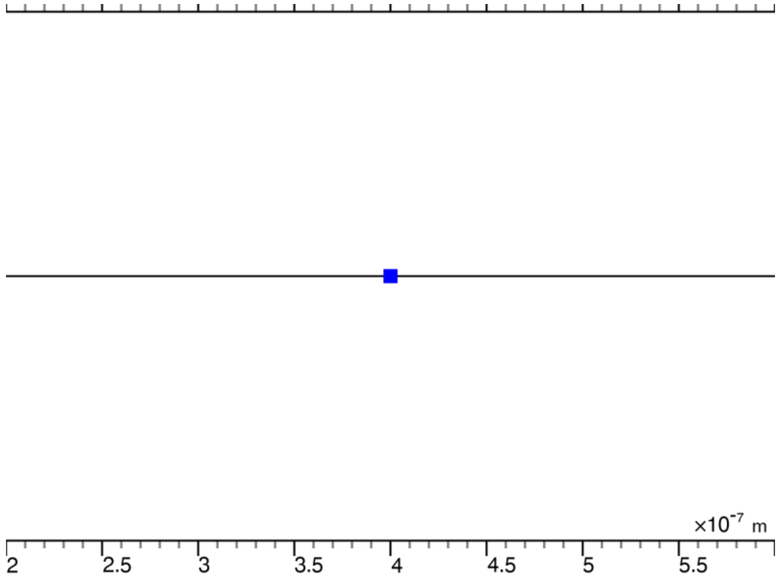

Initial Values 1

SELECTION

Geometric entity level	Boundary
Selection	Geometry geom1: Dimension 0: All boundaries

Initial Values

SETTINGS

Description	Value
Surface concentration	1

2.8.4 Reactions 1

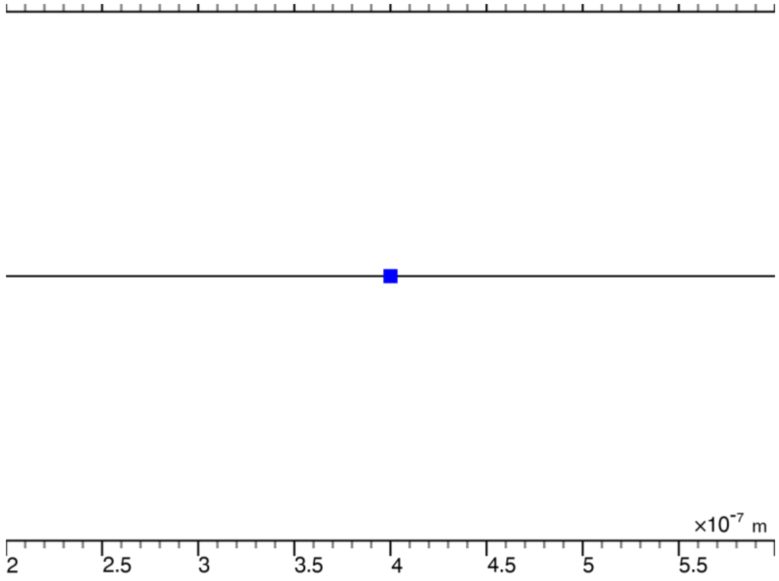

Reactions 1

SELECTION

Geometric entity level	Boundary
Selection	Geometry geom1: Dimension 0: Boundary 3

EQUATIONS

$$\frac{\partial c_{s,i}}{\partial t} = R_{s,i}$$

Reaction Rate for Surface Species

SETTINGS

Description	Value
Reaction rate for surface species	User defined
Reaction rate for surface species	0

Variables

Name	Expression	Unit	Description	Selection	Details
sr2.Rs_csSo	model.input.Rs_csSo	mol/(m²·s)	Reaction rate for surface species	Boundary 3	Meta, + operation

Weak Expressions

Weak expression	Integration order	Integration frame	Selection
model.input.Rs_csSo*test(csSo)	2	Spatial	Boundary 3

2.9 CHEMISTRY CO2 REDUCTION

USED PRODUCTS

COMSOL Multiphysics
Chemical Reaction Engineering Module

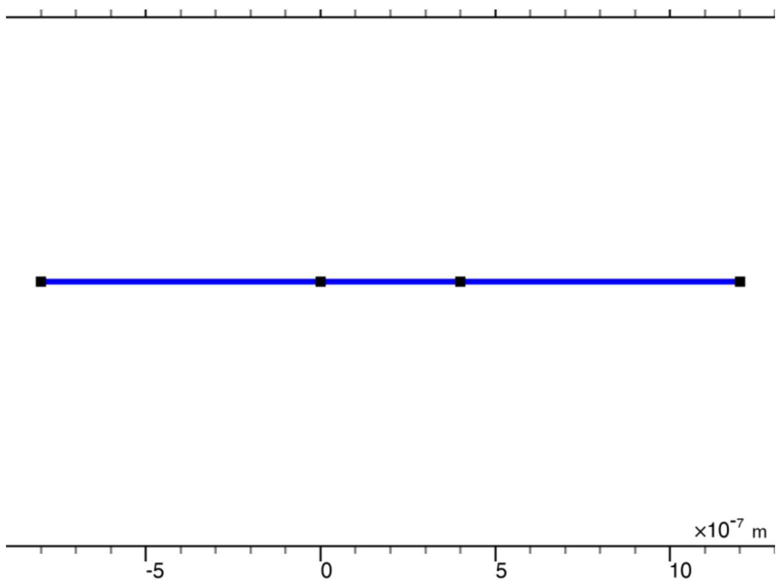

Chemistry CO2 reduction

SELECTION

Geometric entity level	Domain
Selection	Geometry geom1: Dimension 1: All domains

EQUATIONS

$$R_i = \sum_j R_{ij}, \quad R_{\mathrm{ads},i} = \sum_j R_{\mathrm{ads},ij}$$

2.9.1 Interface Settings

Discretization

SETTINGS

Description	Value
Element order	Quadratic

SETTINGS

Description	Value
Equation form	Study controlled

Model Input

SETTINGS

Description	Value
Temperature	Common model input
Pressure	Common model input

Mixture Properties

SETTINGS

Description	Value
Type	Diluted species
Phase	Liquid

Species Matching

SETTINGS

Description	Value
Species solved for	User defined

Species	Type	Molar concentration	Value	Reaction rate	Unit	Equation formulation
Asc						
Ascm						
CO						
CO2						
H2O						
H2PO4						
HPO4						

Species	Type	Molar concentration	Value	Reaction rate	Unit	Equation formulation
Variable						
Variable						
Variable						
Variable						
Solvent						
Variable						
Variable						

Species	Type	Molar concentration	Value	Reaction rate	Unit	Equation formulation

Species	Type	Molar concentration	Value	Reaction rate	Unit	Equation formulation
User defined						
User defined						
User defined						
User defined						
User defined						
User defined						
User defined						

Species	Type	Molar concentration	Value	Reaction rate	Unit	Equation formulation
cAsc						
cAscm						
cCO						
cCO2						
cH2O						
cH2PO4						
cHPO4						

Species	Type	Molar concentration	Value	Reaction rate	Unit	Equation formulation
chem.R_Asc						
chem.R_Ascm						
chem.R_CO						
chem.R_CO2						
solvent						
chem.R_H2PO4						
chem.R_HPO4						

Species	Type	Molar concentration	Value	Reaction rate	Unit	Equation formulation
mol/m^3						
mol/m^3						
mol/m^3						
mol/m^3						
mol/m^3						
mol/m^3						
mol/m^3						

Species	Type	Molar concentration	Value	Reaction rate	Unit	Equation formulation
cAsc						
cAscm						
cCO						
cCO2						
cH2O						
cH2PO4						
cHPO4						

Species	Species concentration type	Surface concentration
CO2(ads)	Variable	csCO2
Sr(ads)	Variable	csSr

Calculate Transport Properties

SETTINGS

Description	Value
Calculate mixture properties	Off

Activity

SETTINGS

Description	Value
Use activity	Off

CHEMKIN Import for Species Properties

SETTINGS

Description	Value
Thermo input file	
Transport input file	

Pellet Chemistry

SETTINGS

Description	Value
Define variables for porous pellets	Off

2.9.2 Variables

Name	Expression	Unit	Description	Selection	Details
chem.T	model.input.T	K	Temperature	Domains 1–3	Meta
chem.p	model.input.p	Pa	Pressure	Domains 1–3	Meta
chem.eeqMean	chem.eeqSum/chem.nEr	V	Mean equilibrium potential	Global	
chem.csum	eps+chem.c_Asc+chem.c_Ascm+chem.c_CO+chem.c_CO2+chem.c_H2O+chem.c_H2PO4+chem.c_HPO4	mol/m³	Total concentration	Global	
chem.m_Asc	max(chem.c_Asc,0)/chem.csum	1	Molar fraction	Global	
chem.m_Ascm	max(chem.c_Ascm,0)/chem.csum	1	Molar fraction	Global	
chem.m_CO	max(chem.c_CO,0)/chem.csum	1	Molar fraction	Global	
chem.m_CO2	max(chem.c_CO2,0)/chem.csum	1	Molar fraction	Global	
chem.m_H2O	max(chem.c_H2O,0)/chem.csum	1	Molar fraction	Global	
chem.m_H2PO4	max(chem.c_H2PO4,0)/chem.csum	1	Molar fraction	Global	
chem.m_HPO4	max(chem.c_HPO4,0)/chem.csum	1	Molar fraction	Global	
chem.rhosum	eps+chem.c_Asc*chem.M_Asc+chem.c_Ascm*chem.M_Ascm+chem.c_CO*chem.M_CO+chem.c_CO2*chem.M_CO2+chem.c_H2O*chem.M_H2O+chem.c_H2PO4*chem.M_H2PO4+chem.c_HPO4*chem.M_HPO4	kg/m³	Density	Global	
chem.w_Asc	chem.c_Asc*chem.M_Asc/chem.rhosum	1	Mass fraction	Global	

Name	Expression	Unit	Description	Selection	Details
chem.w_Ascm	chem.c_Ascm*chem.M_Ascm/chem.rhosum	1	Mass fraction	Global	
chem.w_CO	chem.c_CO*chem.M_CO/chem.rhosum	1	Mass fraction	Global	
chem.w_CO2	chem.c_CO2*chem.M_CO2/chem.rhosum	1	Mass fraction	Global	
chem.w_H2O	chem.c_H2O*chem.M_H2O/chem.rhosum	1	Mass fraction	Global	
chem.w_H2PO4	chem.c_H2PO4*chem.M_H2PO4/chem.rhosum	1	Mass fraction	Global	
chem.w_HPO4	chem.c_HPO4*chem.M_HPO4/chem.rhosum	1	Mass fraction	Global	
chem.c_Asc	cAsc	mol/m³	Concentration	Global	
chem.Rsum_Asc	0	mol/(m³·s)	Reaction rate for species Asc	Global	+ operation
chem.R_Asc	0	mol/(m³·s)	Reaction rate for species Asc	Global	+ operation
chem.Rsurf_Asc	0	mol/(m²·s)	Surface reaction rate for species Asc	Global	+ operation
chem.Rsurfsum_Asc	0	mol/(m²·s)	Surface reaction rate for species Asc	Global	+ operation
chem.c_Ascm	cAscm	mol/m³	Concentration	Global	
chem.Rsum_Ascm	0	mol/(m³·s)	Reaction rate for species Ascm	Global	+ operation
chem.R_Ascm	0	mol/(m³·s)	Reaction rate for species Ascm	Global	+ operation
chem.Rsurf_Ascm	0	mol/(m²·s)	Surface reaction rate for species Ascm	Global	+ operation
chem.Rsurfsum_Ascm	0	mol/(m²·s)	Surface reaction rate for species Ascm	Global	+ operation
chem.c_CO	cCO	mol/m³	Concentration	Global	
chem.Rsum_CO	0	mol/(m³·s)	Reaction rate for species CO	Global	+ operation
chem.R_CO	0	mol/(m³·s)	Reaction rate for species CO	Global	+ operation
chem.Rsurf_CO	0	mol/(m²·s)	Surface reaction	Global	+ operation

Name	Expression	Unit	Description	Selection	Details
			rate for species CO		
chem.Rsurfsum_CO	0	mol/(m²·s)	Surface reaction rate for species CO	Global	+ operation
chem.c_CO2	cCO2	mol/m³	Concentration	Global	
chem.Rsum_CO2	0	mol/(m³·s)	Reaction rate for species CO2	Global	+ operation
chem.R_CO2	0	mol/(m³·s)	Reaction rate for species CO2	Global	+ operation
chem.Rsurf_CO2	0	mol/(m²·s)	Surface reaction rate for species CO2	Global	+ operation
chem.Rsurfsum_CO2	0	mol/(m²·s)	Surface reaction rate for species CO2	Global	+ operation
chem.c_H2O	cH2O	mol/m³	Concentration	Global	
chem.Rsum_H2O	0	mol/(m³·s)	Reaction rate for species H2O	Global	+ operation
chem.c_H2PO4	cH2PO4	mol/m³	Concentration	Global	
chem.Rsum_H2PO4	0	mol/(m³·s)	Reaction rate for species H2PO4	Global	+ operation
chem.R_H2PO4	0	mol/(m³·s)	Reaction rate for species H2PO4	Global	+ operation
chem.Rsurf_H2PO4	0	mol/(m²·s)	Surface reaction rate for species H2PO4	Global	+ operation
chem.Rsurfsum_H2PO4	0	mol/(m²·s)	Surface reaction rate for species H2PO4	Global	+ operation
chem.c_HPO4	cHPO4	mol/m³	Concentration	Global	
chem.Rsum_HPO4	0	mol/(m³·s)	Reaction rate for species HPO4	Global	+ operation
chem.R_HPO4	0	mol/(m³·s)	Reaction rate for species HPO4	Global	+ operation
chem.Rsurf_HPO4	0	mol/(m²·s)	Surface reaction rate for species HPO4	Global	+ operation
chem.Rsurfsum_HPO4	0	mol/(m²·s)	Surface reaction rate for species HPO4	Global	+ operation
chem.csurf_CO2_surf	csCO2	mol/m²	Surface concentration	Global	

Name	Expression	Unit	Description	Selection	Details
chem.Rsum_CO2_surf	0	mol/(m²·s)	Surface reaction rate for surface species CO2_surf	Global	+ operation
chem.R_CO2_surf	0	mol/(m²·s)	Surface reaction rate for surface species CO2_surf	Global	+ operation
chem.csurf_Sr_surf	csSr	mol/m²	Surface concentration	Global	
chem.Rsum_Sr_surf	0	mol/(m²·s)	Surface reaction rate for surface species Sr_surf	Global	+ operation
chem.R_Sr_surf	0	mol/(m²·s)	Surface reaction rate for surface species Sr_surf	Global	+ operation
chem.Qheat	0		Heat source of reactions	Global	+ operation
chem.Qtot	chem.Qheat	W/m³	Heat source of reactions	Global	
chem.Qs	0	W/m²	Surface heat source of reaction	Global	+ operation
chem.Qb	chem.Qs	W/m²	Surface heat source of reaction	Global	
chem.Mn	chem.M_H2O	kg/mol	Mean molar mass	Global	

2.9.3 1: Surface: CO2<=>CO2(ads)

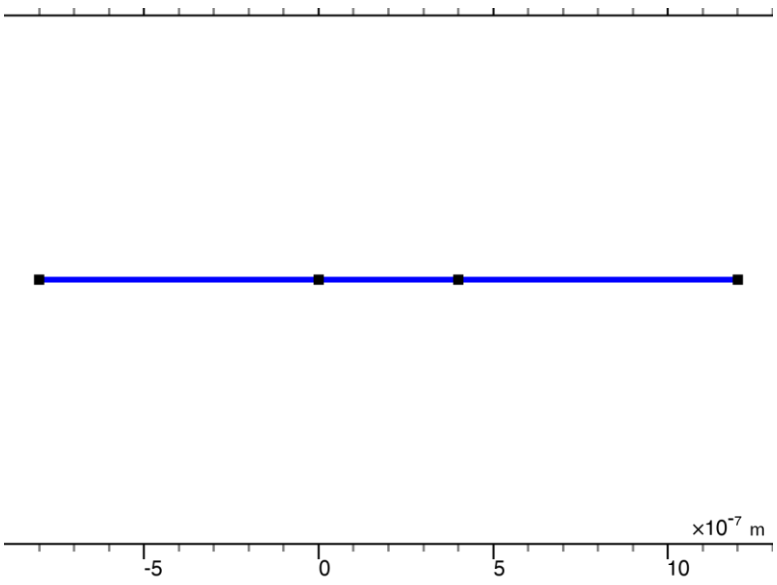

1: Surface: CO2<=>CO2(ads)

SELECTION

Geometric entity level	Domain
Selection	Geometry geom1: Dimension 1: All domains

Reaction Formula

SETTINGS

Description	Value
Formula	CO2<=>CO2(ads)
Reaction type	Reversible

Reaction Rate

SETTINGS

Description	Value
Reaction rate	Mass action law
Description	Overall forward reaction order: 1 (volumetric), 0 (surface) Overall reverse reaction order: 0 (volumetric), 1 (surface)

Rate Constants

SETTINGS

Description	Value
Specify equilibrium constant	Off
Use Arrhenius expressions	Off
Forward rate constant	k_adsCO2
Reverse rate constant	k_desCO2

Reaction Thermodynamic Properties

SETTINGS

Description	Value
Enthalpy of reaction	Automatic
Entropy of reaction	Automatic
Heat source of reaction	Automatic

Variables

Name	Expression	Unit	Description	Selection	Details
chem.Rsurf_CO2	-chem.r_1	mol/(m²·s)	Surface reaction rate for species CO2	Global	+ operation
chem.Rsurfsum_CO2	-chem.r_1	mol/(m²·s)	Surface reaction rate for species	Global	+ operation

Name	Expression	Unit	Description	Selection	Details
			CO2		
chem.Rsum_CO2_surf	chem.r_1	mol/(m²·s)	Surface reaction rate for surface species CO2_surf	Global	+ operation
chem.R_CO2_surf	chem.r_1	mol/(m²·s)	Surface reaction rate for surface species CO2_surf	Global	+ operation
chem.Qs	-chem.r_1*chem.H_1	W/m²	Surface heat source of reaction	Global	+ operation
chem.kf_1	k_adsCO2	m/s	Forward rate constant	Global	
chem.kr_1	k_desCO2	1/s	Reverse rate constant	Global	
chem.r_1	chem.kf_1*chem.c_CO2*(Gamma_sCO2-chem.csurf_CO2_surf) - chem.kr_1*chem.csurf_CO2_surf	mol/(m²·s)	Reaction rate	Global	
chem.H_1	-chem.h_CO2+chem.h_CO2_surf	J/mol	Enthalpy of reaction	Global	
chem.S_1	-chem.s_CO2+chem.s_CO2_surf	J/(mol·K)	Entropy of reaction	Global	
chem.Qs_1	-chem.r_1*chem.H_1	W/m²	Surface heat source of reaction	Global	

2.9.4 Species: CO2

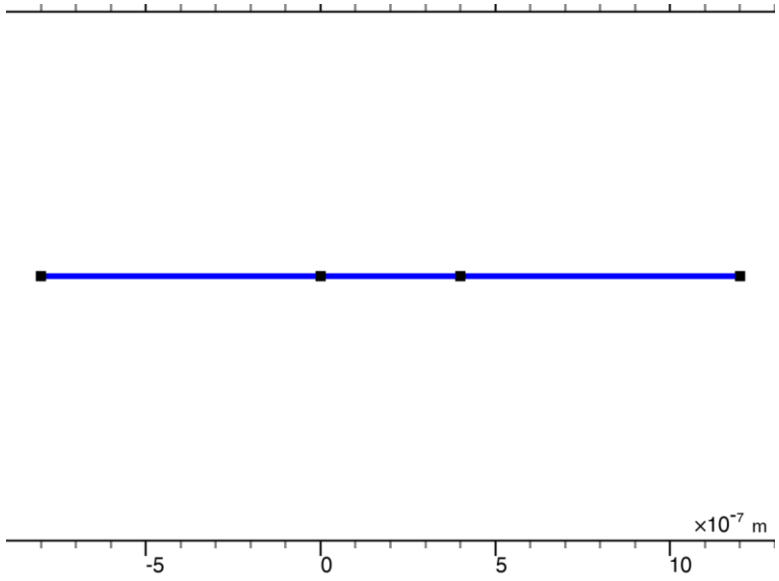

Species: CO2

SELECTION

Geometric entity level	Domain
Selection	Geometry geom1: Dimension 1: All domains

SETTINGS

Description	Value
Species name	CO2

Species Type

SETTINGS

Description	Value
Species type	Bulk species

General Parameters

SETTINGS

Description	Value
Molar mass	44.0094[g/mol]
Charge	0

Reaction Rate

SETTINGS

Description	Value

Description	Value
	Automatic

Species Concentration/Activity

SETTINGS

Description	Value
Constant concentration/activity	Off

Additional Source

SETTINGS

Description	Value
Additional source	Off

Species Thermodynamic Expressions

SETTINGS

Description	Value
Species enthalpy	NASA format
Lower temperature limit	300[K]
Middle temperature limit	1000[K]
Upper temperature limit	5000[K]
	0
	0
	0
	0
	0
	0
	0
	0
	0
	0
	0
	0
	0
	0

Variables

Name	Expression	Unit	Description	Selection
chem.M_CO2	44.0094[g/mol]	kg/mol	Molar mass	Global

Name	Expression	Unit	Description	Selection
chem.z_CO2	0	1	Charge	Global
chem.Tlo_CO2	300[K]	K	Lower temperature limit	Global
chem.Tmid_CO2	1000[K]	K	Middle temperature limit	Global
chem.Thi_CO2	5000[K]	K	Upper temperature limit	Global
chem.aLo1_CO2	0	1	Low polynomial coefficients	Global
chem.aHi1_CO2	0	1	High polynomial coefficients	Global
chem.aLo2_CO2	0	1/K	Low polynomial coefficients	Global
chem.aHi2_CO2	0	1/K	High polynomial coefficients	Global
chem.aLo3_CO2	0	$1/K^2$	Low polynomial coefficients	Global
chem.aHi3_CO2	0	$1/K^2$	High polynomial coefficients	Global
chem.aLo4_CO2	0	$1/K^3$	Low polynomial coefficients	Global
chem.aHi4_CO2	0	$1/K^3$	High polynomial coefficients	Global
chem.aLo5_CO2	0	$1/K^4$	Low polynomial coefficients	Global
chem.aHi5_CO2	0	$1/K^4$	High polynomial coefficients	Global
chem.aLo6_CO2	0	K	Low polynomial coefficients	Global
chem.aHi6_CO2	0	K	High polynomial coefficients	Global
chem.aLo7_CO2	0	1	Low polynomial coefficients	Global
chem.aHi7_CO2	0	1	High polynomial coefficients	Global
chem.Cp_CO2	R_const*((chem.T<=chem.Tlo_CO2)*(chem.aLo1_CO2+chem.Tlo_CO2*(chem.aLo2_CO2+chem.Tlo_CO2*(chem.aLo3_CO2+chem.Tlo_CO2*(chem.aLo4_CO2+chem.Tlo_CO2*chem.aLo5_CO2))))+(chem.T>chem.Tlo_C	J/(mol·K)	Heat capacity at constant pressure	Global

Name	Expression	Unit	Description	Selection
	O2)*(chem.T<=chem.Tmid_CO2)*(chem.aLo1_CO2+chem.T*(chem.aLo2_CO2+chem.T*(chem.aLo3_CO2+chem.T*(chem.aLo4_CO2+chem.T*chem.aLo5_CO2))))+(chem.T>chem.Tmid_CO2)*(chem.T<=chem.Thi_CO2)*(chem.aHi1_CO2+chem.T*(chem.aHi2_CO2+chem.T*(chem.aHi3_CO2+chem.T*(chem.aHi4_CO2+chem.T*chem.aHi5_CO2))))+(chem.T>chem.Thi_CO2)*(chem.aHi1_CO2+chem.Thi_CO2*(chem.aHi2_CO2+chem.Thi_CO2*(chem.aHi3_CO2+chem.Thi_CO2*(chem.aHi4_CO2+chem.Thi_CO2*chem.aHi5_CO2)))))			
chem.h_CO2	R_const*((chem.T<=chem.Tlo_CO2)*(chem.aLo6_CO2+chem.Tlo_CO2*(chem.aLo1_CO2+0.5*chem.Tlo_CO2*(chem.aLo2_CO2+2*chem.Tlo_CO2*(chem.aLo3_CO2+0.75*chem.Tlo_CO2*(chem.aLo4_CO2+0.8*chem.Tlo_CO2*chem.aLo5_CO2))/3)))+(chem.T>chem.Tlo_CO2)*(chem.T<=chem.Tmid_CO2)*(chem.aLo6_CO2+chem.T*(chem.aLo1_CO2+0.5*chem.T*(chem.aLo2_CO2+2*chem.T*(chem.aLo3_CO2+0.75*chem.T*(chem.aLo4_CO2+0.8*chem.T*chem.aLo5_CO2))/3)))+(chem.T>chem.Tmid_CO2)*(chem.T<=chem.Thi_CO2)*(chem.aHi6_CO2+chem.T*(chem.aHi1_CO2+0.5*chem.T*(chem.aHi2_CO2+2*chem.T*(chem.aHi3_CO2+0.75*chem.T*(chem.aHi4_CO2+0.8*chem.T*chem.aHi5_CO2))/3)))+(chem.T>chem.Thi_CO2)*(chem.aHi6_CO2+chem.Thi_CO2*(chem.aHi1_CO2+0.5*chem.Thi_CO2*(chem.aHi2_CO2+2*chem.Thi_CO2*(chem.aHi3_CO2+0.75*chem.Thi_CO2*(chem.aHi4_CO2+0.8*chem.Thi_CO2*chem.aHi5_CO2))/3))))	J/mol	Molar enthalpy	Global
chem.s_CO2	R_const*((chem.T<=chem.Tlo_CO2)*(chem.aLo7_CO2+chem.aLo1_CO2*log(chem.T/1[K])+chem.Tlo_CO2*(chem.aLo2_CO2+0.5*chem.Tlo_CO2*(chem.aLo3_CO2+2*chem.Tlo_CO2*(chem.aLo4_CO2+0.75*chem.Tlo_C	J/(mol·K)	Molar entropy	Global

Name	Expression	Unit	Description	Selection
	O2*chem.aLo5_CO2)/3)))+(chem.T>chem.Tlo_CO2)*(chem.T<=chem.Tmid_CO2)*(chem.aLo7_CO2+chem.aLo1_CO2*log(chem.T/1[K])+chem.T*(chem.aLo2_CO2+0.5*chem.T*(chem.aLo3_CO2+2*chem.T*(chem.aLo4_CO2+0.75*chem.T*chem.aLo5_CO2)/3)))+(chem.T>chem.Tmid_CO2)*(chem.T<=chem.Thi_CO2)*(chem.aHi7_CO2+chem.aHi1_CO2*log(chem.T/1[K])+chem.T*(chem.aHi2_CO2+0.5*chem.T*(chem.aHi3_CO2+2*chem.T*(chem.aHi4_CO2+0.75*chem.T*chem.aHi5_CO2)/3)))+(chem.T>chem.Thi_CO2)*(chem.aHi7_CO2+chem.aHi1_CO2*log(chem.T/1[K])+chem.Thi_CO2*(chem.aHi2_CO2+0.5*chem.Thi_CO2*(chem.aHi3_CO2+2*chem.Thi_CO2*(chem.aHi4_CO2+0.75*chem.Thi_CO2*chem.aHi5_CO2)/3))))			

2.9.5 Surface species: CO2(ads)

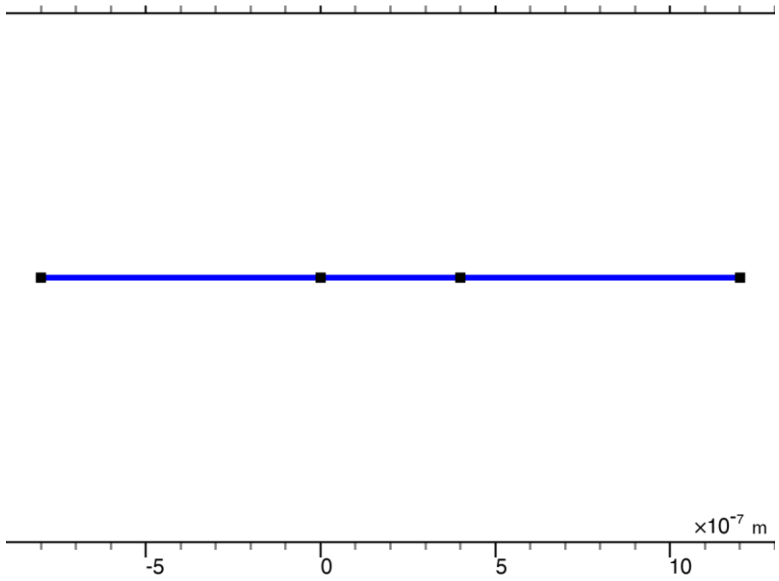

Surface species: CO2(ads)

SELECTION

Geometric entity level	Domain
Selection	Geometry geom1: Dimension 1: All domains

SETTINGS

Description	Value
Species name	CO2_surf

Species Type

SETTINGS

Description	Value
Species type	Surface species

General Parameters

SETTINGS

Description	Value
Molar mass	44.0094[g/mol]
Charge	0

Reaction Rate

SETTINGS

Description	Value
	Automatic

Species Concentration/Activity

SETTINGS

Description	Value
Constant concentration/activity	Off

Additional Source

SETTINGS

Description	Value
Additional source	Off

Species Thermodynamic Expressions

SETTINGS

Description	Value
Species enthalpy	NASA format
Lower temperature limit	300[K]
Middle temperature limit	1000[K]
Upper temperature limit	5000[K]
	0
	0

Description	Value
	0
	0
	0
	0
	0
	0
	0
	0
	0
	0
	0
	0

Variables

Name	Expression	Unit	Description	Selection
chem.M_CO2_surf	44.0094[g/mol]	kg/mol	Molar mass	Global
chem.z_CO2_surf	0	1	Charge	Global
chem.Tlo_CO2_surf	300[K]	K	Lower temperature limit	Global
chem.Tmid_CO2_surf	1000[K]	K	Middle temperature limit	Global
chem.Thi_CO2_surf	5000[K]	K	Upper temperature limit	Global
chem.aLo1_CO2_surf	0	1	Low polynomial coefficients	Global
chem.aHi1_CO2_surf	0	1	High polynomial coefficients	Global
chem.aLo2_CO2_surf	0	1/K	Low polynomial coefficients	Global
chem.aHi2_CO2_surf	0	1/K	High polynomial coefficients	Global
chem.aLo3_CO2_surf	0	$1/K^2$	Low polynomial coefficients	Global
chem.aHi3_CO2_surf	0	$1/K^2$	High polynomial coefficients	Global
chem.aLo4_CO2_surf	0	$1/K^3$	Low polynomial coefficients	Global

Name	Expression	Unit	Description	Selection
chem.aHi4_CO2_surf	0	$1/K^3$	High polynomial coefficients	Global
chem.aLo5_CO2_surf	0	$1/K^4$	Low polynomial coefficients	Global
chem.aHi5_CO2_surf	0	$1/K^4$	High polynomial coefficients	Global
chem.aLo6_CO2_surf	0	K	Low polynomial coefficients	Global
chem.aHi6_CO2_surf	0	K	High polynomial coefficients	Global
chem.aLo7_CO2_surf	0	1	Low polynomial coefficients	Global
chem.aHi7_CO2_surf	0	1	High polynomial coefficients	Global
chem.Cp_CO2_surf	R_const*((chem.T<=chem.Tlo_CO2_surf)*(chem.aLo1_CO2_surf+chem.Tlo_CO2_surf*(chem.aLo2_CO2_surf+chem.Tlo_CO2_surf*(chem.aLo3_CO2_surf+chem.Tlo_CO2_surf*(chem.aLo4_CO2_surf+chem.Tlo_CO2_surf*chem.aLo5_CO2_surf))))+(chem.T>chem.Tlo_CO2_surf)*(chem.T<=chem.Tmid_CO2_surf)*(chem.aLo1_CO2_surf+chem.T*(chem.aLo2_CO2_surf+chem.T*(chem.aLo3_CO2_surf+chem.T*(chem.aLo4_CO2_surf+chem.T*chem.aLo5_CO2_surf))))+(chem.T>chem.Tmid_CO2_surf)*(chem.T<=chem.Thi_CO2_surf)*(chem.aHi1_CO2_surf+chem.T*(chem.aHi2_CO2_surf+chem.T*(chem.aHi3_CO2_surf+chem.T*(chem.aHi4_CO2_surf+chem.T*chem.aHi5_CO2_surf))))+(chem.T>chem.Thi_CO2_surf)*(chem.aHi1_CO2_surf+chem.Thi_CO2_surf*(chem.aHi2_CO2_surf+chem.Thi_CO2_surf*(chem.aHi3_CO2_surf+chem.Thi_CO2_surf*(chem.aHi4_CO2_surf+chem.Thi_CO2_surf*chem.aHi5_CO2_surf)))))	J/(mol·K)	Heat capacity at constant pressure	Global
chem.h_CO2_surf	R_const*((chem.T<=chem.Tlo_CO2_surf)*(chem.aLo6_CO2_surf+chem.Tlo_CO2_surf*(chem.aLo1_CO2_surf+0.5*chem.Tlo_CO2_surf*(chem.aLo2_CO2_surf+2*chem.Tlo_CO2_surf*(chem.aLo3_CO2_surf+0.75*chem.Tlo_CO2_surf*(chem.aLo4_CO2_surf+0.8*chem.Tlo_CO2_surf*chem.aLo5_CO2_surf))/3)))+(chem.T>chem.Tlo_CO2_surf)*(chem.T<=chem.Tmid_CO2_surf)*(chem.	J/mol	Molar enthalpy	Global

Name	Expression	Unit	Description	Selection
	aLo6_CO2_surf+chem.T*(chem.aLo1_CO2_surf+0.5*chem.T*(chem.aLo2_CO2_surf+2*chem.T*(chem.aLo3_CO2_surf+0.75*chem.T*(chem.aLo4_CO2_surf+0.8*chem.T*chem.aLo5_CO2_surf))/3)))+(chem.T>chem.Tmid_CO2_surf)*(chem.T<=chem.Thi_CO2_surf)*(chem.aHi6_CO2_surf+chem.T*(chem.aHi1_CO2_surf+0.5*chem.T*(chem.aHi2_CO2_surf+2*chem.T*(chem.aHi3_CO2_surf+0.75*chem.T*(chem.aHi4_CO2_surf+0.8*chem.T*chem.aHi5_CO2_surf))/3)))+(chem.T>chem.Thi_CO2_surf)*(chem.aHi6_CO2_surf+chem.Thi_CO2_surf*(chem.aHi1_CO2_surf+0.5*chem.Thi_CO2_surf*(chem.aHi2_CO2_surf+2*chem.Thi_CO2_surf*(chem.aHi3_CO2_surf+0.75*chem.Thi_CO2_surf*(chem.aHi4_CO2_surf+0.8*chem.Thi_CO2_surf*chem.aHi5_CO2_surf))/3))))			
chem.s_CO2_surf	R_const*((chem.T<=chem.Tlo_CO2_surf)*(chem.aLo7_CO2_surf+chem.aLo1_CO2_surf*log(chem.T/1[K])+chem.Tlo_CO2_surf*(chem.aLo2_CO2_surf+0.5*chem.Tlo_CO2_surf*(chem.aLo3_CO2_surf+2*chem.Tlo_CO2_surf*(chem.aLo4_CO2_surf+0.75*chem.Tlo_CO2_surf*chem.aLo5_CO2_surf)/3)))+(chem.T>chem.Tlo_CO2_surf)*(chem.T<=chem.Tmid_CO2_surf)*(chem.aLo7_CO2_surf+chem.aLo1_CO2_surf*log(chem.T/1[K])+chem.T*(chem.aLo2_CO2_surf+0.5*chem.T*(chem.aLo3_CO2_surf+2*chem.T*(chem.aLo4_CO2_surf+0.75*chem.T*chem.aLo5_CO2_surf)/3)))+(chem.T>chem.Tmid_CO2_surf)*(chem.T<=chem.Thi_CO2_surf)*(chem.aHi7_CO2_surf+chem.aHi1_CO2_surf*log(chem.T/1[K])+chem.T*(chem.aHi2_CO2_surf+0.5*chem.T*(chem.aHi3_CO2_surf+2*chem.T*(chem.aHi4_CO2_surf+0.75*chem.T*chem.aHi5_CO2_surf)/3)))+(chem.T>chem.Thi_CO2_surf)*(chem.aHi7_CO2_surf+chem.aHi1_CO2_surf*log(chem.T/1[K])+chem.Thi_CO2_surf*(chem.aHi2_CO2_surf+0.5*chem.Thi_CO2_surf*(chem.aHi3_CO2_surf+2*chem.Thi_CO2_surf*(chem.aHi4_CO2_surf+0.75*chem.Thi_CO2_surf*chem.aHi5_CO2_surf)/3))))	J/(mol·K)	Molar entropy	Global

2.9.6 2: Surface: CO2(ads)+2Ascm+2H2PO4+Sr(ads)=>2Asc+CO+H2O+2HPO4+Sr(ads)

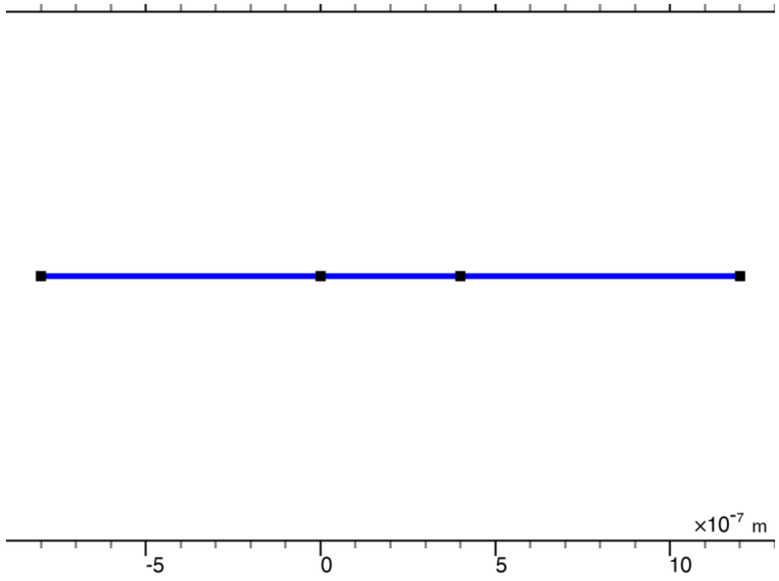

2: Surface: CO2(ads)+2Ascm+2H2PO4+Sr(ads)=>2Asc+CO+H2O+2HPO4+Sr(ads)

SELECTION

Geometric entity level	Domain
Selection	Geometry geom1: Dimension 1: All domains

Reaction Formula

SETTINGS

**Description**	**Value**
Formula	CO2(ads) + 2Ascm + 2H2PO4 + Sr(ads)=>2Asc + CO + H2O + 2HPO4 + Sr(ads)
Reaction type	Irreversible

Reaction Rate

SETTINGS

**Description**	**Value**
Reaction rate	Mass action law
Description	Overall forward reaction order: 4 (volumetric), 2 (surface)

Rate Constants

SETTINGS

**Description**	**Value**
Use Arrhenius expressions	Off
Forward rate constant	kf_CO2*step1(t)

Reaction Thermodynamic Properties

SETTINGS

Description	Value
Enthalpy of reaction	Automatic
Entropy of reaction	Automatic
Heat source of reaction	Automatic

Variables

Name	Expression	Unit	Description	Selection	Details
chem.Rsurf_Asc	2*chem.r_2	mol/(m²·s)	Surface reaction rate for species Asc	Global	+ operation
chem.Rsurfsum_Asc	2*chem.r_2	mol/(m²·s)	Surface reaction rate for species Asc	Global	+ operation
chem.Rsurf_Ascm	-2*chem.r_2	mol/(m²·s)	Surface reaction rate for species Ascm	Global	+ operation
chem.Rsurfsum_Ascm	-2*chem.r_2	mol/(m²·s)	Surface reaction rate for species Ascm	Global	+ operation
chem.Rsurf_CO	chem.r_2	mol/(m²·s)	Surface reaction rate for species CO	Global	+ operation
chem.Rsurfsum_CO	chem.r_2	mol/(m²·s)	Surface reaction rate for species CO	Global	+ operation
chem.Rsurf_H2PO4	-2*chem.r_2	mol/(m²·s)	Surface reaction rate for species H2PO4	Global	+ operation
chem.Rsurfsum_H2PO4	-2*chem.r_2	mol/(m²·s)	Surface reaction rate for species H2PO4	Global	+ operation
chem.Rsurf_HPO4	2*chem.r_2	mol/(m²·s)	Surface reaction rate for species HPO4	Global	+ operation
chem.Rsurfsum_HPO4	2*chem.r_2	mol/(m²·s)	Surface reaction rate for species HPO4	Global	+ operation
chem.Rsum_CO2_surf	-chem.r_2	mol/(m²·s)	Surface reaction rate for surface species CO2_surf	Global	+ operation

Name	Expression	Unit	Description	Selection	Details
chem.R_CO2_surf	-chem.r_2	mol/(m²·s)	Surface reaction rate for surface species CO2_surf	Global	+ operation
chem.Rsum_Sr_surf	0	mol/(m²·s)	Surface reaction rate for surface species Sr_surf	Global	+ operation
chem.R_Sr_surf	0	mol/(m²·s)	Surface reaction rate for surface species Sr_surf	Global	+ operation
chem.Qs	-chem.r_2*chem.H_2	W/m²	Surface heat source of reaction	Global	+ operation
chem.Rsurfsum_H2O	0	mol/(m²·s)	Surface reaction rate for species H2O	Global	+ operation
chem.Rsurf_H2O	0	mol/(m²·s)	Surface reaction rate for species H2O	Global	+ operation
chem.kf_2	kf_CO2*step1(t)	$m^{14}/(s\cdot mol^5)$	Forward rate constant	Global	
chem.r_2	chem.kf_2*chem.csurf_CO2_surf*chem.csurf_Sr_surf*chem.c_Ascm*chem.c_H2PO4	mol/(m²·s)	Reaction rate	Global	
chem.H_2	-chem.h_CO2_surf-2*chem.h_Ascm-2*chem.h_H2PO4+2*chem.h_Asc+chem.h_CO+chem.h_H2O+2*chem.h_HPO4	J/mol	Enthalpy of reaction	Global	
chem.S_2	-chem.s_CO2_surf-2*chem.s_Ascm-2*chem.s_H2PO4+2*chem.s_Asc+chem.s_CO+chem.s_H2O+2*chem.s_HPO4	J/(mol·K)	Entropy of reaction	Global	
chem.Qs_2	-chem.r_2*chem.H_2	W/m²	Surface heat source of reaction	Global	

2.9.7 Species: Ascm

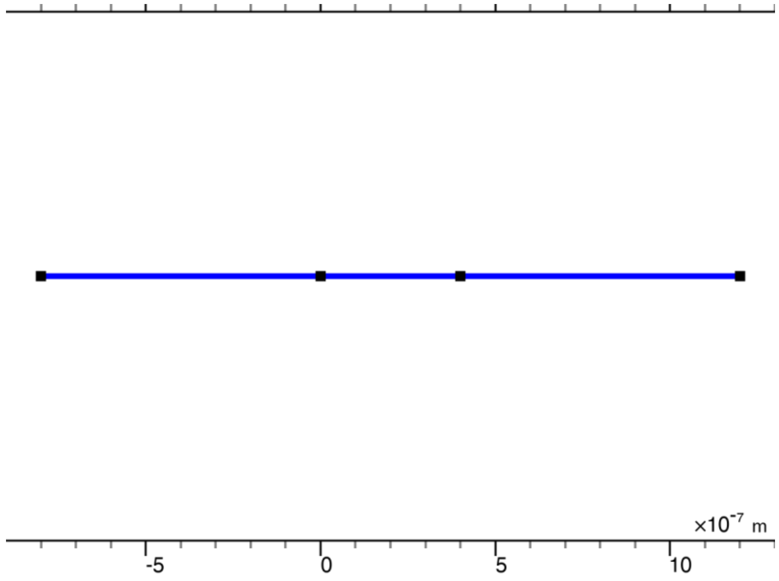

Species: Ascm

SELECTION

Geometric entity level	Domain
Selection	Geometry geom1: Dimension 1: All domains

SETTINGS

Description	Value
Species name	Ascm

Species Type

SETTINGS

Description	Value
Species type	Bulk species

General Parameters

SETTINGS

Description	Value
Molar mass	PM_Ascm
Charge	Z_Ascm

Reaction Rate

SETTINGS

Description	Value

Description	Value
	Automatic

Species Concentration/Activity

SETTINGS

Description	Value
Constant concentration/activity	Off

Additional Source

SETTINGS

Description	Value
Additional source	Off

Species Thermodynamic Expressions

SETTINGS

Description	Value
Species enthalpy	NASA format
Lower temperature limit	300[K]
Middle temperature limit	1000[K]
Upper temperature limit	5000[K]
	0
	0
	0
	0
	0
	0
	0
	0
	0
	0
	0
	0
	0
	0

Variables

Name	Expression	Unit	Description	Selection
chem.M_Asc	PM_Ascm	kg/mol	Molar mass	Global

Name	Expression	Unit	Description	Selection
m				
chem.z_Ascm	Z_Ascm	1	Charge	Global
chem.Tlo_Ascm	300[K]	K	Lower temperature limit	Global
chem.Tmid_Ascm	1000[K]	K	Middle temperature limit	Global
chem.Thi_Ascm	5000[K]	K	Upper temperature limit	Global
chem.aLo1_Ascm	0	1	Low polynomial coefficients	Global
chem.aHi1_Ascm	0	1	High polynomial coefficients	Global
chem.aLo2_Ascm	0	1/K	Low polynomial coefficients	Global
chem.aHi2_Ascm	0	1/K	High polynomial coefficients	Global
chem.aLo3_Ascm	0	$1/K^2$	Low polynomial coefficients	Global
chem.aHi3_Ascm	0	$1/K^2$	High polynomial coefficients	Global
chem.aLo4_Ascm	0	$1/K^3$	Low polynomial coefficients	Global
chem.aHi4_Ascm	0	$1/K^3$	High polynomial coefficients	Global
chem.aLo5_Ascm	0	$1/K^4$	Low polynomial coefficients	Global
chem.aHi5_Ascm	0	$1/K^4$	High polynomial coefficients	Global
chem.aLo6_Ascm	0	K	Low polynomial coefficients	Global
chem.aHi6_Ascm	0	K	High polynomial coefficients	Global
chem.aLo7_Ascm	0	1	Low polynomial coefficients	Global
chem.aHi7_Ascm	0	1	High polynomial coefficients	Global
chem.Cp_Ascm	R_const*((chem.T<=chem.Tlo_Ascm)*(chem.aLo1_Ascm+chem.Tlo_Ascm*(chem.aLo2_Ascm+chem.Tlo_Ascm*(chem.aLo3_Ascm+chem.Tlo_Ascm*(chem.aLo4_Ascm+chem.Tlo_Ascm*chem.aLo5_Ascm))))+(ch	J/(mol·K)	Heat capacity at constant pressure	Global

Name	Expression	Unit	Description	Selection
	em.T>chem.Tlo_Ascm)*(chem.T<=chem.Tmid_Ascm)*(chem.aLo1_Ascm+chem.T*(chem.aLo2_Ascm+chem.T*(chem.aLo3_Ascm+chem.T*(chem.aLo4_Ascm+chem.T*chem.aLo5_Ascm))))+(chem.T>chem.Tmid_Ascm)*(chem.T<=chem.Thi_Ascm)*(chem.aHi1_Ascm+chem.T*(chem.aHi2_Ascm+chem.T*(chem.aHi3_Ascm+chem.T*(chem.aHi4_Ascm+chem.T*chem.aHi5_Ascm))))+(chem.T>chem.Thi_Ascm)*(chem.aHi1_Ascm+chem.Thi_Ascm*(chem.aHi2_Ascm+chem.Thi_Ascm*(chem.aHi3_Ascm+chem.Thi_Ascm*(chem.aHi4_Ascm+chem.Thi_Ascm*chem.aHi5_Ascm)))))			
chem.h_Ascm	R_const*((chem.T<=chem.Tlo_Ascm)*(chem.aLo6_Ascm+chem.Tlo_Ascm*(chem.aLo1_Ascm+0.5*chem.Tlo_Ascm*(chem.aLo2_Ascm+2*chem.Tlo_Ascm*(chem.aLo3_Ascm+0.75*chem.Tlo_Ascm*(chem.aLo4_Ascm+0.8*chem.Tlo_Ascm*chem.aLo5_Ascm))/3)))+(chem.T>chem.Tlo_Ascm)*(chem.T<=chem.Tmid_Ascm)*(chem.aLo6_Ascm+chem.T*(chem.aLo1_Ascm+0.5*chem.T*(chem.aLo2_Ascm+2*chem.T*(chem.aLo3_Ascm+0.75*chem.T*(chem.aLo4_Ascm+0.8*chem.T*chem.aLo5_Ascm))/3)))+(chem.T>chem.Tmid_Ascm)*(chem.T<=chem.Thi_Ascm)*(chem.aHi6_Ascm+chem.T*(chem.aHi1_Ascm+0.5*chem.T*(chem.aHi2_Ascm+2*chem.T*(chem.aHi3_Ascm+0.75*chem.T*(chem.aHi4_Ascm+0.8*chem.T*chem.aHi5_Ascm))/3)))+(chem.T>chem.Thi_Ascm)*(chem.aHi6_Ascm+chem.Thi_Ascm*(chem.aHi1_Ascm+0.5*chem.Thi_Ascm*(chem.aHi2_Ascm+2*chem.Thi_Ascm*(chem.aHi3_Ascm+0.75*chem.Thi_Ascm*(chem.aHi4_Ascm+0.8*chem.Thi_Ascm*chem.aHi5_Ascm))/3))))	J/mol	Molar enthalpy	Global
chem.s_Ascm	R_const*((chem.T<=chem.Tlo_Ascm)*(chem.aLo7_Ascm+chem.aLo1_Ascm*log(chem.T/1[K])+chem.Tlo_Ascm*(chem.aLo2_Ascm+0.5*chem.Tlo_Ascm*(chem.aLo3_Ascm+2*chem.Tlo_Ascm*(chem.aLo4_Ascm+0.75*chem.Tlo_Ascm*chem.aLo5_Ascm)/3)))+(chem.T>chem.Tlo_Ascm)*(chem.T<=chem.Tmid_Ascm)*(chem.aLo7_Ascm+chem.aLo1_Ascm*log(chem.T/1[K])+chem.	J/(mol·K)	Molar entropy	Global

Name	Expression	Unit	Description	Selection
	T*(chem.aLo2_Ascm+0.5*chem.T*(chem.aLo3_Ascm+2*chem.T*(chem.aLo4_Ascm+0.75*chem.T*chem.aLo5_Ascm)/3)))+(chem.T>chem.Tmid_Ascm)*(chem.T<=chem.Thi_Ascm)*(chem.aHi7_Ascm+chem.aHi1_Ascm*log(chem.T/1[K])+chem.T*(chem.aHi2_Ascm+0.5*chem.T*(chem.aHi3_Ascm+2*chem.T*(chem.aHi4_Ascm+0.75*chem.T*chem.aHi5_Ascm)/3)))+(chem.T>chem.Thi_Ascm)*(chem.aHi7_Ascm+chem.aHi1_Ascm*log(chem.T/1[K])+chem.Thi_Ascm*(chem.aHi2_Ascm+0.5*chem.Thi_Ascm*(chem.aHi3_Ascm+2*chem.Thi_Ascm*(chem.aHi4_Ascm+0.75*chem.Thi_Ascm*chem.aHi5_Ascm)/3))))			

2.9.8 Species: Asc

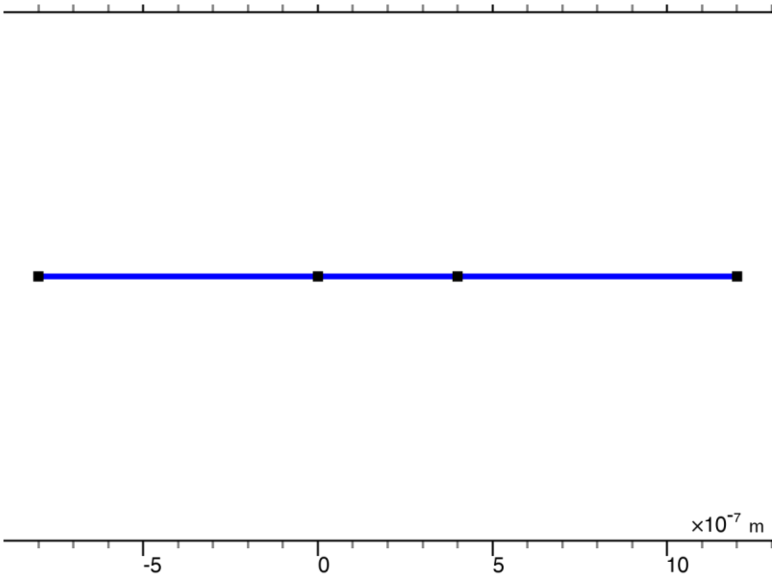

Species: Asc

SELECTION

Geometric entity level	Domain
Selection	Geometry geom1: Dimension 1: All domains

SETTINGS

Description	Value
Species name	Asc

Species Type

SETTINGS

Description	Value
Species type	Bulk species

General Parameters

SETTINGS

Description	Value
Molar mass	PM_Asc
Charge	0

Reaction Rate

SETTINGS

Description	Value
	Automatic

Species Concentration/Activity

SETTINGS

Description	Value
Constant concentration/activity	Off

Additional Source

SETTINGS

Description	Value
Additional source	Off

Species Thermodynamic Expressions

SETTINGS

Description	Value
Species enthalpy	NASA format
Lower temperature limit	300[K]
Middle temperature limit	1000[K]
Upper temperature limit	5000[K]
	0
	0
	0
	0
	0

Description	Value
	0
	0
	0
	0
	0
	0
	0
	0
	0

Variables

Name	Expression	Unit	Description	Selection
chem.M_Asc	PM_Asc	kg/mol	Molar mass	Global
chem.z_Asc	0	1	Charge	Global
chem.Tlo_Asc	300[K]	K	Lower temperature limit	Global
chem.Tmid_Asc	1000[K]	K	Middle temperature limit	Global
chem.Thi_Asc	5000[K]	K	Upper temperature limit	Global
chem.aLo1_Asc	0	1	Low polynomial coefficients	Global
chem.aHi1_Asc	0	1	High polynomial coefficients	Global
chem.aLo2_Asc	0	1/K	Low polynomial coefficients	Global
chem.aHi2_Asc	0	1/K	High polynomial coefficients	Global
chem.aLo3_Asc	0	$1/K^2$	Low polynomial coefficients	Global
chem.aHi3_Asc	0	$1/K^2$	High polynomial coefficients	Global
chem.aLo4_Asc	0	$1/K^3$	Low polynomial coefficients	Global
chem.aHi4_Asc	0	$1/K^3$	High polynomial coefficients	Global
chem.aLo5_Asc	0	$1/K^4$	Low polynomial coefficients	Global
chem.aHi5_Asc	0	$1/K^4$	High polynomial	Global

Name	Expression	Unit	Description	Selection
			coefficients	
chem.aLo6_Asc	0	K	Low polynomial coefficients	Global
chem.aHi6_Asc	0	K	High polynomial coefficients	Global
chem.aLo7_Asc	0	1	Low polynomial coefficients	Global
chem.aHi7_Asc	0	1	High polynomial coefficients	Global
chem.Cp_Asc	R_const*((chem.T<=chem.Tlo_Asc)*(chem.aLo1_Asc+chem.Tlo_Asc*(chem.aLo2_Asc+chem.Tlo_Asc*(chem.aLo3_Asc+chem.Tlo_Asc*(chem.aLo4_Asc+chem.Tlo_Asc*chem.aLo5_Asc))))+(chem.T>chem.Tlo_Asc)*(chem.T<=chem.Tmid_Asc)*(chem.aLo1_Asc+chem.T*(chem.aLo2_Asc+chem.T*(chem.aLo3_Asc+chem.T*(chem.aLo4_Asc+chem.T*chem.aLo5_Asc))))+(chem.T>chem.Tmid_Asc)*(chem.T<=chem.Thi_Asc)*(chem.aHi1_Asc+chem.T*(chem.aHi2_Asc+chem.T*(chem.aHi3_Asc+chem.T*(chem.aHi4_Asc+chem.T*chem.aHi5_Asc))))+(chem.T>chem.Thi_Asc)*(chem.aHi1_Asc+chem.Thi_Asc*(chem.aHi2_Asc+chem.Thi_Asc*(chem.aHi3_Asc+chem.Thi_Asc*(chem.aHi4_Asc+chem.Thi_Asc*chem.aHi5_Asc)))))	J/(mol·K)	Heat capacity at constant pressure	Global
chem.h_Asc	R_const*((chem.T<=chem.Tlo_Asc)*(chem.aLo6_Asc+chem.Tlo_Asc*(chem.aLo1_Asc+0.5*chem.Tlo_Asc*(chem.aLo2_Asc+2*chem.Tlo_Asc*(chem.aLo3_Asc+0.75*chem.Tlo_Asc*(chem.aLo4_Asc+0.8*chem.Tlo_Asc*chem.aLo5_Asc))/3)))+(chem.T>chem.Tlo_Asc)*(chem.T<=chem.Tmid_Asc)*(chem.aLo6_Asc+chem.T*(chem.aLo1_Asc+0.5*chem.T*(chem.aLo2_Asc+2*chem.T*(chem.aLo3_Asc+0.75*chem.T*(chem.aLo4_Asc+0.8*chem.T*chem.aLo5_Asc))/3)))+(chem.T>chem.Tmid_Asc)*(chem.T<=chem.Thi_Asc)*(chem.aHi6_Asc+chem.T*(chem.aHi1_Asc+0.5*chem.T*(chem.aHi2_Asc+2*chem.T*(chem.aHi3_Asc+0	J/mol	Molar enthalpy	Global

Name	Expression	Unit	Description	Selection
	.75*chem.T*(chem.aHi4_Asc+0.8*chem.T*chem.aHi5_Asc))/3)))+(chem.T>chem.Thi_Asc)*(chem.aHi6_Asc+chem.Thi_Asc*(chem.aHi1_Asc+0.5*chem.Thi_Asc*(chem.aHi2_Asc+2*chem.Thi_Asc*(chem.aHi3_Asc+0.75*chem.Thi_Asc*(chem.aHi4_Asc+0.8*chem.Thi_Asc*chem.aHi5_Asc))/3))))			
chem.s_Asc	R_const*((chem.T<=chem.Tlo_Asc)*(chem.aLo7_Asc+chem.aLo1_Asc*log(chem.T/1[K])+chem.Tlo_Asc*(chem.aLo2_Asc+0.5*chem.Tlo_Asc*(chem.aLo3_Asc+2*chem.Tlo_Asc*(chem.aLo4_Asc+0.75*chem.Tlo_Asc*chem.aLo5_Asc)/3)))+(chem.T>chem.Tlo_Asc)*(chem.T<=chem.Tmid_Asc)*(chem.aLo7_Asc+chem.aLo1_Asc*log(chem.T/1[K])+chem.T*(chem.aLo2_Asc+0.5*chem.T*(chem.aLo3_Asc+2*chem.T*(chem.aLo4_Asc+0.75*chem.T*chem.aLo5_Asc)/3)))+(chem.T>chem.Tmid_Asc)*(chem.T<=chem.Thi_Asc)*(chem.aHi7_Asc+chem.aHi1_Asc*log(chem.T/1[K])+chem.T*(chem.aHi2_Asc+0.5*chem.T*(chem.aHi3_Asc+2*chem.T*(chem.aHi4_Asc+0.75*chem.T*chem.aHi5_Asc)/3)))+(chem.T>chem.Thi_Asc)*(chem.aHi7_Asc+chem.aHi1_Asc*log(chem.T/1[K])+chem.Thi_Asc*(chem.aHi2_Asc+0.5*chem.Thi_Asc*(chem.aHi3_Asc+2*chem.Thi_Asc*(chem.aHi4_Asc+0.75*chem.Thi_Asc*chem.aHi5_Asc)/3))))	J/(mol·K)	Molar entropy	Global

2.9.9 Species: CO

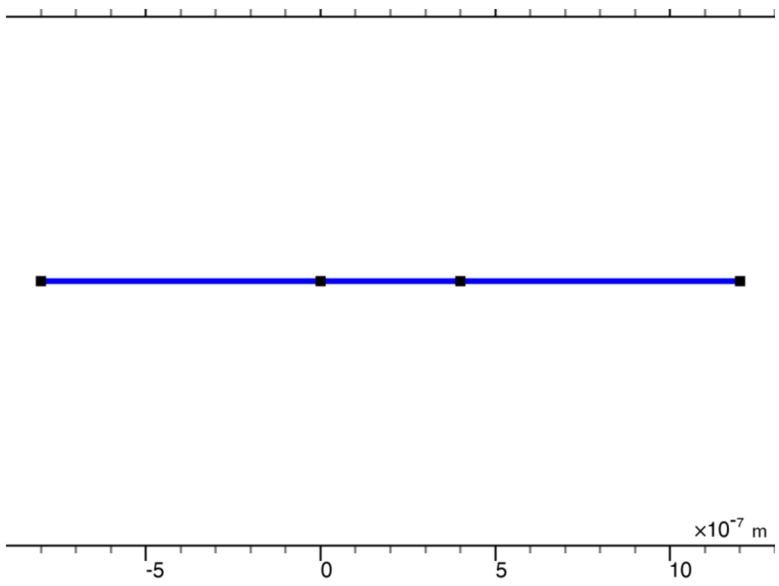

Species: CO

SELECTION

Geometric entity level	Domain
Selection	Geometry geom1: Dimension 1: All domains

SETTINGS

Description	Value
Species name	CO

Species Type

SETTINGS

Description	Value
Species type	Bulk species

General Parameters

SETTINGS

Description	Value
Molar mass	PM_CO
Charge	0

Reaction Rate

SETTINGS

Description	Value

Description	Value
	Automatic

Species Concentration/Activity

SETTINGS

Description	Value
Constant concentration/activity	Off

Additional Source

SETTINGS

Description	Value
Additional source	Off

Species Thermodynamic Expressions

SETTINGS

Description	Value
Species enthalpy	NASA format
Lower temperature limit	300[K]
Middle temperature limit	1000[K]
Upper temperature limit	5000[K]
	0
	0
	0
	0
	0
	0
	0
	0
	0
	0
	0
	0
	0
	0

Variables

Name	Expression	Unit	Description	Selection
chem.M_CO	PM_CO	kg/mol	Molar mass	Global

Name	Expression	Unit	Description	Selection
chem.z_CO	0	1	Charge	Global
chem.Tlo_CO	300[K]	K	Lower temperature limit	Global
chem.Tmid_CO	1000[K]	K	Middle temperature limit	Global
chem.Thi_CO	5000[K]	K	Upper temperature limit	Global
chem.aLo1_CO	0	1	Low polynomial coefficients	Global
chem.aHi1_CO	0	1	High polynomial coefficients	Global
chem.aLo2_CO	0	1/K	Low polynomial coefficients	Global
chem.aHi2_CO	0	1/K	High polynomial coefficients	Global
chem.aLo3_CO	0	$1/K^2$	Low polynomial coefficients	Global
chem.aHi3_CO	0	$1/K^2$	High polynomial coefficients	Global
chem.aLo4_CO	0	$1/K^3$	Low polynomial coefficients	Global
chem.aHi4_CO	0	$1/K^3$	High polynomial coefficients	Global
chem.aLo5_CO	0	$1/K^4$	Low polynomial coefficients	Global
chem.aHi5_CO	0	$1/K^4$	High polynomial coefficients	Global
chem.aLo6_CO	0	K	Low polynomial coefficients	Global
chem.aHi6_CO	0	K	High polynomial coefficients	Global
chem.aLo7_CO	0	1	Low polynomial coefficients	Global
chem.aHi7_CO	0	1	High polynomial coefficients	Global
chem.Cp_CO	R_const*((chem.T<=chem.Tlo_CO)*(chem.aLo1_CO+chem.Tlo_CO*(chem.aLo2_CO+chem.Tlo_CO*(chem.aLo3_CO+chem.Tlo_CO*(chem.aLo4_CO+chem.Tlo_CO*chem.aLo5_CO))))+(chem.T>chem.Tlo_CO)*(chem.T<=chem.T	J/(mol·K)	Heat capacity at constant pressure	Global

Name	Expression	Unit	Description	Selection
	mid_CO)*(chem.aLo1_CO+chem.T*(chem.aLo2_CO+chem.T*(chem.aLo3_CO+chem.T*(chem.aLo4_CO+chem.T*chem.aLo5_CO))))+(chem.T>chem.Tmid_CO)*(chem.T<=chem.Thi_CO)*(chem.aHi1_CO+chem.T*(chem.aHi2_CO+chem.T*(chem.aHi3_CO+chem.T*(chem.aHi4_CO+chem.T*chem.aHi5_CO))))+(chem.T>chem.Thi_CO)*(chem.aHi1_CO+chem.Thi_CO*(chem.aHi2_CO+chem.Thi_CO*(chem.aHi3_CO+chem.Thi_CO*(chem.aHi4_CO+chem.Thi_CO*chem.aHi5_CO)))))			
chem.h_CO	R_const*((chem.T<=chem.Tlo_CO)*(chem.aLo6_CO+chem.Tlo_CO*(chem.aLo1_CO+0.5*chem.Tlo_CO*(chem.aLo2_CO+2*chem.Tlo_CO*(chem.aLo3_CO+0.75*chem.Tlo_CO*(chem.aLo4_CO+0.8*chem.Tlo_CO*chem.aLo5_CO))/3)))+(chem.T>chem.Tlo_CO)*(chem.T<=chem.Tmid_CO)*(chem.aLo6_CO+chem.T*(chem.aLo1_CO+0.5*chem.T*(chem.aLo2_CO+2*chem.T*(chem.aLo3_CO+0.75*chem.T*(chem.aLo4_CO+0.8*chem.T*chem.aLo5_CO))/3)))+(chem.T>chem.Tmid_CO)*(chem.T<=chem.Thi_CO)*(chem.aHi6_CO+chem.T*(chem.aHi1_CO+0.5*chem.T*(chem.aHi2_CO+2*chem.T*(chem.aHi3_CO+0.75*chem.T*(chem.aHi4_CO+0.8*chem.T*chem.aHi5_CO))/3)))+(chem.T>chem.Thi_CO)*(chem.aHi6_CO+chem.Thi_CO*(chem.aHi1_CO+0.5*chem.Thi_CO*(chem.aHi2_CO+2*chem.Thi_CO*(chem.aHi3_CO+0.75*chem.Thi_CO*(chem.aHi4_CO+0.8*chem.Thi_CO*chem.aHi5_CO))/3))))	J/mol	Molar enthalpy	Global
chem.s_CO	R_const*((chem.T<=chem.Tlo_CO)*(chem.aLo7_CO+chem.aLo1_CO*log(chem.T/1[K])+chem.Tlo_CO*(chem.aLo2_CO+0.5*chem.Tlo_CO*(chem.aLo3_CO+2*chem.Tlo_CO*(chem.aLo4_CO+0.75*chem.Tlo_CO*chem.aLo5_CO)/3)))+(chem.T>chem.Tlo_CO)*(chem.T<=chem.Tmid_CO)*(chem.aLo7_CO+chem.aLo1_CO*log(chem.T/1[K])+chem.T*(chem.aLo2_CO+0.5*chem.T*(ch	J/(mol·K)	Molar entropy	Global

Name	Expression	Unit	Description	Selection
	em.aLo3_CO+2*chem.T*(chem.aLo4_CO+0.75*chem.T*chem.aLo5_CO)/3)))+(chem.T>chem.Tmid_CO)*(chem.T<=chem.Thi_CO)*(chem.aHi7_CO+chem.aHi1_CO*log(chem.T/1[K])+chem.T*(chem.aHi2_CO+0.5*chem.T*(chem.aHi3_CO+2*chem.T*(chem.aHi4_CO+0.75*chem.T*chem.aHi5_CO)/3)))+(chem.T>chem.Thi_CO)*(chem.aHi7_CO+chem.aHi1_CO*log(chem.T/1[K])+chem.Thi_CO*(chem.aHi2_CO+0.5*chem.Thi_CO*(chem.aHi3_CO+2*chem.Thi_CO*(chem.aHi4_CO+0.75*chem.Thi_CO*chem.aHi5_CO)/3))))			

2.9.10 Species: H2O

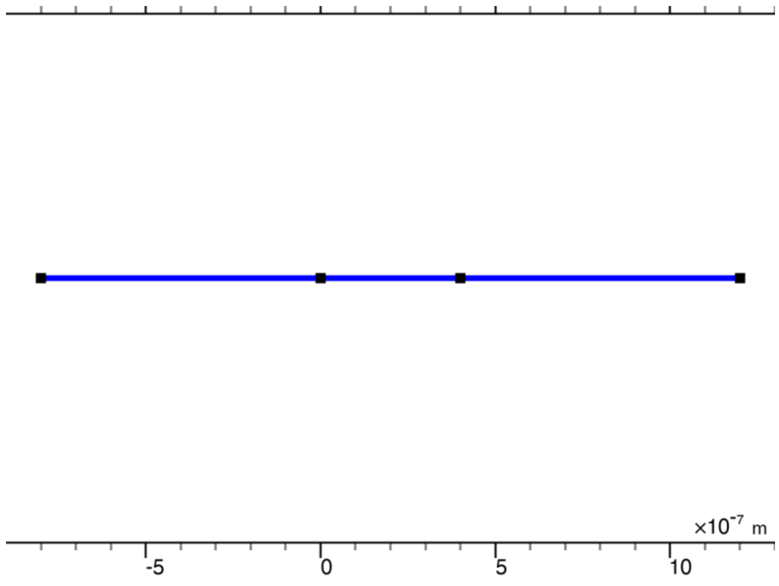

Species: H2O

SELECTION

Geometric entity level	Domain
Selection	Geometry geom1: Dimension 1: All domains

SETTINGS

Description	Value
Species name	H2O

Species Type

SETTINGS

Description	Value
Species type	Solvent

General Parameters

SETTINGS

Description	Value
Molar mass	PM_H2O
Charge	0

Species Thermodynamic Expressions

SETTINGS

Description	Value
Species enthalpy	NASA format
Lower temperature limit	300[K]
Middle temperature limit	1000[K]
Upper temperature limit	5000[K]
	0
	0
	0
	0
	0
	0
	0
	0
	0
	0
	0
	0
	0
	0

Variables

Name	Expression	Unit	Description	Selection	Details
chem.Rsum_H2O	0	$mol/(m^3 \cdot s)$	Reaction rate for species H2O	Global	+ operation
chem.Rsurfsum_H2O	0	$mol/(m^2 \cdot s)$	Surface reaction rate for species H2O	Global	+ operation

Name	Expression	Unit	Description	Selection	Details
chem.Rsurf_H2O	0	mol/(m²·s)	Surface reaction rate for species H2O	Global	+ operation
chem.M_H2O	PM_H2O	kg/mol	Molar mass	Global	
chem.z_H2O	0	1	Charge	Global	
chem.R_H2O	0	mol/(m³·s)	Reaction rate for species H2O	Global	+ operation
chem.Tlo_H2O	300[K]	K	Lower temperature limit	Global	
chem.Tmid_H2O	1000[K]	K	Middle temperature limit	Global	
chem.Thi_H2O	5000[K]	K	Upper temperature limit	Global	
chem.aLo1_H2O	0	1	Low polynomial coefficients	Global	
chem.aHi1_H2O	0	1	High polynomial coefficients	Global	
chem.aLo2_H2O	0	1/K	Low polynomial coefficients	Global	
chem.aHi2_H2O	0	1/K	High polynomial coefficients	Global	
chem.aLo3_H2O	0	1/K²	Low polynomial coefficients	Global	
chem.aHi3_H2O	0	1/K²	High polynomial coefficients	Global	
chem.aLo4_H2O	0	1/K³	Low polynomial coefficients	Global	
chem.aHi4_H2O	0	1/K³	High polynomial coefficients	Global	
chem.aLo5_H2O	0	1/K⁴	Low polynomial coefficients	Global	
chem.aHi5_H2O	0	1/K⁴	High polynomial coefficients	Global	
chem.aLo6_H2O	0	K	Low polynomial coefficients	Global	
chem.aHi6_H2O	0	K	High polynomial coefficients	Global	
chem.aLo7_H2O	0	1	Low polynomial coefficients	Global	
chem.aHi7_H2O	0	1	High polynomial coefficients	Global	

Name	Expression	Unit	Description	Selection	Details
chem.Cp_H2O	R_const*((chem.T<=chem.Tlo_H2O)*(chem.aLo1_H2O+chem.Tlo_H2O*(chem.aLo2_H2O+chem.Tlo_H2O*(chem.aLo3_H2O+chem.Tlo_H2O*(chem.aLo4_H2O+chem.Tlo_H2O*chem.aLo5_H2O))))+(chem.T>chem.Tlo_H2O)*(chem.T<=chem.Tmid_H2O)*(chem.aLo1_H2O+chem.T*(chem.aLo2_H2O+chem.T*(chem.aLo3_H2O+chem.T*(chem.aLo4_H2O+chem.T*chem.aLo5_H2O))))+(chem.T>chem.Tmid_H2O)*(chem.T<=chem.Thi_H2O)*(chem.aHi1_H2O+chem.T*(chem.aHi2_H2O+chem.T*(chem.aHi3_H2O+chem.T*(chem.aHi4_H2O+chem.T*chem.aHi5_H2O))))+(chem.T>chem.Thi_H2O)*(chem.aHi1_H2O+chem.Thi_H2O*(chem.aHi2_H2O+chem.Thi_H2O*(chem.aHi3_H2O+chem.Thi_H2O*(chem.aHi4_H2O+chem.Thi_H2O*chem.aHi5_H2O)))))	J/(mol·K)	Heat capacity at constant pressure	Global	
chem.h_H2O	R_const*((chem.T<=chem.Tlo_H2O)*(chem.aLo6_H2O+chem.Tlo_H2O*(chem.aLo1_H2O+0.5*chem.Tlo_H2O*(chem.aLo2_H2O+2*chem.Tlo_H2O*(chem.aLo3_H2O+0.75*chem.Tlo_H2O*(chem.aLo4_H2O+0.8*chem.Tlo_H2O*chem.aLo5_H2O))/3)))+(chem.T>ch	J/mol	Molar enthalpy	Global	

Name	Expression	Unit	Description	Selection	Details
	em.Tlo_H2O)*(chem.T<=chem.Tmid_H2O)*(chem.aLo6_H2O+chem.T*(chem.aLo1_H2O+0.5*chem.T*(chem.aLo2_H2O+2*chem.T*(chem.aLo3_H2O+0.75*chem.T*(chem.aLo4_H2O+0.8*chem.T*chem.aLo5_H2O))/3)))+(chem.T>chem.Tmid_H2O)*(chem.T<=chem.Thi_H2O)*(chem.aHi6_H2O+chem.T*(chem.aHi1_H2O+0.5*chem.T*(chem.aHi2_H2O+2*chem.T*(chem.aHi3_H2O+0.75*chem.T*(chem.aHi4_H2O+0.8*chem.T*chem.aHi5_H2O))/3)))+(chem.T>chem.Thi_H2O)*(chem.aHi6_H2O+chem.Thi_H2O*(chem.aHi1_H2O+0.5*chem.Thi_H2O*(chem.aHi2_H2O+2*chem.Thi_H2O*(chem.aHi3_H2O+0.75*chem.Thi_H2O*(chem.aHi4_H2O+0.8*chem.Thi_H2O*chem.aHi5_H2O))/3))))				
chem.s_H2O	R_const*((chem.T<=chem.Tlo_H2O)*(chem.aLo7_H2O+chem.aLo1_H2O*log(chem.T/1[K])+chem.Tlo_H2O*(chem.aLo2_H2O+0.5*chem.Tlo_H2O*(chem.aLo3_H2O+2*chem.Tlo_H2O*(chem.aLo4_H2O+0.75*chem.Tlo_H2O*chem.aLo5_H2O)/3)))+(chem.T>chem.Tlo_H2O)*(chem.T<=chem.Tmid_H2O)*(chem.aLo7_H2O+chem.a	J/(mol·K)	Molar entropy	Global	

Name	Expression	Unit	Description	Selection	Details
	Lo1_H2O*log(chem.T/1[K])+chem.T*(chem.aLo2_H2O+0.5*chem.T*(chem.aLo3_H2O+2*chem.T*(chem.aLo4_H2O+0.75*chem.T*chem.aLo5_H2O)/3)))+(chem.T>chem.Tmid_H2O)*(chem.T<=chem.Thi_H2O)*(chem.aHi7_H2O+chem.aHi1_H2O*log(chem.T/1[K])+chem.T*(chem.aHi2_H2O+0.5*chem.T*(chem.aHi3_H2O+2*chem.T*(chem.aHi4_H2O+0.75*chem.T*chem.aHi5_H2O)/3)))+(chem.T>chem.Thi_H2O)*(chem.aHi7_H2O+chem.aHi1_H2O*log(chem.T/1[K])+chem.Thi_H2O*(chem.aHi2_H2O+0.5*chem.Thi_H2O*(chem.aHi3_H2O+2*chem.Thi_H2O*(chem.aHi4_H2O+0.75*chem.Thi_H2O*chem.aHi5_H2O)/3))))				

2.9.11 Surface species: Sr(ads)

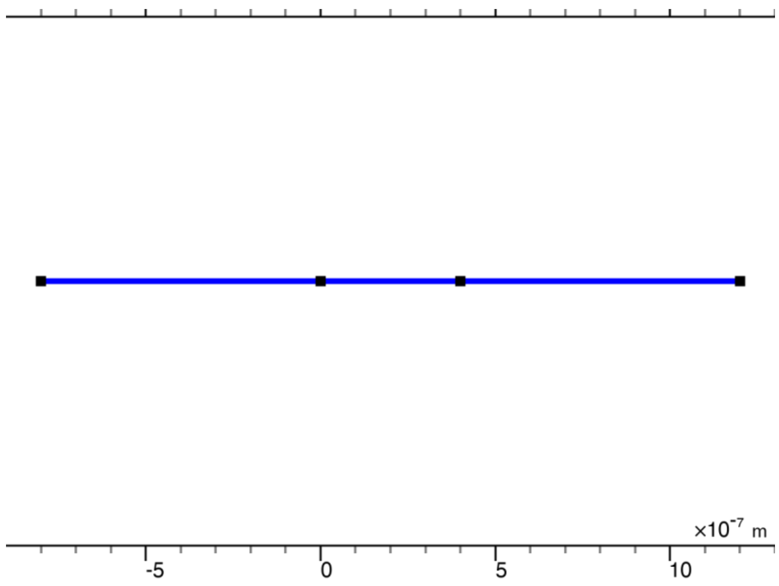

Surface species: Sr(ads)

SELECTION

Geometric entity level	Domain
Selection	Geometry geom1: Dimension 1: All domains

SETTINGS

Description	Value
Species name	Sr_surf

Species Type

SETTINGS

Description	Value
Species type	Surface species

General Parameters

SETTINGS

Description	Value
Molar mass	0.032[kg/mol]
Charge	0

Reaction Rate

SETTINGS

Description	Value

Description	Value
	Automatic

Species Concentration/Activity

SETTINGS

Description	Value
Constant concentration/activity	Off

Additional Source

SETTINGS

Description	Value
Additional source	Off

Species Thermodynamic Expressions

SETTINGS

Description	Value
Species enthalpy	NASA format
Lower temperature limit	300[K]
Middle temperature limit	1000[K]
Upper temperature limit	5000[K]
	0
	0
	0
	0
	0
	0
	0
	0
	0
	0
	0
	0
	0
	0

Variables

Name	Expression	Unit	Description	Selection
chem.M_Sr_su	0.032[kg/mol]	kg/mol	Molar mass	Global

Name	Expression	Unit	Description	Selection
rf				
chem.z_Sr_surf	0	1	Charge	Global
chem.Tlo_Sr_surf	300[K]	K	Lower temperature limit	Global
chem.Tmid_Sr_surf	1000[K]	K	Middle temperature limit	Global
chem.Thi_Sr_surf	5000[K]	K	Upper temperature limit	Global
chem.aLo1_Sr_surf	0	1	Low polynomial coefficients	Global
chem.aHi1_Sr_surf	0	1	High polynomial coefficients	Global
chem.aLo2_Sr_surf	0	1/K	Low polynomial coefficients	Global
chem.aHi2_Sr_surf	0	1/K	High polynomial coefficients	Global
chem.aLo3_Sr_surf	0	$1/K^2$	Low polynomial coefficients	Global
chem.aHi3_Sr_surf	0	$1/K^2$	High polynomial coefficients	Global
chem.aLo4_Sr_surf	0	$1/K^3$	Low polynomial coefficients	Global
chem.aHi4_Sr_surf	0	$1/K^3$	High polynomial coefficients	Global
chem.aLo5_Sr_surf	0	$1/K^4$	Low polynomial coefficients	Global
chem.aHi5_Sr_surf	0	$1/K^4$	High polynomial coefficients	Global
chem.aLo6_Sr_surf	0	K	Low polynomial coefficients	Global
chem.aHi6_Sr_surf	0	K	High polynomial coefficients	Global
chem.aLo7_Sr_surf	0	1	Low polynomial coefficients	Global
chem.aHi7_Sr_surf	0	1	High polynomial coefficients	Global
chem.Cp_Sr_surf	R_const*((chem.T<=chem.Tlo_Sr_surf)*(chem.aLo1_Sr_surf+chem.Tlo_Sr_surf*(chem.aLo2_Sr_surf+chem.Tlo_Sr_surf*(chem.aLo3_Sr_surf+chem.Tlo_Sr_surf*(chem.aL	J/(mol·K)	Heat capacity at constant pressure	Global

Name	Expression	Unit	Description	Selection
	o4_Sr_surf+chem.Tlo_Sr_surf*chem.aLo5_Sr_surf))))+(chem.T>chem.Tlo_Sr_surf)*(chem.T<=chem.Tmid_Sr_surf)*(chem.aLo1_Sr_surf+chem.T*(chem.aLo2_Sr_surf+chem.T*(chem.aLo3_Sr_surf+chem.T*(chem.aLo4_Sr_surf+chem.T*chem.aLo5_Sr_surf))))+(chem.T>chem.Tmid_Sr_surf)*(chem.T<=chem.Thi_Sr_surf)*(chem.aHi1_Sr_surf+chem.T*(chem.aHi2_Sr_surf+chem.T*(chem.aHi3_Sr_surf+chem.T*(chem.aHi4_Sr_surf+chem.T*chem.aHi5_Sr_surf))))+(chem.T>chem.Thi_Sr_surf)*(chem.aHi1_Sr_surf+chem.Thi_Sr_surf*(chem.aHi2_Sr_surf+chem.Thi_Sr_surf*(chem.aHi3_Sr_surf+chem.Thi_Sr_surf*(chem.aHi4_Sr_surf+chem.Thi_Sr_surf*chem.aHi5_Sr_surf)))))			
chem.h_Sr_surf	R_const*((chem.T<=chem.Tlo_Sr_surf)*(chem.aLo6_Sr_surf+chem.Tlo_Sr_surf*(chem.aLo1_Sr_surf+0.5*chem.Tlo_Sr_surf*(chem.aLo2_Sr_surf+2*chem.Tlo_Sr_surf*(chem.aLo3_Sr_surf+0.75*chem.Tlo_Sr_surf*(chem.aLo4_Sr_surf+0.8*chem.Tlo_Sr_surf*chem.aLo5_Sr_surf))/3)))+(chem.T>chem.Tlo_Sr_surf)*(chem.T<=chem.Tmid_Sr_surf)*(chem.aLo6_Sr_surf+chem.T*(chem.aLo1_Sr_surf+0.5*chem.T*(chem.aLo2_Sr_surf+2*chem.T*(chem.aLo3_Sr_surf+0.75*chem.T*(chem.aLo4_Sr_surf+0.8*chem.T*chem.aLo5_Sr_surf))/3)))+(chem.T>chem.Tmid_Sr_surf)*(chem.T<=chem.Thi_Sr_surf)*(chem.aHi6_Sr_surf+chem.T*(chem.aHi1_Sr_surf+0.5*chem.T*(chem.aHi2_Sr_surf+2*chem.T*(chem.aHi3_Sr_surf+0.75*chem.T*(chem.aHi4_Sr_surf+0.8*chem.T*chem.aHi5_Sr_surf))/3)))+(chem.T>chem.Thi_Sr_surf)*(chem.aHi6_Sr_surf+chem.Thi_Sr_surf*(chem.aHi1_Sr_surf+0.5*chem.Thi_Sr_surf*(chem.aHi2_Sr_surf+2*chem.Thi_Sr_surf*(chem.aHi3_Sr_surf+0.75*chem.Thi_Sr_surf*(chem.aHi4_Sr_surf+0.8*chem.Thi_Sr_surf*chem.aHi5_Sr_surf))/3))))	J/mol	Molar enthalpy	Global
chem.s_Sr_surf	R_const*((chem.T<=chem.Tlo_Sr_surf)*(chem.aLo7_Sr_surf+chem.aLo1_Sr_surf*log(chem.T/1[K])+chem.Tlo_Sr_surf*(chem.aLo2_Sr_surf+0.5*chem.Tlo_Sr_surf*(chem.aLo3_Sr_surf+2*chem.Tlo_Sr_surf*(chem.aLo4_Sr_surf+0.75*chem.Tlo_Sr_surf*chem.a	J/(mol·K)	Molar entropy	Global

Name	Expression	Unit	Description	Selection
	Lo5_Sr_surf)/3)))+(chem.T>chem.Tlo_Sr_surf)*(chem.T<=chem.Tmid_Sr_surf)*(chem.aLo7_Sr_surf+chem.aLo1_Sr_surf*log(chem.T/1[K])+chem.T*(chem.aLo2_Sr_surf+0.5*chem.T*(chem.aLo3_Sr_surf+2*chem.T*(chem.aLo4_Sr_surf+0.75*chem.T*chem.aLo5_Sr_surf)/3)))+(chem.T>chem.Tmid_Sr_surf)*(chem.T<=chem.Thi_Sr_surf)*(chem.aHi7_Sr_surf+chem.aHi1_Sr_surf*log(chem.T/1[K])+chem.T*(chem.aHi2_Sr_surf+0.5*chem.T*(chem.aHi3_Sr_surf+2*chem.T*(chem.aHi4_Sr_surf+0.75*chem.T*chem.aHi5_Sr_surf)/3)))+(chem.T>chem.Thi_Sr_surf)*(chem.aHi7_Sr_surf+chem.aHi1_Sr_surf*log(chem.T/1[K])+chem.Thi_Sr_surf*(chem.aHi2_Sr_surf+0.5*chem.Thi_Sr_surf*(chem.aHi3_Sr_surf+2*chem.Thi_Sr_surf*(chem.aHi4_Sr_surf+0.75*chem.Thi_Sr_surf*chem.aHi5_Sr_surf)/3))))			

2.9.12 Species: H2PO4

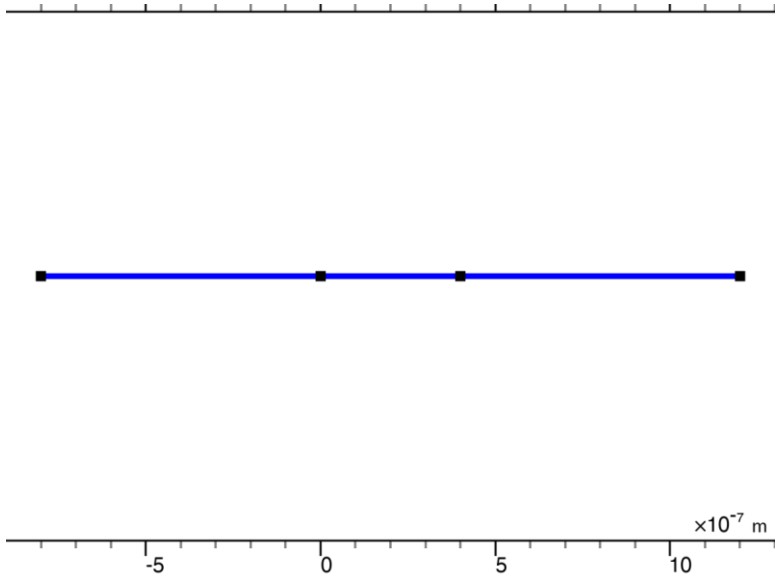

Species: H2PO4

SELECTION

Geometric entity level	Domain
Selection	Geometry geom1: Dimension 1: All domains

SETTINGS

Description	Value
Species name	H2PO4

Species Type

SETTINGS

Description	Value
Species type	Bulk species

General Parameters

SETTINGS

Description	Value
Molar mass	PM_H2PO4
Charge	-1

Reaction Rate

SETTINGS

Description	Value
	Automatic

Species Concentration/Activity

SETTINGS

Description	Value
Constant concentration/activity	Off

Additional Source

SETTINGS

Description	Value
Additional source	Off

Species Thermodynamic Expressions

SETTINGS

Description	Value
Species enthalpy	NASA format
Lower temperature limit	300[K]
Middle temperature limit	1000[K]
Upper temperature limit	5000[K]
	0
	0

Description	Value
	0
	0
	0
	0
	0
	0
	0
	0
	0
	0
	0
	0

Variables

Name	Expression	Unit	Description	Selection
chem.M_H2PO4	PM_H2PO4	kg/mol	Molar mass	Global
chem.z_H2PO4	-1	1	Charge	Global
chem.Tlo_H2PO4	300[K]	K	Lower temperature limit	Global
chem.Tmid_H2PO4	1000[K]	K	Middle temperature limit	Global
chem.Thi_H2PO4	5000[K]	K	Upper temperature limit	Global
chem.aLo1_H2PO4	0	1	Low polynomial coefficients	Global
chem.aHi1_H2PO4	0	1	High polynomial coefficients	Global
chem.aLo2_H2PO4	0	1/K	Low polynomial coefficients	Global
chem.aHi2_H2PO4	0	1/K	High polynomial coefficients	Global
chem.aLo3_H2PO4	0	$1/K^2$	Low polynomial coefficients	Global
chem.aHi3_H2PO4	0	$1/K^2$	High polynomial coefficients	Global
chem.aLo4_H2PO4	0	$1/K^3$	Low polynomial coefficients	Global

Name	Expression	Unit	Description	Selection
chem.aHi4_H2PO4	0	1/K³	High polynomial coefficients	Global
chem.aLo5_H2PO4	0	1/K⁴	Low polynomial coefficients	Global
chem.aHi5_H2PO4	0	1/K⁴	High polynomial coefficients	Global
chem.aLo6_H2PO4	0	K	Low polynomial coefficients	Global
chem.aHi6_H2PO4	0	K	High polynomial coefficients	Global
chem.aLo7_H2PO4	0	1	Low polynomial coefficients	Global
chem.aHi7_H2PO4	0	1	High polynomial coefficients	Global
chem.Cp_H2PO4	R_const*((chem.T<=chem.Tlo_H2PO4)*(chem.aLo1_H2PO4+chem.Tlo_H2PO4*(chem.aLo2_H2PO4+chem.Tlo_H2PO4*(chem.aLo3_H2PO4+chem.Tlo_H2PO4*(chem.aLo4_H2PO4+chem.Tlo_H2PO4*chem.aLo5_H2PO4))))+(chem.T>chem.Tlo_H2PO4)*(chem.T<=chem.Tmid_H2PO4)*(chem.aLo1_H2PO4+chem.T*(chem.aLo2_H2PO4+chem.T*(chem.aLo3_H2PO4+chem.T*(chem.aLo4_H2PO4+chem.T*chem.aLo5_H2PO4))))+(chem.T>chem.Tmid_H2PO4)*(chem.T<=chem.Thi_H2PO4)*(chem.aHi1_H2PO4+chem.T*(chem.aHi2_H2PO4+chem.T*(chem.aHi3_H2PO4+chem.T*(chem.aHi4_H2PO4+chem.T*chem.aHi5_H2PO4))))+(chem.T>chem.Thi_H2PO4)*(chem.aHi1_H2PO4+chem.Thi_H2PO4*(chem.aHi2_H2PO4+chem.Thi_H2PO4*(chem.aHi3_H2PO4+chem.Thi_H2PO4*(chem.aHi4_H2PO4+chem.Thi_H2PO4*chem.aHi5_H2PO4)))))	J/(mol·K)	Heat capacity at constant pressure	Global
chem.h_H2PO4	R_const*((chem.T<=chem.Tlo_H2PO4)*(chem.aLo6_H2PO4+chem.Tlo_H2PO4*(chem.aLo1_H2PO4+0.5*chem.Tlo_H2PO4*(chem.aLo2_H2PO4+2*chem.Tlo_H2PO4*(chem.aLo3_H2PO4+0.75*chem.Tlo_H2PO4*(chem.aLo4_H2PO4+0.8*chem.Tlo_H2PO4*chem.aLo5_H2PO4))/3)))+(chem.T>chem.Tlo_H2PO4)*(chem.T<=chem.Tmid_H2PO4)*(chem.aLo6_H2PO4+chem.T*(chem.aLo1_H2PO4+0.5*chem.T*(chem.aLo2_H2PO4+2*chem.T*(chem.aLo3_H2PO4+0.	J/mol	Molar enthalpy	Global

Name	Expression	Unit	Description	Selection
	75*chem.T*(chem.aLo4_H2PO4+0.8*chem.T*chem.aLo5_H2PO4))/3)))+(chem.T>chem.Tmid_H2PO4)*(chem.T<=chem.Thi_H2PO4)*(chem.aHi6_H2PO4+chem.T*(chem.aHi1_H2PO4+0.5*chem.T*(chem.aHi2_H2PO4+2*chem.T*(chem.aHi3_H2PO4+0.75*chem.T*(chem.aHi4_H2PO4+0.8*chem.T*chem.aHi5_H2PO4))/3)))+(chem.T>chem.Thi_H2PO4)*(chem.aHi6_H2PO4+chem.Thi_H2PO4*(chem.aHi1_H2PO4+0.5*chem.Thi_H2PO4*(chem.aHi2_H2PO4+2*chem.Thi_H2PO4*(chem.aHi3_H2PO4+0.75*chem.Thi_H2PO4*(chem.aHi4_H2PO4+0.8*chem.Thi_H2PO4*chem.aHi5_H2PO4))/3))))			
chem.s_H2PO4	R_const*((chem.T<=chem.Tlo_H2PO4)*(chem.aLo7_H2PO4+chem.aLo1_H2PO4*log(chem.T/1[K])+chem.Tlo_H2PO4*(chem.aLo2_H2PO4+0.5*chem.Tlo_H2PO4*(chem.aLo3_H2PO4+2*chem.Tlo_H2PO4*(chem.aLo4_H2PO4+0.75*chem.Tlo_H2PO4*chem.aLo5_H2PO4)/3)))+(chem.T>chem.Tlo_H2PO4)*(chem.T<=chem.Tmid_H2PO4)*(chem.aLo7_H2PO4+chem.aLo1_H2PO4*log(chem.T/1[K])+chem.T*(chem.aLo2_H2PO4+0.5*chem.T*(chem.aLo3_H2PO4+2*chem.T*(chem.aLo4_H2PO4+0.75*chem.T*chem.aLo5_H2PO4)/3)))+(chem.T>chem.Tmid_H2PO4)*(chem.T<=chem.Thi_H2PO4)*(chem.aHi7_H2PO4+chem.aHi1_H2PO4*log(chem.T/1[K])+chem.T*(chem.aHi2_H2PO4+0.5*chem.T*(chem.aHi3_H2PO4+2*chem.T*(chem.aHi4_H2PO4+0.75*chem.T*chem.aHi5_H2PO4)/3)))+(chem.T>chem.Thi_H2PO4)*(chem.aHi7_H2PO4+chem.aHi1_H2PO4*log(chem.T/1[K])+chem.Thi_H2PO4*(chem.aHi2_H2PO4+0.5*chem.Thi_H2PO4*(chem.aHi3_H2PO4+2*chem.Thi_H2PO4*(chem.aHi4_H2PO4+0.75*chem.Thi_H2PO4*chem.aHi5_H2PO4)/3))))	J/(mol·K)	Molar entropy	Global

2.9.13 Species: HPO4

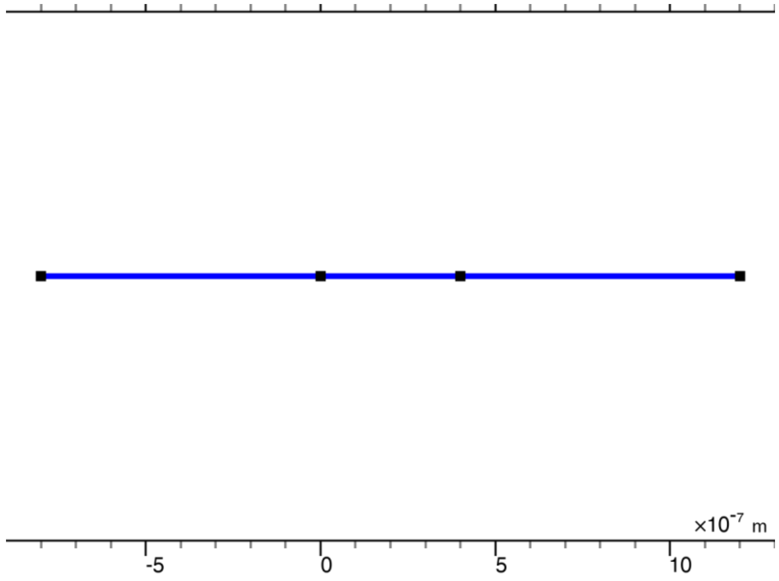

Species: HPO4

SELECTION

Geometric entity level	Domain
Selection	Geometry geom1: Dimension 1: All domains

SETTINGS

Description	Value
Species name	HPO4

Species Type

SETTINGS

Description	Value
Species type	Bulk species

General Parameters

SETTINGS

Description	Value
Molar mass	PM_HPO4
Charge	-2

Reaction Rate

SETTINGS

Description	Value

Description	Value
	Automatic

Species Concentration/Activity

SETTINGS

Description	Value
Constant concentration/activity	Off

Additional Source

SETTINGS

Description	Value
Additional source	Off

Species Thermodynamic Expressions

SETTINGS

Description	Value
Species enthalpy	NASA format
Lower temperature limit	300[K]
Middle temperature limit	1000[K]
Upper temperature limit	5000[K]
	0
	0
	0
	0
	0
	0
	0
	0
	0
	0
	0
	0
	0
	0

Variables

Name	Expression	Unit	Description	Selection
chem.M_HPO	PM_HPO4	kg/mol	Molar mass	Global

Name	Expression	Unit	Description	Selection
4				
chem.z_HPO4	-2	1	Charge	Global
chem.Tlo_HPO4	300[K]	K	Lower temperature limit	Global
chem.Tmid_HPO4	1000[K]	K	Middle temperature limit	Global
chem.Thi_HPO4	5000[K]	K	Upper temperature limit	Global
chem.aLo1_HPO4	0	1	Low polynomial coefficients	Global
chem.aHi1_HPO4	0	1	High polynomial coefficients	Global
chem.aLo2_HPO4	0	1/K	Low polynomial coefficients	Global
chem.aHi2_HPO4	0	1/K	High polynomial coefficients	Global
chem.aLo3_HPO4	0	1/K²	Low polynomial coefficients	Global
chem.aHi3_HPO4	0	1/K²	High polynomial coefficients	Global
chem.aLo4_HPO4	0	1/K³	Low polynomial coefficients	Global
chem.aHi4_HPO4	0	1/K³	High polynomial coefficients	Global
chem.aLo5_HPO4	0	1/K⁴	Low polynomial coefficients	Global
chem.aHi5_HPO4	0	1/K⁴	High polynomial coefficients	Global
chem.aLo6_HPO4	0	K	Low polynomial coefficients	Global
chem.aHi6_HPO4	0	K	High polynomial coefficients	Global
chem.aLo7_HPO4	0	1	Low polynomial coefficients	Global
chem.aHi7_HPO4	0	1	High polynomial coefficients	Global
chem.Cp_HPO4	R_const*((chem.T<=chem.Tlo_HPO4)*(chem.aLo1_HPO4+chem.Tlo_HPO4*(chem.aLo2_HPO4+chem.Tlo_HPO4*(chem.aLo3_HPO4+chem.Tlo_HPO4*(chem.aLo4_HPO4+chem.Tlo_HPO4*chem.aLo5_HPO4))))+	J/(mol·K)	Heat capacity at constant pressure	Global

Name	Expression	Unit	Description	Selection
	(chem.T>chem.Tlo_HPO4)*(chem.T<=chem.Tmid_HPO4)*(chem.aLo1_HPO4+chem.T*(chem.aLo2_HPO4+chem.T*(chem.aLo3_HPO4+chem.T*(chem.aLo4_HPO4+chem.T*chem.aLo5_HPO4))))+(chem.T>chem.Tmid_HPO4)*(chem.T<=chem.Thi_HPO4)*(chem.aHi1_HPO4+chem.T*(chem.aHi2_HPO4+chem.T*(chem.aHi3_HPO4+chem.T*(chem.aHi4_HPO4+chem.T*chem.aHi5_HPO4))))+(chem.T>chem.Thi_HPO4)*(chem.aHi1_HPO4+chem.Thi_HPO4*(chem.aHi2_HPO4+chem.Thi_HPO4*(chem.aHi3_HPO4+chem.Thi_HPO4*(chem.aHi4_HPO4+chem.Thi_HPO4*chem.aHi5_HPO4)))))			
chem.h_HPO4	R_const*((chem.T<=chem.Tlo_HPO4)*(chem.aLo6_HPO4+chem.Tlo_HPO4*(chem.aLo1_HPO4+0.5*chem.Tlo_HPO4*(chem.aLo2_HPO4+2*chem.Tlo_HPO4*(chem.aLo3_HPO4+0.75*chem.Tlo_HPO4*(chem.aLo4_HPO4+0.8*chem.Tlo_HPO4*chem.aLo5_HPO4))/3)))+(chem.T>chem.Tlo_HPO4)*(chem.T<=chem.Tmid_HPO4)*(chem.aLo6_HPO4+chem.T*(chem.aLo1_HPO4+0.5*chem.T*(chem.aLo2_HPO4+2*chem.T*(chem.aLo3_HPO4+0.75*chem.T*(chem.aLo4_HPO4+0.8*chem.T*chem.aLo5_HPO4))/3)))+(chem.T>chem.Tmid_HPO4)*(chem.T<=chem.Thi_HPO4)*(chem.aHi6_HPO4+chem.T*(chem.aHi1_HPO4+0.5*chem.T*(chem.aHi2_HPO4+2*chem.T*(chem.aHi3_HPO4+0.75*chem.T*(chem.aHi4_HPO4+0.8*chem.T*chem.aHi5_HPO4))/3)))+(chem.T>chem.Thi_HPO4)*(chem.aHi6_HPO4+chem.Thi_HPO4*(chem.aHi1_HPO4+0.5*chem.Thi_HPO4*(chem.aHi2_HPO4+2*chem.Thi_HPO4*(chem.aHi3_HPO4+0.75*chem.Thi_HPO4*(chem.aHi4_HPO4+0.8*chem.Thi_HPO4*chem.aHi5_HPO4))/3))))	J/mol	Molar enthalpy	Global
chem.s_HPO4	R_const*((chem.T<=chem.Tlo_HPO4)*(chem.aLo7_HPO4+chem.aLo1_HPO4*log(chem.T/1[K])+chem.Tlo_HPO4*(chem.aLo2_HPO4+0.5*chem.Tlo_HPO4*(chem.aLo3_HPO4+2*chem.Tlo_HPO4*(chem.aLo4_HPO4+0.75*chem.Tlo_HPO4*chem.aLo5_HPO4)/3)))+(chem.T>chem.Tlo_HPO4)*(chem.T<=chem.Tmid_HPO4)*(chem.aLo7_HPO4+chem.aLo1_HPO4*log(chem.T/1[K])+	J/(mol·K)	Molar entropy	Global

Name	Expression	Unit	Description	Selection
	chem.T*(chem.aLo2_HPO4+0.5*chem.T*(chem.aLo3_HPO4+2*chem.T*(chem.aLo4_HPO4+0.75*chem.T*chem.aLo5_HPO4)/3)))+(chem.T>chem.Tmid_HPO4)*(chem.T<=chem.Thi_HPO4)*(chem.aHi7_HPO4+chem.aHi1_HPO4*log(chem.T/1[K])+chem.T*(chem.aHi2_HPO4+0.5*chem.T*(chem.aHi3_HPO4+2*chem.T*(chem.aHi4_HPO4+0.75*chem.T*chem.aHi5_HPO4)/3)))+(chem.T>chem.Thi_HPO4)*(chem.aHi7_HPO4+chem.aHi1_HPO4*log(chem.T/1[K])+chem.Thi_HPO4*(chem.aHi2_HPO4+0.5*chem.Thi_HPO4*(chem.aHi3_HPO4+2*chem.Thi_HPO4*(chem.aHi4_HPO4+0.75*chem.Thi_HPO4*chem.aHi5_HPO4)/3))))			

2.10 CHEMISTRY H2O OXIDATION

USED PRODUCTS

COMSOL Multiphysics
Chemical Reaction Engineering Module

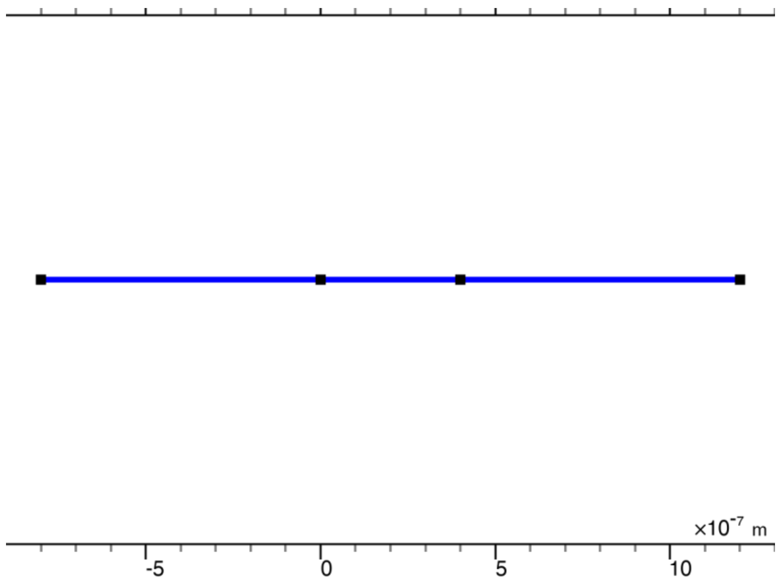

Chemistry H2O oxidation

SELECTION

Geometric entity level	Domain
Selection	Geometry geom1: Dimension 1: All domains

EQUATIONS

$$R_i = \sum_j R_{ij}, \quad R_{\mathrm{ads},i} = \sum_j R_{\mathrm{ads},ij}$$

2.10.1 Interface Settings

Discretization

SETTINGS

Description	Value
Element order	Quadratic

SETTINGS

Description	Value
Equation form	Study controlled

Model Input

SETTINGS

Description	Value
Temperature	Common model input
Pressure	Common model input

Mixture Properties

SETTINGS

Description	Value
Type	Diluted species
Phase	Liquid

Species Matching

SETTINGS

Description	Value
Species solved for	User defined

Species	Type	Molar concentration	Value	Reaction rate	Unit	Equation formulation
Asc						
Ascm						
H2O						
H2PO4						
HPO4						
O2						

Species	Type	Molar concentration	Value	Reaction rate	Unit	Equation formulation
Variable						
Variable						
Solvent						
Variable						
Variable						
Variable						

Species	Type	Molar concentration	Value	Reaction rate	Unit	Equation formulation
User defined						
User defined						
User defined						
User defined						
User defined						
User defined						

Species	Type	Molar concentration	Value	Reaction rate	Unit	Equation formulation
cAsc						
cAscm						
cH2O						
cH2PO4						
cHPO4						
cO2						

Species	Type	Molar concentration	Value	Reaction rate	Unit	Equation formulation
chem2.R_Asc						
chem2.R_Ascm						
solvent						
chem2.R_H						

Species	Type	Molar concentration	Value	Reaction rate	Unit	Equation formulation
2PO4						
chem2.R_HPO4						
chem2.R_O2						

Species	Type	Molar concentration	Value	Reaction rate	Unit	Equation formulation
mol/m^3						
mol/m^3						
mol/m^3						
mol/m^3						
mol/m^3						
mol/m^3						

Species	Type	Molar concentration	Value	Reaction rate	Unit	Equation formulation
cAsc						
cAscm						
cH2O						
cH2PO4						
cHPO4						
cO2						

Species	Species concentration type	Surface concentration
So(ads)	Variable	csSo

Calculate Transport Properties

SETTINGS

Description	Value
Calculate mixture properties	Off

Activity

SETTINGS

Description	Value
Use activity	Off

CHEMKIN Import for Species Properties

SETTINGS

Description	Value
Thermo input file	
Transport input file	

Pellet Chemistry

SETTINGS

Description	Value
Define variables for porous pellets	Off

2.10.2 Variables

Name	Expression	Unit	Description	Selection	Details
chem2.T	model.input.T	K	Temperature	Domains 1–3	Meta
chem2.p	model.input.p	Pa	Pressure	Domains 1–3	Meta
chem2.eeqMean	chem2.eeqSum/chem2.nEr	V	Mean equilibrium potential	Global	
chem2.csum	eps+chem2.c_Asc+chem2.c_Ascm+chem2.c_H2O+chem2.c_H2PO4+chem2.c_HPO4+chem2.c_O2	mol/m³	Total concentration	Global	
chem2.m_Asc	max(chem2.c_Asc,0)/chem2.csum	1	Molar fraction	Global	
chem2.m_Ascm	max(chem2.c_Ascm,0)/chem2.csum	1	Molar fraction	Global	
chem2.m_H2O	max(chem2.c_H2O,0)/chem2.csum	1	Molar fraction	Global	
chem2.m_H2PO4	max(chem2.c_H2PO4,0)/chem2.csum	1	Molar fraction	Global	
chem2.m_HPO4	max(chem2.c_HPO4,0)/chem2.csum	1	Molar fraction	Global	
chem2.m_O2	max(chem2.c_O2,0)/chem2.csum	1	Molar fraction	Global	
chem2.rhosum	eps+chem2.c_Asc*chem2.M_Asc+chem2.c_Ascm*chem2.M_Ascm+chem2.c_H2O*chem2.M_H2O+chem2.c_H2PO4*chem2.M_H2PO4+chem2.c_HP	kg/m³	Density	Global	

Name	Expression	Unit	Description	Selection	Details
	O4*chem2.M_HPO4+chem2.c_O2*chem2.M_O2				
chem2.w_Asc	chem2.c_Asc*chem2.M_Asc/chem2.rhosum	1	Mass fraction	Global	
chem2.w_Ascm	chem2.c_Ascm*chem2.M_Ascm/chem2.rhosum	1	Mass fraction	Global	
chem2.w_H2O	chem2.c_H2O*chem2.M_H2O/chem2.rhosum	1	Mass fraction	Global	
chem2.w_H2PO4	chem2.c_H2PO4*chem2.M_H2PO4/chem2.rhosum	1	Mass fraction	Global	
chem2.w_HPO4	chem2.c_HPO4*chem2.M_HPO4/chem2.rhosum	1	Mass fraction	Global	
chem2.w_O2	chem2.c_O2*chem2.M_O2/chem2.rhosum	1	Mass fraction	Global	
chem2.c_Asc	cAsc	mol/m^3	Concentration	Global	
chem2.Rsum_Asc	0	$mol/(m^3 \cdot s)$	Reaction rate for species Asc	Global	+ operation
chem2.R_Asc	0	$mol/(m^3 \cdot s)$	Reaction rate for species Asc	Global	+ operation
chem2.Rsurf_Asc	0	$mol/(m^2 \cdot s)$	Surface reaction rate for species Asc	Global	+ operation
chem2.Rsurfsum_Asc	0	$mol/(m^2 \cdot s)$	Surface reaction rate for species Asc	Global	+ operation
chem2.c_Ascm	cAscm	mol/m^3	Concentration	Global	
chem2.Rsum_Ascm	0	$mol/(m^3 \cdot s)$	Reaction rate for species Ascm	Global	+ operation
chem2.R_Ascm	0	$mol/(m^3 \cdot s)$	Reaction rate for species Ascm	Global	+ operation
chem2.Rsurf_Ascm	0	$mol/(m^2 \cdot s)$	Surface reaction rate for species Ascm	Global	+ operation
chem2.Rsurfsum_Ascm	0	$mol/(m^2 \cdot s)$	Surface reaction rate for species Ascm	Global	+ operation
chem2.c_H2O	cH2O	mol/m^3	Concentration	Global	

Name	Expression	Unit	Description	Selection	Details
chem2.Rsum_H2O	0	mol/(m³·s)	Reaction rate for species H2O	Global	+ operation
chem2.c_H2PO4	cH2PO4	mol/m³	Concentration	Global	
chem2.Rsum_H2PO4	0	mol/(m³·s)	Reaction rate for species H2PO4	Global	+ operation
chem2.R_H2PO4	0	mol/(m³·s)	Reaction rate for species H2PO4	Global	+ operation
chem2.Rsurf_H2PO4	0	mol/(m²·s)	Surface reaction rate for species H2PO4	Global	+ operation
chem2.Rsurfsum_H2PO4	0	mol/(m²·s)	Surface reaction rate for species H2PO4	Global	+ operation
chem2.c_HPO4	cHPO4	mol/m³	Concentration	Global	
chem2.Rsum_HPO4	0	mol/(m³·s)	Reaction rate for species HPO4	Global	+ operation
chem2.R_HPO4	0	mol/(m³·s)	Reaction rate for species HPO4	Global	+ operation
chem2.Rsurf_HPO4	0	mol/(m²·s)	Surface reaction rate for species HPO4	Global	+ operation
chem2.Rsurfsum_HPO4	0	mol/(m²·s)	Surface reaction rate for species HPO4	Global	+ operation
chem2.c_O2	cO2	mol/m³	Concentration	Global	
chem2.Rsum_O2	0	mol/(m³·s)	Reaction rate for species O2	Global	+ operation
chem2.R_O2	0	mol/(m³·s)	Reaction rate for species O2	Global	+ operation
chem2.Rsurf_O2	0	mol/(m²·s)	Surface reaction rate for species O2	Global	+ operation
chem2.Rsurfsum_O2	0	mol/(m²·s)	Surface reaction rate for species O2	Global	+ operation
chem2.csurf_So_surf	csSo	mol/m²	Surface concentration	Global	
chem2.Rsum_So_surf	0	mol/(m²·s)	Surface reaction rate for surface species So_surf	Global	+ operation
chem2.R_So_surf	0	mol/(m²·s)	Surface reaction rate for surface species So_surf	Global	+ operation

Name	Expression	Unit	Description	Selection	Details
chem2.Qheat	0		Heat source of reactions	Global	+ operation
chem2.Qtot	chem2.Qheat	W/m³	Heat source of reactions	Global	
chem2.Qs	0	W/m²	Surface heat source of reaction	Global	+ operation
chem2.Qb	chem2.Qs	W/m²	Surface heat source of reaction	Global	
chem2.Mn	chem2.M_H2O	kg/mol	Mean molar mass	Global	

2.10.3 1: Surface: H2O+2Asc+2HPO4+So(ads)=>1/2O2+2Ascm+2H2PO4+So(ads)

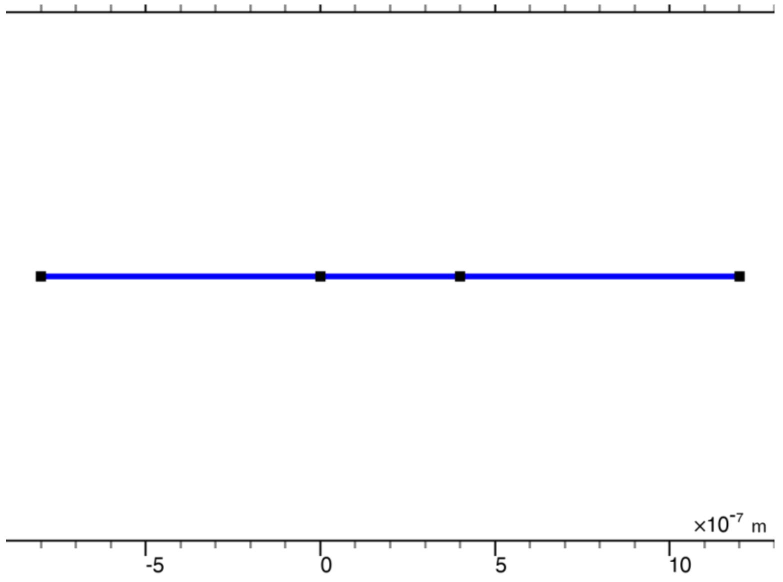

1: Surface: H2O+2Asc+2HPO4+So(ads)=>1/2O2+2Ascm+2H2PO4+So(ads)

SELECTION

Geometric entity level	Domain
Selection	Geometry geom1: Dimension 1: All domains

Reaction Formula

SETTINGS

Description	Value
Formula	H2O + 2Asc + 2HPO4 + So(ads)=>1/2O2 + 2Ascm + 2H2PO4 + So(ads)
Reaction type	Irreversible

Reaction Rate

SETTINGS

Description	Value
Reaction rate	Mass action law
Description	Overall forward reaction order: 5 (volumetric), 1 (surface)

Rate Constants

SETTINGS

Description	Value
Use Arrhenius expressions	Off
Forward rate constant	kf_H2O*step1(t)

Reaction Thermodynamic Properties

SETTINGS

Description	Value
Enthalpy of reaction	Automatic
Entropy of reaction	Automatic
Heat source of reaction	Automatic

Variables

Name	Expression	Unit	Description	Selection	Details
chem2.Rsurf_Asc	-2*chem2.r_1	mol/(m²·s)	Surface reaction rate for species Asc	Global	+ operation
chem2.Rsurfsum_Asc	-2*chem2.r_1	mol/(m²·s)	Surface reaction rate for species Asc	Global	+ operation
chem2.Rsurf_Ascm	2*chem2.r_1	mol/(m²·s)	Surface reaction rate for species Ascm	Global	+ operation
chem2.Rsurfsum_Ascm	2*chem2.r_1	mol/(m²·s)	Surface reaction rate for species Ascm	Global	+ operation
chem2.Rsurf_H2PO4	2*chem2.r_1	mol/(m²·s)	Surface reaction rate for species H2PO4	Global	+ operation
chem2.Rsurfsum_H2PO4	2*chem2.r_1	mol/(m²·s)	Surface reaction rate for species H2PO4	Global	+ operation
chem2.Rsurf_HPO4	-2*chem2.r_1	mol/(m²·s)	Surface reaction rate for species HPO4	Global	+ operation
chem2.Rsurfsum_HPO	-2*chem2.r_1	mol/(m²·s)	Surface reaction	Global	+ operation

Name	Expression	Unit	Description	Selection	Details
4			rate for species HPO4		
chem2.Rsurf_O2	0.5*chem2.r_1	mol/(m²·s)	Surface reaction rate for species O2	Global	+ operation
chem2.Rsurfsum_O2	0.5*chem2.r_1	mol/(m²·s)	Surface reaction rate for species O2	Global	+ operation
chem2.Rsum_So_surf	0	mol/(m²·s)	Surface reaction rate for surface species So_surf	Global	+ operation
chem2.R_So_surf	0	mol/(m²·s)	Surface reaction rate for surface species So_surf	Global	+ operation
chem2.Qs	-chem2.r_1*chem2.H_1	W/m²	Surface heat source of reaction	Global	+ operation
chem2.Rsurfsum_H2O	0	mol/(m²·s)	Surface reaction rate for species H2O	Global	+ operation
chem2.Rsurf_H2O	0	mol/(m²·s)	Surface reaction rate for species H2O	Global	+ operation
chem2.kf_1	kf_H2O*step1(t)	$m^{15}/(s \cdot mol^5)$	Forward rate constant	Global	
chem2.r_1	chem2.kf_1*chem2.c_H2O*chem2.csurf_So_surf*chem2.c_Asc*chem2.c_HPO4	mol/(m²·s)	Reaction rate	Global	
chem2.H_1	-chem2.h_H2O-2*chem2.h_Asc-2*chem2.h_HPO4+0.5*chem2.h_O2+2*chem2.h_Ascm+2*chem2.h_H2PO4	J/mol	Enthalpy of reaction	Global	
chem2.S_1	-chem2.s_H2O-2*chem2.s_Asc-2*chem2.s_HPO4+0.5*chem2.s_O2+2*chem2.s_Ascm+2*chem2.s_H2PO4	J/(mol·K)	Entropy of reaction	Global	
chem2.Qs_1	-chem2.r_1*chem2.H_1	W/m²	Surface heat source of	Global	

Name	Expression	Unit	Description	Selection	Details
			reaction		

2.10.4 Species: H2O

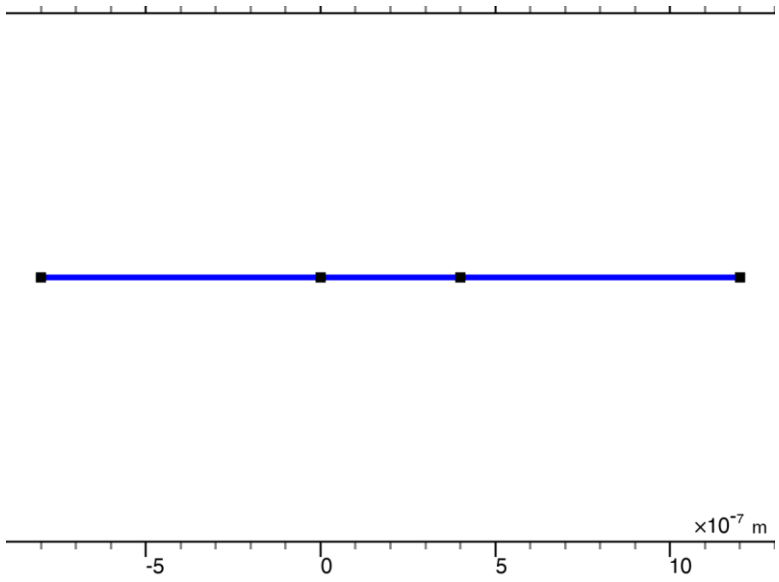

Species: H2O

SELECTION

Geometric entity level	Domain
Selection	Geometry geom1: Dimension 1: All domains

SETTINGS

Description	Value
Species name	H2O

Species Type

SETTINGS

Description	Value
Species type	Solvent

General Parameters

SETTINGS

Description	Value
Molar mass	PM_H2O
Charge	0

Species Thermodynamic Expressions

SETTINGS

Description	Value
Species enthalpy	NASA format
Lower temperature limit	300[K]
Middle temperature limit	1000[K]
Upper temperature limit	5000[K]
	0
	0
	0
	0
	0
	0
	0
	0
	0
	0
	0
	0
	0
	0

Variables

Name	Expression	Unit	Description	Selection	Details
chem2.Rsum_H2O	0	$mol/(m^3 \cdot s)$	Reaction rate for species H2O	Global	+ operation
chem2.Rsurfsum_H2O	0	$mol/(m^2 \cdot s)$	Surface reaction rate for species H2O	Global	+ operation
chem2.Rsurf_H2O	0	$mol/(m^2 \cdot s)$	Surface reaction rate for species H2O	Global	+ operation
chem2.M_H2O	PM_H2O	kg/mol	Molar mass	Global	
chem2.z_H2O	0	1	Charge	Global	
chem2.R_H2O	0	$mol/(m^3 \cdot s)$	Reaction rate for species H2O	Global	+ operation
chem2.Tlo_H2O	300[K]	K	Lower temperature limit	Global	
chem2.Tmid_H2O	1000[K]	K	Middle	Global	

Name	Expression	Unit	Description	Selection	Details
			temperature limit		
chem2.Thi_H2O	5000[K]	K	Upper temperature limit	Global	
chem2.aLo1_H2O	0	1	Low polynomial coefficients	Global	
chem2.aHi1_H2O	0	1	High polynomial coefficients	Global	
chem2.aLo2_H2O	0	1/K	Low polynomial coefficients	Global	
chem2.aHi2_H2O	0	1/K	High polynomial coefficients	Global	
chem2.aLo3_H2O	0	$1/K^2$	Low polynomial coefficients	Global	
chem2.aHi3_H2O	0	$1/K^2$	High polynomial coefficients	Global	
chem2.aLo4_H2O	0	$1/K^3$	Low polynomial coefficients	Global	
chem2.aHi4_H2O	0	$1/K^3$	High polynomial coefficients	Global	
chem2.aLo5_H2O	0	$1/K^4$	Low polynomial coefficients	Global	
chem2.aHi5_H2O	0	$1/K^4$	High polynomial coefficients	Global	
chem2.aLo6_H2O	0	K	Low polynomial coefficients	Global	
chem2.aHi6_H2O	0	K	High polynomial coefficients	Global	
chem2.aLo7_H2O	0	1	Low polynomial coefficients	Global	
chem2.aHi7_H2O	0	1	High polynomial coefficients	Global	
chem2.Cp_H2O	R_const*((chem2.T<=chem2.Tlo_H2O)*(chem2.aLo1_H2O+chem2.Tlo_H2O*(chem2.aLo2_H2O+chem2.Tlo_H2O*(chem2.aLo3_H2O+chem2.Tlo_H2O*(chem2.aLo4_H2O+chem2.Tlo_H2O*chem2.aLo5_H2O))))+(chem2.T>chem2.Tlo_H2O)*(c	J/(mol·K)	Heat capacity at constant pressure	Global	

Name	Expression	Unit	Description	Selection	Details
	hem2.T<=chem2.Tmid_H2O)*(chem2.aLo1_H2O+chem2.T*(chem2.aLo2_H2O+chem2.T*(chem2.aLo3_H2O+chem2.T*(chem2.aLo4_H2O+chem2.T*chem2.aLo5_H2O))))+(chem2.T>chem2.Tmid_H2O)*(chem2.T<=chem2.Thi_H2O)*(chem2.aHi1_H2O+chem2.T*(chem2.aHi2_H2O+chem2.T*(chem2.aHi3_H2O+chem2.T*(chem2.aHi4_H2O+chem2.T*chem2.aHi5_H2O))))+(chem2.T>chem2.Thi_H2O)*(chem2.aHi1_H2O+chem2.Thi_H2O*(chem2.aHi2_H2O+chem2.Thi_H2O*(chem2.aHi3_H2O+chem2.Thi_H2O*(chem2.aHi4_H2O+chem2.Thi_H2O*chem2.aHi5_H2O))))				
chem2.h_H2O	R_const*((chem2.T<=chem2.Tlo_H2O)*(chem2.aLo6_H2O+chem2.Tlo_H2O*(chem2.aLo1_H2O+0.5*chem2.Tlo_H2O*(chem2.aLo2_H2O+2*chem2.Tlo_H2O*(chem2.aLo3_H2O+0.75*chem2.Tlo_H2O*(chem2.aLo4_H2O+0.8*chem2.Tlo_H2O*chem2.aLo5_H2O))/3)))+(chem2.T>chem2.Tlo_H2O)*(chem2.T<=chem2.Tmid_H2O)*(chem2.aLo6_H2O+chem2.T*(chem2.aLo1_H2O+0.5*chem2.T*(chem2.aLo2_H2O+2*chem2.T*(chem2.aLo3_	J/mol	Molar enthalpy	Global	

Name	Expression	Unit	Description	Selection	Details
	H2O+0.75*chem2.T*(chem2.aLo4_H2O+0.8*chem2.T*chem2.aLo5_H2O))/3)))+(chem2.T>chem2.Tmid_H2O)*(chem2.T<=chem2.Thi_H2O)*(chem2.aHi6_H2O+chem2.T*(chem2.aHi1_H2O+0.5*chem2.T*(chem2.aHi2_H2O+2*chem2.T*(chem2.aHi3_H2O+0.75*chem2.T*(chem2.aHi4_H2O+0.8*chem2.T*chem2.aHi5_H2O))/3)))+(chem2.T>chem2.Thi_H2O)*(chem2.aHi6_H2O+chem2.Thi_H2O*(chem2.aHi1_H2O+0.5*chem2.Thi_H2O*(chem2.aHi2_H2O+2*chem2.Thi_H2O*(chem2.aHi3_H2O+0.75*chem2.Thi_H2O*(chem2.aHi4_H2O+0.8*chem2.Thi_H2O*chem2.aHi5_H2O))/3))))				
chem2.s_H2O	R_const*((chem2.T<=chem2.Tlo_H2O)*(chem2.aLo7_H2O+chem2.aLo1_H2O*log(chem2.T/1[K])+chem2.Tlo_H2O*(chem2.aLo2_H2O+0.5*chem2.Tlo_H2O*(chem2.aLo3_H2O+2*chem2.Tlo_H2O*(chem2.aLo4_H2O+0.75*chem2.Tlo_H2O*chem2.aLo5_H2O)/3)))+(chem2.T>chem2.Tlo_H2O)*(chem2.T<=chem2.Tmid_H2O)*(chem2.aLo7_H2O+chem2.aLo1_H2O*log(chem2.T/1[K])+chem2.T*(chem2.aLo2_H2O+0.5*chem2.T*(chem2.a	J/(mol·K)	Molar entropy	Global	

Name	Expression	Unit	Description	Selection	Details
	Lo3_H2O+2*chem2.T*(chem2.aLo4_H2O+0.75*chem2.T*chem2.aLo5_H2O)/3)))+(chem2.T>chem2.Tmid_H2O)*(chem2.T<=chem2.Thi_H2O)*(chem2.aHi7_H2O+chem2.aHi1_H2O*log(chem2.T/1[K])+chem2.T*(chem2.aHi2_H2O+0.5*chem2.T*(chem2.aHi3_H2O+2*chem2.T*(chem2.aHi4_H2O+0.75*chem2.T*chem2.aHi5_H2O)/3)))+(chem2.T>chem2.Thi_H2O)*(chem2.aHi7_H2O+chem2.aHi1_H2O*log(chem2.T/1[K])+chem2.Thi_H2O*(chem2.aHi2_H2O+0.5*chem2.Thi_H2O*(chem2.aHi3_H2O+2*chem2.Thi_H2O*(chem2.aHi4_H2O+0.75*chem2.Thi_H2O*chem2.aHi5_H2O)/3))))				

2.10.5 Species: Asc

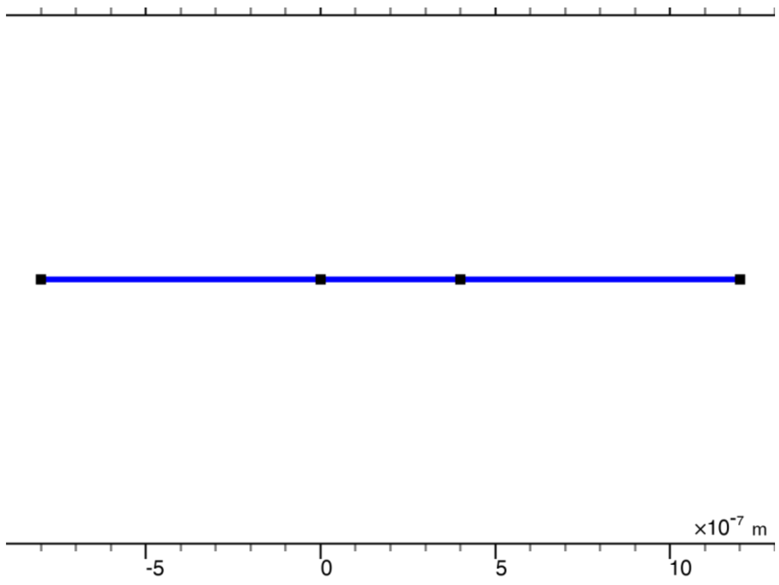

Species: Asc

SELECTION

Geometric entity level	Domain
Selection	Geometry geom1: Dimension 1: All domains

SETTINGS

Description	Value
Species name	Asc

Species Type

SETTINGS

Description	Value
Species type	Bulk species

General Parameters

SETTINGS

Description	Value
Molar mass	PM_Asc
Charge	0

Reaction Rate

SETTINGS

Description	Value
	Automatic

Species Concentration/Activity

SETTINGS

Description	Value
Constant concentration/activity	Off

Additional Source

SETTINGS

Description	Value
Additional source	Off

Species Thermodynamic Expressions

SETTINGS

Description	Value
Species enthalpy	NASA format
Lower temperature limit	300[K]

Description	Value
Middle temperature limit	1000[K]
Upper temperature limit	5000[K]
	0
	0
	0
	0
	0
	0
	0
	0
	0
	0
	0
	0
	0
	0

Variables

Name	Expression	Unit	Description	Selection
chem2.M_Asc	PM_Asc	kg/mol	Molar mass	Global
chem2.z_Asc	0	1	Charge	Global
chem2.Tlo_Asc	300[K]	K	Lower temperature limit	Global
chem2.Tmid_Asc	1000[K]	K	Middle temperature limit	Global
chem2.Thi_Asc	5000[K]	K	Upper temperature limit	Global
chem2.aLo1_Asc	0	1	Low polynomial coefficients	Global
chem2.aHi1_Asc	0	1	High polynomial coefficients	Global
chem2.aLo2_Asc	0	1/K	Low polynomial coefficients	Global
chem2.aHi2_Asc	0	1/K	High polynomial coefficients	Global
chem2.aLo3_Asc	0	$1/K^2$	Low polynomial coefficients	Global
chem2.aHi3_As	0	$1/K^2$	High polynomial	Global

Name	Expression	Unit	Description	Selection
c			coefficients	
chem2.aLo4_Asc	0	1/K³	Low polynomial coefficients	Global
chem2.aHi4_Asc	0	1/K³	High polynomial coefficients	Global
chem2.aLo5_Asc	0	1/K⁴	Low polynomial coefficients	Global
chem2.aHi5_Asc	0	1/K⁴	High polynomial coefficients	Global
chem2.aLo6_Asc	0	K	Low polynomial coefficients	Global
chem2.aHi6_Asc	0	K	High polynomial coefficients	Global
chem2.aLo7_Asc	0	1	Low polynomial coefficients	Global
chem2.aHi7_Asc	0	1	High polynomial coefficients	Global
chem2.Cp_Asc	R_const*((chem2.T<=chem2.Tlo_Asc)*(chem2.aLo1_Asc+chem2.Tlo_Asc*(chem2.aLo2_Asc+chem2.Tlo_Asc*(chem2.aLo3_Asc+chem2.Tlo_Asc*(chem2.aLo4_Asc+chem2.Tlo_Asc*chem2.aLo5_Asc))))+(chem2.T>chem2.Tlo_Asc)*(chem2.T<=chem2.Tmid_Asc)*(chem2.aLo1_Asc+chem2.T*(chem2.aLo2_Asc+chem2.T*(chem2.aLo3_Asc+chem2.T*(chem2.aLo4_Asc+chem2.T*chem2.aLo5_Asc))))+(chem2.T>chem2.Tmid_Asc)*(chem2.T<=chem2.Thi_Asc)*(chem2.aHi1_Asc+chem2.T*(chem2.aHi2_Asc+chem2.T*(chem2.aHi3_Asc+chem2.T*(chem2.aHi4_Asc+chem2.T*chem2.aHi5_Asc))))+(chem2.T>chem2.Thi_Asc)*(chem2.aHi1_Asc+chem2.Thi_Asc*(chem2.aHi2_Asc+chem2.Thi_Asc*(chem2.aHi3_Asc+chem2.Thi_Asc*(chem2.aHi4_Asc+chem2.Thi_Asc*chem2.aHi5_Asc)))))	J/(mol·K)	Heat capacity at constant pressure	Global
chem2.h_Asc	R_const*((chem2.T<=chem2.Tlo_Asc)*(chem2.aLo6_Asc+chem2.Tlo_Asc*(chem2.aLo1_Asc+0.5*chem2.Tlo_Asc*(chem2.aLo2_Asc+2*chem2.Tlo_Asc*(chem2.aLo3_Asc+0.75*chem2.Tlo_Asc*(chem2.aLo4_Asc+0.8*chem2.Tlo_Asc*chem2.aLo5_Asc))/3)))+(chem2.T>chem2.Tlo_Asc)*(chem2.T<=chem2.Tmid_Asc)*(chem2.aLo6_	J/mol	Molar enthalpy	Global

Name	Expression	Unit	Description	Selection
	Asc+chem2.T*(chem2.aLo1_Asc+0.5*chem2.T*(chem2.aLo2_Asc+2*chem2.T*(chem2.aLo3_Asc+0.75*chem2.T*(chem2.aLo4_Asc+0.8*chem2.T*chem2.aLo5_Asc))/3)))+(chem2.T>chem2.Tmid_Asc)*(chem2.T<=chem2.Thi_Asc)*(chem2.aHi6_Asc+chem2.T*(chem2.aHi1_Asc+0.5*chem2.T*(chem2.aHi2_Asc+2*chem2.T*(chem2.aHi3_Asc+0.75*chem2.T*(chem2.aHi4_Asc+0.8*chem2.T*chem2.aHi5_Asc))/3)))+(chem2.T>chem2.Thi_Asc)*(chem2.aHi6_Asc+chem2.Thi_Asc*(chem2.aHi1_Asc+0.5*chem2.Thi_Asc*(chem2.aHi2_Asc+2*chem2.Thi_Asc*(chem2.aHi3_Asc+0.75*chem2.Thi_Asc*(chem2.aHi4_Asc+0.8*chem2.Thi_Asc*chem2.aHi5_Asc))/3))))			
chem2.s_Asc	R_const*((chem2.T<=chem2.Tlo_Asc)*(chem2.aLo7_Asc+chem2.aLo1_Asc*log(chem2.T/1[K])+chem2.Tlo_Asc*(chem2.aLo2_Asc+0.5*chem2.Tlo_Asc*(chem2.aLo3_Asc+2*chem2.Tlo_Asc*(chem2.aLo4_Asc+0.75*chem2.Tlo_Asc*chem2.aLo5_Asc)/3)))+(chem2.T>chem2.Tlo_Asc)*(chem2.T<=chem2.Tmid_Asc)*(chem2.aLo7_Asc+chem2.aLo1_Asc*log(chem2.T/1[K])+chem2.T*(chem2.aLo2_Asc+0.5*chem2.T*(chem2.aLo3_Asc+2*chem2.T*(chem2.aLo4_Asc+0.75*chem2.T*chem2.aLo5_Asc)/3)))+(chem2.T>chem2.Tmid_Asc)*(chem2.T<=chem2.Thi_Asc)*(chem2.aHi7_Asc+chem2.aHi1_Asc*log(chem2.T/1[K])+chem2.T*(chem2.aHi2_Asc+0.5*chem2.T*(chem2.aHi3_Asc+2*chem2.T*(chem2.aHi4_Asc+0.75*chem2.T*chem2.aHi5_Asc)/3)))+(chem2.T>chem2.Thi_Asc)*(chem2.aHi7_Asc+chem2.aHi1_Asc*log(chem2.T/1[K])+chem2.Thi_Asc*(chem2.aHi2_Asc+0.5*chem2.Thi_Asc*(chem2.aHi3_Asc+2*chem2.Thi_Asc*(chem2.aHi4_Asc+0.75*chem2.Thi_Asc*chem2.aHi5_Asc)/3))))	J/(mol·K)	Molar entropy	Global

2.10.6 Species: HPO4

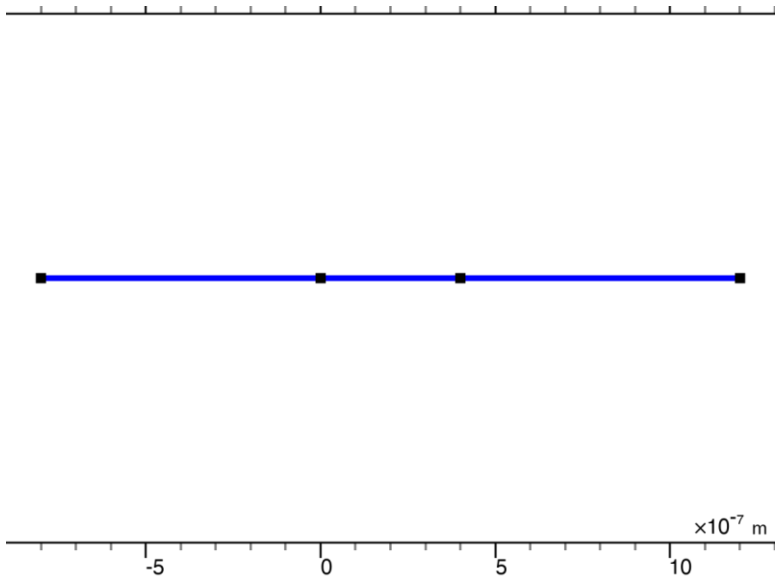

Species: HPO4

SELECTION

Geometric entity level	Domain
Selection	Geometry geom1: Dimension 1: All domains

SETTINGS

**Description**	**Value**
Species name	HPO4

Species Type

SETTINGS

**Description**	**Value**
Species type	Bulk species

General Parameters

SETTINGS

**Description**	**Value**
Molar mass	PM_HPO4
Charge	-2

Reaction Rate

SETTINGS

**Description**	**Value**

Description	Value
	Automatic

Species Concentration/Activity

SETTINGS

Description	Value
Constant concentration/activity	Off

Additional Source

SETTINGS

Description	Value
Additional source	Off

Species Thermodynamic Expressions

SETTINGS

Description	Value
Species enthalpy	NASA format
Lower temperature limit	300[K]
Middle temperature limit	1000[K]
Upper temperature limit	5000[K]
	0
	0
	0
	0
	0
	0
	0
	0
	0
	0
	0
	0
	0
	0

Variables

Name	Expression	Unit	Description	Selection
chem2.M_HPO	PM_HPO4	kg/mol	Molar mass	Global

Name	Expression	Unit	Description	Selection
4				
chem2.z_HPO4	-2	1	Charge	Global
chem2.Tlo_HPO4	300[K]	K	Lower temperature limit	Global
chem2.Tmid_HPO4	1000[K]	K	Middle temperature limit	Global
chem2.Thi_HPO4	5000[K]	K	Upper temperature limit	Global
chem2.aLo1_HPO4	0	1	Low polynomial coefficients	Global
chem2.aHi1_HPO4	0	1	High polynomial coefficients	Global
chem2.aLo2_HPO4	0	1/K	Low polynomial coefficients	Global
chem2.aHi2_HPO4	0	1/K	High polynomial coefficients	Global
chem2.aLo3_HPO4	0	$1/K^2$	Low polynomial coefficients	Global
chem2.aHi3_HPO4	0	$1/K^2$	High polynomial coefficients	Global
chem2.aLo4_HPO4	0	$1/K^3$	Low polynomial coefficients	Global
chem2.aHi4_HPO4	0	$1/K^3$	High polynomial coefficients	Global
chem2.aLo5_HPO4	0	$1/K^4$	Low polynomial coefficients	Global
chem2.aHi5_HPO4	0	$1/K^4$	High polynomial coefficients	Global
chem2.aLo6_HPO4	0	K	Low polynomial coefficients	Global
chem2.aHi6_HPO4	0	K	High polynomial coefficients	Global
chem2.aLo7_HPO4	0	1	Low polynomial coefficients	Global
chem2.aHi7_HPO4	0	1	High polynomial coefficients	Global
chem2.Cp_HPO4	R_const*((chem2.T<=chem2.Tlo_HPO4)*(chem2.aLo1_HPO4+chem2.Tlo_HPO4*(chem2.aLo2_HPO4+chem2.Tlo_HPO4*(chem2.aLo3_HPO4+chem2.Tlo_HPO4*(chem2.aLo4_HPO4+chem2.Tlo_HPO4*che	J/(mol·K)	Heat capacity at constant pressure	Global

Name	Expression	Unit	Description	Selection
	m2.aLo5_HPO4))))+(chem2.T>chem2.Tlo_HPO4)*(chem2.T<=chem2.Tmid_HPO4)*(chem2.aLo1_HPO4+chem2.T*(chem2.aLo2_HPO4+chem2.T*(chem2.aLo3_HPO4+chem2.T*(chem2.aLo4_HPO4+chem2.T*chem2.aLo5_HPO4))))+(chem2.T>chem2.Tmid_HPO4)*(chem2.T<=chem2.Thi_HPO4)*(chem2.aHi1_HPO4+chem2.T*(chem2.aHi2_HPO4+chem2.T*(chem2.aHi3_HPO4+chem2.T*(chem2.aHi4_HPO4+chem2.T*chem2.aHi5_HPO4))))+(chem2.T>chem2.Thi_HPO4)*(chem2.aHi1_HPO4+chem2.Thi_HPO4*(chem2.aHi2_HPO4+chem2.Thi_HPO4*(chem2.aHi3_HPO4+chem2.Thi_HPO4*(chem2.aHi4_HPO4+chem2.Thi_HPO4*chem2.aHi5_HPO4)))))			
chem2.h_HPO4	R_const*((chem2.T<=chem2.Tlo_HPO4)*(chem2.aLo6_HPO4+chem2.Tlo_HPO4*(chem2.aLo1_HPO4+0.5*chem2.Tlo_HPO4*(chem2.aLo2_HPO4+2*chem2.Tlo_HPO4*(chem2.aLo3_HPO4+0.75*chem2.Tlo_HPO4*(chem2.aLo4_HPO4+0.8*chem2.Tlo_HPO4*chem2.aLo5_HPO4))/3)))+(chem2.T>chem2.Tlo_HPO4)*(chem2.T<=chem2.Tmid_HPO4)*(chem2.aLo6_HPO4+chem2.T*(chem2.aLo1_HPO4+0.5*chem2.T*(chem2.aLo2_HPO4+2*chem2.T*(chem2.aLo3_HPO4+0.75*chem2.T*(chem2.aLo4_HPO4+0.8*chem2.T*chem2.aLo5_HPO4))/3)))+(chem2.T>chem2.Tmid_HPO4)*(chem2.T<=chem2.Thi_HPO4)*(chem2.aHi6_HPO4+chem2.T*(chem2.aHi1_HPO4+0.5*chem2.T*(chem2.aHi2_HPO4+2*chem2.T*(chem2.aHi3_HPO4+0.75*chem2.T*(chem2.aHi4_HPO4+0.8*chem2.T*chem2.aHi5_HPO4))/3)))+(chem2.T>chem2.Thi_HPO4)*(chem2.aHi6_HPO4+chem2.Thi_HPO4*(chem2.aHi1_HPO4+0.5*chem2.Thi_HPO4*(chem2.aHi2_HPO4+2*chem2.Thi_HPO4*(chem2.aHi3_HPO4+0.75*chem2.Thi_HPO4*(chem2.aHi4_HPO4+0.8*chem2.Thi_HPO4*chem2.aHi5_HPO4))/3))))	J/mol	Molar enthalpy	Global
chem2.s_HPO4	R_const*((chem2.T<=chem2.Tlo_HPO4)*(chem2.aLo7_HPO4+chem2.aLo1_HPO4*log(chem2.T/1[K])+chem2.Tlo_HPO4*(chem2.aLo2_HPO4+0.5*chem2.Tlo_HPO4	J/(mol·K)	Molar entropy	Global

Name	Expression	Unit	Description	Selection
	*(chem2.aLo3_HPO4+2*chem2.Tlo_HPO4*(chem2.aLo4_HPO4+0.75*chem2.Tlo_HPO4*chem2.aLo5_HPO4)/3)))+(chem2.T>chem2.Tlo_HPO4)*(chem2.T<=chem2.Tmid_HPO4)*(chem2.aLo7_HPO4+chem2.aLo1_HPO4*log(chem2.T/1[K])+chem2.T*(chem2.aLo2_HPO4+0.5*chem2.T*(chem2.aLo3_HPO4+2*chem2.T*(chem2.aLo4_HPO4+0.75*chem2.T*chem2.aLo5_HPO4)/3)))+(chem2.T>chem2.Tmid_HPO4)*(chem2.T<=chem2.Thi_HPO4)*(chem2.aHi7_HPO4+chem2.aHi1_HPO4*log(chem2.T/1[K])+chem2.T*(chem2.aHi2_HPO4+0.5*chem2.T*(chem2.aHi3_HPO4+2*chem2.T*(chem2.aHi4_HPO4+0.75*chem2.T*chem2.aHi5_HPO4)/3)))+(chem2.T>chem2.Thi_HPO4)*(chem2.aHi7_HPO4+chem2.aHi1_HPO4*log(chem2.T/1[K])+chem2.Thi_HPO4*(chem2.aHi2_HPO4+0.5*chem2.Thi_HPO4*(chem2.aHi3_HPO4+2*chem2.Thi_HPO4*(chem2.aHi4_HPO4+0.75*chem2.Thi_HPO4*chem2.aHi5_HPO4)/3))))			

2.10.7 Surface species: So(ads)

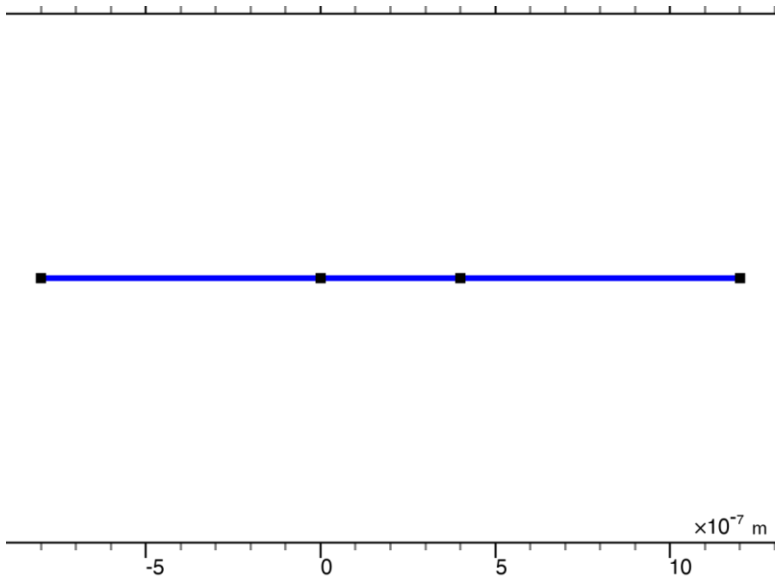

Surface species: So(ads)

SELECTION

Geometric entity level	Domain

Selection	Geometry geom1: Dimension 1: All domains

SETTINGS

Description	Value
Species name	So_surf

Species Type

SETTINGS

Description	Value
Species type	Surface species

General Parameters

SETTINGS

Description	Value
Molar mass	0.032[kg/mol]
Charge	0

Reaction Rate

SETTINGS

Description	Value
	Automatic

Species Concentration/Activity

SETTINGS

Description	Value
Constant concentration/activity	Off

Additional Source

SETTINGS

Description	Value
Additional source	Off

Species Thermodynamic Expressions

SETTINGS

Description	Value
Species enthalpy	NASA format
Lower temperature limit	300[K]
Middle temperature limit	1000[K]
Upper temperature limit	5000[K]

Description	Value
	0
	0
	0
	0
	0
	0
	0
	0
	0
	0
	0
	0
	0
	0

Variables

Name	Expression	Unit	Description	Selection
chem2.M_So_surf	0.032[kg/mol]	kg/mol	Molar mass	Global
chem2.z_So_surf	0	1	Charge	Global
chem2.Tlo_So_surf	300[K]	K	Lower temperature limit	Global
chem2.Tmid_So_surf	1000[K]	K	Middle temperature limit	Global
chem2.Thi_So_surf	5000[K]	K	Upper temperature limit	Global
chem2.aLo1_So_surf	0	1	Low polynomial coefficients	Global
chem2.aHi1_So_surf	0	1	High polynomial coefficients	Global
chem2.aLo2_So_surf	0	1/K	Low polynomial coefficients	Global
chem2.aHi2_So_surf	0	1/K	High polynomial coefficients	Global
chem2.aLo3_So_surf	0	$1/K^2$	Low polynomial coefficients	Global
chem2.aHi3_So_surf	0	$1/K^2$	High polynomial coefficients	Global

Name	Expression	Unit	Description	Selection
chem2.aLo4_So_surf	0	$1/K^3$	Low polynomial coefficients	Global
chem2.aHi4_So_surf	0	$1/K^3$	High polynomial coefficients	Global
chem2.aLo5_So_surf	0	$1/K^4$	Low polynomial coefficients	Global
chem2.aHi5_So_surf	0	$1/K^4$	High polynomial coefficients	Global
chem2.aLo6_So_surf	0	K	Low polynomial coefficients	Global
chem2.aHi6_So_surf	0	K	High polynomial coefficients	Global
chem2.aLo7_So_surf	0	1	Low polynomial coefficients	Global
chem2.aHi7_So_surf	0	1	High polynomial coefficients	Global
chem2.Cp_So_surf	R_const*((chem2.T<=chem2.Tlo_So_surf)*(chem2.aLo1_So_surf+chem2.Tlo_So_surf*(chem2.aLo2_So_surf+chem2.Tlo_So_surf*(chem2.aLo3_So_surf+chem2.Tlo_So_surf*(chem2.aLo4_So_surf+chem2.Tlo_So_surf*chem2.aLo5_So_surf))))+(chem2.T>chem2.Tlo_So_surf)*(chem2.T<=chem2.Tmid_So_surf)*(chem2.aLo1_So_surf+chem2.T*(chem2.aLo2_So_surf+chem2.T*(chem2.aLo3_So_surf+chem2.T*(chem2.aLo4_So_surf+chem2.T*chem2.aLo5_So_surf))))+(chem2.T>chem2.Tmid_So_surf)*(chem2.T<=chem2.Thi_So_surf)*(chem2.aHi1_So_surf+chem2.T*(chem2.aHi2_So_surf+chem2.T*(chem2.aHi3_So_surf+chem2.T*(chem2.aHi4_So_surf+chem2.T*chem2.aHi5_So_surf))))+(chem2.T>chem2.Thi_So_surf)*(chem2.aHi1_So_surf+chem2.Thi_So_surf*(chem2.aHi2_So_surf+chem2.Thi_So_surf*(chem2.aHi3_So_surf+chem2.Thi_So_surf*(chem2.aHi4_So_surf+chem2.Thi_So_surf*chem2.aHi5_So_surf)))))	J/(mol·K)	Heat capacity at constant pressure	Global
chem2.h_So_surf	R_const*((chem2.T<=chem2.Tlo_So_surf)*(chem2.aLo6_So_surf+chem2.Tlo_So_surf*(chem2.aLo1_So_surf+0.5*chem2.Tlo_So_surf*(chem2.aLo2_So_surf+2*chem2.Tlo_So_surf*(chem2.aLo3_So_surf+0.75*	J/mol	Molar enthalpy	Global

Name	Expression	Unit	Description	Selection
	chem2.Tlo_So_surf*(chem2.aLo4_So_surf+0.8*chem2.Tlo_So_surf*chem2.aLo5_So_surf))/3)))+(chem2.T>chem2.Tlo_So_surf)*(chem2.T<=chem2.Tmid_So_surf)*(chem2.aLo6_So_surf+chem2.T*(chem2.aLo1_So_surf+0.5*chem2.T*(chem2.aLo2_So_surf+2*chem2.T*(chem2.aLo3_So_surf+0.75*chem2.T*(chem2.aLo4_So_surf+0.8*chem2.T*chem2.aLo5_So_surf))/3)))+(chem2.T>chem2.Tmid_So_surf)*(chem2.T<=chem2.Thi_So_surf)*(chem2.aHi6_So_surf+chem2.T*(chem2.aHi1_So_surf+0.5*chem2.T*(chem2.aHi2_So_surf+2*chem2.T*(chem2.aHi3_So_surf+0.75*chem2.T*(chem2.aHi4_So_surf+0.8*chem2.T*chem2.aHi5_So_surf))/3)))+(chem2.T>chem2.Thi_So_surf)*(chem2.aHi6_So_surf+chem2.Thi_So_surf*(chem2.aHi1_So_surf+0.5*chem2.Thi_So_surf*(chem2.aHi2_So_surf+2*chem2.Thi_So_surf*(chem2.aHi3_So_surf+0.75*chem2.Thi_So_surf*(chem2.aHi4_So_surf+0.8*chem2.Thi_So_surf*chem2.aHi5_So_surf))/3))))			
chem2.s_So_surf	R_const*((chem2.T<=chem2.Tlo_So_surf)*(chem2.aLo7_So_surf+chem2.aLo1_So_surf*log(chem2.T/1[K])+chem2.Tlo_So_surf*(chem2.aLo2_So_surf+0.5*chem2.Tlo_So_surf*(chem2.aLo3_So_surf+2*chem2.Tlo_So_surf*(chem2.aLo4_So_surf+0.75*chem2.Tlo_So_surf*chem2.aLo5_So_surf)/3)))+(chem2.T>chem2.Tlo_So_surf)*(chem2.T<=chem2.Tmid_So_surf)*(chem2.aLo7_So_surf+chem2.aLo1_So_surf*log(chem2.T/1[K])+chem2.T*(chem2.aLo2_So_surf+0.5*chem2.T*(chem2.aLo3_So_surf+2*chem2.T*(chem2.aLo4_So_surf+0.75*chem2.T*chem2.aLo5_So_surf)/3)))+(chem2.T>chem2.Tmid_So_surf)*(chem2.T<=chem2.Thi_So_surf)*(chem2.aHi7_So_surf+chem2.aHi1_So_surf*log(chem2.T/1[K])+chem2.T*(chem2.aHi2_So_surf+0.5*chem2.T*(chem2.aHi3_So_surf+2*chem2.T*(chem2.aHi4_So_surf+0.75*chem2.T*chem2.aHi5_So_surf)/3)))+(chem2.T>chem2.Thi_So_surf)*(chem2.aHi7_So_surf+chem2.aHi1_So_surf*log(chem2.T/1[K])+chem2.Thi_So_surf*(chem2.aHi2_So_surf+	J/(mol·K)	Molar entropy	Global

Name	Expression	Unit	Description	Selection
	0.5*chem2.Thi_So_surf*(chem2.aHi3_So_surf+2*chem2.Thi_So_surf*(chem2.aHi4_So_surf+0.75*chem2.Thi_So_surf*chem2.aHi5_So_surf)/3))))			

2.10.8 Species: O2

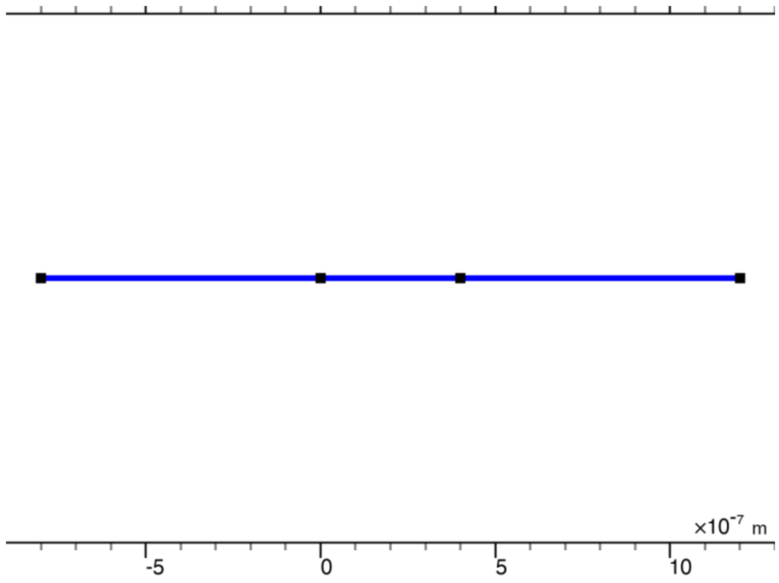

Species: O2

SELECTION

Geometric entity level	Domain
Selection	Geometry geom1: Dimension 1: All domains

SETTINGS

Description	Value
Species name	O2

Species Type

SETTINGS

Description	Value
Species type	Bulk species

General Parameters

SETTINGS

Description	Value
Molar mass	PM_O2

Description	Value
Charge	0

Reaction Rate

SETTINGS

Description	Value
	Automatic

Species Concentration/Activity

SETTINGS

Description	Value
Constant concentration/activity	Off

Additional Source

SETTINGS

Description	Value
Additional source	Off

Species Thermodynamic Expressions

SETTINGS

Description	Value
Species enthalpy	NASA format
Lower temperature limit	300[K]
Middle temperature limit	1000[K]
Upper temperature limit	5000[K]
	0
	0
	0
	0
	0
	0
	0
	0
	0
	0
	0
	0
	0

Description	Value
	0

Variables

Name	Expression	Unit	Description	Selection
chem2.M_O2	PM_O2	kg/mol	Molar mass	Global
chem2.z_O2	0	1	Charge	Global
chem2.Tlo_O2	300[K]	K	Lower temperature limit	Global
chem2.Tmid_O2	1000[K]	K	Middle temperature limit	Global
chem2.Thi_O2	5000[K]	K	Upper temperature limit	Global
chem2.aLo1_O2	0	1	Low polynomial coefficients	Global
chem2.aHi1_O2	0	1	High polynomial coefficients	Global
chem2.aLo2_O2	0	1/K	Low polynomial coefficients	Global
chem2.aHi2_O2	0	1/K	High polynomial coefficients	Global
chem2.aLo3_O2	0	$1/K^2$	Low polynomial coefficients	Global
chem2.aHi3_O2	0	$1/K^2$	High polynomial coefficients	Global
chem2.aLo4_O2	0	$1/K^3$	Low polynomial coefficients	Global
chem2.aHi4_O2	0	$1/K^3$	High polynomial coefficients	Global
chem2.aLo5_O2	0	$1/K^4$	Low polynomial coefficients	Global
chem2.aHi5_O2	0	$1/K^4$	High polynomial coefficients	Global
chem2.aLo6_O2	0	K	Low polynomial coefficients	Global
chem2.aHi6_O2	0	K	High polynomial coefficients	Global
chem2.aLo7_O2	0	1	Low polynomial coefficients	Global
chem2.aHi7_O2	0	1	High polynomial coefficients	Global

Name	Expression	Unit	Description	Selection
chem2.Cp_O2	R_const*((chem2.T<=chem2.Tlo_O2)*(chem2.aLo1_O2+chem2.Tlo_O2*(chem2.aLo2_O2+chem2.Tlo_O2*(chem2.aLo3_O2+chem2.Tlo_O2*(chem2.aLo4_O2+chem2.Tlo_O2*chem2.aLo5_O2))))+(chem2.T>chem2.Tlo_O2)*(chem2.T<=chem2.Tmid_O2)*(chem2.aLo1_O2+chem2.T*(chem2.aLo2_O2+chem2.T*(chem2.aLo3_O2+chem2.T*(chem2.aLo4_O2+chem2.T*chem2.aLo5_O2))))+(chem2.T>chem2.Tmid_O2)*(chem2.T<=chem2.Thi_O2)*(chem2.aHi1_O2+chem2.T*(chem2.aHi2_O2+chem2.T*(chem2.aHi3_O2+chem2.T*(chem2.aHi4_O2+chem2.T*chem2.aHi5_O2))))+(chem2.T>chem2.Thi_O2)*(chem2.aHi1_O2+chem2.Thi_O2*(chem2.aHi2_O2+chem2.Thi_O2*(chem2.aHi3_O2+chem2.Thi_O2*(chem2.aHi4_O2+chem2.Thi_O2*chem2.aHi5_O2)))))	J/(mol·K)	Heat capacity at constant pressure	Global
chem2.h_O2	R_const*((chem2.T<=chem2.Tlo_O2)*(chem2.aLo6_O2+chem2.Tlo_O2*(chem2.aLo1_O2+0.5*chem2.Tlo_O2*(chem2.aLo2_O2+2*chem2.Tlo_O2*(chem2.aLo3_O2+0.75*chem2.Tlo_O2*(chem2.aLo4_O2+0.8*chem2.Tlo_O2*chem2.aLo5_O2))/3)))+(chem2.T>chem2.Tlo_O2)*(chem2.T<=chem2.Tmid_O2)*(chem2.aLo6_O2+chem2.T*(chem2.aLo1_O2+0.5*chem2.T*(chem2.aLo2_O2+2*chem2.T*(chem2.aLo3_O2+0.75*chem2.T*(chem2.aLo4_O2+0.8*chem2.T*chem2.aLo5_O2))/3)))+(chem2.T>chem2.Tmid_O2)*(chem2.T<=chem2.Thi_O2)*(chem2.aHi6_O2+chem2.T*(chem2.aHi1_O2+0.5*chem2.T*(chem2.aHi2_O2+2*chem2.T*(chem2.aHi3_O2+0.75*chem2.T*(chem2.aHi4_O2+0.8*chem2.T*chem2.aHi5_O2))/3)))+(chem2.T>chem2.Thi_O2)*(chem2.aHi6_O2+chem2.Thi_O2*(chem2.aHi1_O2+0.5*chem2.Thi_O2*(chem2.aHi2_O2+2*chem2.Thi_O2*(chem2.aHi3_O2+0.75*chem2.Thi_O2*(chem2.aHi4_O2+0.8*chem2.Thi_O2*chem2.aHi5_O	J/mol	Molar enthalpy	Global

Name	Expression	Unit	Description	Selection
	2))/3))))			
chem2.s_O2	R_const*((chem2.T<=chem2.Tlo_O2)*(chem2.aLo7_O2+chem2.aLo1_O2*log(chem2.T/1[K])+chem2.Tlo_O2*(chem2.aLo2_O2+0.5*chem2.Tlo_O2*(chem2.aLo3_O2+2*chem2.Tlo_O2*(chem2.aLo4_O2+0.75*chem2.Tlo_O2*chem2.aLo5_O2)/3)))+(chem2.T>chem2.Tlo_O2)*(chem2.T<=chem2.Tmid_O2)*(chem2.aLo7_O2+chem2.aLo1_O2*log(chem2.T/1[K])+chem2.T*(chem2.aLo2_O2+0.5*chem2.T*(chem2.aLo3_O2+2*chem2.T*(chem2.aLo4_O2+0.75*chem2.T*chem2.aLo5_O2)/3)))+(chem2.T>chem2.Tmid_O2)*(chem2.T<=chem2.Thi_O2)*(chem2.aHi7_O2+chem2.aHi1_O2*log(chem2.T/1[K])+chem2.T*(chem2.aHi2_O2+0.5*chem2.T*(chem2.aHi3_O2+2*chem2.T*(chem2.aHi4_O2+0.75*chem2.T*chem2.aHi5_O2)/3)))+(chem2.T>chem2.Thi_O2)*(chem2.aHi7_O2+chem2.aHi1_O2*log(chem2.T/1[K])+chem2.Thi_O2*(chem2.aHi2_O2+0.5*chem2.Thi_O2*(chem2.aHi3_O2+2*chem2.Thi_O2*(chem2.aHi4_O2+0.75*chem2.Thi_O2*chem2.aHi5_O2)/3))))	J/(mol·K)	Molar entropy	Global

2.10.9 Species: Ascm

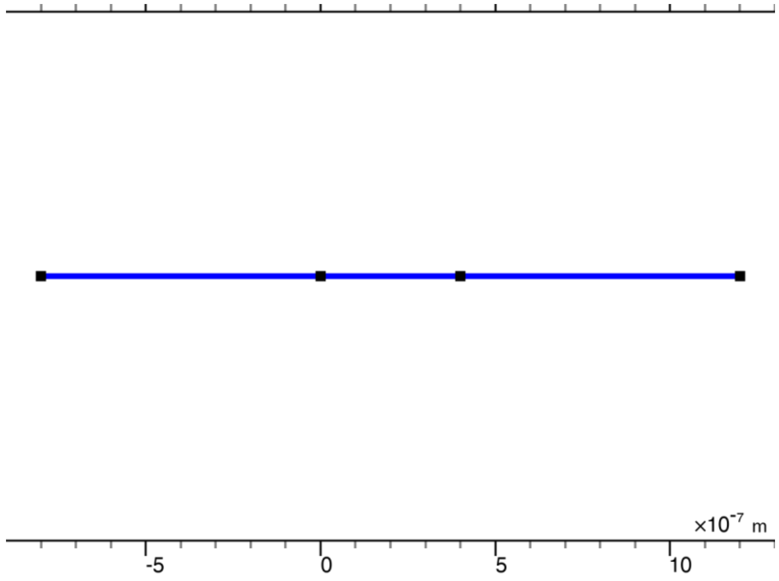

Species: Ascm

SELECTION

Geometric entity level	Domain
Selection	Geometry geom1: Dimension 1: All domains

SETTINGS

Description	Value
Species name	Ascm

Species Type

SETTINGS

Description	Value
Species type	Bulk species

General Parameters

SETTINGS

Description	Value
Molar mass	PM_Ascm
Charge	Z_Ascm

Reaction Rate

SETTINGS

Description	Value

Description	Value
	Automatic

Species Concentration/Activity

SETTINGS

Description	Value
Constant concentration/activity	Off

Additional Source

SETTINGS

Description	Value
Additional source	Off

Species Thermodynamic Expressions

SETTINGS

Description	Value
Species enthalpy	NASA format
Lower temperature limit	300[K]
Middle temperature limit	1000[K]
Upper temperature limit	5000[K]
	0
	0
	0
	0
	0
	0
	0
	0
	0
	0
	0
	0
	0
	0

Variables

Name	Expression	Unit	Description	Selection
chem2.M_Asc	PM_Ascm	kg/mol	Molar mass	Global

Name	Expression	Unit	Description	Selection
m				
chem2.z_Ascm	Z_Ascm	1	Charge	Global
chem2.Tlo_Ascm	300[K]	K	Lower temperature limit	Global
chem2.Tmid_Ascm	1000[K]	K	Middle temperature limit	Global
chem2.Thi_Ascm	5000[K]	K	Upper temperature limit	Global
chem2.aLo1_Ascm	0	1	Low polynomial coefficients	Global
chem2.aHi1_Ascm	0	1	High polynomial coefficients	Global
chem2.aLo2_Ascm	0	1/K	Low polynomial coefficients	Global
chem2.aHi2_Ascm	0	1/K	High polynomial coefficients	Global
chem2.aLo3_Ascm	0	$1/K^2$	Low polynomial coefficients	Global
chem2.aHi3_Ascm	0	$1/K^2$	High polynomial coefficients	Global
chem2.aLo4_Ascm	0	$1/K^3$	Low polynomial coefficients	Global
chem2.aHi4_Ascm	0	$1/K^3$	High polynomial coefficients	Global
chem2.aLo5_Ascm	0	$1/K^4$	Low polynomial coefficients	Global
chem2.aHi5_Ascm	0	$1/K^4$	High polynomial coefficients	Global
chem2.aLo6_Ascm	0	K	Low polynomial coefficients	Global
chem2.aHi6_Ascm	0	K	High polynomial coefficients	Global
chem2.aLo7_Ascm	0	1	Low polynomial coefficients	Global
chem2.aHi7_Ascm	0	1	High polynomial coefficients	Global
chem2.Cp_Ascm	R_const*((chem2.T<=chem2.Tlo_Ascm)*(chem2.aLo1_Ascm+chem2.Tlo_Ascm*(chem2.aLo2_Ascm+chem2.Tlo_Ascm*(chem2.aLo3_Ascm+chem2.Tlo_Ascm*(chem2.aLo4_Ascm+chem2.Tlo_Ascm*chem	J/(mol·K)	Heat capacity at constant pressure	Global

Name	Expression	Unit	Description	Selection
	2.aLo5_Ascm))))+(chem2.T>chem2.Tlo_Ascm)*(chem2.T<=chem2.Tmid_Ascm)*(chem2.aLo1_Ascm+chem2.T*(chem2.aLo2_Ascm+chem2.T*(chem2.aLo3_Ascm+chem2.T*(chem2.aLo4_Ascm+chem2.T*chem2.aLo5_Ascm))))+(chem2.T>chem2.Tmid_Ascm)*(chem2.T<=chem2.Thi_Ascm)*(chem2.aHi1_Ascm+chem2.T*(chem2.aHi2_Ascm+chem2.T*(chem2.aHi3_Ascm+chem2.T*(chem2.aHi4_Ascm+chem2.T*chem2.aHi5_Ascm))))+(chem2.T>chem2.Thi_Ascm)*(chem2.aHi1_Ascm+chem2.Thi_Ascm*(chem2.aHi2_Ascm+chem2.Thi_Ascm*(chem2.aHi3_Ascm+chem2.Thi_Ascm*(chem2.aHi4_Ascm+chem2.Thi_Ascm*chem2.aHi5_Ascm)))))			
chem2.h_Ascm	R_const*((chem2.T<=chem2.Tlo_Ascm)*(chem2.aLo6_Ascm+chem2.Tlo_Ascm*(chem2.aLo1_Ascm+0.5*chem2.Tlo_Ascm*(chem2.aLo2_Ascm+2*chem2.Tlo_Ascm*(chem2.aLo3_Ascm+0.75*chem2.Tlo_Ascm*(chem2.aLo4_Ascm+0.8*chem2.Tlo_Ascm*chem2.aLo5_Ascm))/3)))+(chem2.T>chem2.Tlo_Ascm)*(chem2.T<=chem2.Tmid_Ascm)*(chem2.aLo6_Ascm+chem2.T*(chem2.aLo1_Ascm+0.5*chem2.T*(chem2.aLo2_Ascm+2*chem2.T*(chem2.aLo3_Ascm+0.75*chem2.T*(chem2.aLo4_Ascm+0.8*chem2.T*chem2.aLo5_Ascm))/3)))+(chem2.T>chem2.Tmid_Ascm)*(chem2.T<=chem2.Thi_Ascm)*(chem2.aHi6_Ascm+chem2.T*(chem2.aHi1_Ascm+0.5*chem2.T*(chem2.aHi2_Ascm+2*chem2.T*(chem2.aHi3_Ascm+0.75*chem2.T*(chem2.aHi4_Ascm+0.8*chem2.T*chem2.aHi5_Ascm))/3)))+(chem2.T>chem2.Thi_Ascm)*(chem2.aHi6_Ascm+chem2.Thi_Ascm*(chem2.aHi1_Ascm+0.5*chem2.Thi_Ascm*(chem2.aHi2_Ascm+2*chem2.Thi_Ascm*(chem2.aHi3_Ascm+0.75*chem2.Thi_Ascm*(chem2.aHi4_Ascm+0.8*chem2.Thi_Ascm*chem2.aHi5_Ascm))/3))))	J/mol	Molar enthalpy	Global
chem2.s_Ascm	R_const*((chem2.T<=chem2.Tlo_Ascm)*(chem2.aLo7_Ascm+chem2.aLo1_Ascm*log(chem2.T/1[K])+chem2.Tlo_Ascm*(chem2.aLo2_Ascm+0.5*chem2.Tlo_Ascm*(chem2.aLo3_Ascm+2*chem2.Tlo_Ascm*(	J/(mol·K)	Molar entropy	Global

Name	Expression	Unit	Description	Selection
	chem2.aLo4_Ascm+0.75*chem2.Tlo_Ascm*chem2.aLo5_Ascm)/3)))+(chem2.T>chem2.Tlo_Ascm)*(chem2.T<=chem2.Tmid_Ascm)*(chem2.aLo7_Ascm+chem2.aLo1_Ascm*log(chem2.T/1[K])+chem2.T*(chem2.aLo2_Ascm+0.5*chem2.T*(chem2.aLo3_Ascm+2*chem2.T*(chem2.aLo4_Ascm+0.75*chem2.T*chem2.aLo5_Ascm)/3)))+(chem2.T>chem2.Tmid_Ascm)*(chem2.T<=chem2.Thi_Ascm)*(chem2.aHi7_Ascm+chem2.aHi1_Ascm*log(chem2.T/1[K])+chem2.T*(chem2.aHi2_Ascm+0.5*chem2.T*(chem2.aHi3_Ascm+2*chem2.T*(chem2.aHi4_Ascm+0.75*chem2.T*chem2.aHi5_Ascm)/3)))+(chem2.T>chem2.Thi_Ascm)*(chem2.aHi7_Ascm+chem2.aHi1_Ascm*log(chem2.T/1[K])+chem2.Thi_Ascm*(chem2.aHi2_Ascm+0.5*chem2.Thi_Ascm*(chem2.aHi3_Ascm+2*chem2.Thi_Ascm*(chem2.aHi4_Ascm+0.75*chem2.Thi_Ascm*chem2.aHi5_Ascm)/3))))			

2.10.10 Species: H2PO4

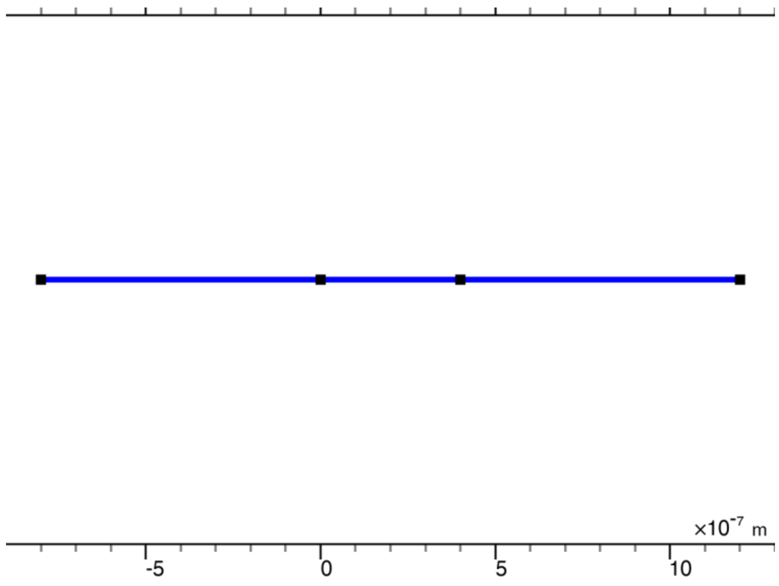

Species: H2PO4

SELECTION

Geometric entity level	Domain
Selection	Geometry geom1: Dimension 1: All domains

SETTINGS

Description	Value
Species name	H2PO4

Species Type

SETTINGS

Description	Value
Species type	Bulk species

General Parameters

SETTINGS

Description	Value
Molar mass	PM_H2PO4
Charge	-1

Reaction Rate

SETTINGS

Description	Value
	Automatic

Species Concentration/Activity

SETTINGS

Description	Value
Constant concentration/activity	Off

Additional Source

SETTINGS

Description	Value
Additional source	Off

Species Thermodynamic Expressions

SETTINGS

Description	Value
Species enthalpy	NASA format
Lower temperature limit	300[K]
Middle temperature limit	1000[K]
Upper temperature limit	5000[K]
	0

Description	Value
	0
	0
	0
	0
	0
	0
	0
	0
	0
	0
	0
	0
	0

Variables

Name	Expression	Unit	Description	Selection
chem2.M_H2PO4	PM_H2PO4	kg/mol	Molar mass	Global
chem2.z_H2PO4	-1	1	Charge	Global
chem2.Tlo_H2PO4	300[K]	K	Lower temperature limit	Global
chem2.Tmid_H2PO4	1000[K]	K	Middle temperature limit	Global
chem2.Thi_H2PO4	5000[K]	K	Upper temperature limit	Global
chem2.aLo1_H2PO4	0	1	Low polynomial coefficients	Global
chem2.aHi1_H2PO4	0	1	High polynomial coefficients	Global
chem2.aLo2_H2PO4	0	1/K	Low polynomial coefficients	Global
chem2.aHi2_H2PO4	0	1/K	High polynomial coefficients	Global
chem2.aLo3_H2PO4	0	$1/K^2$	Low polynomial coefficients	Global
chem2.aHi3_H2PO4	0	$1/K^2$	High polynomial coefficients	Global
chem2.aLo4_H	0	$1/K^3$	Low polynomial	Global

Name	Expression	Unit	Description	Selection
2PO4			coefficients	
chem2.aHi4_H2PO4	0	$1/K^3$	High polynomial coefficients	Global
chem2.aLo5_H2PO4	0	$1/K^4$	Low polynomial coefficients	Global
chem2.aHi5_H2PO4	0	$1/K^4$	High polynomial coefficients	Global
chem2.aLo6_H2PO4	0	K	Low polynomial coefficients	Global
chem2.aHi6_H2PO4	0	K	High polynomial coefficients	Global
chem2.aLo7_H2PO4	0	1	Low polynomial coefficients	Global
chem2.aHi7_H2PO4	0	1	High polynomial coefficients	Global
chem2.Cp_H2PO4	R_const*((chem2.T<=chem2.Tlo_H2PO4)*(chem2.aLo1_H2PO4+chem2.Tlo_H2PO4*(chem2.aLo2_H2PO4+chem2.Tlo_H2PO4*(chem2.aLo3_H2PO4+chem2.Tlo_H2PO4*(chem2.aLo4_H2PO4+chem2.Tlo_H2PO4*chem2.aLo5_H2PO4))))+(chem2.T>chem2.Tlo_H2PO4)*(chem2.T<=chem2.Tmid_H2PO4)*(chem2.aLo1_H2PO4+chem2.T*(chem2.aLo2_H2PO4+chem2.T*(chem2.aLo3_H2PO4+chem2.T*(chem2.aLo4_H2PO4+chem2.T*chem2.aLo5_H2PO4))))+(chem2.T>chem2.Tmid_H2PO4)*(chem2.T<=chem2.Thi_H2PO4)*(chem2.aHi1_H2PO4+chem2.T*(chem2.aHi2_H2PO4+chem2.T*(chem2.aHi3_H2PO4+chem2.T*(chem2.aHi4_H2PO4+chem2.T*chem2.aHi5_H2PO4))))+(chem2.T>chem2.Thi_H2PO4)*(chem2.aHi1_H2PO4+chem2.Thi_H2PO4*(chem2.aHi2_H2PO4+chem2.Thi_H2PO4*(chem2.aHi3_H2PO4+chem2.Thi_H2PO4*(chem2.aHi4_H2PO4+chem2.Thi_H2PO4*chem2.aHi5_H2PO4)))))	J/(mol·K)	Heat capacity at constant pressure	Global
chem2.h_H2PO4	R_const*((chem2.T<=chem2.Tlo_H2PO4)*(chem2.aLo6_H2PO4+chem2.Tlo_H2PO4*(chem2.aLo1_H2PO4+0.5*chem2.Tlo_H2PO4*(chem2.aLo2_H2PO4+2*chem2.Tlo_H2PO4*(chem2.aLo3_H2PO4+0.75*chem2.Tlo_H2PO4*(chem2.aLo4_H2PO4+0.8*chem2.Tlo_H2PO4*chem2.aLo5_H2P	J/mol	Molar enthalpy	Global

Name	Expression	Unit	Description	Selection
	O4))/3)))+(chem2.T>chem2.Tlo_H2PO4)*(chem2.T<=chem2.Tmid_H2PO4)*(chem2.aLo6_H2PO4+chem2.T*(chem2.aLo1_H2PO4+0.5*chem2.T*(chem2.aLo2_H2PO4+2*chem2.T*(chem2.aLo3_H2PO4+0.75*chem2.T*(chem2.aLo4_H2PO4+0.8*chem2.T*chem2.aLo5_H2PO4))/3)))+(chem2.T>chem2.Tmid_H2PO4)*(chem2.T<=chem2.Thi_H2PO4)*(chem2.aHi6_H2PO4+chem2.T*(chem2.aHi1_H2PO4+0.5*chem2.T*(chem2.aHi2_H2PO4+2*chem2.T*(chem2.aHi3_H2PO4+0.75*chem2.T*(chem2.aHi4_H2PO4+0.8*chem2.T*chem2.aHi5_H2PO4))/3)))+(chem2.T>chem2.Thi_H2PO4)*(chem2.aHi6_H2PO4+chem2.Thi_H2PO4*(chem2.aHi1_H2PO4+0.5*chem2.Thi_H2PO4*(chem2.aHi2_H2PO4+2*chem2.Thi_H2PO4*(chem2.aHi3_H2PO4+0.75*chem2.Thi_H2PO4*(chem2.aHi4_H2PO4+0.8*chem2.Thi_H2PO4*chem2.aHi5_H2PO4))/3))))			
chem2.s_H2PO4	R_const*((chem2.T<=chem2.Tlo_H2PO4)*(chem2.aLo7_H2PO4+chem2.aLo1_H2PO4*log(chem2.T/1[K])+chem2.Tlo_H2PO4*(chem2.aLo2_H2PO4+0.5*chem2.Tlo_H2PO4*(chem2.aLo3_H2PO4+2*chem2.Tlo_H2PO4*(chem2.aLo4_H2PO4+0.75*chem2.Tlo_H2PO4*chem2.aLo5_H2PO4)/3)))+(chem2.T>chem2.Tlo_H2PO4)*(chem2.T<=chem2.Tmid_H2PO4)*(chem2.aLo7_H2PO4+chem2.aLo1_H2PO4*log(chem2.T/1[K])+chem2.T*(chem2.aLo2_H2PO4+0.5*chem2.T*(chem2.aLo3_H2PO4+2*chem2.T*(chem2.aLo4_H2PO4+0.75*chem2.T*chem2.aLo5_H2PO4)/3)))+(chem2.T>chem2.Tmid_H2PO4)*(chem2.T<=chem2.Thi_H2PO4)*(chem2.aHi7_H2PO4+chem2.aHi1_H2PO4*log(chem2.T/1[K])+chem2.T*(chem2.aHi2_H2PO4+0.5*chem2.T*(chem2.aHi3_H2PO4+2*chem2.T*(chem2.aHi4_H2PO4+0.75*chem2.T*chem2.aHi5_H2PO4)/3)))+(chem2.T>chem2.Thi_H2PO4)*(chem2.aHi7_H2PO4+chem2.aHi1_H2PO4*log(chem2.T/1[K])+chem2.Thi_H2PO4*(chem2.aHi2_H2PO4+0.5*chem2.Thi_H2PO4*(chem2.aHi3_H2PO4+2*chem2.Thi_H2PO4*(chem2.aHi4_H2P	J/(mol·K)	Molar entropy	Global

Name	Expression	Unit	Description	Selection
	O4+0.75*chem2.Thi_H2PO4*chem2.aHi5_H2PO4)/3))))			

2.11 CHEMISTRY EQUILIBRIA

USED PRODUCTS

COMSOL Multiphysics
Chemical Reaction Engineering Module

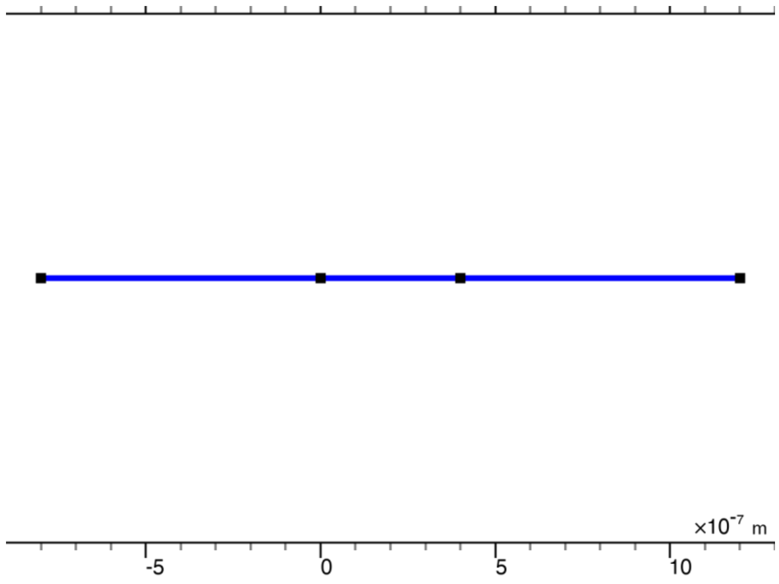

Chemistry equilibria

SELECTION

Geometric entity level	Domain
Selection	Geometry geom1: Dimension 1: All domains

EQUATIONS

$$R_i = \sum_j R_{ij}$$

2.11.1 Interface Settings

Discretization

SETTINGS

Description	Value
Element order	Quadratic

SETTINGS

Description	Value
Equation form	Study controlled

Model Input

SETTINGS

Description	Value
Temperature	Common model input
Pressure	Common model input

Mixture Properties

SETTINGS

Description	Value
Type	Diluted species
Phase	Liquid

Species Matching

SETTINGS

Description	Value
Species solved for	User defined

Species	Type	Molar concentration	Value	Reaction rate	Unit	Equation formulation
CO2	Variable	UserDefined	cCO2	chem3.R_CO2	mol/m^3	cCO2
CO3	Variable	UserDefined	cCO3	chem3.R_CO3	mol/m^3	cCO3
H	Variable	UserDefined	cH	chem3.R_H	mol/m^3	cH
H2O	Solvent	UserDefined	cH2O	solvent	mol/m^3	cH2O
H2PO4	Variable	UserDefined	cH2PO4	chem3.R_H2PO4	mol/m^3	cH2PO4
H3PO4	Variable	UserDefined	cH3PO4	chem3.R_H3PO4	mol/m^3	cH3PO4
HCO3	Variable	UserDefined	cHCO3	chem3.R_HCO3	mol/m^3	cHCO3
HPO4	Variable	UserDefined	cHPO4	chem3.R_HPO4	mol/m^3	cHPO4
OH	Variable	UserDefined	cOH	chem3.R_OH	mol/m^3	cOH
PO4	Variable	UserDefined	cPO4	chem3.R_PO4	mol/m^3	cPO4

Calculate Transport Properties

SETTINGS

Description	Value
Calculate mixture properties	Off

Activity

SETTINGS

Description	Value
Use activity	Off

CHEMKIN Import for Species Properties

SETTINGS

Description	Value
Thermo input file	
Transport input file	

Pellet Chemistry

SETTINGS

Description	Value
Define variables for porous pellets	Off

2.11.2 Variables

Name	Expression	Unit	Description	Selection	Details
chem3.T	model.input.T	K	Temperature	Domains 1–3	Meta
chem3.p	model.input.p	Pa	Pressure	Domains 1–3	Meta
chem3.eeqMean	chem3.eeqSum/chem3.nEr	V	Mean equilibrium potential	Global	
chem3.csum	eps+chem3.c_CO2+chem3.c_CO3+chem3.c_H+chem3.c_H2O+chem3.c_H2PO4+chem3.c_H3PO4+chem3.c_HCO3+chem3.c_HPO4+chem3.c_OH+chem3.c_PO4	mol/m³	Total concentration	Global	
chem3.m_CO2	max(chem3.c_CO2,0)/chem3.csum	1	Molar fraction	Global	
chem3.m_CO3	max(chem3.c_CO3,0)/chem3.csum	1	Molar fraction	Global	
chem3.m_H	max(chem3.c_H,0)/ch	1	Molar fraction	Global	

Name	Expression	Unit	Description	Selection	Details
	em3.csum				
chem3.m_H2O	max(chem3.c_H2O,0)/chem3.csum	1	Molar fraction	Global	
chem3.m_H2PO4	max(chem3.c_H2PO4,0)/chem3.csum	1	Molar fraction	Global	
chem3.m_H3PO4	max(chem3.c_H3PO4,0)/chem3.csum	1	Molar fraction	Global	
chem3.m_HCO3	max(chem3.c_HCO3,0)/chem3.csum	1	Molar fraction	Global	
chem3.m_HPO4	max(chem3.c_HPO4,0)/chem3.csum	1	Molar fraction	Global	
chem3.m_OH	max(chem3.c_OH,0)/chem3.csum	1	Molar fraction	Global	
chem3.m_PO4	max(chem3.c_PO4,0)/chem3.csum	1	Molar fraction	Global	
chem3.rhosum	eps+chem3.c_CO2*chem3.M_CO2+chem3.c_CO3*chem3.M_CO3+chem3.c_H*chem3.M_H+chem3.c_H2O*chem3.M_H2O+chem3.c_H2PO4*chem3.M_H2PO4+chem3.c_H3PO4*chem3.M_H3PO4+chem3.c_HCO3*chem3.M_HCO3+chem3.c_HPO4*chem3.M_HPO4+chem3.c_OH*chem3.M_OH+chem3.c_PO4*chem3.M_PO4	kg/m^3	Density	Global	
chem3.w_CO2	chem3.c_CO2*chem3.M_CO2/chem3.rhosum	1	Mass fraction	Global	
chem3.w_CO3	chem3.c_CO3*chem3.M_CO3/chem3.rhosum	1	Mass fraction	Global	
chem3.w_H	chem3.c_H*chem3.M_H/chem3.rhosum	1	Mass fraction	Global	
chem3.w_H2O	chem3.c_H2O*chem3.M_H2O/chem3.rhosum	1	Mass fraction	Global	
chem3.w_H2PO4	chem3.c_H2PO4*che	1	Mass fraction	Global	

Name	Expression	Unit	Description	Selection	Details
	m3.M_H2PO4/chem3.rhosum				
chem3.w_H3PO4	chem3.c_H3PO4*chem3.M_H3PO4/chem3.rhosum	1	Mass fraction	Global	
chem3.w_HCO3	chem3.c_HCO3*chem3.M_HCO3/chem3.rhosum	1	Mass fraction	Global	
chem3.w_HPO4	chem3.c_HPO4*chem3.M_HPO4/chem3.rhosum	1	Mass fraction	Global	
chem3.w_OH	chem3.c_OH*chem3.M_OH/chem3.rhosum	1	Mass fraction	Global	
chem3.w_PO4	chem3.c_PO4*chem3.M_PO4/chem3.rhosum	1	Mass fraction	Global	
chem3.c_CO2	cCO2	mol/m^3	Concentration	Global	
chem3.Rsum_CO2	0	$mol/(m^3 \cdot s)$	Reaction rate for species CO2	Global	+ operation
chem3.R_CO2	0	$mol/(m^3 \cdot s)$	Reaction rate for species CO2	Global	+ operation
chem3.Rsurf_CO2	0	$mol/(m^2 \cdot s)$	Surface reaction rate for species CO2	Global	+ operation
chem3.Rsurfsum_CO2	0	$mol/(m^2 \cdot s)$	Surface reaction rate for species CO2	Global	+ operation
chem3.c_CO3	cCO3	mol/m^3	Concentration	Global	
chem3.Rsum_CO3	0	$mol/(m^3 \cdot s)$	Reaction rate for species CO3	Global	+ operation
chem3.R_CO3	0	$mol/(m^3 \cdot s)$	Reaction rate for species CO3	Global	+ operation
chem3.Rsurf_CO3	0	$mol/(m^2 \cdot s)$	Surface reaction rate for species CO3	Global	+ operation
chem3.Rsurfsum_CO3	0	$mol/(m^2 \cdot s)$	Surface reaction rate for species CO3	Global	+ operation
chem3.c_H	cH	mol/m^3	Concentration	Global	
chem3.Rsum_H	0	$mol/(m^3 \cdot s)$	Reaction rate for species H	Global	+ operation

Name	Expression	Unit	Description	Selection	Details
chem3.R_H	0	mol/(m³·s)	Reaction rate for species H	Global	+ operation
chem3.Rsurf_H	0	mol/(m²·s)	Surface reaction rate for species H	Global	+ operation
chem3.Rsurfsum_H	0	mol/(m²·s)	Surface reaction rate for species H	Global	+ operation
chem3.c_H2O	cH2O	mol/m³	Concentration	Global	
chem3.Rsum_H2O	0	mol/(m³·s)	Reaction rate for species H2O	Global	+ operation
chem3.c_H2PO4	cH2PO4	mol/m³	Concentration	Global	
chem3.Rsum_H2PO4	0	mol/(m³·s)	Reaction rate for species H2PO4	Global	+ operation
chem3.R_H2PO4	0	mol/(m³·s)	Reaction rate for species H2PO4	Global	+ operation
chem3.Rsurf_H2PO4	0	mol/(m²·s)	Surface reaction rate for species H2PO4	Global	+ operation
chem3.Rsurfsum_H2PO4	0	mol/(m²·s)	Surface reaction rate for species H2PO4	Global	+ operation
chem3.c_H3PO4	cH3PO4	mol/m³	Concentration	Global	
chem3.Rsum_H3PO4	0	mol/(m³·s)	Reaction rate for species H3PO4	Global	+ operation
chem3.R_H3PO4	0	mol/(m³·s)	Reaction rate for species H3PO4	Global	+ operation
chem3.Rsurf_H3PO4	0	mol/(m²·s)	Surface reaction rate for species H3PO4	Global	+ operation
chem3.Rsurfsum_H3PO4	0	mol/(m²·s)	Surface reaction rate for species H3PO4	Global	+ operation
chem3.c_HCO3	cHCO3	mol/m³	Concentration	Global	
chem3.Rsum_HCO3	0	mol/(m³·s)	Reaction rate for species HCO3	Global	+ operation
chem3.R_HCO3	0	mol/(m³·s)	Reaction rate for species HCO3	Global	+ operation
chem3.Rsurf_HCO3	0	mol/(m²·s)	Surface reaction rate for species HCO3	Global	+ operation
chem3.Rsurfsum_HCO3	0	mol/(m²·s)	Surface reaction rate for species	Global	+ operation

Name	Expression	Unit	Description	Selection	Details
			HCO3		
chem3.c_HPO4	cHPO4	mol/m³	Concentration	Global	
chem3.Rsum_HPO4	0	mol/(m³·s)	Reaction rate for species HPO4	Global	+ operation
chem3.R_HPO4	0	mol/(m³·s)	Reaction rate for species HPO4	Global	+ operation
chem3.Rsurf_HPO4	0	mol/(m²·s)	Surface reaction rate for species HPO4	Global	+ operation
chem3.Rsurfsum_HPO4	0	mol/(m²·s)	Surface reaction rate for species HPO4	Global	+ operation
chem3.c_OH	cOH	mol/m³	Concentration	Global	
chem3.Rsum_OH	0	mol/(m³·s)	Reaction rate for species OH	Global	+ operation
chem3.R_OH	0	mol/(m³·s)	Reaction rate for species OH	Global	+ operation
chem3.Rsurf_OH	0	mol/(m²·s)	Surface reaction rate for species OH	Global	+ operation
chem3.Rsurfsum_OH	0	mol/(m²·s)	Surface reaction rate for species OH	Global	+ operation
chem3.c_PO4	cPO4	mol/m³	Concentration	Global	
chem3.Rsum_PO4	0	mol/(m³·s)	Reaction rate for species PO4	Global	+ operation
chem3.R_PO4	0	mol/(m³·s)	Reaction rate for species PO4	Global	+ operation
chem3.Rsurf_PO4	0	mol/(m²·s)	Surface reaction rate for species PO4	Global	+ operation
chem3.Rsurfsum_PO4	0	mol/(m²·s)	Surface reaction rate for species PO4	Global	+ operation
chem3.Qheat	0	W/m³	Heat source of reactions	Global	+ operation
chem3.Qtot	chem3.Qheat	W/m³	Heat source of reactions	Global	
chem3.Mn	chem3.M_H2O	kg/mol	Mean molar mass	Global	

2.11.3 1: H3PO4<=>H2PO4+H

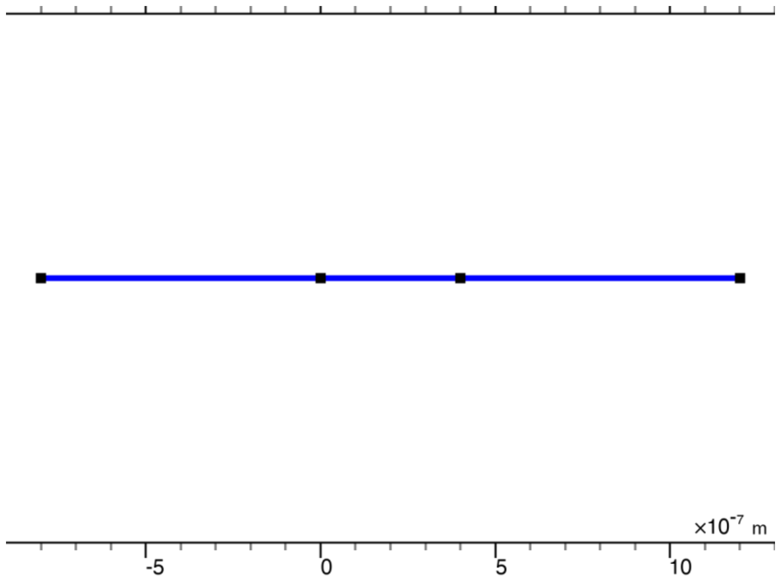

1: H3PO4<=>H2PO4+H

SELECTION

Geometric entity level	Domain
Selection	Geometry geom1: Dimension 1: All domains

Reaction Formula

SETTINGS

Description	Value
Formula	H3PO4<=>H2PO4 + H
Reaction type	Reversible

Reaction Rate

SETTINGS

Description	Value
Reaction rate	Mass action law
Description	Overall forward reaction order: 1 Overall reverse reaction order: 2

Rate Constants

SETTINGS

Description	Value
Specify equilibrium constant	On
Use Arrhenius expressions	Off
Forward rate constant	1E5

Equilibrium Settings

SETTINGS

Description	Value
Equilibrium constant	User defined
Equilibrium constant	K_H3PO4

Reaction Thermodynamic Properties

SETTINGS

Description	Value
Enthalpy of reaction	Automatic
Entropy of reaction	Automatic
Heat source of reaction	Automatic

Variables

Name	Expression	Unit	Description	Selection	Details
chem3.Rsum_H	chem3.r_1	mol/(m³·s)	Reaction rate for species H	Global	+ operation
chem3.R_H	chem3.r_1	mol/(m³·s)	Reaction rate for species H	Global	+ operation
chem3.Rsum_H2PO4	chem3.r_1	mol/(m³·s)	Reaction rate for species H2PO4	Global	+ operation
chem3.R_H2PO4	chem3.r_1	mol/(m³·s)	Reaction rate for species H2PO4	Global	+ operation
chem3.Rsum_H3PO4	-chem3.r_1	mol/(m³·s)	Reaction rate for species H3PO4	Global	+ operation
chem3.R_H3PO4	-chem3.r_1	mol/(m³·s)	Reaction rate for species H3PO4	Global	+ operation
chem3.Qheat	-chem3.r_1*chem3.H_1	W/m³	Heat source of reactions	Global	+ operation
chem3.kf_1	100000	1/s	Forward rate constant	Global	
chem3.Keq0_1	K_H3PO4		Equilibrium constant	Global	
chem3.kr_1	chem3.kf_1*1[m^3/(s*mol)]/(chem3.Keq0_1*1[1/s])	m³/(s·mol)	Reverse rate constant	Global	
chem3.r_1	chem3.kf_1*chem3.c_H3PO4-	mol/(m³·s)	Reaction rate	Global	

Name	Expression	Unit	Description	Selection	Details
	chem3.kr_1*chem3.c_H2PO4*chem3.c_H				
chem3.H_1	-chem3.h_H3PO4+chem3.h_H2PO4+chem3.h_H	J/mol	Enthalpy of reaction	Global	
chem3.S_1	-chem3.s_H3PO4+chem3.s_H2PO4+chem3.s_H	J/(mol·K)	Entropy of reaction	Global	
chem3.Q_1	-chem3.r_1*chem3.H_1	W/m³	Heat source of reaction	Global	

2.11.4 Species: H3PO4

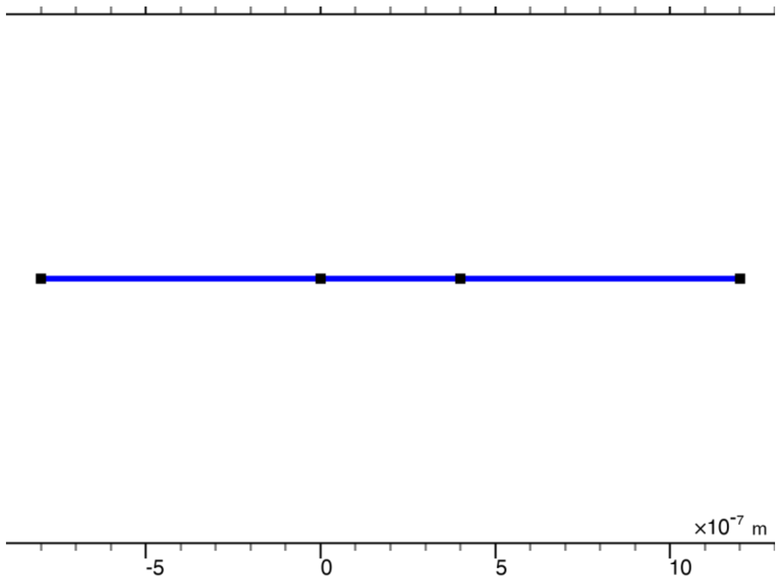

Species: H3PO4

SELECTION

Geometric entity level	Domain
Selection	Geometry geom1: Dimension 1: All domains

SETTINGS

Description	Value
Species name	H3PO4

Species Type

SETTINGS

Description	Value
Species type	Bulk species

General Parameters

SETTINGS

Description	Value
Molar mass	PM_H3PO4
Charge	0

Reaction Rate

SETTINGS

Description	Value
	Automatic

Species Concentration/Activity

SETTINGS

Description	Value
Constant concentration/activity	Off

Additional Source

SETTINGS

Description	Value
Additional source	Off

Species Thermodynamic Expressions

SETTINGS

Description	Value
Species enthalpy	NASA format
Lower temperature limit	300[K]
Middle temperature limit	1000[K]
Upper temperature limit	5000[K]
	0
	0
	0
	0
	0
	0
	0
	0
	0
	0

Description	Value
	0
	0
	0
	0

Variables

Name	Expression	Unit	Description	Selection
chem3.M_H3PO4	PM_H3PO4	kg/mol	Molar mass	Global
chem3.z_H3PO4	0	1	Charge	Global
chem3.Tlo_H3PO4	300[K]	K	Lower temperature limit	Global
chem3.Tmid_H3PO4	1000[K]	K	Middle temperature limit	Global
chem3.Thi_H3PO4	5000[K]	K	Upper temperature limit	Global
chem3.aLo1_H3PO4	0	1	Low polynomial coefficients	Global
chem3.aHi1_H3PO4	0	1	High polynomial coefficients	Global
chem3.aLo2_H3PO4	0	1/K	Low polynomial coefficients	Global
chem3.aHi2_H3PO4	0	1/K	High polynomial coefficients	Global
chem3.aLo3_H3PO4	0	$1/K^2$	Low polynomial coefficients	Global
chem3.aHi3_H3PO4	0	$1/K^2$	High polynomial coefficients	Global
chem3.aLo4_H3PO4	0	$1/K^3$	Low polynomial coefficients	Global
chem3.aHi4_H3PO4	0	$1/K^3$	High polynomial coefficients	Global
chem3.aLo5_H3PO4	0	$1/K^4$	Low polynomial coefficients	Global
chem3.aHi5_H3PO4	0	$1/K^4$	High polynomial coefficients	Global
chem3.aLo6_H3PO4	0	K	Low polynomial coefficients	Global
chem3.aHi6_H	0	K	High polynomial	Global

Name	Expression	Unit	Description	Selection
3PO4			coefficients	
chem3.aLo7_H3PO4	0	1	Low polynomial coefficients	Global
chem3.aHi7_H3PO4	0	1	High polynomial coefficients	Global
chem3.Cp_H3PO4	R_const*((chem3.T<=chem3.Tlo_H3PO4)*(chem3.aLo1_H3PO4+chem3.Tlo_H3PO4*(chem3.aLo2_H3PO4+chem3.Tlo_H3PO4*(chem3.aLo3_H3PO4+chem3.Tlo_H3PO4*(chem3.aLo4_H3PO4+chem3.Tlo_H3PO4*chem3.aLo5_H3PO4))))+(chem3.T>chem3.Tlo_H3PO4)*(chem3.T<=chem3.Tmid_H3PO4)*(chem3.aLo1_H3PO4+chem3.T*(chem3.aLo2_H3PO4+chem3.T*(chem3.aLo3_H3PO4+chem3.T*(chem3.aLo4_H3PO4+chem3.T*chem3.aLo5_H3PO4))))+(chem3.T>chem3.Tmid_H3PO4)*(chem3.T<=chem3.Thi_H3PO4)*(chem3.aHi1_H3PO4+chem3.T*(chem3.aHi2_H3PO4+chem3.T*(chem3.aHi3_H3PO4+chem3.T*(chem3.aHi4_H3PO4+chem3.T*chem3.aHi5_H3PO4))))+(chem3.T>chem3.Thi_H3PO4)*(chem3.aHi1_H3PO4+chem3.Thi_H3PO4*(chem3.aHi2_H3PO4+chem3.Thi_H3PO4*(chem3.aHi3_H3PO4+chem3.Thi_H3PO4*(chem3.aHi4_H3PO4+chem3.Thi_H3PO4*chem3.aHi5_H3PO4)))))	J/(mol·K)	Heat capacity at constant pressure	Global
chem3.h_H3PO4	R_const*((chem3.T<=chem3.Tlo_H3PO4)*(chem3.aLo6_H3PO4+chem3.Tlo_H3PO4*(chem3.aLo1_H3PO4+0.5*chem3.Tlo_H3PO4*(chem3.aLo2_H3PO4+2*chem3.Tlo_H3PO4*(chem3.aLo3_H3PO4+0.75*chem3.Tlo_H3PO4*(chem3.aLo4_H3PO4+0.8*chem3.Tlo_H3PO4*chem3.aLo5_H3PO4))/3)))+(chem3.T>chem3.Tlo_H3PO4)*(chem3.T<=chem3.Tmid_H3PO4)*(chem3.aLo6_H3PO4+chem3.T*(chem3.aLo1_H3PO4+0.5*chem3.T*(chem3.aLo2_H3PO4+2*chem3.T*(chem3.aLo3_H3PO4+0.75*chem3.T*(chem3.aLo4_H3PO4+0.8*chem3.T*chem3.aLo5_H3PO4))/3)))+(chem3.T>chem3.Tmid_H3PO4)*(chem3.T<=chem3.Thi_H3PO4)*(chem3.aHi6_H3PO4+chem3.T*(chem3.aHi1_H3PO4+0.5*chem3.T*(chem3.aHi2_H3PO4+2*chem3	J/mol	Molar enthalpy	Global

Name	Expression	Unit	Description	Selection
	.T*(chem3.aHi3_H3PO4+0.75*chem3.T*(chem3.aHi4_H3PO4+0.8*chem3.T*chem3.aHi5_H3PO4))/3)))+(chem3.T>chem3.Thi_H3PO4)*(chem3.aHi6_H3PO4+chem3.Thi_H3PO4*(chem3.aHi1_H3PO4+0.5*chem3.Thi_H3PO4*(chem3.aHi2_H3PO4+2*chem3.Thi_H3PO4*(chem3.aHi3_H3PO4+0.75*chem3.Thi_H3PO4*(chem3.aHi4_H3PO4+0.8*chem3.Thi_H3PO4*chem3.aHi5_H3PO4))/3))))			
chem3.s_H3PO4	R_const*((chem3.T<=chem3.Tlo_H3PO4)*(chem3.aLo7_H3PO4+chem3.aLo1_H3PO4*log(chem3.T/1[K])+chem3.Tlo_H3PO4*(chem3.aLo2_H3PO4+0.5*chem3.Tlo_H3PO4*(chem3.aLo3_H3PO4+2*chem3.Tlo_H3PO4*(chem3.aLo4_H3PO4+0.75*chem3.Tlo_H3PO4*chem3.aLo5_H3PO4)/3)))+(chem3.T>chem3.Tlo_H3PO4)*(chem3.T<=chem3.Tmid_H3PO4)*(chem3.aLo7_H3PO4+chem3.aLo1_H3PO4*log(chem3.T/1[K])+chem3.T*(chem3.aLo2_H3PO4+0.5*chem3.T*(chem3.aLo3_H3PO4+2*chem3.T*(chem3.aLo4_H3PO4+0.75*chem3.T*chem3.aLo5_H3PO4)/3)))+(chem3.T>chem3.Tmid_H3PO4)*(chem3.T<=chem3.Thi_H3PO4)*(chem3.aHi7_H3PO4+chem3.aHi1_H3PO4*log(chem3.T/1[K])+chem3.T*(chem3.aHi2_H3PO4+0.5*chem3.T*(chem3.aHi3_H3PO4+2*chem3.T*(chem3.aHi4_H3PO4+0.75*chem3.T*chem3.aHi5_H3PO4)/3)))+(chem3.T>chem3.Thi_H3PO4)*(chem3.aHi7_H3PO4+chem3.aHi1_H3PO4*log(chem3.T/1[K])+chem3.Thi_H3PO4*(chem3.aHi2_H3PO4+0.5*chem3.Thi_H3PO4*(chem3.aHi3_H3PO4+2*chem3.Thi_H3PO4*(chem3.aHi4_H3PO4+0.75*chem3.Thi_H3PO4*chem3.aHi5_H3PO4)/3))))	J/(mol·K)	Molar entropy	Global

2.11.5 Species: H2PO4

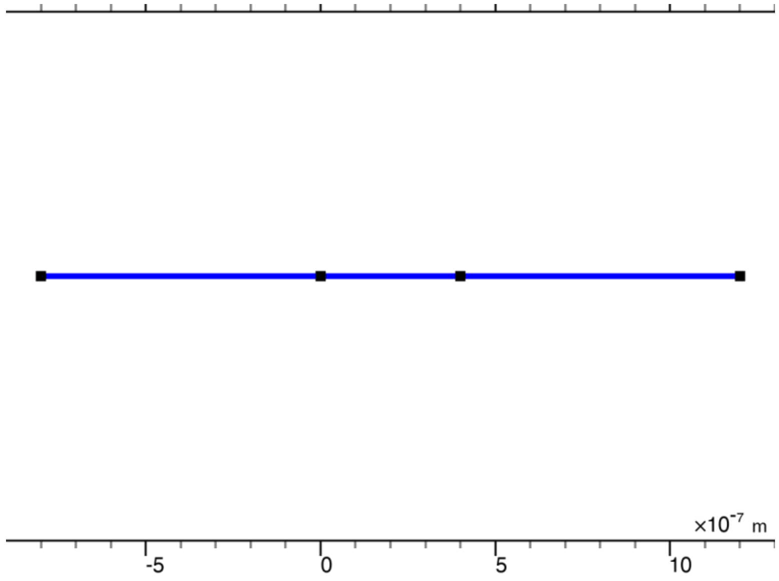

Species: H2PO4

SELECTION

Geometric entity level	Domain
Selection	Geometry geom1: Dimension 1: All domains

SETTINGS

Description	Value
Species name	H2PO4

Species Type

SETTINGS

Description	Value
Species type	Bulk species

General Parameters

SETTINGS

Description	Value
Molar mass	PM_H2PO4
Charge	-1

Reaction Rate

SETTINGS

Description	Value

Description	Value
	Automatic

Species Concentration/Activity

SETTINGS

Description	Value
Constant concentration/activity	Off

Additional Source

SETTINGS

Description	Value
Additional source	Off

Species Thermodynamic Expressions

SETTINGS

Description	Value
Species enthalpy	NASA format
Lower temperature limit	300[K]
Middle temperature limit	1000[K]
Upper temperature limit	5000[K]
	0
	0
	0
	0
	0
	0
	0
	0
	0
	0
	0
	0
	0
	0

Variables

Name	Expression	Unit	Description	Selection
chem3.M_H2P	PM_H2PO4	kg/mol	Molar mass	Global

Name	Expression	Unit	Description	Selection
O4				
chem3.z_H2PO4	-1	1	Charge	Global
chem3.Tlo_H2PO4	300[K]	K	Lower temperature limit	Global
chem3.Tmid_H2PO4	1000[K]	K	Middle temperature limit	Global
chem3.Thi_H2PO4	5000[K]	K	Upper temperature limit	Global
chem3.aLo1_H2PO4	0	1	Low polynomial coefficients	Global
chem3.aHi1_H2PO4	0	1	High polynomial coefficients	Global
chem3.aLo2_H2PO4	0	1/K	Low polynomial coefficients	Global
chem3.aHi2_H2PO4	0	1/K	High polynomial coefficients	Global
chem3.aLo3_H2PO4	0	$1/K^2$	Low polynomial coefficients	Global
chem3.aHi3_H2PO4	0	$1/K^2$	High polynomial coefficients	Global
chem3.aLo4_H2PO4	0	$1/K^3$	Low polynomial coefficients	Global
chem3.aHi4_H2PO4	0	$1/K^3$	High polynomial coefficients	Global
chem3.aLo5_H2PO4	0	$1/K^4$	Low polynomial coefficients	Global
chem3.aHi5_H2PO4	0	$1/K^4$	High polynomial coefficients	Global
chem3.aLo6_H2PO4	0	K	Low polynomial coefficients	Global
chem3.aHi6_H2PO4	0	K	High polynomial coefficients	Global
chem3.aLo7_H2PO4	0	1	Low polynomial coefficients	Global
chem3.aHi7_H2PO4	0	1	High polynomial coefficients	Global
chem3.Cp_H2PO4	R_const*((chem3.T<=chem3.Tlo_H2PO4)*(chem3.aLo1_H2PO4+chem3.Tlo_H2PO4*(chem3.aLo2_H2PO4+chem3.Tlo_H2PO4*(chem3.aLo3_H2PO4+chem3.Tlo_H2	J/(mol·K)	Heat capacity at constant pressure	Global

Name	Expression	Unit	Description	Selection
	PO4*(chem3.aLo4_H2PO4+chem3.Tlo_H2PO4*chem3.aLo5_H2PO4))))+(chem3.T>chem3.Tlo_H2PO4)*(chem3.T<=chem3.Tmid_H2PO4)*(chem3.aLo1_H2PO4+chem3.T*(chem3.aLo2_H2PO4+chem3.T*(chem3.aLo3_H2PO4+chem3.T*(chem3.aLo4_H2PO4+chem3.T*chem3.aLo5_H2PO4))))+(chem3.T>chem3.Tmid_H2PO4)*(chem3.T<=chem3.Thi_H2PO4)*(chem3.aHi1_H2PO4+chem3.T*(chem3.aHi2_H2PO4+chem3.T*(chem3.aHi3_H2PO4+chem3.T*(chem3.aHi4_H2PO4+chem3.T*chem3.aHi5_H2PO4))))+(chem3.T>chem3.Thi_H2PO4)*(chem3.aHi1_H2PO4+chem3.Thi_H2PO4*(chem3.aHi2_H2PO4+chem3.Thi_H2PO4*(chem3.aHi3_H2PO4+chem3.Thi_H2PO4*(chem3.aHi4_H2PO4+chem3.Thi_H2PO4*chem3.aHi5_H2PO4)))))			
chem3.h_H2PO4	R_const*((chem3.T<=chem3.Tlo_H2PO4)*(chem3.aLo6_H2PO4+chem3.Tlo_H2PO4*(chem3.aLo1_H2PO4+0.5*chem3.Tlo_H2PO4*(chem3.aLo2_H2PO4+2*chem3.Tlo_H2PO4*(chem3.aLo3_H2PO4+0.75*chem3.Tlo_H2PO4*(chem3.aLo4_H2PO4+0.8*chem3.Tlo_H2PO4*chem3.aLo5_H2PO4))/3)))+(chem3.T>chem3.Tlo_H2PO4)*(chem3.T<=chem3.Tmid_H2PO4)*(chem3.aLo6_H2PO4+chem3.T*(chem3.aLo1_H2PO4+0.5*chem3.T*(chem3.aLo2_H2PO4+2*chem3.T*(chem3.aLo3_H2PO4+0.75*chem3.T*(chem3.aLo4_H2PO4+0.8*chem3.T*chem3.aLo5_H2PO4))/3)))+(chem3.T>chem3.Tmid_H2PO4)*(chem3.T<=chem3.Thi_H2PO4)*(chem3.aHi6_H2PO4+chem3.T*(chem3.aHi1_H2PO4+0.5*chem3.T*(chem3.aHi2_H2PO4+2*chem3.T*(chem3.aHi3_H2PO4+0.75*chem3.T*(chem3.aHi4_H2PO4+0.8*chem3.T*chem3.aHi5_H2PO4))/3)))+(chem3.T>chem3.Thi_H2PO4)*(chem3.aHi6_H2PO4+chem3.Thi_H2PO4*(chem3.aHi1_H2PO4+0.5*chem3.Thi_H2PO4*(chem3.aHi2_H2PO4+2*chem3.Thi_H2PO4*(chem3.aHi3_H2PO4+0.75*chem3.Thi_H2PO4*(chem3.aHi4_H2PO4+0.8*chem3.Thi_H2PO4*chem3.aHi5_H2PO4))/3))))	J/mol	Molar enthalpy	Global

Name	Expression	Unit	Description	Selection
chem3.s_H2PO4	R_const*((chem3.T<=chem3.Tlo_H2PO4)*(chem3.aLo7_H2PO4+chem3.aLo1_H2PO4*log(chem3.T/1[K])+chem3.Tlo_H2PO4*(chem3.aLo2_H2PO4+0.5*chem3.Tlo_H2PO4*(chem3.aLo3_H2PO4+2*chem3.Tlo_H2PO4*(chem3.aLo4_H2PO4+0.75*chem3.Tlo_H2PO4*chem3.aLo5_H2PO4)/3)))+(chem3.T>chem3.Tlo_H2PO4)*(chem3.T<=chem3.Tmid_H2PO4)*(chem3.aLo7_H2PO4+chem3.aLo1_H2PO4*log(chem3.T/1[K])+chem3.T*(chem3.aLo2_H2PO4+0.5*chem3.T*(chem3.aLo3_H2PO4+2*chem3.T*(chem3.aLo4_H2PO4+0.75*chem3.T*chem3.aLo5_H2PO4)/3)))+(chem3.T>chem3.Tmid_H2PO4)*(chem3.T<=chem3.Thi_H2PO4)*(chem3.aHi7_H2PO4+chem3.aHi1_H2PO4*log(chem3.T/1[K])+chem3.T*(chem3.aHi2_H2PO4+0.5*chem3.T*(chem3.aHi3_H2PO4+2*chem3.T*(chem3.aHi4_H2PO4+0.75*chem3.T*chem3.aHi5_H2PO4)/3)))+(chem3.T>chem3.Thi_H2PO4)*(chem3.aHi7_H2PO4+chem3.aHi1_H2PO4*log(chem3.T/1[K])+chem3.Thi_H2PO4*(chem3.aHi2_H2PO4+0.5*chem3.Thi_H2PO4*(chem3.aHi3_H2PO4+2*chem3.Thi_H2PO4*(chem3.aHi4_H2PO4+0.75*chem3.Thi_H2PO4*chem3.aHi5_H2PO4)/3))))	J/(mol·K)	Molar entropy	Global

2.11.6 Species: H

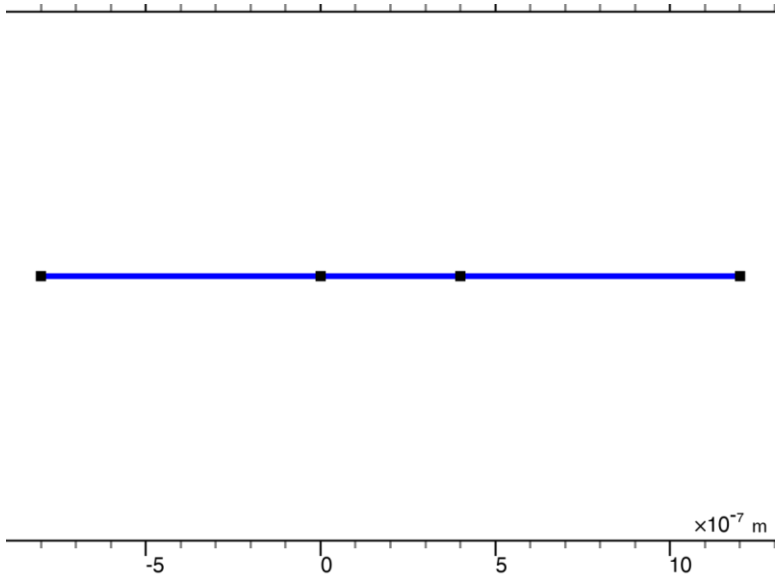

Species: H

SELECTION

Geometric entity level	Domain
Selection	Geometry geom1: Dimension 1: All domains

SETTINGS

Description	Value
Species name	H

Species Type

SETTINGS

Description	Value
Species type	Bulk species

General Parameters

SETTINGS

Description	Value
Molar mass	PM_H
Charge	+1

Reaction Rate

SETTINGS

Description	Value

Description	Value
	Automatic

Species Concentration/Activity

SETTINGS

Description	Value
Constant concentration/activity	Off

Additional Source

SETTINGS

Description	Value
Additional source	Off

Species Thermodynamic Expressions

SETTINGS

Description	Value
Species enthalpy	NASA format
Lower temperature limit	300[K]
Middle temperature limit	1000[K]
Upper temperature limit	5000[K]
	0
	0
	0
	0
	0
	0
	0
	0
	0
	0
	0
	0
	0
	0

Variables

Name	Expression	Unit	Description	Selection
chem3.M_H	PM_H	kg/mol	Molar mass	Global

Name	Expression	Unit	Description	Selection
chem3.z_H	1	1	Charge	Global
chem3.Tlo_H	300[K]	K	Lower temperature limit	Global
chem3.Tmid_H	1000[K]	K	Middle temperature limit	Global
chem3.Thi_H	5000[K]	K	Upper temperature limit	Global
chem3.aLo1_H	0	1	Low polynomial coefficients	Global
chem3.aHi1_H	0	1	High polynomial coefficients	Global
chem3.aLo2_H	0	1/K	Low polynomial coefficients	Global
chem3.aHi2_H	0	1/K	High polynomial coefficients	Global
chem3.aLo3_H	0	$1/K^2$	Low polynomial coefficients	Global
chem3.aHi3_H	0	$1/K^2$	High polynomial coefficients	Global
chem3.aLo4_H	0	$1/K^3$	Low polynomial coefficients	Global
chem3.aHi4_H	0	$1/K^3$	High polynomial coefficients	Global
chem3.aLo5_H	0	$1/K^4$	Low polynomial coefficients	Global
chem3.aHi5_H	0	$1/K^4$	High polynomial coefficients	Global
chem3.aLo6_H	0	K	Low polynomial coefficients	Global
chem3.aHi6_H	0	K	High polynomial coefficients	Global
chem3.aLo7_H	0	1	Low polynomial coefficients	Global
chem3.aHi7_H	0	1	High polynomial coefficients	Global
chem3.Cp_H	R_const*((chem3.T<=chem3.Tlo_H)*(chem3.aLo1_H+chem3.Tlo_H*(chem3.aLo2_H+chem3.Tlo_H*(chem3.aLo3_H+chem3.Tlo_H*(chem3.aLo4_H+chem3.Tlo_H*chem3.aLo5_H))))+(chem3.T>chem3.Tlo_H)*(chem3.T<=chem3.	J/(mol·K)	Heat capacity at constant pressure	Global

Name	Expression	Unit	Description	Selection
	Tmid_H)*(chem3.aLo1_H+chem3.T*(chem3.aLo2_H+chem3.T*(chem3.aLo3_H+chem3.T*(chem3.aLo4_H+chem3.T*chem3.aLo5_H))))+(chem3.T>chem3.Tmid_H)*(chem3.T<=chem3.Thi_H)*(chem3.aHi1_H+chem3.T*(chem3.aHi2_H+chem3.T*(chem3.aHi3_H+chem3.T*(chem3.aHi4_H+chem3.T*chem3.aHi5_H))))+(chem3.T>chem3.Thi_H)*(chem3.aHi1_H+chem3.Thi_H*(chem3.aHi2_H+chem3.Thi_H*(chem3.aHi3_H+chem3.Thi_H*(chem3.aHi4_H+chem3.Thi_H*chem3.aHi5_H)))))			
chem3.h_H	R_const*((chem3.T<=chem3.Tlo_H)*(chem3.aLo6_H+chem3.Tlo_H*(chem3.aLo1_H+0.5*chem3.Tlo_H*(chem3.aLo2_H+2*chem3.Tlo_H*(chem3.aLo3_H+0.75*chem3.Tlo_H*(chem3.aLo4_H+0.8*chem3.Tlo_H*chem3.aLo5_H))/3)))+(chem3.T>chem3.Tlo_H)*(chem3.T<=chem3.Tmid_H)*(chem3.aLo6_H+chem3.T*(chem3.aLo1_H+0.5*chem3.T*(chem3.aLo2_H+2*chem3.T*(chem3.aLo3_H+0.75*chem3.T*(chem3.aLo4_H+0.8*chem3.T*chem3.aLo5_H))/3)))+(chem3.T>chem3.Tmid_H)*(chem3.T<=chem3.Thi_H)*(chem3.aHi6_H+chem3.T*(chem3.aHi1_H+0.5*chem3.T*(chem3.aHi2_H+2*chem3.T*(chem3.aHi3_H+0.75*chem3.T*(chem3.aHi4_H+0.8*chem3.T*chem3.aHi5_H))/3)))+(chem3.T>chem3.Thi_H)*(chem3.aHi6_H+chem3.Thi_H*(chem3.aHi1_H+0.5*chem3.Thi_H*(chem3.aHi2_H+2*chem3.Thi_H*(chem3.aHi3_H+0.75*chem3.Thi_H*(chem3.aHi4_H+0.8*chem3.Thi_H*chem3.aHi5_H))/3))))	J/mol	Molar enthalpy	Global
chem3.s_H	R_const*((chem3.T<=chem3.Tlo_H)*(chem3.aLo7_H+chem3.aLo1_H*log(chem3.T/1[K])+chem3.Tlo_H*(chem3.aLo2_H+0.5*chem3.Tlo_H*(chem3.aLo3_H+2*chem3.Tlo_H*(chem3.aLo4_H+0.75*chem3.Tlo_H*chem3.aLo5_H)/3)))+(chem3.T>chem3.Tlo_H)*(chem3.T<=chem3.Tmid_H)*(chem3.aLo7_H+chem3.aLo1_H*log(chem3.T/1[K])+chem3.T*(chem3.aLo2_H+0.5*chem3.T	J/(mol·K)	Molar entropy	Global

Name	Expression	Unit	Description	Selection
	*(chem3.aLo3_H+2*chem3.T*(chem3.aLo4_H+0.75*chem3.T*chem3.aLo5_H)/3)))+(chem3.T>chem3.Tmid_H)*(chem3.T<=chem3.Thi_H)*(chem3.aHi7_H+chem3.aHi1_H*log(chem3.T/1[K])+chem3.T*(chem3.aHi2_H+0.5*chem3.T*(chem3.aHi3_H+2*chem3.T*(chem3.aHi4_H+0.75*chem3.T*chem3.aHi5_H)/3)))+(chem3.T>chem3.Thi_H)*(chem3.aHi7_H+chem3.aHi1_H*log(chem3.T/1[K])+chem3.Thi_H*(chem3.aHi2_H+0.5*chem3.Thi_H*(chem3.aHi3_H+2*chem3.Thi_H*(chem3.aHi4_H+0.75*chem3.Thi_H*chem3.aHi5_H)/3))))			

2.11.7 2: H2PO4<=>HPO4+H

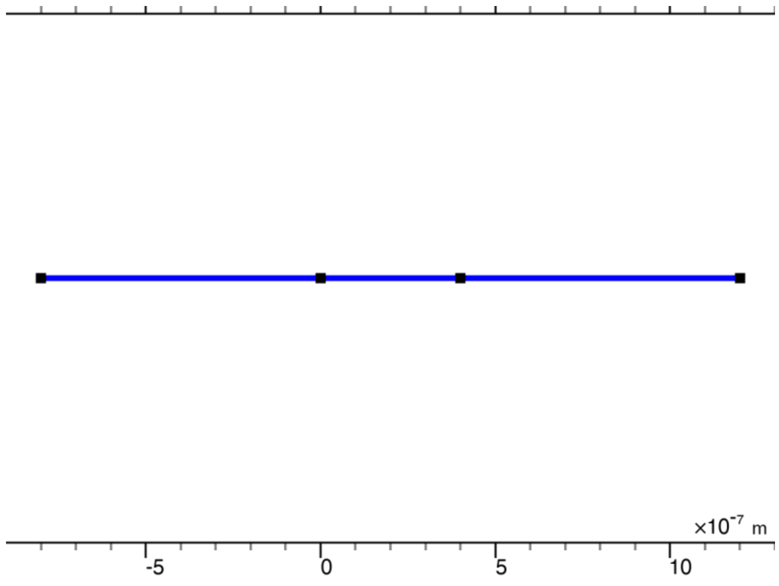

2: H2PO4<=>HPO4+H

SELECTION

Geometric entity level	Domain
Selection	Geometry geom1: Dimension 1: All domains

Reaction Formula

SETTINGS

Description	Value
Formula	H2PO4<=>HPO4 + H
Reaction type	Reversible

Reaction Rate

SETTINGS

Description	Value
Reaction rate	Mass action law
Description	Overall forward reaction order: 1 Overall reverse reaction order: 2

Rate Constants

SETTINGS

Description	Value
Specify equilibrium constant	On
Use Arrhenius expressions	Off
Forward rate constant	1e5

Equilibrium Settings

SETTINGS

Description	Value
Equilibrium constant	User defined
Equilibrium constant	K_H2PO4

Reaction Thermodynamic Properties

SETTINGS

Description	Value
Enthalpy of reaction	Automatic
Entropy of reaction	Automatic
Heat source of reaction	Automatic

Variables

Name	Expression	Unit	Description	Selection	Details
chem3.Rsum_H	chem3.r_2	mol/(m^3·s)	Reaction rate for species H	Global	+ operation
chem3.R_H	chem3.r_2	mol/(m^3·s)	Reaction rate for species H	Global	+ operation
chem3.Rsum_H2PO4	-chem3.r_2	mol/(m^3·s)	Reaction rate for species H2PO4	Global	+ operation
chem3.R_H2PO4	-chem3.r_2	mol/(m^3·s)	Reaction rate for species H2PO4	Global	+ operation
chem3.Rsum_HP	chem3.r_2	mol/(m^3·s)	Reaction rate for species	Global	+ operation

Name	Expression	Unit	Description	Selection	Details
O4			HPO4		
chem3.R_HPO4	chem3.r_2	mol/(m³·s)	Reaction rate for species HPO4	Global	+ operation
chem3.Qheat	-chem3.r_2*chem3.H_2	W/m³	Heat source of reactions	Global	+ operation
chem3.kf_2	100000	1/s	Forward rate constant	Global	
chem3.Keq0_2	K_H2PO4		Equilibrium constant	Global	
chem3.kr_2	chem3.kf_2*1[m^3/(s*mol)]/(chem3.Keq0_2*1[1/s])	m³/(s·mol)	Reverse rate constant	Global	
chem3.r_2	chem3.kf_2*chem3.c_H2PO4-chem3.kr_2*chem3.c_HPO4*chem3.c_H	mol/(m³·s)	Reaction rate	Global	
chem3.H_2	-chem3.h_H2PO4+chem3.h_HPO4+chem3.h_H	J/mol	Enthalpy of reaction	Global	
chem3.S_2	-chem3.s_H2PO4+chem3.s_HPO4+chem3.s_H	J/(mol·K)	Entropy of reaction	Global	
chem3.Q_2	-chem3.r_2*chem3.H_2	W/m³	Heat source of reaction	Global	

2.11.8 Species: HPO4

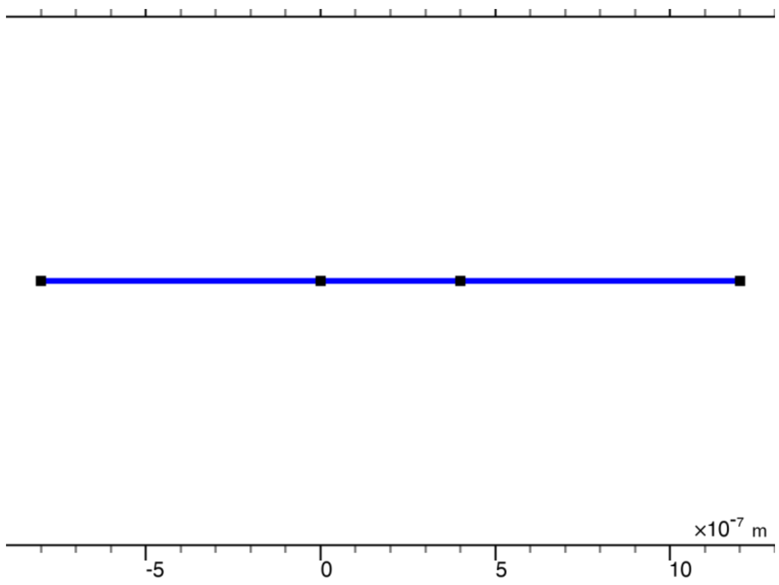

Species: HPO4

SELECTION

Geometric entity level	Domain
Selection	Geometry geom1: Dimension 1: All domains

SETTINGS

Description	Value
Species name	HPO4

Species Type

SETTINGS

Description	Value
Species type	Bulk species

General Parameters

SETTINGS

Description	Value
Molar mass	PM_HPO4
Charge	-2

Reaction Rate

SETTINGS

Description	Value
	Automatic

Species Concentration/Activity

SETTINGS

Description	Value
Constant concentration/activity	Off

Additional Source

SETTINGS

Description	Value
Additional source	Off

Species Thermodynamic Expressions

SETTINGS

Description	Value
Species enthalpy	NASA format
Lower temperature limit	300[K]

Description	Value
Middle temperature limit	1000[K]
Upper temperature limit	5000[K]
	0
	0
	0
	0
	0
	0
	0
	0
	0
	0
	0
	0
	0
	0

Variables

Name	Expression	Unit	Description	Selection
chem3.M_HPO4	PM_HPO4	kg/mol	Molar mass	Global
chem3.z_HPO4	-2	1	Charge	Global
chem3.Tlo_HPO4	300[K]	K	Lower temperature limit	Global
chem3.Tmid_HPO4	1000[K]	K	Middle temperature limit	Global
chem3.Thi_HPO4	5000[K]	K	Upper temperature limit	Global
chem3.aLo1_HPO4	0	1	Low polynomial coefficients	Global
chem3.aHi1_HPO4	0	1	High polynomial coefficients	Global
chem3.aLo2_HPO4	0	1/K	Low polynomial coefficients	Global
chem3.aHi2_HPO4	0	1/K	High polynomial coefficients	Global
chem3.aLo3_HPO4	0	$1/K^2$	Low polynomial coefficients	Global

Name	Expression	Unit	Description	Selection
chem3.aHi3_HPO4	0	1/K²	High polynomial coefficients	Global
chem3.aLo4_HPO4	0	1/K³	Low polynomial coefficients	Global
chem3.aHi4_HPO4	0	1/K³	High polynomial coefficients	Global
chem3.aLo5_HPO4	0	1/K⁴	Low polynomial coefficients	Global
chem3.aHi5_HPO4	0	1/K⁴	High polynomial coefficients	Global
chem3.aLo6_HPO4	0	K	Low polynomial coefficients	Global
chem3.aHi6_HPO4	0	K	High polynomial coefficients	Global
chem3.aLo7_HPO4	0	1	Low polynomial coefficients	Global
chem3.aHi7_HPO4	0	1	High polynomial coefficients	Global
chem3.Cp_HPO4	R_const*((chem3.T<=chem3.Tlo_HPO4)*(chem3.aLo1_HPO4+chem3.Tlo_HPO4*(chem3.aLo2_HPO4+chem3.Tlo_HPO4*(chem3.aLo3_HPO4+chem3.Tlo_HPO4*(chem3.aLo4_HPO4+chem3.Tlo_HPO4*chem3.aLo5_HPO4))))+(chem3.T>chem3.Tlo_HPO4)*(chem3.T<=chem3.Tmid_HPO4)*(chem3.aLo1_HPO4+chem3.T*(chem3.aLo2_HPO4+chem3.T*(chem3.aLo3_HPO4+chem3.T*(chem3.aLo4_HPO4+chem3.T*chem3.aLo5_HPO4))))+(chem3.T>chem3.Tmid_HPO4)*(chem3.T<=chem3.Thi_HPO4)*(chem3.aHi1_HPO4+chem3.T*(chem3.aHi2_HPO4+chem3.T*(chem3.aHi3_HPO4+chem3.T*(chem3.aHi4_HPO4+chem3.T*chem3.aHi5_HPO4))))+(chem3.T>chem3.Thi_HPO4)*(chem3.aHi1_HPO4+chem3.Thi_HPO4*(chem3.aHi2_HPO4+chem3.Thi_HPO4*(chem3.aHi3_HPO4+chem3.Thi_HPO4*(chem3.aHi4_HPO4+chem3.Thi_HPO4*chem3.aHi5_HPO4)))))	J/(mol·K)	Heat capacity at constant pressure	Global
chem3.h_HPO4	R_const*((chem3.T<=chem3.Tlo_HPO4)*(chem3.aLo6_HPO4+chem3.Tlo_HPO4*(chem3.aLo1_HPO4+0.5*chem3.Tlo_HPO4*(chem3.aLo2_HPO4+2*chem3.Tlo_HPO4*(chem3.aLo3_HPO4+0.75*chem3.Tlo	J/mol	Molar enthalpy	Global

Name	Expression	Unit	Description	Selection
	_HPO4*(chem3.aLo4_HPO4+0.8*chem3.Tlo_HPO4*chem3.aLo5_HPO4))/3)))+(chem3.T>chem3.Tlo_HPO4)*(chem3.T<=chem3.Tmid_HPO4)*(chem3.aLo6_HPO4+chem3.T*(chem3.aLo1_HPO4+0.5*chem3.T*(chem3.aLo2_HPO4+2*chem3.T*(chem3.aLo3_HPO4+0.75*chem3.T*(chem3.aLo4_HPO4+0.8*chem3.T*chem3.aLo5_HPO4))/3)))+(chem3.T>chem3.Tmid_HPO4)*(chem3.T<=chem3.Thi_HPO4)*(chem3.aHi6_HPO4+chem3.T*(chem3.aHi1_HPO4+0.5*chem3.T*(chem3.aHi2_HPO4+2*chem3.T*(chem3.aHi3_HPO4+0.75*chem3.T*(chem3.aHi4_HPO4+0.8*chem3.T*chem3.aHi5_HPO4))/3)))+(chem3.T>chem3.Thi_HPO4)*(chem3.aHi6_HPO4+chem3.Thi_HPO4*(chem3.aHi1_HPO4+0.5*chem3.Thi_HPO4*(chem3.aHi2_HPO4+2*chem3.Thi_HPO4*(chem3.aHi3_HPO4+0.75*chem3.Thi_HPO4*(chem3.aHi4_HPO4+0.8*chem3.Thi_HPO4*chem3.aHi5_HPO4))/3))))			
chem3.s_HPO4	R_const*((chem3.T<=chem3.Tlo_HPO4)*(chem3.aLo7_HPO4+chem3.aLo1_HPO4*log(chem3.T/1[K])+chem3.Tlo_HPO4*(chem3.aLo2_HPO4+0.5*chem3.Tlo_HPO4*(chem3.aLo3_HPO4+2*chem3.Tlo_HPO4*(chem3.aLo4_HPO4+0.75*chem3.Tlo_HPO4*chem3.aLo5_HPO4)/3)))+(chem3.T>chem3.Tlo_HPO4)*(chem3.T<=chem3.Tmid_HPO4)*(chem3.aLo7_HPO4+chem3.aLo1_HPO4*log(chem3.T/1[K])+chem3.T*(chem3.aLo2_HPO4+0.5*chem3.T*(chem3.aLo3_HPO4+2*chem3.T*(chem3.aLo4_HPO4+0.75*chem3.T*chem3.aLo5_HPO4)/3)))+(chem3.T>chem3.Tmid_HPO4)*(chem3.T<=chem3.Thi_HPO4)*(chem3.aHi7_HPO4+chem3.aHi1_HPO4*log(chem3.T/1[K])+chem3.T*(chem3.aHi2_HPO4+0.5*chem3.T*(chem3.aHi3_HPO4+2*chem3.T*(chem3.aHi4_HPO4+0.75*chem3.T*chem3.aHi5_HPO4)/3)))+(chem3.T>chem3.Thi_HPO4)*(chem3.aHi7_HPO4+chem3.aHi1_HPO4*log(chem3.T/1[K])+chem3.Thi_HPO4*(chem3.aHi2_HPO4+0.5*chem3.Thi_HPO4*(chem3.aHi3_HPO4+2*chem3.Thi_HPO4*(chem3.aHi4_HPO4+0.	J/(mol·K)	Molar entropy	Global

Name	Expression	Unit	Description	Selection
	75*chem3.Thi_HPO4*chem3.aHi5_HPO4)/3))))			

2.11.9 3: HPO4<=>PO4+H

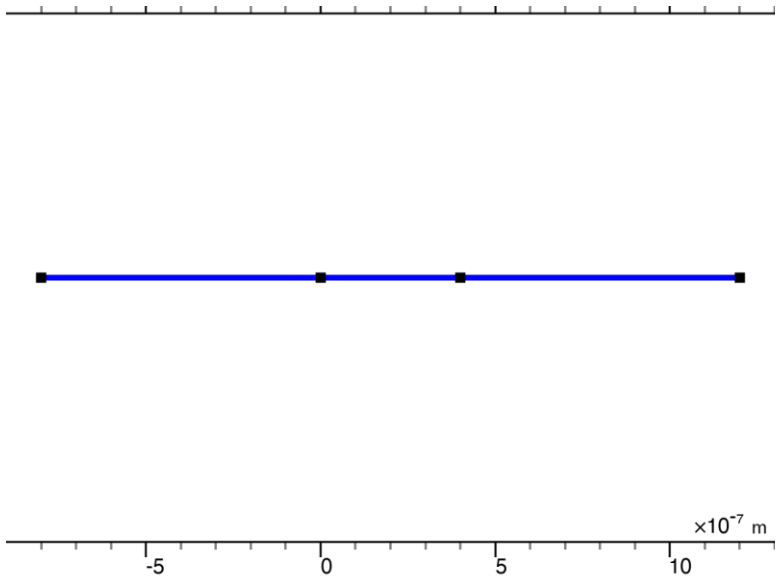

3: HPO4<=>PO4+H

SELECTION

Geometric entity level	Domain
Selection	Geometry geom1: Dimension 1: All domains

Reaction Formula

SETTINGS

Description	Value
Formula	HPO4<=>PO4 + H
Reaction type	Reversible

Reaction Rate

SETTINGS

Description	Value
Reaction rate	Mass action law
Description	Overall forward reaction order: 1 Overall reverse reaction order: 2

Rate Constants

SETTINGS

Description	Value

Description	Value
Specify equilibrium constant	On
Use Arrhenius expressions	Off
Forward rate constant	1E5

Equilibrium Settings

SETTINGS

Description	Value
Equilibrium constant	User defined
Equilibrium constant	K_HPO4

Reaction Thermodynamic Properties

SETTINGS

Description	Value
Enthalpy of reaction	Automatic
Entropy of reaction	Automatic
Heat source of reaction	Automatic

Variables

Name	Expression	Unit	Description	Selection	Details
chem3.Rsum_H	chem3.r_3	mol/(m³·s)	Reaction rate for species H	Global	+ operation
chem3.R_H	chem3.r_3	mol/(m³·s)	Reaction rate for species H	Global	+ operation
chem3.Rsum_HPO4	-chem3.r_3	mol/(m³·s)	Reaction rate for species HPO4	Global	+ operation
chem3.R_HPO4	-chem3.r_3	mol/(m³·s)	Reaction rate for species HPO4	Global	+ operation
chem3.Rsum_PO4	chem3.r_3	mol/(m³·s)	Reaction rate for species PO4	Global	+ operation
chem3.R_PO4	chem3.r_3	mol/(m³·s)	Reaction rate for species PO4	Global	+ operation
chem3.Qheat	-chem3.r_3*chem3.H_3	W/m³	Heat source of reactions	Global	+ operation
chem3.kf_3	100000	1/s	Forward rate constant	Global	
chem3.Keq0_3	K_HPO4		Equilibrium constant	Global	

Name	Expression	Unit	Description	Selection	Details
chem3.kr_3	chem3.kf_3*1[m^3/(s*mol)]/(chem3.Keq0_3*1[1/s])	$m^3/(s\cdot mol)$	Reverse rate constant	Global	
chem3.r_3	chem3.kf_3*chem3.c_HPO4-chem3.kr_3*chem3.c_PO4*chem3.c_H	$mol/(m^3\cdot s)$	Reaction rate	Global	
chem3.H_3	-chem3.h_HPO4+chem3.h_PO4+chem3.h_H	J/mol	Enthalpy of reaction	Global	
chem3.S_3	-chem3.s_HPO4+chem3.s_PO4+chem3.s_H	J/(mol·K)	Entropy of reaction	Global	
chem3.Q_3	-chem3.r_3*chem3.H_3	W/m^3	Heat source of reaction	Global	

2.11.10 Species: PO4

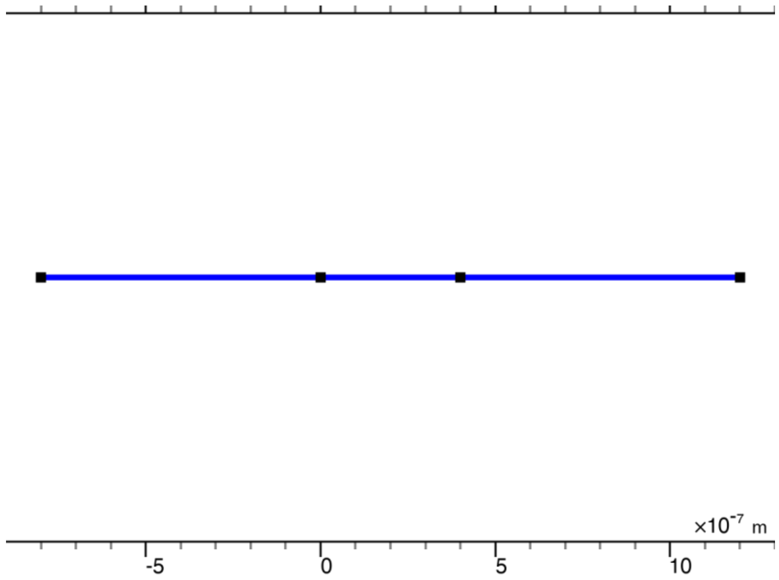

Species: PO4

SELECTION

Geometric entity level	Domain
Selection	Geometry geom1: Dimension 1: All domains

SETTINGS

Description	Value
Species name	PO4

Species Type

SETTINGS

Description	Value
Species type	Bulk species

General Parameters

SETTINGS

Description	Value
Molar mass	PM_PO4
Charge	-3

Reaction Rate

SETTINGS

Description	Value
	Automatic

Species Concentration/Activity

SETTINGS

Description	Value
Constant concentration/activity	Off

Additional Source

SETTINGS

Description	Value
Additional source	Off

Species Thermodynamic Expressions

SETTINGS

Description	Value
Species enthalpy	NASA format
Lower temperature limit	300[K]
Middle temperature limit	1000[K]
Upper temperature limit	5000[K]
	0
	0
	0
	0
	0

Description	Value
	0
	0
	0
	0
	0
	0
	0
	0
	0

Variables

Name	Expression	Unit	Description	Selection
chem3.M_PO4	PM_PO4	kg/mol	Molar mass	Global
chem3.z_PO4	-3	1	Charge	Global
chem3.Tlo_PO4	300[K]	K	Lower temperature limit	Global
chem3.Tmid_PO4	1000[K]	K	Middle temperature limit	Global
chem3.Thi_PO4	5000[K]	K	Upper temperature limit	Global
chem3.aLo1_PO4	0	1	Low polynomial coefficients	Global
chem3.aHi1_PO4	0	1	High polynomial coefficients	Global
chem3.aLo2_PO4	0	1/K	Low polynomial coefficients	Global
chem3.aHi2_PO4	0	1/K	High polynomial coefficients	Global
chem3.aLo3_PO4	0	$1/K^2$	Low polynomial coefficients	Global
chem3.aHi3_PO4	0	$1/K^2$	High polynomial coefficients	Global
chem3.aLo4_PO4	0	$1/K^3$	Low polynomial coefficients	Global
chem3.aHi4_PO4	0	$1/K^3$	High polynomial coefficients	Global
chem3.aLo5_PO4	0	$1/K^4$	Low polynomial coefficients	Global
chem3.aHi5_P	0	$1/K^4$	High polynomial	Global

Name	Expression	Unit	Description	Selection
O4			coefficients	
chem3.aLo6_PO4	0	K	Low polynomial coefficients	Global
chem3.aHi6_PO4	0	K	High polynomial coefficients	Global
chem3.aLo7_PO4	0	1	Low polynomial coefficients	Global
chem3.aHi7_PO4	0	1	High polynomial coefficients	Global
chem3.Cp_PO4	R_const*((chem3.T<=chem3.Tlo_PO4)*(chem3.aLo1_PO4+chem3.Tlo_PO4*(chem3.aLo2_PO4+chem3.Tlo_PO4*(chem3.aLo3_PO4+chem3.Tlo_PO4*(chem3.aLo4_PO4+chem3.Tlo_PO4*chem3.aLo5_PO4))))+(chem3.T>chem3.Tlo_PO4)*(chem3.T<=chem3.Tmid_PO4)*(chem3.aLo1_PO4+chem3.T*(chem3.aLo2_PO4+chem3.T*(chem3.aLo3_PO4+chem3.T*(chem3.aLo4_PO4+chem3.T*chem3.aLo5_PO4))))+(chem3.T>chem3.Tmid_PO4)*(chem3.T<=chem3.Thi_PO4)*(chem3.aHi1_PO4+chem3.T*(chem3.aHi2_PO4+chem3.T*(chem3.aHi3_PO4+chem3.T*(chem3.aHi4_PO4+chem3.T*chem3.aHi5_PO4))))+(chem3.T>chem3.Thi_PO4)*(chem3.aHi1_PO4+chem3.Thi_PO4*(chem3.aHi2_PO4+chem3.Thi_PO4*(chem3.aHi3_PO4+chem3.Thi_PO4*(chem3.aHi4_PO4+chem3.Thi_PO4*chem3.aHi5_PO4)))))	J/(mol·K)	Heat capacity at constant pressure	Global
chem3.h_PO4	R_const*((chem3.T<=chem3.Tlo_PO4)*(chem3.aLo6_PO4+chem3.Tlo_PO4*(chem3.aLo1_PO4+0.5*chem3.Tlo_PO4*(chem3.aLo2_PO4+2*chem3.Tlo_PO4*(chem3.aLo3_PO4+0.75*chem3.Tlo_PO4*(chem3.aLo4_PO4+0.8*chem3.Tlo_PO4*chem3.aLo5_PO4))/3)))+(chem3.T>chem3.Tlo_PO4)*(chem3.T<=chem3.Tmid_PO4)*(chem3.aLo6_PO4+chem3.T*(chem3.aLo1_PO4+0.5*chem3.T*(chem3.aLo2_PO4+2*chem3.T*(chem3.aLo3_PO4+0.75*chem3.T*(chem3.aLo4_PO4+0.8*chem3.T*chem3.aLo5_PO4))/3)))+(chem3.T>chem3.Tmid_PO4)*(chem3.T<=chem3.Thi_PO4)*(chem3.aHi6_PO4+chem3.T*(chem3.aHi1_PO4+0.5*chem3.T*(chem3.aHi2_PO4+2*chem3.T*(chem3.aHi3_PO4+0.75*chem3.	J/mol	Molar enthalpy	Global

Name	Expression	Unit	Description	Selection
	T*(chem3.aHi4_PO4+0.8*chem3.T*chem3.aHi5_PO4))/3)))+(chem3.T>chem3.Thi_PO4)*(chem3.aHi6_PO4+chem3.Thi_PO4*(chem3.aHi1_PO4+0.5*chem3.Thi_PO4*(chem3.aHi2_PO4+2*chem3.Thi_PO4*(chem3.aHi3_PO4+0.75*chem3.Thi_PO4*(chem3.aHi4_PO4+0.8*chem3.Thi_PO4*chem3.aHi5_PO4))/3))))			
chem3.s_PO4	R_const*((chem3.T<=chem3.Tlo_PO4)*(chem3.aLo7_PO4+chem3.aLo1_PO4*log(chem3.T/1[K])+chem3.Tlo_PO4*(chem3.aLo2_PO4+0.5*chem3.Tlo_PO4*(chem3.aLo3_PO4+2*chem3.Tlo_PO4*(chem3.aLo4_PO4+0.75*chem3.Tlo_PO4*chem3.aLo5_PO4)/3)))+(chem3.T>chem3.Tlo_PO4)*(chem3.T<=chem3.Tmid_PO4)*(chem3.aLo7_PO4+chem3.aLo1_PO4*log(chem3.T/1[K])+chem3.T*(chem3.aLo2_PO4+0.5*chem3.T*(chem3.aLo3_PO4+2*chem3.T*(chem3.aLo4_PO4+0.75*chem3.T*chem3.aLo5_PO4)/3)))+(chem3.T>chem3.Tmid_PO4)*(chem3.T<=chem3.Thi_PO4)*(chem3.aHi7_PO4+chem3.aHi1_PO4*log(chem3.T/1[K])+chem3.T*(chem3.aHi2_PO4+0.5*chem3.T*(chem3.aHi3_PO4+2*chem3.T*(chem3.aHi4_PO4+0.75*chem3.T*chem3.aHi5_PO4)/3)))+(chem3.T>chem3.Thi_PO4)*(chem3.aHi7_PO4+chem3.aHi1_PO4*log(chem3.T/1[K])+chem3.Thi_PO4*(chem3.aHi2_PO4+0.5*chem3.Thi_PO4*(chem3.aHi3_PO4+2*chem3.Thi_PO4*(chem3.aHi4_PO4+0.75*chem3.Thi_PO4*chem3.aHi5_PO4)/3))))	J/(mol·K)	Molar entropy	Global

2.11.11 4: H2O<=>OH+H

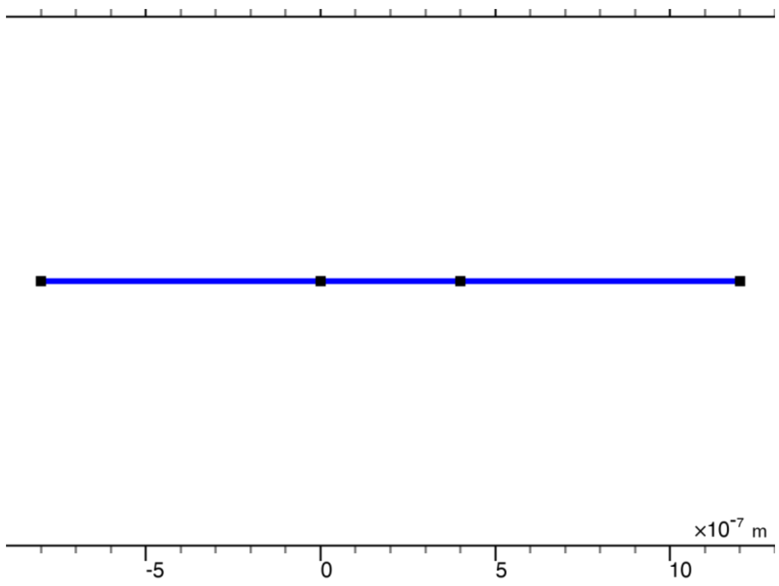

4: H2O<=>OH+H

SELECTION

Geometric entity level	Domain
Selection	Geometry geom1: Dimension 1: All domains

Reaction Formula

SETTINGS

Description	Value
Formula	H2O<=>OH + H
Reaction type	Reversible

Reaction Rate

SETTINGS

Description	Value
Reaction rate	Mass action law
Description	Overall forward reaction order: 1 Overall reverse reaction order: 2

Rate Constants

SETTINGS

Description	Value
Specify equilibrium constant	On
Use Arrhenius expressions	Off
Forward rate constant	1E5

Equilibrium Settings

SETTINGS

Description	Value
Equilibrium constant	User defined
Equilibrium constant	K_H2O

Reaction Thermodynamic Properties

SETTINGS

Description	Value
Enthalpy of reaction	Automatic
Entropy of reaction	Automatic
Heat source of reaction	Automatic

Variables

Name	Expression	Unit	Description	Selection	Details
chem3.Rsum_H	chem3.r_4	mol/(m³·s)	Reaction rate for species H	Global	+ operation
chem3.R_H	chem3.r_4	mol/(m³·s)	Reaction rate for species H	Global	+ operation
chem3.Rsum_H2O	0	mol/(m³·s)	Reaction rate for species H2O	Global	+ operation
chem3.Rsum_OH	chem3.r_4	mol/(m³·s)	Reaction rate for species OH	Global	+ operation
chem3.R_OH	chem3.r_4	mol/(m³·s)	Reaction rate for species OH	Global	+ operation
chem3.Qheat	-chem3.r_4*chem3.H_4	W/m³	Heat source of reactions	Global	+ operation
chem3.R_H2O	0	mol/(m³·s)	Reaction rate for species H2O	Global	+ operation
chem3.kf_4	100000	1/s	Forward rate constant	Global	
chem3.Keq0_4	K_H2O		Equilibrium constant	Global	
chem3.kr_4	chem3.kf_4*1[m^3/(s*mol)]/(chem3.Keq0_4*1[1/s])	m³/(s·mol)	Reverse rate constant	Global	
chem3.r_4	chem3.kf_4*chem3.c_H2O-chem3.kr_4*chem3.c_OH*chem3.c_H	mol/(m³·s)	Reaction rate	Global	
chem3.H_4	-chem3.h_H2O+chem3.h_OH+chem3.h_H	J/mol	Enthalpy of reaction	Global	

Name	Expression	Unit	Description	Selection	Details
chem3.S_4	-chem3.s_H2O+chem3.s_OH+chem3.s_H	J/(mol·K)	Entropy of reaction	Global	
chem3.Q_4	-chem3.r_4*chem3.H_4	W/m³	Heat source of reaction	Global	

2.11.12 Species: H2O

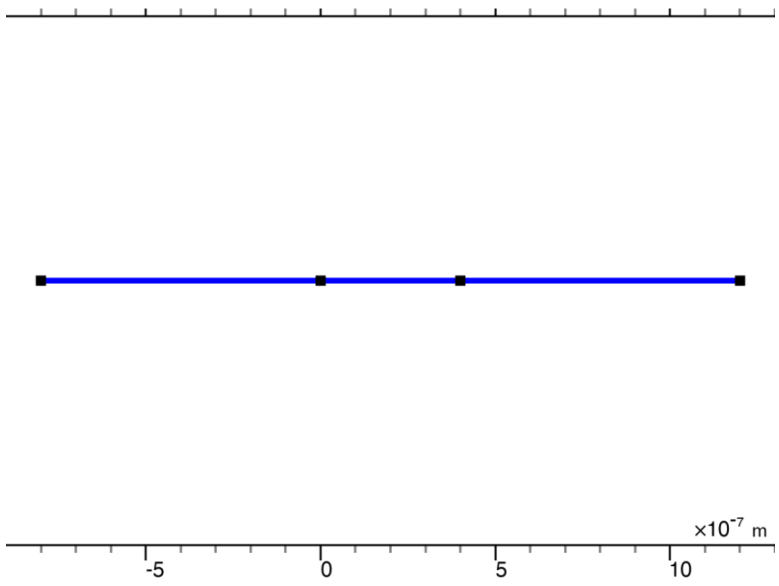

Species: H2O

SELECTION

Geometric entity level	Domain
Selection	Geometry geom1: Dimension 1: All domains

SETTINGS

Description	Value
Species name	H2O

Species Type

SETTINGS

Description	Value
Species type	Solvent

General Parameters

SETTINGS

Description	Value

Description	Value
Molar mass	PM_H2O
Charge	0

Species Thermodynamic Expressions

SETTINGS

Description	Value
Species enthalpy	NASA format
Lower temperature limit	300[K]
Middle temperature limit	1000[K]
Upper temperature limit	5000[K]
	0
	0
	0
	0
	0
	0
	0
	0
	0
	0
	0
	0
	0
	0

Variables

Name	Expression	Unit	Description	Selection	Details
chem3.Rsum_H2O	0	mol/(m³·s)	Reaction rate for species H2O	Global	+ operation
chem3.R_H2O	0	mol/(m³·s)	Reaction rate for species H2O	Global	+ operation
chem3.M_H2O	PM_H2O	kg/mol	Molar mass	Global	
chem3.z_H2O	0	1	Charge	Global	
chem3.Tlo_H2O	300[K]	K	Lower temperature limit	Global	
chem3.Tmid_H2O	1000[K]	K	Middle temperature limit	Global	

Name	Expression	Unit	Description	Selection	Details
chem3.Thi_H2O	5000[K]	K	Upper temperature limit	Global	
chem3.aLo1_H2O	0	1	Low polynomial coefficients	Global	
chem3.aHi1_H2O	0	1	High polynomial coefficients	Global	
chem3.aLo2_H2O	0	1/K	Low polynomial coefficients	Global	
chem3.aHi2_H2O	0	1/K	High polynomial coefficients	Global	
chem3.aLo3_H2O	0	$1/K^2$	Low polynomial coefficients	Global	
chem3.aHi3_H2O	0	$1/K^2$	High polynomial coefficients	Global	
chem3.aLo4_H2O	0	$1/K^3$	Low polynomial coefficients	Global	
chem3.aHi4_H2O	0	$1/K^3$	High polynomial coefficients	Global	
chem3.aLo5_H2O	0	$1/K^4$	Low polynomial coefficients	Global	
chem3.aHi5_H2O	0	$1/K^4$	High polynomial coefficients	Global	
chem3.aLo6_H2O	0	K	Low polynomial coefficients	Global	
chem3.aHi6_H2O	0	K	High polynomial coefficients	Global	
chem3.aLo7_H2O	0	1	Low polynomial coefficients	Global	
chem3.aHi7_H2O	0	1	High polynomial coefficients	Global	
chem3.Cp_H2O	R_const*((chem3.T<=chem3.Tlo_H2O)*(chem3.aLo1_H2O+chem3.Tlo_H2O*(chem3.aLo2_H2O+chem3.Tlo_H2O*(chem3.aLo3_H2O+chem3.Tlo_H2O*(chem3.aLo4_H2O+chem3.Tlo_H2O*chem3.aLo5_H2O))))+(chem3.T>chem3.Tlo_H2O)*(chem3.T<=chem3.Tmid_H2O)*(chem3.aLo1_H2O+chem	J/(mol·K)	Heat capacity at constant pressure	Global	

Name	Expression	Unit	Description	Selection	Details
	3.T*(chem3.aLo2_H2O+chem3.T*(chem3.aLo3_H2O+chem3.T*(chem3.aLo4_H2O+chem3.T*chem3.aLo5_H2O))))+(chem3.T>chem3.Tmid_H2O)*(chem3.T<=chem3.Thi_H2O)*(chem3.aHi1_H2O+chem3.T*(chem3.aHi2_H2O+chem3.T*(chem3.aHi3_H2O+chem3.T*(chem3.aHi4_H2O+chem3.T*chem3.aHi5_H2O))))+(chem3.T>chem3.Thi_H2O)*(chem3.aHi1_H2O+chem3.Thi_H2O*(chem3.aHi2_H2O+chem3.Thi_H2O*(chem3.aHi3_H2O+chem3.Thi_H2O*(chem3.aHi4_H2O+chem3.Thi_H2O*chem3.aHi5_H2O)))))				
chem3.h_H2O	R_const*((chem3.T<=chem3.Tlo_H2O)*(chem3.aLo6_H2O+chem3.Tlo_H2O*(chem3.aLo1_H2O+0.5*chem3.Tlo_H2O*(chem3.aLo2_H2O+2*chem3.Tlo_H2O*(chem3.aLo3_H2O+0.75*chem3.Tlo_H2O*(chem3.aLo4_H2O+0.8*chem3.Tlo_H2O*chem3.aLo5_H2O))/3)))+(chem3.T>chem3.Tlo_H2O)*(chem3.T<=chem3.Tmid_H2O)*(chem3.aLo6_H2O+chem3.T*(chem3.aLo1_H2O+0.5*chem3.T*(chem3.aLo2_H2O+2*chem3.T*(chem3.aLo3_H2O+0.75*chem3.T*(chem3.aLo4_H2O+0.8*chem3.T*chem3.aLo5_H2O))/3)))+(chem3.T>chem3.Tmid_H2O)*(chem3.T<=chem3.Thi_H2O)*(chem3.aHi6_H2O+chem3.T*(chem3.aHi1_H2O+0.5*chem3.T*(c	J/mol	Molar enthalpy	Global	

Name	Expression	Unit	Description	Selection	Details
	hem3.aHi2_H2O+2*chem3.T*(chem3.aHi3_H2O+0.75*chem3.T*(chem3.aHi4_H2O+0.8*chem3.T*chem3.aHi5_H2O))/3)))+(chem3.T>chem3.Thi_H2O)*(chem3.aHi6_H2O+chem3.Thi_H2O*(chem3.aHi1_H2O+0.5*chem3.Thi_H2O*(chem3.aHi2_H2O+2*chem3.Thi_H2O*(chem3.aHi3_H2O+0.75*chem3.Thi_H2O*(chem3.aHi4_H2O+0.8*chem3.Thi_H2O*chem3.aHi5_H2O))/3))))				
chem3.s_H2O	R_const*((chem3.T<=chem3.Tlo_H2O)*(chem3.aLo7_H2O+chem3.aLo1_H2O*log(chem3.T/1[K])+chem3.Tlo_H2O*(chem3.aLo2_H2O+0.5*chem3.Tlo_H2O*(chem3.aLo3_H2O+2*chem3.Tlo_H2O*(chem3.aLo4_H2O+0.75*chem3.Tlo_H2O*chem3.aLo5_H2O)/3)))+(chem3.T>chem3.Tlo_H2O)*(chem3.T<=chem3.Tmid_H2O)*(chem3.aLo7_H2O+chem3.aLo1_H2O*log(chem3.T/1[K])+chem3.T*(chem3.aLo2_H2O+0.5*chem3.T*(chem3.aLo3_H2O+2*chem3.T*(chem3.aLo4_H2O+0.75*chem3.T*chem3.aLo5_H2O)/3)))+(chem3.T>chem3.Tmid_H2O)*(chem3.T<=chem3.Thi_H2O)*(chem3.aHi7_H2O+chem3.aHi1_H2O*log(chem3.T/1[K])+chem3.T*(chem3.aHi2_H2O+0.5*chem3.T*(chem3.aHi3_H2O+2*chem3.T*(chem3.aHi4_H2O+0.75*chem3.T*chem3.aHi5_H2O)/3)	J/(mol·K)	Molar entropy	Global	

Name	Expression	Unit	Description	Selection	Details
	))+(chem3.T>chem3.Thi_H2O)*(chem3.aHi7_H2O+chem3.aHi1_H2O*log(chem3.T/1[K])+chem3.Thi_H2O*(chem3.aHi2_H2O+0.5*chem3.Thi_H2O*(chem3.aHi3_H2O+2*chem3.Thi_H2O*(chem3.aHi4_H2O+0.75*chem3.Thi_H2O*chem3.aHi5_H2O)/3))))				

2.11.13 Species: OH

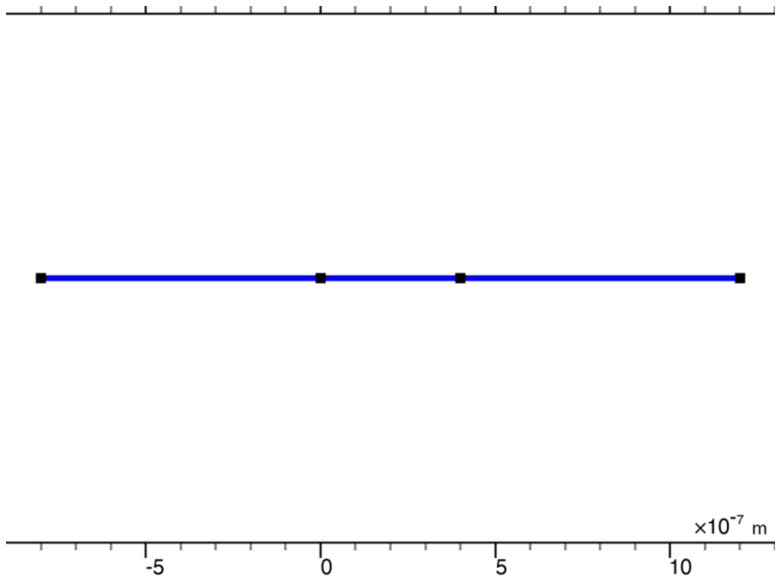

Species: OH

SELECTION

Geometric entity level	Domain
Selection	Geometry geom1: Dimension 1: All domains

SETTINGS

Description	Value
Species name	OH

Species Type

SETTINGS

Description	Value
Species type	Bulk species

General Parameters

SETTINGS

Description	Value
Molar mass	PM_OH
Charge	-1

Reaction Rate

SETTINGS

Description	Value
	Automatic

Species Concentration/Activity

SETTINGS

Description	Value
Constant concentration/activity	Off

Additional Source

SETTINGS

Description	Value
Additional source	Off

Species Thermodynamic Expressions

SETTINGS

Description	Value
Species enthalpy	NASA format
Lower temperature limit	300[K]
Middle temperature limit	1000[K]
Upper temperature limit	5000[K]
	0
	0
	0
	0
	0
	0
	0
	0
	0
	0

Description	Value
	0
	0
	0
	0

Variables

Name	Expression	Unit	Description	Selection
chem3.M_OH	PM_OH	kg/mol	Molar mass	Global
chem3.z_OH	-1	1	Charge	Global
chem3.Tlo_OH	300[K]	K	Lower temperature limit	Global
chem3.Tmid_OH	1000[K]	K	Middle temperature limit	Global
chem3.Thi_OH	5000[K]	K	Upper temperature limit	Global
chem3.aLo1_OH	0	1	Low polynomial coefficients	Global
chem3.aHi1_OH	0	1	High polynomial coefficients	Global
chem3.aLo2_OH	0	1/K	Low polynomial coefficients	Global
chem3.aHi2_OH	0	1/K	High polynomial coefficients	Global
chem3.aLo3_OH	0	$1/K^2$	Low polynomial coefficients	Global
chem3.aHi3_OH	0	$1/K^2$	High polynomial coefficients	Global
chem3.aLo4_OH	0	$1/K^3$	Low polynomial coefficients	Global
chem3.aHi4_OH	0	$1/K^3$	High polynomial coefficients	Global
chem3.aLo5_OH	0	$1/K^4$	Low polynomial coefficients	Global
chem3.aHi5_OH	0	$1/K^4$	High polynomial coefficients	Global
chem3.aLo6_OH	0	K	Low polynomial coefficients	Global
chem3.aHi6_OH	0	K	High polynomial coefficients	Global
chem3.aLo7_OH	0	1	Low polynomial	Global

Name	Expression	Unit	Description	Selection
			coefficients	
chem3.aHi7_OH	0	1	High polynomial coefficients	Global
chem3.Cp_OH	R_const*((chem3.T<=chem3.Tlo_OH)*(chem3.aLo1_OH+chem3.Tlo_OH*(chem3.aLo2_OH+chem3.Tlo_OH*(chem3.aLo3_OH+chem3.Tlo_OH*(chem3.aLo4_OH+chem3.Tlo_OH*chem3.aLo5_OH))))+(chem3.T>chem3.Tlo_OH)*(chem3.T<=chem3.Tmid_OH)*(chem3.aLo1_OH+chem3.T*(chem3.aLo2_OH+chem3.T*(chem3.aLo3_OH+chem3.T*(chem3.aLo4_OH+chem3.T*chem3.aLo5_OH))))+(chem3.T>chem3.Tmid_OH)*(chem3.T<=chem3.Thi_OH)*(chem3.aHi1_OH+chem3.T*(chem3.aHi2_OH+chem3.T*(chem3.aHi3_OH+chem3.T*(chem3.aHi4_OH+chem3.T*chem3.aHi5_OH))))+(chem3.T>chem3.Thi_OH)*(chem3.aHi1_OH+chem3.Thi_OH*(chem3.aHi2_OH+chem3.Thi_OH*(chem3.aHi3_OH+chem3.Thi_OH*(chem3.aHi4_OH+chem3.Thi_OH*chem3.aHi5_OH)))))	J/(mol·K)	Heat capacity at constant pressure	Global
chem3.h_OH	R_const*((chem3.T<=chem3.Tlo_OH)*(chem3.aLo6_OH+chem3.Tlo_OH*(chem3.aLo1_OH+0.5*chem3.Tlo_OH*(chem3.aLo2_OH+2*chem3.Tlo_OH*(chem3.aLo3_OH+0.75*chem3.Tlo_OH*(chem3.aLo4_OH+0.8*chem3.Tlo_OH*chem3.aLo5_OH))/3)))+(chem3.T>chem3.Tlo_OH)*(chem3.T<=chem3.Tmid_OH)*(chem3.aLo6_OH+chem3.T*(chem3.aLo1_OH+0.5*chem3.T*(chem3.aLo2_OH+2*chem3.T*(chem3.aLo3_OH+0.75*chem3.T*(chem3.aLo4_OH+0.8*chem3.T*chem3.aLo5_OH))/3)))+(chem3.T>chem3.Tmid_OH)*(chem3.T<=chem3.Thi_OH)*(chem3.aHi6_OH+chem3.T*(chem3.aHi1_OH+0.5*chem3.T*(chem3.aHi2_OH+2*chem3.T*(chem3.aHi3_OH+0.75*chem3.T*(chem3.aHi4_OH+0.8*chem3.T*chem3.aHi5_OH))/3)))+(chem3.T>chem3.Thi_OH)*(chem3.aHi6_OH+chem3.Thi_OH*(chem3.aHi1_	J/mol	Molar enthalpy	Global

Name	Expression	Unit	Description	Selection
	OH+0.5*chem3.Thi_OH*(chem3.aHi2_OH+2*chem3.Thi_OH*(chem3.aHi3_OH+0.75*chem3.Thi_OH*(chem3.aHi4_OH+0.8*chem3.Thi_OH*chem3.aHi5_OH))/3))))			
chem3.s_OH	R_const*((chem3.T<=chem3.Tlo_OH)*(chem3.aLo7_OH+chem3.aLo1_OH*log(chem3.T/1[K])+chem3.Tlo_OH*(chem3.aLo2_OH+0.5*chem3.Tlo_OH*(chem3.aLo3_OH+2*chem3.Tlo_OH*(chem3.aLo4_OH+0.75*chem3.Tlo_OH*chem3.aLo5_OH)/3)))+(chem3.T>chem3.Tlo_OH)*(chem3.T<=chem3.Tmid_OH)*(chem3.aLo7_OH+chem3.aLo1_OH*log(chem3.T/1[K])+chem3.T*(chem3.aLo2_OH+0.5*chem3.T*(chem3.aLo3_OH+2*chem3.T*(chem3.aLo4_OH+0.75*chem3.T*chem3.aLo5_OH)/3)))+(chem3.T>chem3.Tmid_OH)*(chem3.T<=chem3.Thi_OH)*(chem3.aHi7_OH+chem3.aHi1_OH*log(chem3.T/1[K])+chem3.T*(chem3.aHi2_OH+0.5*chem3.T*(chem3.aHi3_OH+2*chem3.T*(chem3.aHi4_OH+0.75*chem3.T*chem3.aHi5_OH)/3)))+(chem3.T>chem3.Thi_OH)*(chem3.aHi7_OH+chem3.aHi1_OH*log(chem3.T/1[K])+chem3.Thi_OH*(chem3.aHi2_OH+0.5*chem3.Thi_OH*(chem3.aHi3_OH+2*chem3.Thi_OH*(chem3.aHi4_OH+0.75*chem3.Thi_OH*chem3.aHi5_OH)/3))))	J/(mol·K)	Molar entropy	Global

2.11.14 5: CO2+H2O<=>HCO3+H

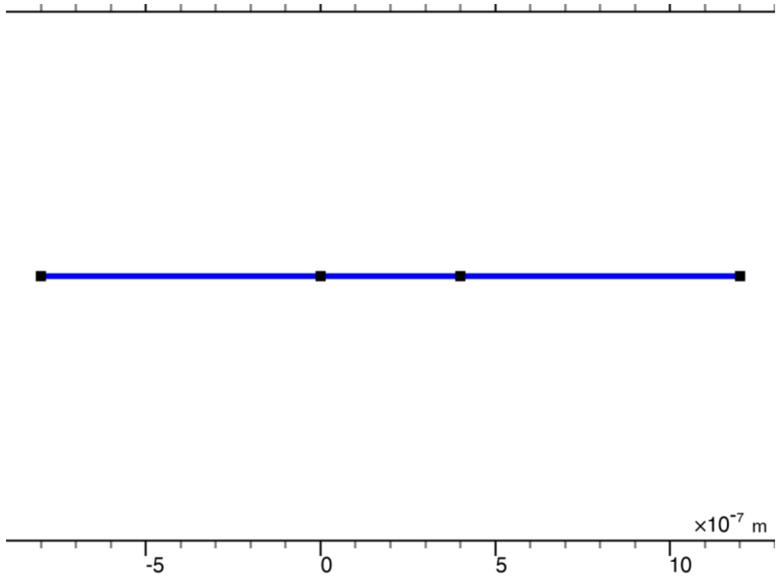

5: CO2+H2O<=>HCO3+H

SELECTION

Geometric entity level	Domain
Selection	Geometry geom1: Dimension 1: All domains

Reaction Formula

SETTINGS

**Description**	**Value**
Formula	CO2 + H2O<=>HCO3 + H
Reaction type	Reversible

Reaction Rate

SETTINGS

**Description**	**Value**
Reaction rate	Mass action law
Description	Overall forward reaction order: 2 Overall reverse reaction order: 2

Rate Constants

SETTINGS

**Description**	**Value**
Specify equilibrium constant	On
Use Arrhenius expressions	Off
Forward rate constant	K_CO2kf

Equilibrium Settings

SETTINGS

Description	Value
Equilibrium constant	User defined
Equilibrium constant	K_CO2

Reaction Thermodynamic Properties

SETTINGS

Description	Value
Enthalpy of reaction	Automatic
Entropy of reaction	Automatic
Heat source of reaction	Automatic

Variables

Name	Expression	Unit	Description	Selection	Details
chem3.Rsum_CO2	-chem3.r_5	mol/(m³·s)	Reaction rate for species CO2	Global	+ operation
chem3.R_CO2	-chem3.r_5	mol/(m³·s)	Reaction rate for species CO2	Global	+ operation
chem3.Rsum_H	chem3.r_5	mol/(m³·s)	Reaction rate for species H	Global	+ operation
chem3.R_H	chem3.r_5	mol/(m³·s)	Reaction rate for species H	Global	+ operation
chem3.Rsum_H2O	0	mol/(m³·s)	Reaction rate for species H2O	Global	+ operation
chem3.Rsum_HCO3	chem3.r_5	mol/(m³·s)	Reaction rate for species HCO3	Global	+ operation
chem3.R_HCO3	chem3.r_5	mol/(m³·s)	Reaction rate for species HCO3	Global	+ operation
chem3.Qheat	-chem3.r_5*chem3.H_5	W/m³	Heat source of reactions	Global	+ operation
chem3.R_H2O	0	mol/(m³·s)	Reaction rate for species H2O	Global	+ operation
chem3.kf_5	K_CO2kf	m³/(s·mol)	Forward rate constant	Global	
chem3.Keq0_5	K_CO2		Equilibrium constant	Global	
chem3.kr_5	chem3.kf_5/chem3.Keq0_5	m³/(s·mol)	Reverse rate constant	Global	

Name	Expression	Unit	Description	Selection	Details
chem3.r_5	chem3.kf_5*chem3.c_CO2*chem3.c_H2O-chem3.kr_5*chem3.c_HCO3*chem3.c_H	mol/(m³·s)	Reaction rate	Global	
chem3.H_5	-chem3.h_CO2-chem3.h_H2O+chem3.h_HCO3+chem3.h_H	J/mol	Enthalpy of reaction	Global	
chem3.S_5	-chem3.s_CO2-chem3.s_H2O+chem3.s_HCO3+chem3.s_H	J/(mol·K)	Entropy of reaction	Global	
chem3.Q_5	-chem3.r_5*chem3.H_5	W/m³	Heat source of reaction	Global	

2.11.15 Species: CO2

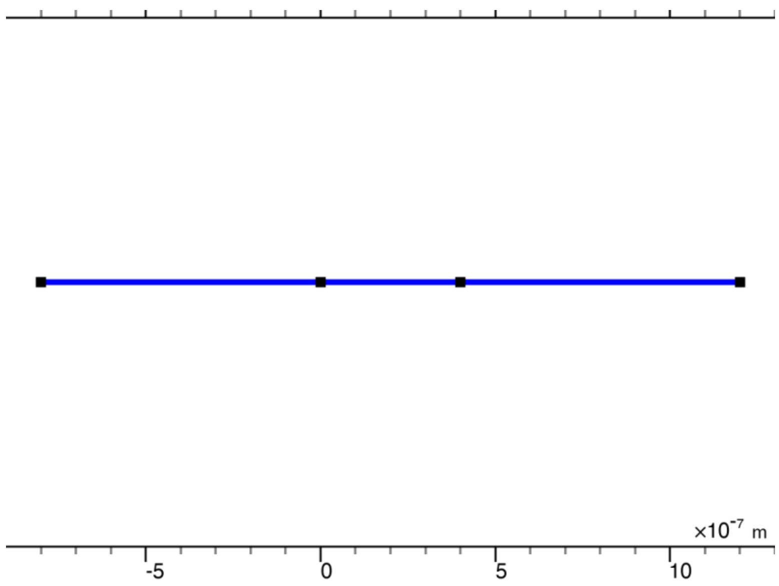

Species: CO2

SELECTION

Geometric entity level	Domain
Selection	Geometry geom1: Dimension 1: All domains

SETTINGS

Description	Value
Species name	CO2

Species Type

SETTINGS

Description	Value
Species type	Bulk species

General Parameters

SETTINGS

Description	Value
Molar mass	PM_CO2
Charge	0

Reaction Rate

SETTINGS

Description	Value
	Automatic

Species Concentration/Activity

SETTINGS

Description	Value
Constant concentration/activity	Off

Additional Source

SETTINGS

Description	Value
Additional source	Off

Species Thermodynamic Expressions

SETTINGS

Description	Value
Species enthalpy	NASA format
Lower temperature limit	300[K]
Middle temperature limit	1000[K]
Upper temperature limit	5000[K]
	0
	0
	0
	0
	0
	0
	0

Description	Value
	0
	0
	0
	0
	0
	0
	0

Variables

Name	Expression	Unit	Description	Selection
chem3.M_CO2	PM_CO2	kg/mol	Molar mass	Global
chem3.z_CO2	0	1	Charge	Global
chem3.Tlo_CO2	300[K]	K	Lower temperature limit	Global
chem3.Tmid_CO2	1000[K]	K	Middle temperature limit	Global
chem3.Thi_CO2	5000[K]	K	Upper temperature limit	Global
chem3.aLo1_CO2	0	1	Low polynomial coefficients	Global
chem3.aHi1_CO2	0	1	High polynomial coefficients	Global
chem3.aLo2_CO2	0	1/K	Low polynomial coefficients	Global
chem3.aHi2_CO2	0	1/K	High polynomial coefficients	Global
chem3.aLo3_CO2	0	$1/K^2$	Low polynomial coefficients	Global
chem3.aHi3_CO2	0	$1/K^2$	High polynomial coefficients	Global
chem3.aLo4_CO2	0	$1/K^3$	Low polynomial coefficients	Global
chem3.aHi4_CO2	0	$1/K^3$	High polynomial coefficients	Global
chem3.aLo5_CO2	0	$1/K^4$	Low polynomial coefficients	Global
chem3.aHi5_CO2	0	$1/K^4$	High polynomial coefficients	Global
chem3.aLo6_C	0	K	Low polynomial	Global

Name	Expression	Unit	Description	Selection
O2			coefficients	
chem3.aHi6_CO2	0	K	High polynomial coefficients	Global
chem3.aLo7_CO2	0	1	Low polynomial coefficients	Global
chem3.aHi7_CO2	0	1	High polynomial coefficients	Global
chem3.Cp_CO2	R_const*((chem3.T<=chem3.Tlo_CO2)*(chem3.aLo1_CO2+chem3.Tlo_CO2*(chem3.aLo2_CO2+chem3.Tlo_CO2*(chem3.aLo3_CO2+chem3.Tlo_CO2*(chem3.aLo4_CO2+chem3.Tlo_CO2*chem3.aLo5_CO2))))+(chem3.T>chem3.Tlo_CO2)*(chem3.T<=chem3.Tmid_CO2)*(chem3.aLo1_CO2+chem3.T*(chem3.aLo2_CO2+chem3.T*(chem3.aLo3_CO2+chem3.T*(chem3.aLo4_CO2+chem3.T*chem3.aLo5_CO2))))+(chem3.T>chem3.Tmid_CO2)*(chem3.T<=chem3.Thi_CO2)*(chem3.aHi1_CO2+chem3.T*(chem3.aHi2_CO2+chem3.T*(chem3.aHi3_CO2+chem3.T*(chem3.aHi4_CO2+chem3.T*chem3.aHi5_CO2))))+(chem3.T>chem3.Thi_CO2)*(chem3.aHi1_CO2+chem3.Thi_CO2*(chem3.aHi2_CO2+chem3.Thi_CO2*(chem3.aHi3_CO2+chem3.Thi_CO2*(chem3.aHi4_CO2+chem3.Thi_CO2*chem3.aHi5_CO2)))))	J/(mol·K)	Heat capacity at constant pressure	Global
chem3.h_CO2	R_const*((chem3.T<=chem3.Tlo_CO2)*(chem3.aLo6_CO2+chem3.Tlo_CO2*(chem3.aLo1_CO2+0.5*chem3.Tlo_CO2*(chem3.aLo2_CO2+2*chem3.Tlo_CO2*(chem3.aLo3_CO2+0.75*chem3.Tlo_CO2*(chem3.aLo4_CO2+0.8*chem3.Tlo_CO2*chem3.aLo5_CO2))/3)))+(chem3.T>chem3.Tlo_CO2)*(chem3.T<=chem3.Tmid_CO2)*(chem3.aLo6_CO2+chem3.T*(chem3.aLo1_CO2+0.5*chem3.T*(chem3.aLo2_CO2+2*chem3.T*(chem3.aLo3_CO2+0.75*chem3.T*(chem3.aLo4_CO2+0.8*chem3.T*chem3.aLo5_CO2))/3)))+(chem3.T>chem3.Tmid_CO2)*(chem3.T<=chem3.Thi_CO2)*(chem3.aHi6_CO2+chem3.T*(chem3.aHi1_CO2+0.5*chem3.T*(chem3.aHi2_CO2+2*chem3.T*(chem3.aHi3_CO2+0.75*chem3.T*(chem3.aHi4_CO2+0.8*chem3.T*chem3.aHi5_CO2))/3)))+(chem3.T>chem	J/mol	Molar enthalpy	Global

Name	Expression	Unit	Description	Selection
	3.Thi_CO2)*(chem3.aHi6_CO2+chem3.Thi_CO2*(chem3.aHi1_CO2+0.5*chem3.Thi_CO2*(chem3.aHi2_CO2+2*chem3.Thi_CO2*(chem3.aHi3_CO2+0.75*chem3.Thi_CO2*(chem3.aHi4_CO2+0.8*chem3.Thi_CO2*chem3.aHi5_CO2))/3))))			
chem3.s_CO2	R_const*((chem3.T<=chem3.Tlo_CO2)*(chem3.aLo7_CO2+chem3.aLo1_CO2*log(chem3.T/1[K])+chem3.Tlo_CO2*(chem3.aLo2_CO2+0.5*chem3.Tlo_CO2*(chem3.aLo3_CO2+2*chem3.Tlo_CO2*(chem3.aLo4_CO2+0.75*chem3.Tlo_CO2*chem3.aLo5_CO2)/3)))+(chem3.T>chem3.Tlo_CO2)*(chem3.T<=chem3.Tmid_CO2)*(chem3.aLo7_CO2+chem3.aLo1_CO2*log(chem3.T/1[K])+chem3.T*(chem3.aLo2_CO2+0.5*chem3.T*(chem3.aLo3_CO2+2*chem3.T*(chem3.aLo4_CO2+0.75*chem3.T*chem3.aLo5_CO2)/3)))+(chem3.T>chem3.Tmid_CO2)*(chem3.T<=chem3.Thi_CO2)*(chem3.aHi7_CO2+chem3.aHi1_CO2*log(chem3.T/1[K])+chem3.T*(chem3.aHi2_CO2+0.5*chem3.T*(chem3.aHi3_CO2+2*chem3.T*(chem3.aHi4_CO2+0.75*chem3.T*chem3.aHi5_CO2)/3)))+(chem3.T>chem3.Thi_CO2)*(chem3.aHi7_CO2+chem3.aHi1_CO2*log(chem3.T/1[K])+chem3.Thi_CO2*(chem3.aHi2_CO2+0.5*chem3.Thi_CO2*(chem3.aHi3_CO2+2*chem3.Thi_CO2*(chem3.aHi4_CO2+0.75*chem3.Thi_CO2*chem3.aHi5_CO2)/3))))	J/(mol·K)	Molar entropy	Global

2.11.16 Species: HCO3

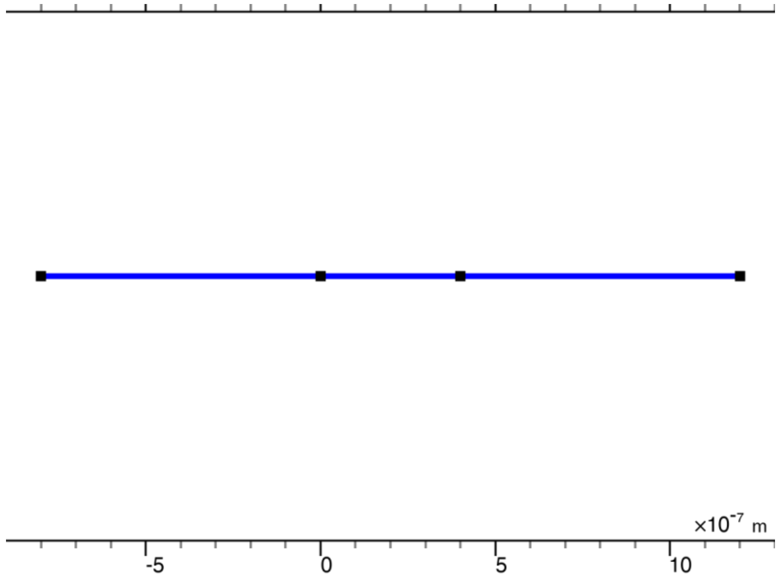

Species: HCO3

SELECTION

Geometric entity level	Domain
Selection	Geometry geom1: Dimension 1: All domains

SETTINGS

Description	Value
Species name	HCO3

Species Type

SETTINGS

Description	Value
Species type	Bulk species

General Parameters

SETTINGS

Description	Value
Molar mass	PM_HCO3
Charge	-1

Reaction Rate

SETTINGS

Description	Value

Description	Value
	Automatic

Species Concentration/Activity

SETTINGS

Description	Value
Constant concentration/activity	Off

Additional Source

SETTINGS

Description	Value
Additional source	Off

Species Thermodynamic Expressions

SETTINGS

Description	Value
Species enthalpy	NASA format
Lower temperature limit	300[K]
Middle temperature limit	1000[K]
Upper temperature limit	5000[K]
	0
	0
	0
	0
	0
	0
	0
	0
	0
	0
	0
	0
	0
	0

Variables

Name	Expression	Unit	Description	Selection
chem3.M_HCO	PM_HCO3	kg/mol	Molar mass	Global

Name	Expression	Unit	Description	Selection
3				
chem3.z_HCO3	-1	1	Charge	Global
chem3.Tlo_HCO3	300[K]	K	Lower temperature limit	Global
chem3.Tmid_HCO3	1000[K]	K	Middle temperature limit	Global
chem3.Thi_HCO3	5000[K]	K	Upper temperature limit	Global
chem3.aLo1_HCO3	0	1	Low polynomial coefficients	Global
chem3.aHi1_HCO3	0	1	High polynomial coefficients	Global
chem3.aLo2_HCO3	0	1/K	Low polynomial coefficients	Global
chem3.aHi2_HCO3	0	1/K	High polynomial coefficients	Global
chem3.aLo3_HCO3	0	$1/K^2$	Low polynomial coefficients	Global
chem3.aHi3_HCO3	0	$1/K^2$	High polynomial coefficients	Global
chem3.aLo4_HCO3	0	$1/K^3$	Low polynomial coefficients	Global
chem3.aHi4_HCO3	0	$1/K^3$	High polynomial coefficients	Global
chem3.aLo5_HCO3	0	$1/K^4$	Low polynomial coefficients	Global
chem3.aHi5_HCO3	0	$1/K^4$	High polynomial coefficients	Global
chem3.aLo6_HCO3	0	K	Low polynomial coefficients	Global
chem3.aHi6_HCO3	0	K	High polynomial coefficients	Global
chem3.aLo7_HCO3	0	1	Low polynomial coefficients	Global
chem3.aHi7_HCO3	0	1	High polynomial coefficients	Global
chem3.Cp_HCO3	R_const*((chem3.T<=chem3.Tlo_HCO3)*(chem3.aLo1_HCO3+chem3.Tlo_HCO3*(chem3.aLo2_HCO3+chem3.Tlo_HCO3*(chem3.aLo3_HCO3+chem3.Tlo_HCO3*(chem3.aLo4_HCO3+chem3.Tlo_HCO3*ch	J/(mol·K)	Heat capacity at constant pressure	Global

Name	Expression	Unit	Description	Selection
	em3.aLo5_HCO3))))+(chem3.T>chem3.Tlo_HCO3)*(chem3.T<=chem3.Tmid_HCO3)*(chem3.aLo1_HCO3+chem3.T*(chem3.aLo2_HCO3+chem3.T*(chem3.aLo3_HCO3+chem3.T*(chem3.aLo4_HCO3+chem3.T*chem3.aLo5_HCO3))))+(chem3.T>chem3.Tmid_HCO3)*(chem3.T<=chem3.Thi_HCO3)*(chem3.aHi1_HCO3+chem3.T*(chem3.aHi2_HCO3+chem3.T*(chem3.aHi3_HCO3+chem3.T*(chem3.aHi4_HCO3+chem3.T*chem3.aHi5_HCO3))))+(chem3.T>chem3.Thi_HCO3)*(chem3.aHi1_HCO3+chem3.Thi_HCO3*(chem3.aHi2_HCO3+chem3.Thi_HCO3*(chem3.aHi3_HCO3+chem3.Thi_HCO3*(chem3.aHi4_HCO3+chem3.Thi_HCO3*chem3.aHi5_HCO3)))))			
chem3.h_HCO3	R_const*((chem3.T<=chem3.Tlo_HCO3)*(chem3.aLo6_HCO3+chem3.Tlo_HCO3*(chem3.aLo1_HCO3+0.5*chem3.Tlo_HCO3*(chem3.aLo2_HCO3+2*chem3.Tlo_HCO3*(chem3.aLo3_HCO3+0.75*chem3.Tlo_HCO3*(chem3.aLo4_HCO3+0.8*chem3.Tlo_HCO3*chem3.aLo5_HCO3))/3)))+(chem3.T>chem3.Tlo_HCO3)*(chem3.T<=chem3.Tmid_HCO3)*(chem3.aLo6_HCO3+chem3.T*(chem3.aLo1_HCO3+0.5*chem3.T*(chem3.aLo2_HCO3+2*chem3.T*(chem3.aLo3_HCO3+0.75*chem3.T*(chem3.aLo4_HCO3+0.8*chem3.T*chem3.aLo5_HCO3))/3)))+(chem3.T>chem3.Tmid_HCO3)*(chem3.T<=chem3.Thi_HCO3)*(chem3.aHi6_HCO3+chem3.T*(chem3.aHi1_HCO3+0.5*chem3.T*(chem3.aHi2_HCO3+2*chem3.T*(chem3.aHi3_HCO3+0.75*chem3.T*(chem3.aHi4_HCO3+0.8*chem3.T*chem3.aHi5_HCO3))/3)))+(chem3.T>chem3.Thi_HCO3)*(chem3.aHi6_HCO3+chem3.Thi_HCO3*(chem3.aHi1_HCO3+0.5*chem3.Thi_HCO3*(chem3.aHi2_HCO3+2*chem3.Thi_HCO3*(chem3.aHi3_HCO3+0.75*chem3.Thi_HCO3*(chem3.aHi4_HCO3+0.8*chem3.Thi_HCO3*chem3.aHi5_HCO3))/3))))	J/mol	Molar enthalpy	Global
chem3.s_HCO3	R_const*((chem3.T<=chem3.Tlo_HCO3)*(chem3.aLo7_HCO3+chem3.aLo1_HCO3*log(chem3.T/1[K])+chem3.Tlo_HCO3*(c	J/(mol·K)	Molar entropy	Global

Name	Expression	Unit	Description	Selection
	hem3.aLo2_HCO3+0.5*chem3.Tlo_HCO3*(chem3.aLo3_HCO3+2*chem3.Tlo_HCO3*(chem3.aLo4_HCO3+0.75*chem3.Tlo_HCO3*chem3.aLo5_HCO3)/3)))+(chem3.T>chem3.Tlo_HCO3)*(chem3.T<=chem3.Tmid_HCO3)*(chem3.aLo7_HCO3+chem3.aLo1_HCO3*log(chem3.T/1[K])+chem3.T*(chem3.aLo2_HCO3+0.5*chem3.T*(chem3.aLo3_HCO3+2*chem3.T*(chem3.aLo4_HCO3+0.75*chem3.T*chem3.aLo5_HCO3)/3)))+(chem3.T>chem3.Tmid_HCO3)*(chem3.T<=chem3.Thi_HCO3)*(chem3.aHi7_HCO3+chem3.aHi1_HCO3*log(chem3.T/1[K])+chem3.T*(chem3.aHi2_HCO3+0.5*chem3.T*(chem3.aHi3_HCO3+2*chem3.T*(chem3.aHi4_HCO3+0.75*chem3.T*chem3.aHi5_HCO3)/3)))+(chem3.T>chem3.Thi_HCO3)*(chem3.aHi7_HCO3+chem3.aHi1_HCO3*log(chem3.T/1[K])+chem3.Thi_HCO3*(chem3.aHi2_HCO3+0.5*chem3.Thi_HCO3*(chem3.aHi3_HCO3+2*chem3.Thi_HCO3*(chem3.aHi4_HCO3+0.75*chem3.Thi_HCO3*chem3.aHi5_HCO3)/3))))			

2.11.17 6: HCO3<=>CO3+H

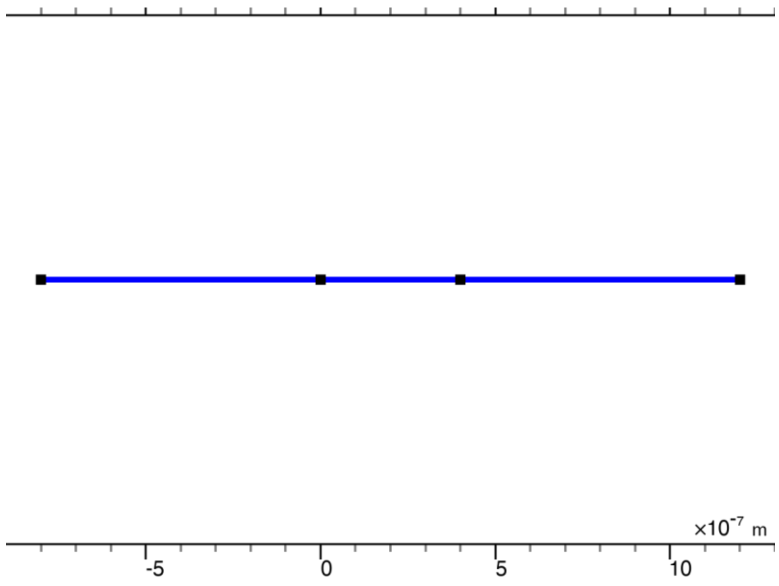

6: HCO3<=>CO3+H

SELECTION

Geometric entity level	Domain
Selection	Geometry geom1: Dimension 1: All domains

Reaction Formula

SETTINGS

Description	Value
Formula	HCO3<=>CO3 + H
Reaction type	Reversible

Reaction Rate

SETTINGS

Description	Value
Reaction rate	Mass action law
Description	Overall forward reaction order: 1 Overall reverse reaction order: 2

Rate Constants

SETTINGS

Description	Value
Specify equilibrium constant	On
Use Arrhenius expressions	Off
Forward rate constant	K_HCO3kf

Equilibrium Settings

SETTINGS

Description	Value
Equilibrium constant	User defined
Equilibrium constant	K_HCO3

Reaction Thermodynamic Properties

SETTINGS

Description	Value
Enthalpy of reaction	Automatic
Entropy of reaction	Automatic
Heat source of reaction	Automatic

Variables

Name	Expression	Unit	Description	Selection	Details
chem3.Rsum_CO3	chem3.r_6	$mol/(m^3 \cdot s)$	Reaction rate for species CO3	Global	+ operation

Name	Expression	Unit	Description	Selection	Details
chem3.R_CO3	chem3.r_6	mol/(m³·s)	Reaction rate for species CO3	Global	+ operation
chem3.Rsum_H	chem3.r_6	mol/(m³·s)	Reaction rate for species H	Global	+ operation
chem3.R_H	chem3.r_6	mol/(m³·s)	Reaction rate for species H	Global	+ operation
chem3.Rsum_HCO3	-chem3.r_6	mol/(m³·s)	Reaction rate for species HCO3	Global	+ operation
chem3.R_HCO3	-chem3.r_6	mol/(m³·s)	Reaction rate for species HCO3	Global	+ operation
chem3.Qheat	-chem3.r_6*chem3.H_6	W/m³	Heat source of reactions	Global	+ operation
chem3.kf_6	K_HCO3kf	1/s	Forward rate constant	Global	
chem3.Keq0_6	K_HCO3		Equilibrium constant	Global	
chem3.kr_6	chem3.kf_6*1[m^3/(s*mol)]/(chem3.Keq0_6*1[1/s])	m³/(s·mol)	Reverse rate constant	Global	
chem3.r_6	chem3.kf_6*chem3.c_HCO3-chem3.kr_6*chem3.c_CO3*chem3.c_H	mol/(m³·s)	Reaction rate	Global	
chem3.H_6	-chem3.h_HCO3+chem3.h_CO3+chem3.h_H	J/mol	Enthalpy of reaction	Global	
chem3.S_6	-chem3.s_HCO3+chem3.s_CO3+chem3.s_H	J/(mol·K)	Entropy of reaction	Global	
chem3.Q_6	-chem3.r_6*chem3.H_6	W/m³	Heat source of reaction	Global	

2.11.18 Species: CO3

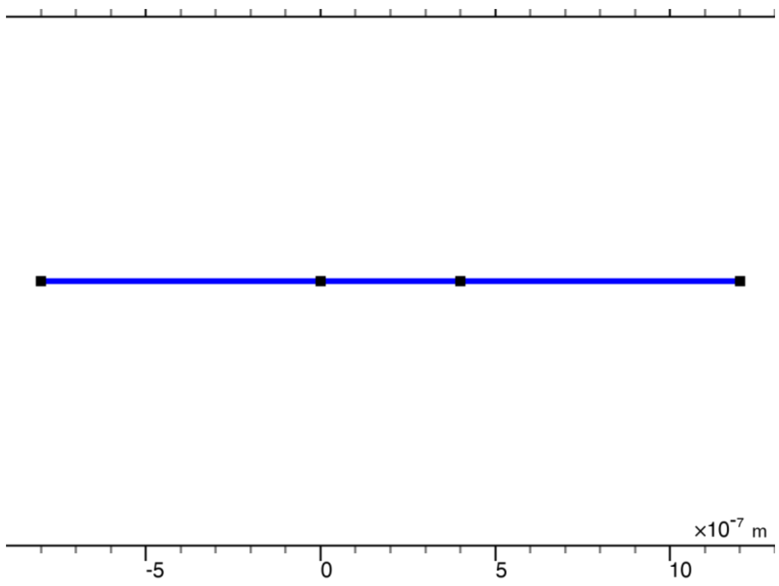

Species: CO3

SELECTION

Geometric entity level	Domain
Selection	Geometry geom1: Dimension 1: All domains

SETTINGS

Description	Value
Species name	CO3

Species Type

SETTINGS

Description	Value
Species type	Bulk species

General Parameters

SETTINGS

Description	Value
Molar mass	PM_CO3
Charge	-2

Reaction Rate

SETTINGS

Description	Value

Description	Value
	Automatic

Species Concentration/Activity

SETTINGS

Description	Value
Constant concentration/activity	Off

Additional Source

SETTINGS

Description	Value
Additional source	Off

Species Thermodynamic Expressions

SETTINGS

Description	Value
Species enthalpy	NASA format
Lower temperature limit	300[K]
Middle temperature limit	1000[K]
Upper temperature limit	5000[K]
	0
	0
	0
	0
	0
	0
	0
	0
	0
	0
	0
	0
	0
	0

Variables

Name	Expression	Unit	Description	Selection
chem3.M_CO3	PM_CO3	kg/mol	Molar mass	Global

Name	Expression	Unit	Description	Selection
chem3.z_CO3	-2	1	Charge	Global
chem3.Tlo_CO3	300[K]	K	Lower temperature limit	Global
chem3.Tmid_CO3	1000[K]	K	Middle temperature limit	Global
chem3.Thi_CO3	5000[K]	K	Upper temperature limit	Global
chem3.aLo1_CO3	0	1	Low polynomial coefficients	Global
chem3.aHi1_CO3	0	1	High polynomial coefficients	Global
chem3.aLo2_CO3	0	1/K	Low polynomial coefficients	Global
chem3.aHi2_CO3	0	1/K	High polynomial coefficients	Global
chem3.aLo3_CO3	0	1/K²	Low polynomial coefficients	Global
chem3.aHi3_CO3	0	1/K²	High polynomial coefficients	Global
chem3.aLo4_CO3	0	1/K³	Low polynomial coefficients	Global
chem3.aHi4_CO3	0	1/K³	High polynomial coefficients	Global
chem3.aLo5_CO3	0	1/K⁴	Low polynomial coefficients	Global
chem3.aHi5_CO3	0	1/K⁴	High polynomial coefficients	Global
chem3.aLo6_CO3	0	K	Low polynomial coefficients	Global
chem3.aHi6_CO3	0	K	High polynomial coefficients	Global
chem3.aLo7_CO3	0	1	Low polynomial coefficients	Global
chem3.aHi7_CO3	0	1	High polynomial coefficients	Global
chem3.Cp_CO3	R_const*((chem3.T<=chem3.Tlo_CO3)*(chem3.aLo1_CO3+chem3.Tlo_CO3*(chem3.aLo2_CO3+chem3.Tlo_CO3*(chem3.aLo3_CO3+chem3.Tlo_CO3*(chem3.aLo4_CO3+chem3.Tlo_CO3*chem3.aLo5_CO3))))+(chem3.T>chem3.Tlo_CO3)*(chem3.	J/(mol·K)	Heat capacity at constant pressure	Global

Name	Expression	Unit	Description	Selection
	T<=chem3.Tmid_CO3)*(chem3.aLo1_CO3+chem3.T*(chem3.aLo2_CO3+chem3.T*(chem3.aLo3_CO3+chem3.T*(chem3.aLo4_CO3+chem3.T*chem3.aLo5_CO3))))+(chem3.T>chem3.Tmid_CO3)*(chem3.T<=chem3.Thi_CO3)*(chem3.aHi1_CO3+chem3.T*(chem3.aHi2_CO3+chem3.T*(chem3.aHi3_CO3+chem3.T*(chem3.aHi4_CO3+chem3.T*chem3.aHi5_CO3))))+(chem3.T>chem3.Thi_CO3)*(chem3.aHi1_CO3+chem3.Thi_CO3*(chem3.aHi2_CO3+chem3.Thi_CO3*(chem3.aHi3_CO3+chem3.Thi_CO3*(chem3.aHi4_CO3+chem3.Thi_CO3*chem3.aHi5_CO3)))))			
chem3.h_CO3	R_const*((chem3.T<=chem3.Tlo_CO3)*(chem3.aLo6_CO3+chem3.Tlo_CO3*(chem3.aLo1_CO3+0.5*chem3.Tlo_CO3*(chem3.aLo2_CO3+2*chem3.Tlo_CO3*(chem3.aLo3_CO3+0.75*chem3.Tlo_CO3*(chem3.aLo4_CO3+0.8*chem3.Tlo_CO3*chem3.aLo5_CO3))/3)))+(chem3.T>chem3.Tlo_CO3)*(chem3.T<=chem3.Tmid_CO3)*(chem3.aLo6_CO3+chem3.T*(chem3.aLo1_CO3+0.5*chem3.T*(chem3.aLo2_CO3+2*chem3.T*(chem3.aLo3_CO3+0.75*chem3.T*(chem3.aLo4_CO3+0.8*chem3.T*chem3.aLo5_CO3))/3)))+(chem3.T>chem3.Tmid_CO3)*(chem3.T<=chem3.Thi_CO3)*(chem3.aHi6_CO3+chem3.T*(chem3.aHi1_CO3+0.5*chem3.T*(chem3.aHi2_CO3+2*chem3.T*(chem3.aHi3_CO3+0.75*chem3.T*(chem3.aHi4_CO3+0.8*chem3.T*chem3.aHi5_CO3))/3)))+(chem3.T>chem3.Thi_CO3)*(chem3.aHi6_CO3+chem3.Thi_CO3*(chem3.aHi1_CO3+0.5*chem3.Thi_CO3*(chem3.aHi2_CO3+2*chem3.Thi_CO3*(chem3.aHi3_CO3+0.75*chem3.Thi_CO3*(chem3.aHi4_CO3+0.8*chem3.Thi_CO3*chem3.aHi5_CO3))/3))))	J/mol	Molar enthalpy	Global
chem3.s_CO3	R_const*((chem3.T<=chem3.Tlo_CO3)*(chem3.aLo7_CO3+chem3.aLo1_CO3*log(chem3.T/1[K])+chem3.Tlo_CO3*(chem3.aLo2_CO3+0.5*chem3.Tlo_CO3*(chem3.aLo3_CO3+2*chem3.Tlo_CO3*(chem3.aLo4_CO3+0.75*chem3.Tlo_CO3*chem3.aLo5_CO3)/3)))+(chem3.T>chem3.Tlo_CO3)*(chem3.T<=chem3.Tmid_CO3)*(chem3	J/(mol·K)	Molar entropy	Global

Name	Expression	Unit	Description	Selection
	.aLo7_CO3+chem3.aLo1_CO3*log(chem3.T/1[K])+chem3.T*(chem3.aLo2_CO3+0.5*chem3.T*(chem3.aLo3_CO3+2*chem3.T*(chem3.aLo4_CO3+0.75*chem3.T*chem3.aLo5_CO3)/3)))+(chem3.T>chem3.Tmid_CO3)*(chem3.T<=chem3.Thi_CO3)*(chem3.aHi7_CO3+chem3.aHi1_CO3*log(chem3.T/1[K])+chem3.T*(chem3.aHi2_CO3+0.5*chem3.T*(chem3.aHi3_CO3+2*chem3.T*(chem3.aHi4_CO3+0.75*chem3.T*chem3.aHi5_CO3)/3)))+(chem3.T>chem3.Thi_CO3)*(chem3.aHi7_CO3+chem3.aHi1_CO3*log(chem3.T/1[K])+chem3.Thi_CO3*(chem3.aHi2_CO3+0.5*chem3.Thi_CO3*(chem3.aHi3_CO3+2*chem3.Thi_CO3*(chem3.aHi4_CO3+0.75*chem3.Thi_CO3*chem3.aHi5_CO3)/3))))			

2.11.19 7: CO2+OH<=>HCO3

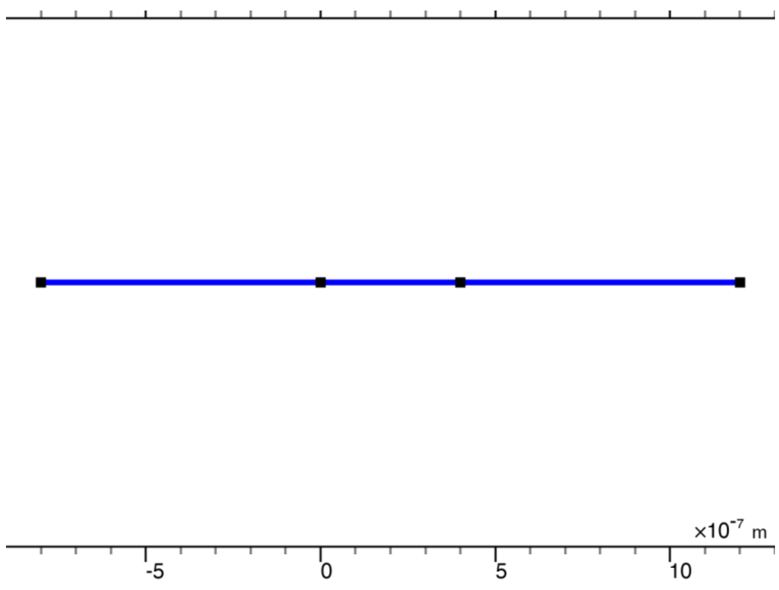

7: CO2+OH<=>HCO3

SELECTION

Geometric entity level	Domain
Selection	Geometry geom1: Dimension 1: All domains

Reaction Formula

SETTINGS

Description	Value

Description	Value
Formula	CO2 + OH<=>HCO3
Reaction type	Reversible

Reaction Rate

SETTINGS

Description	Value
Reaction rate	Mass action law
Description	Overall forward reaction order: 2 Overall reverse reaction order: 1

Rate Constants

SETTINGS

Description	Value
Specify equilibrium constant	On
Use Arrhenius expressions	Off
Forward rate constant	K_CO2_OHkf

Equilibrium Settings

SETTINGS

Description	Value
Equilibrium constant	User defined
Equilibrium constant	K_CO2/K_H2O*1e6

Reaction Thermodynamic Properties

SETTINGS

Description	Value
Enthalpy of reaction	Automatic
Entropy of reaction	Automatic
Heat source of reaction	Automatic

Variables

Name	Expression	Unit	Description	Selection	Details
chem3.Rsum_CO2	-chem3.r_7	$mol/(m^3 \cdot s)$	Reaction rate for species CO2	Global	+ operation
chem3.R_CO2	-chem3.r_7	$mol/(m^3 \cdot s)$	Reaction rate for species CO2	Global	+ operation
chem3.Rsum_HCO3	chem3.r_7	$mol/(m^3 \cdot s)$	Reaction rate for species HCO3	Global	+ operation

Name	Expression	Unit	Description	Selection	Details
chem3.R_HCO3	chem3.r_7	mol/(m³·s)	Reaction rate for species HCO3	Global	+ operation
chem3.Rsum_OH	-chem3.r_7	mol/(m³·s)	Reaction rate for species OH	Global	+ operation
chem3.R_OH	-chem3.r_7	mol/(m³·s)	Reaction rate for species OH	Global	+ operation
chem3.Qheat	-chem3.r_7*chem3.H_7	W/m³	Heat source of reactions	Global	+ operation
chem3.kf_7	K_CO2_OHkf	m³/(s·mol)	Forward rate constant	Global	
chem3.Keq0_7	1000000*K_CO2/K_H2O		Equilibrium constant	Global	
chem3.kr_7	chem3.kf_7*1[1/s]/(chem3.Keq0_7*1[m^3/(s*mol)])	1/s	Reverse rate constant	Global	
chem3.r_7	chem3.kf_7*chem3.c_CO2*chem3.c_OH-chem3.kr_7*chem3.c_HCO3	mol/(m³·s)	Reaction rate	Global	
chem3.H_7	-chem3.h_CO2-chem3.h_OH+chem3.h_HCO3	J/mol	Enthalpy of reaction	Global	
chem3.S_7	-chem3.s_CO2-chem3.s_OH+chem3.s_HCO3	J/(mol·K)	Entropy of reaction	Global	
chem3.Q_7	-chem3.r_7*chem3.H_7	W/m³	Heat source of reaction	Global	

2.11.20 8: HCO3+OH<=>H2O+CO3

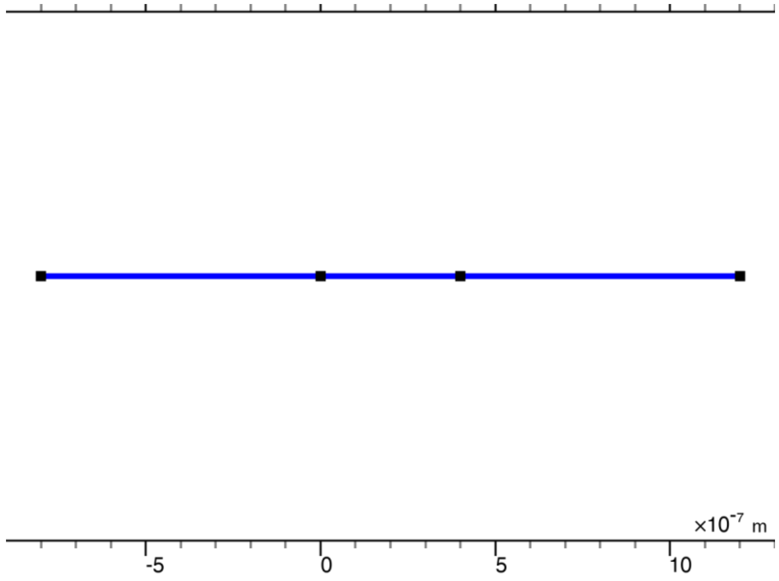

8: HCO3+OH<=>H2O+CO3

SELECTION

Geometric entity level	Domain
Selection	Geometry geom1: Dimension 1: All domains

Reaction Formula

SETTINGS

Description	Value
Formula	HCO3 + OH<=>H2O + CO3
Reaction type	Reversible

Reaction Rate

SETTINGS

Description	Value
Reaction rate	Mass action law
Description	Overall forward reaction order: 2 Overall reverse reaction order: 2

Rate Constants

SETTINGS

Description	Value
Specify equilibrium constant	On
Use Arrhenius expressions	Off
Forward rate constant	K_HCO3_OHkf

Equilibrium Settings

SETTINGS

Description	Value
Equilibrium constant	User defined
Equilibrium constant	K_HCO3/K_H2O*1e6

Reaction Thermodynamic Properties

SETTINGS

Description	Value
Enthalpy of reaction	Automatic
Entropy of reaction	Automatic
Heat source of reaction	Automatic

Variables

Name	Expression	Unit	Description	Selection	Details
chem3.Rsum_CO3	chem3.r_8	mol/(m³·s)	Reaction rate for species CO3	Global	+ operation
chem3.R_CO3	chem3.r_8	mol/(m³·s)	Reaction rate for species CO3	Global	+ operation
chem3.Rsum_H2O	0	mol/(m³·s)	Reaction rate for species H2O	Global	+ operation
chem3.Rsum_HCO3	-chem3.r_8	mol/(m³·s)	Reaction rate for species HCO3	Global	+ operation
chem3.R_HCO3	-chem3.r_8	mol/(m³·s)	Reaction rate for species HCO3	Global	+ operation
chem3.Rsum_OH	-chem3.r_8	mol/(m³·s)	Reaction rate for species OH	Global	+ operation
chem3.R_OH	-chem3.r_8	mol/(m³·s)	Reaction rate for species OH	Global	+ operation
chem3.Qheat	-chem3.r_8*chem3.H_8	W/m³	Heat source of reactions	Global	+ operation
chem3.R_H2O	0	mol/(m³·s)	Reaction rate for species H2O	Global	+ operation
chem3.kf_8	K_HCO3_OHkf	m³/(s·mol)	Forward rate constant	Global	
chem3.Keq0_8	1000000*K_HCO3/K_H2O		Equilibrium constant	Global	
chem3.kr_8	chem3.kf_8/chem3.Keq0_8	m³/(s·mol)	Reverse rate constant	Global	

Name	Expression	Unit	Description	Selection	Details
chem3.r_8	chem3.kf_8*chem3.c_HCO3*chem3.c_OH-chem3.kr_8*chem3.c_H2O*chem3.c_CO3	mol/(m³·s)	Reaction rate	Global	
chem3.H_8	-chem3.h_HCO3-chem3.h_OH+chem3.h_H2O+chem3.h_CO3	J/mol	Enthalpy of reaction	Global	
chem3.S_8	-chem3.s_HCO3-chem3.s_OH+chem3.s_H2O+chem3.s_CO3	J/(mol·K)	Entropy of reaction	Global	
chem3.Q_8	-chem3.r_8*chem3.H_8	W/m³	Heat source of reaction	Global	

2.12 MULTIPHYSICS

2.12.1 Potential Coupling 1

USED PRODUCTS

COMSOL Multiphysics

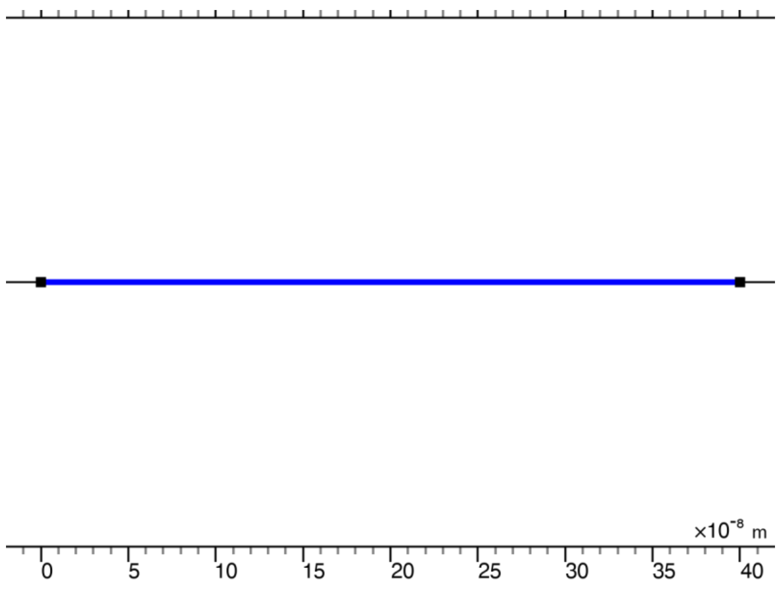

Potential Coupling 1

SELECTION

Geometric entity level	Domain
Selection	Geometry geom1: Dimension 1: All domains

Coupled Interfaces

SETTINGS

Description	Value
Source	Electrostatics (es)
Destination	Transport of Diluted Species (tds)

Variables

Name	Expression	Unit	Description	Selection
pc1.V	V	V	Electric potential	Domain 2

2.12.2 Space Charge Density Coupling 1

USED PRODUCTS

COMSOL Multiphysics

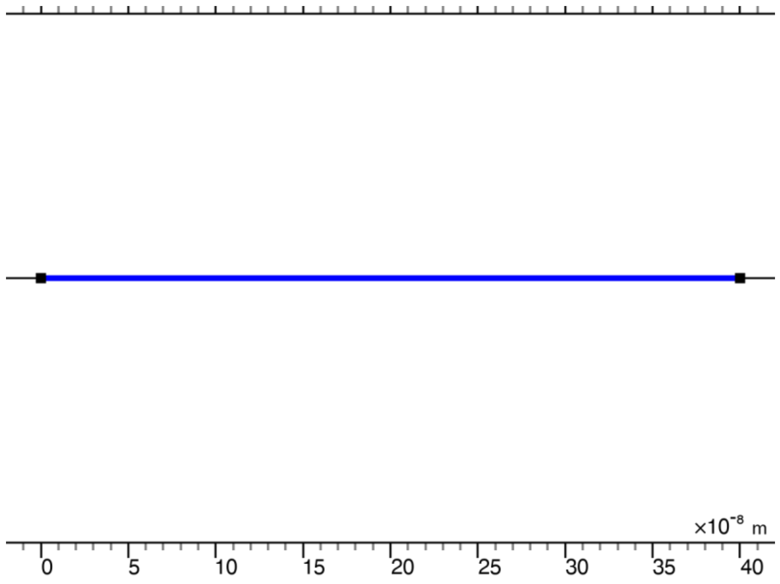

Space Charge Density Coupling 1

SELECTION

Geometric entity level	Domain
Selection	Geometry geom1: Dimension 1: All domains

EQUATIONS

$$\nabla \cdot \mathbf{D} = \rho_v, \quad \rho_v = F\sum_i z_i c_i$$

Coupled Interfaces

SETTINGS

Description	Value
Source	Transport of Diluted Species (tds)
Destination	Electrostatics (es)

Variables

Name	Expression	Unit	Description	Selection	Details
scdc1.sumzc	tds.z_cAsc*cAsc+tds.z_cAscm*cAscm+tds.z_cCO2*cCO2+tds.z_cCO*cCO+tds.z_cO2*cO2+tds.z_cH2O*cH2O+tds.z_cH2PO4*cH2PO4+tds.z_cHPO4*cHPO4+tds.z_cNa*cNa+tds.z_cCl*cCl+tds.z_cH3PO4*cH3PO4+tds.z_cPO4*cPO4+tds.z_cH*cH+tds.z_cOH*cOH+tds.z_cHCO3*cHCO3+tds.z_cCO3*cCO3	mol/m³	Help variable	Domain 2	+ operation
scdc1.rhoq	F_const*scdc1.sumzc	C/m³	Space charge density	Domain 2	
scdc1.sumztfluxx	tds.z_cAsc*tds.tflux_cAscx+tds.z_cAscm*tds.tflux_cAscmx+tds.z_cCO2*tds.tflux_cCO2x+tds.z_cCO*tds.tflux_cCOx+tds.z_cO2*tds.tflux_cO2x+tds.z_cH2O*tds.tflux_cH2Ox+tds.z_cH2PO4*tds.tflux_cH2PO4x+tds.z_cHPO4*tds.tflux_cHPO4x+tds.z_cNa*tds.tflux_cNax+tds.z_cCl*tds.tflux_cClx+tds.z_cH3PO4*tds.tflux_cH3PO4x+tds.z_cPO4*tds.tflux_cPO4x+tds.z_cH*tds.tflux_cHx+tds.z_cOH*tds.tflux_cOHx+tds.z_cHCO3*tds.tflux_cHCO3x+tds.z_cCO3*tds.tflux_cCO3x	mol/(m²·s)	Help variable, x component	Domain 2	+ operation
scdc1.sumztfluxy	tds.z_cAsc*tds.tflux_cAscy+tds.z_cAscm*tds.tflux_cAscmy+tds.z_cC	mol/(m²·s)	Help variable, y component	Domain 2	+ operation

Name	Expression	Unit	Description	Selection	Details
	O2*tds.tflux_cCO2y+tds.z_cCO*tds.tflux_cCOy+tds.z_cO2*tds.tflux_cO2y+tds.z_cH2O*tds.tflux_cH2Oy+tds.z_cH2PO4*tds.tflux_cH2PO4y+tds.z_cHPO4*tds.tflux_cHPO4y+tds.z_cNa*tds.tflux_cNay+tds.z_cCl*tds.tflux_cCly+tds.z_cH3PO4*tds.tflux_cH3PO4y+tds.z_cPO4*tds.tflux_cPO4y+tds.z_cH*tds.tflux_cHy+tds.z_cOH*tds.tflux_cOHy+tds.z_cHCO3*tds.tflux_cHCO3y+tds.z_cCO3*tds.tflux_cCO3y				
scdc1.sumztfluxz	tds.z_cAsc*tds.tflux_cAscz+tds.z_cAscm*tds.tflux_cAscmz+tds.z_cCO2*tds.tflux_cCO2z+tds.z_cCO*tds.tflux_cCOz+tds.z_cO2*tds.tflux_cO2z+tds.z_cH2O*tds.tflux_cH2Oz+tds.z_cH2PO4*tds.tflux_cH2PO4z+tds.z_cHPO4*tds.tflux_cHPO4z+tds.z_cNa*tds.tflux_cNaz+tds.z_cCl*tds.tflux_cClz+tds.z_cH3PO4*tds.tflux_cH3PO4z+tds.z_cPO4*tds.tflux_cPO4z+tds.z_cH*tds.tflux_cHz+tds.z_cOH*tds.tflux_cOHz+tds.z_cHCO3*tds.tflux_cHCO3z+tds.z_cCO3*tds.tflux_cCO3z	mol/(m²·s)	Help variable, z component	Domain 2	+ operation
scdc1.Ilx	F_const*scdc1.sumztfluxx	A/m²	Electrolyte current density vector, x component	Domain 2	
scdc1.Ily	F_const*scdc1.sumztfluxy	A/m²	Electrolyte current density vector, y	Domain 2	

Name	Expression	Unit	Description	Selection	Details
			component		
scdc1.Ilz	F_const*scdc1.sumztfluxz	A/m²	Electrolyte current density vector, z component	Domain 2	
scdc1.sumzntflux	tds.z_cAsc*tds.ntflux_cAsc+tds.z_cAscm*tds.ntflux_cAscm+tds.z_cCO2*tds.ntflux_cCO2+tds.z_cCO*tds.ntflux_cCO+tds.z_cO2*tds.ntflux_cO2+tds.z_cH2O*tds.ntflux_cH2O+tds.z_cH2PO4*tds.ntflux_cH2PO4+tds.z_cHPO4*tds.ntflux_cHPO4+tds.z_cNa*tds.ntflux_cNa+tds.z_cCl*tds.ntflux_cCl+tds.z_cH3PO4*tds.ntflux_cH3PO4+tds.z_cPO4*tds.ntflux_cPO4+tds.z_cH*tds.ntflux_cH+tds.z_cOH*tds.ntflux_cOH+tds.z_cHCO3*tds.ntflux_cHCO3+tds.z_cCO3*tds.ntflux_cCO3	mol/(m²·s)	Help variable	Boundaries 2–3	+ operation
scdc1.nIl	F_const*scdc1.sumzntflux	A/m²	Normal electrolyte current density	Boundaries 2–3	
es.rhoq	scdc1.rhoq	C/m³	Space charge density	Domain 2	+ operation

2.13 MESH 1

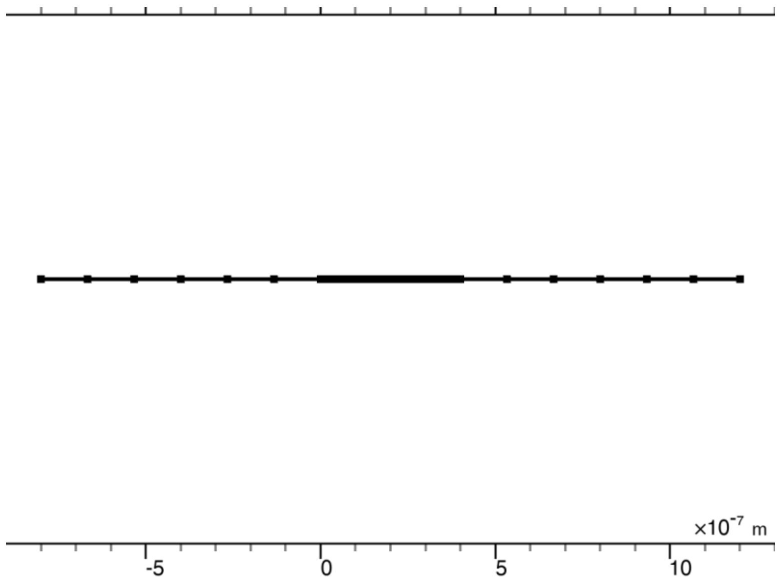

Mesh 1

MESH STATISTICS

Description	Value
Status	Complete mesh
Mesh vertices	513
Edge elements	512
Vertex elements	4
Number of elements	512
Minimum element quality	1
Average element quality	1
Element length ratio	2.9272E-4
Mesh length	2E-6 m

2.13.1 Size (size)

SETTINGS

Description	Value
Maximum element size	1.34E-7
Minimum element size	6.0E-10
Curvature factor	0.3
Maximum element growth rate	1.3

2.13.2 Edge 1 (edg1)

SELECTION

Geometric entity level	Domain
Selection	Remaining

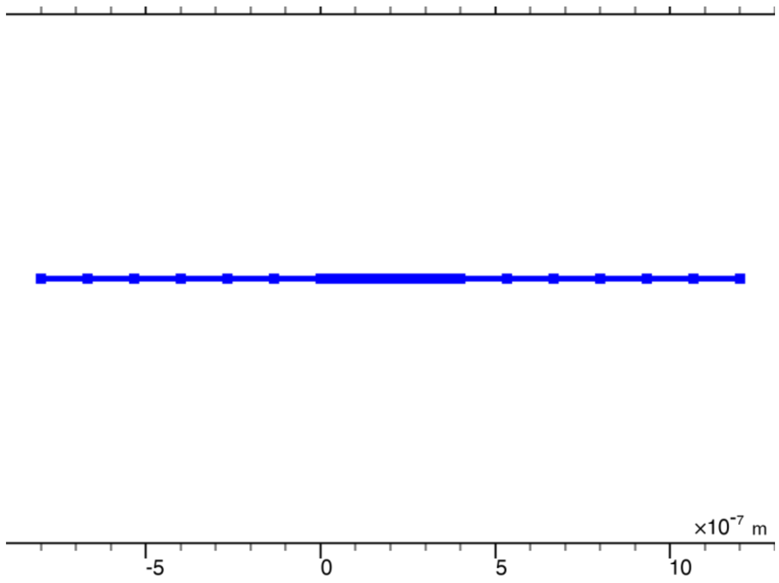

Edge 1

SETTINGS

Description	Value
Last build time	0
Built with	COMSOL 6.0.0.354 (win64)|2022 - 04 - 04T09:03:17.346357600

Distribution 1 (dis1)

SELECTION

Geometric entity level	Domain
Selection	Geometry geom1: Dimension 1: Domain 2

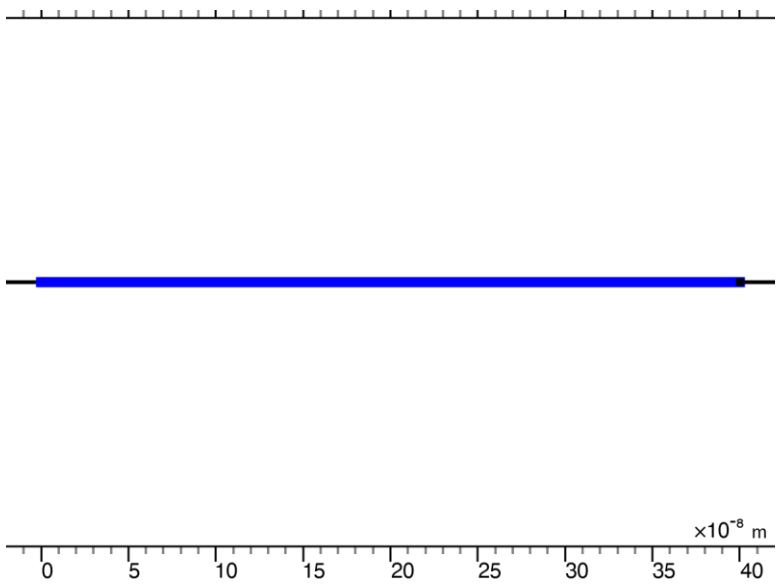

Distribution 1

SETTINGS

Description	Value
Distribution type	Predefined
Number of elements	250*round(L/200e-9)
Element ratio	40
Symmetric distribution	On

2.13.3 Edge 2 (edg2)

SELECTION

Geometric entity level	Remaining

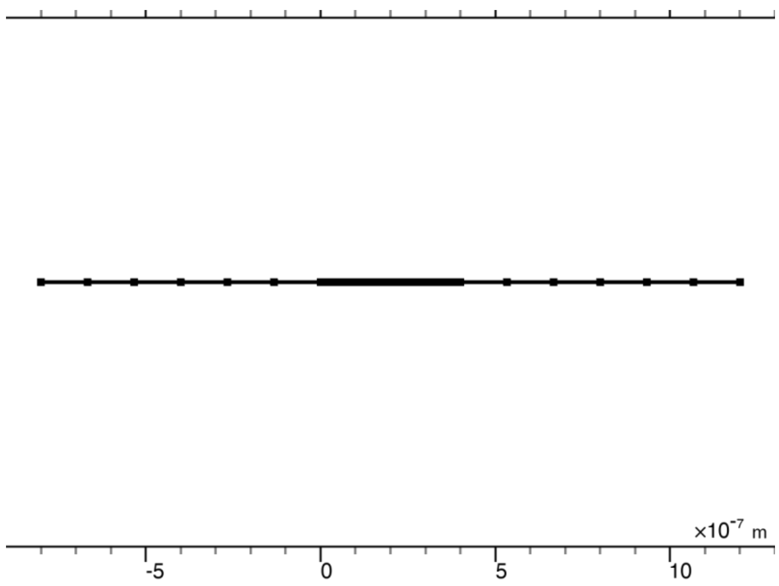

Edge 2

SETTINGS

Description	Value
Last build time	0
Built with	COMSOL 6.0.0.354 (win64)|2022 - 04 - 04T09:03:17.362332400

Distribution 1 (dis1)

SELECTION

Geometric entity level	Domain
Selection	Geometry geom1: Dimension 1: Domains 1, 3

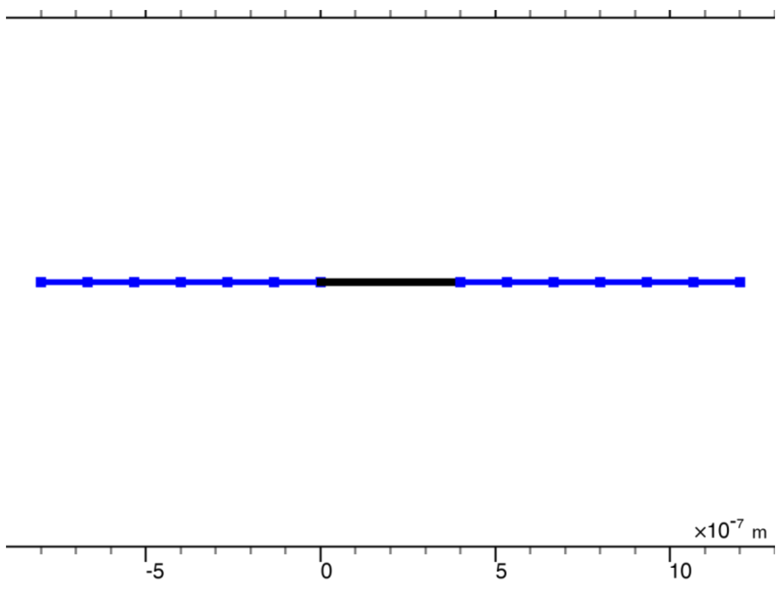

Distribution 1

SETTINGS

Description	Value
Distribution type	Predefined
Number of elements	10*round(L/200e-9)
Symmetric distribution	On
